# Identification of rhizome-specific genes by genome-wide differential expression Analysis in *Oryza longistaminata*

**DOI:** 10.1186/1471-2229-11-18

**Published:** 2011-01-24

**Authors:** Fengyi Hu, Di Wang, Xiuqin Zhao, Ting Zhang, Haixi Sun, Linghua Zhu, Fan Zhang, Lijuan Li, Qiong Li, Dayun Tao, Binying Fu, Zhikang Li

**Affiliations:** 1 Institute of Crop Sciences/National Key Facility for Crop Gene Resources and Genetic Improvement, Chinese Academy of Agricultural Sciences, 12 South Zhong-Guan-Cun St., Beijing 100081, China; 2 Food Crops Research Institute, Yunnan Academy of Agricultural Sciences, Kunming 650205, China; 3 College of Life Sciences, Wuhan University, 430072, China; 4 Institute of Genetics and Developmental Biology, Chinese Academy of Sciences, Beijing 100101, China; 5 International Rice Research Institute, DAPO Box 7777, Metro Manila, the Philippines

## Abstract

**Background:**

Rhizomatousness is a key component of perenniality of many grasses that contribute to competitiveness and invasiveness of many noxious grass weeds, but can potentially be used to develop perennial cereal crops for sustainable farmers in hilly areas of tropical Asia. *Oryza longistaminata*, a perennial wild rice with strong rhizomes, has been used as the model species for genetic and molecular dissection of rhizome development and in breeding efforts to transfer rhizome-related traits into annual rice species. In this study, an effort was taken to get insights into the genes and molecular mechanisms underlying the rhizomatous trait in *O. longistaminata *by comparative analysis of the genome-wide tissue-specific gene expression patterns of five different tissues of *O. longistaminata *using the Affymetrix GeneChip Rice Genome Array.

**Results:**

A total of 2,566 tissue-specific genes were identified in five different tissues of *O. longistaminata*, including 58 and 61 unique genes that were specifically expressed in the rhizome tips (RT) and internodes (RI), respectively. In addition, 162 genes were up-regulated and 261 genes were down-regulated in RT compared to the shoot tips. Six distinct *cis*-regulatory elements (CGACG, GCCGCC, GAGAC, AACGG, CATGCA, and TAAAG) were found to be significantly more abundant in the promoter regions of genes differentially expressed in RT than in the promoter regions of genes uniformly expressed in all other tissues. Many of the RT and/or RI specifically or differentially expressed genes were located in the QTL regions associated with rhizome expression, rhizome abundance and rhizome growth-related traits in *O. longistaminata *and thus are good candidate genes for these QTLs.

**Conclusion:**

The initiation and development of the rhizomatous trait in *O. longistaminata *are controlled by very complex gene networks involving several plant hormones and regulatory genes, different members of gene families showing tissue specificity and their regulated pathways. Auxin/IAA appears to act as a negative regulator in rhizome development, while GA acts as the activator in rhizome development. Co-localization of the genes specifically expressed in rhizome tips and rhizome internodes with the QTLs for rhizome traits identified a large set of candidate genes for rhizome initiation and development in rice for further confirmation.

## Background

Rhizomes are horizontal, underground plant stems and the primary energy storage organ of many perennial grass species. As the primary means of propagation and dispersal, rhizomes play a key role in the persistence of many perennial grasses [1]. In agriculture, rhizomes have two contrasting roles. On one hand, strong rhizomes are a desirable trait for many species of turf and forage grasses. On the other hand, strong rhizomes are a negative trait contributing to the competitiveness and invasiveness of many grasses which are noxious weeds in crop fields [2].

In many mountainous areas where people depend upon annual crops for subsistence, development and cultivation of perennial crop cultivars with strong rhizomes have been proposed as an environmentally sound and economically viable alternative for use and protection of the fragile rainfed ecosystems [3-5]. For example, upland rice is grown annually in many steep hillsides of tropical Asia as the primary food crop for sustainable farmers. But growing upland rice in the hilly areas often causes severe soil erosion and damages the ecosystem in these areas. Thus, breeding perennial upland rice varieties with strong rhizomes could be an effective way to resolve this problem because rhizomes of a perennial cultivar would trap soil and minimize soil disturbance associated with annual tillage.

As the staple food for more than half of the world's population, rice (*Oryza sativa *L.) is the model system for genetic and genomic studies of grasses. Of the two cultivated and 22 wild species of rice, *O. longistaminata *from Africa is the only wild perennial species that has both strong rhizomatous stems and the same AA genome as *O. sativa *[6,7]. Thus, *O. longistaminata *provides a model system for genetic and molecular dissection of the rhizomatous trait in grasses. Previous genetic studies have shown that rhizome expression in *O. longistaminata *is controlled either by two complementary lethal genes, *D1 *and *D2 *[8,9], or by a single major gene loosely linked to the *lg *locus on chromosome 4 plus several modifying genes [10]. Using an F_2 _and two backcross populations derived from crosses between an *O. longistaminata *accession and an *O. sativa *line, RD23, Hu et al. (2003) reported that the rhizome expression in *O. longistaminata *is controlled by two dominant-complementary genes, *Rhz2 *and *Rhz3 *on rice chromosome 3 and 4 [11]. Comparative analysis further revealed that each gene closely corresponds to a major QTL controlling rhizome expression in *Sorghum propinquum*. Many additional QTLs affecting abundance of rhizomes in *O. longistaminata *were also identified, and found to correspond to the locations of the rhizome-controlling QTLs in *S. propinquum *[11]. All these results provided the basis for cloning genes related to the rhizomatous traits in rice.

Because plant rhizomes and tillers both originate from axillary buds on the most basal portion of the seedling shoot [12], genes controlling plant axillary bud initiation and outgrowth may also contribute to rhizome development and growth. Several genes involved in rice axillary bud initiation or outgrowth have been cloned. *MONOCULM1 *(*MOC1*), a member of the GRAS transcription factor family, is the first cloned gene which is involved in the axillary bud initiation and tiller outgrowth in rice [13]. The second one is O*sTB1 *which acts as a negative regulator controlling tiller outgrowth in rice [14]. Two other genes, *LAX *and *SPA*, were identified as the main regulators of the axillary meristem formation in rice [15] and *LAX1 *function is required for all types of axillary meristems at both the vegetative and reproductive phases of rice [16]. Recently, the *DWARF *gene was reported to be functionally involved in tiller bud outgrowth [17]. Although the functions of these genes and molecular mechanisms in rice tiller development have largely been characterized, it remains to be elucidated whether the molecular mechanism controlling rhizome initiation and elongation is parallel to that of the tiller development.

With the availability of the whole genome sequence in rice [18], several rice genome arrays have been developed by Affymetrix, Agilent, NSF, Yale University and BGI [19-23]. These DNA microarrays have been used for many purposes, especially for genome-wide transcriptome analyses in different cells/tissues/organs or developmental stages of rice. Previously, different research groups have shown that the rice cell transcriptome exhibits both qualitative and quantitative differences consistent with the specialized functions of different cell types [24], and unique gene sets are exclusively expressed in different tissues/organs at different developmental stages of rice [25-28]. Using a cDNA macroarray, a set of genes and their *cis*-elements motifs with rhizome-enriched expression were identified in sorghum [2]. Comparative analysis showed that many of these highly expressed sorghum rhizome genes were aligned to the previously identified rhizome-related QTL regions in rice and sorghum, providing an important basis for further molecular dissection of rhizome development in grasses.

Following our previous study in genetic dissection of rhizomatousness in *O. longistaminata*, we report here an effort to understand the molecular mechanisms of tissue specificity in *O. longistaminata *by exploring the genome-wide gene expression patterns. Our results provide insights into the genes and molecular mechanisms underlying the rhizomatousness in *O. longistaminata*.

## Results

### Global changes of gene expression in five different tissues

Rhizomes, which are underground stems, are expected to be closely related developmentally to aboveground stems. In this study, of the five different tissues, rhizome tips (RT) and rhizome internodes (RI) were chosen because they are known to contain tissue-specifically expressed genes responsible for rhizome development and growth [2], whereas shoot tips (ST), shoot internodes (SI) were chosen to represent cells at a later stage of development, and young leaves (YL) to establish the activity of housekeeping genes unrelated to rhizome- and stem-specific development. Thus, comparisons between expressed genes from different tissues allow us to discover specific sets of genes responsible for rhizome development and growth.

**Figure 1:**
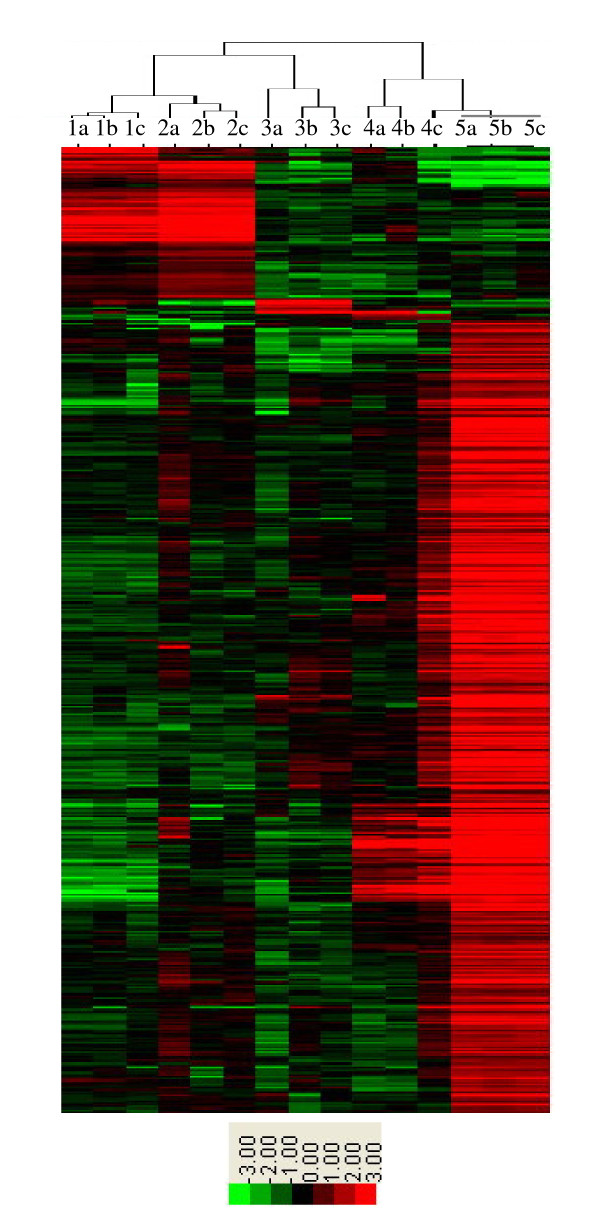
**Dendrogram of 2566 tissue-specifically expressed genes in the five tissues of *O. longistaminata***. 1. Rhizome tips, 2. Shoot tips, 3. Rhizome internodes, 4. Stem internodes, 5. Young leaves. The suffixes a, b, and c indicate the three biological repeats. In the color panels, each horizontal line represents a single gene and the color of the line shows the expression level of the gene relative to the median in a specific sample: high expression in red, low expression in green. The row data represented here is provided in Additional file 2. Results from the three replicates of the microarray experiments were consistent, indicating the consistency of the gene expression patterns in the five sampled tissues. Two subsets of genes are apparent. Rhizome tips (labeled 1) and shoot tips (labeled 2) show high expression of genes near the top of the panel and moderate or low expression of genes below, while leaves (labeled 5) show low or moderate expression of genes near the top of the panel and high expression of genes below. Rhizome internodes (labeled 3) and stem internodes (labeled 4) show moderate or low expression of both subsets. The difference between rhizomes and shoots appears small in comparison with the difference between tips and internodes of both organs.

The microarray experiments identified a total of 21,372 genes that were expressed in at least one of the five sampled tissues of *O. longistaminata*, including 16,981 genes expressed in RT, 15,662 genes expressed in RI, 16026 genes expressed in ST, 15,732 genes expressed in SI, and 15,294 genes expressed in YL. These include 10,801 genes that were expressed in all five tissues, and 2,566 genes that were specifically expressed in only one of the five tissues (Additional files 1, 2). The two tip tissues (RT and ST) had similar genome expression patterns, and so did the two internode tissues (RI and SI). The greatest difference in expression pattern was observed between the tip tissues and YL (Figure 1 and Additional file 1).

### The tissue-enriched genes in five tissues in O. longistaminata and their inferred functions

Multiclass analyses and Wilcoxon Rank-Sum tests of the expression data led us to the identification of a total of 2,566 tissue-specific genes, including 58, 61, 299, 29 and 1,974 unique genes specifically enriched in RT, RI, ST, SI and YL, respectively (Table 1, Additional files 2, 3, 4, 5, 6). These tissue-specifically expressed genes represent the most important set of genes that determine the specificities and functions of the five sampled tissues. As expected, genes specifically expressed in each tissue have inferred functions strongly related to the known functions of the corresponding tissues.

**Table 1:** The list of genes specifically enriched in the rhizome tips relative to other tissues

Probe Name	OsGI	Function Annotation	*q*-value %	RT/ST	RT/RI	RT/SI	RT/YL
Os.34982.1.A1_at	Os04g17660	Rhodanese-like domain containing protein	0.003	2.60	23.33	7.82	167.88
Os.8120.1.S1_at	Os04g33570	CEN-like protein 2	<0.001	1.77	14.03	8.51	42.22
Os.49726.1.S1_at	Os11g05470	CEN-like protein 3	0.029	2.24	5.13	6.04	66.16
Os.8203.1.S1_at	Os10g05750	proline-rich protein	<0.001	1.77	12.97	19.60	105.02
Os.21805.1.S1_s_at	Os06g51320	Gibberellin regulated protein, expressed	0.046	3.62	4.94	7.33	8.44
Os.2367.1.S1_at	Os03g21820	Alpha-expansin 10 precursor	<0.001	3.19	12.74	7.64	28.19
OsAffx.15319.1.S1_at	Os06g08830	UDP-glucoronosyl and UDP-glucosyl transferase	0.975	1.60	1.85	2.03	1.65
Os.50483.1.S1_at	Os04g42860	GDSL-like Lipase/Acylhydrolase family protein	0.003	2.19	2.84	26.46	60.78
Os.8666.1.S1_at	Os02g57110	GDSL-like Lipase/Acylhydrolase family protein	<0.001	1.72	14.10	11.25	14.40
OsAffx.15187.1.S1_at	Os05g50960	Polygalacturonase family protein	0.003	1.61	2.60	1.85	73.35
Os.17076.1.S1_at	Os09g10340	Cytochrome P450 family protein	<0.001	3.63	14.96	6.62	19.18
Os.49861.1.S1_at	Os04g04330	Leucine Rich Repeat family protein	0.003	3.07	3.12	2.21	9.92
Os.15219.1.S1_at	Os06g11320	peptidyl-prolyl cis-trans isomerase	<0.001	4.23	13.23	16.75	27.63
Os.15454.2.S1_at	Os06g06760	U-box domain containing protein	0.003	4.04	14.32	9.09	44.53
Os.15789.1.S1_at	Os12g08920	Peroxidase 43 precursor	0.019	3.66	6.61	16.15	18.90
Os.53726.1.S1_at	Os07g05370	protein kinase family protein	0.013	2.14	6.81	3.04	57.21
Os.5682.1.S1_at	Os09g30320	BURP domain containing protein	0.006	2.08	2.37	2.48	2.85
Os.8655.1.S1_at	Os06g31960	Plant thionin family protein	<0.001	1.72	16.42	8.35	53.07
OsAffx.17468.1.S1_s_at	Os08g42080	ACT domain containing protein	<0.001	1.60	7.34	7.73	4.92
Os.33336.1.S1_at	Os01g11350	bZIP transcription factor family protein	0.003	2.97	16.06	4.16	30.68
OsAffx.2611.1.S1_at	Os02g14910	bZIP transcription factor family protein	<0.001	1.53	7.98	7.43	14.36
Os.28450.1.S1_at	Os01g70730	flowering promoting factor-like 1	0.003	4.81	3.12	7.95	5.34
Os.6271.1.S1_at	Os07g39320	Homeobox domain containing protein	0.069	1.95	2.51	2.54	4.83
Os.9086.1.S1_at	Os03g10210	Homeobox domain containing protein	0.003	2.21	1.63	3.59	19.50
Os.10050.1.S1_at	Os01g62660	Myb-like DNA-binding domain	0.003	14.12	15.62	15.82	271.93
Os.12994.1.S1_at	Os12g38400	Myb-like DNA-binding domain containing protein	<0.001	25.60	9.56	41.36	82.54
Os.47323.1.S1_at	Os02g45570	transcription activator	0.270	3.09	2.40	2.88	10.09
Os.49711.1.S1_at	Os08g35110	auxin-responsive protein	<0.001	2.27	11.19	12.76	18.16
Os.13012.1.S1_at	Os03g49880	TCP family transcription factor containing protein	<0.001	8.88	9.27	22.59	45.56
Os.151.1.S1_x_at	Os03g51690	Homeobox protein OSH1	<0.001	5.12	13.95	15.18	22.66
Os.54612.1.A1_at	Os02g07310	Piwi domain containing protein	0.644	2.09	3.48	2.53	4.67
Os.33534.1.S1_s_at	Os07g06620	YABBY protein	0.046	2.97	3.04	11.15	101.27
Os.4174.1.S1_at	Os08g02070	Agamous-like MADS box protein AGL12	0.003	2.42	20.57	6.84	8.35
Os.11344.1.S1_s_at	Os05g48040	MATE efflux family protein	<0.001	10.12	13.69	12.84	45.27
Os.28462.1.S1_s_at	Os12g02290	Nonspecific lipid-transfer protein 5 precursor	<0.001	3.08	21.75	17.68	60.66
Os.54305.1.S1_at	Os06g12610	Auxin efflux carrier component 1	<0.001	2.11	6.12	4.90	14.89
Os.14955.1.S1_at	Os03g31730	expressed protein	0.003	8.31	17.88	12.88	57.28
Os.15725.1.S1_at	Os03g64050	expressed protein	0.029	3.71	3.83	5.88	3.67
Os.22569.1.S1_at	Os03g30740	expressed protein	0.003	3.89	3.40	4.57	8.44
Os.27641.1.A1_at	Os04g23140	expressed protein	0.006	3.18	3.76	4.31	3.35
Os.3496.1.S1_at	Os01g12110	expressed protein	0.006	2.87	5.95	3.63	11.49
Os.47356.1.A1_at	Os10g31930	expressed protein	0.011	2.27	4.40	4.46	11.80
Os.8682.1.S1_a_at	Os10g08780	expressed protein	<0.001	1.95	1.68	3.24	6.41
Os.8682.2.S1_x_at	Os10g08780	expressed protein	0.013	1.63	2.96	3.23	2.53
OsAffx.11145.1.S1_s_at	Os01g21590	expressed protein	0.139	1.82	1.77	1.61	1.83
OsAffx.28068.1.S1_at	Os06g42730	expressed protein	<0.001	1.52	1.75	2.20	5.51
OsAffx.30149.1.S1_s_at	Os09g36160	expressed protein	<0.001	1.51	4.94	3.72	14.06
Os.9836.1.S1_at	Os11g10590	hypothetical protein	0.003	1.62	4.21	3.15	61.66
Os.28030.2.A1_at	Os06g0696400	Xyloglycan endo-transglycosylase precursor	0.003	3.15	6.76	6.45	29.74
Os.57006.1.S1_at	Os09g0459200	Conserved hypothetical protein	<0.001	1.99	12.54	11.69	56.03
Os.7285.1.S1_at	Os05g0518600	SL-TPS/P	<0.001	1.91	2.67	6.60	2.21
Os.7317.2.S1_at	Os01g0914300	Plant lipid transfer domain containing protein	0.011	1.88	3.24	8.35	8.22
Os.7431.1.S1_a_at	Os04g0272700	UDP-glucuronosyl/UDP-glucosyltransferase	0.006	1.87	5.92	3.91	5.73
Os.7567.1.S1_at	Os10g0554800	Plant lipid transfer domain containing protein	0.003	1.84	4.24	6.96	13.89
Os.7575.1.S1_at	Os04g0619800	Conserved hypothetical protein	0.106	1.90	2.57	1.83	4.64
Os.9167.1.A1_at	Os06g0649600	Non-protein coding transcript	0.011	1.62	7.47	3.17	21.16
OsAffx.22476.1.S1_x_at	Os07g0160100	YABBY2	<0.001	1.59	2.46	2.69	299.82
OsAffx.27291.1.S1_at	Os05g43440	DNA-binding protein	<0.001	1.53	1.96	2.23	222.60

***Note***: RT/ST, RT/RI, RT/SI, and RT/YL indicate ratio of signal1 (RT)/signal2 (ST, RI, SI, and YL) from Wilcoxon Rank-Sum tests, respectively.

YL has 1974 tissue-specially expressed genes, far more than the other tissues (Additional file 6). This is not surprising since plant leaves contain the primary machinery for photosynthesis. As expected, most of these YL enriched genes were related to photosynthesis, metabolism, transport, signal transduction, etc, of known physiological functions of leaves. These included genes encoding photosystem I and II components, the PGR5 protein involved in cyclic electron flow around photosystem I and essential for photoprotection [29], *RPT2 *(a signal transducer involved in phototropic response and stomata opening) [30], ZEITLUPE and early flowering proteins related to the circadian clock function and early photomorphogenesis [31,32] and AS2, a protein required for the formation of a symmetric flat leaf lamina [33].

In ST, the 299 specifically enriched genes were mainly functionally classified as cell cycle, cell wall components and biogenesis, DNA replication and repairing, signal transduction, and transcriptional regulation involved in shoot morphogenesis (additional file 4). These included 60 genes encode transcription factor proteins, such as TCP (*Os03g57190*), FL (*Os04g51000*), *OsSBP5*, and a growth regulating factor (*Os06g02560*), which are reported to be involved in the regulation of shoot apical meristem activities and morphogenesis of shoot organs [34-37]. Of particular interest are four genes (*OsARF2*, *OsARF8*, *OsARF-GAP*, and *Auxin efflux carrier component 3*) that are implicated in the auxin responses and have effects on shoot growth and development [38]. Two genes encoding PINHEAD proteins were also ST-enriched, which are involved in the fate determination of central shoot meristem cells [39,40].

Most of the 29 SI-enriched genes encode proteins of unknown function, but a few are inferred to be related to metabolism, signal transduction, and redox regulation (Additional file 5). Of these, a BCL-2 binding anthanogene-1 gene reportedly has functions in regulating development and apoptosis-like processes during pathogen attack and abiotic stress [41]. Another gene of interest encodes the cytokinin synthase involved in the biosynthesis of cytokinin [42].

Of the 61 RI-enriched genes (Additional file 3), 11 encode proteins with transport functions, including three proteins containing heavy-metal-associated domains, a transmembrane amino acid transporter; 7 proteins related to cell cycle and cell wall biogenesis (including a dirigent-like protein, a glycine rich protein and a pectinesterase inhibitor-domain containing protein), and one gene encoding a flavin-binding monooxygenase-like family protein which has the inferred function in auxin biosynthesis [43].

Of specific interest are the 58 RT-specifically expressed genes (Table 1). Of these, 15 are related to transcription regulation, including an agamous-like MADS box gene (*AGL12*), 2 *YABBY *genes (*Os07g06620 *and *Os07g0160100*), and a TCP gene (*Os03g49880*). Three genes encoding homeobox proteins such as *OSH1 *were of this group. Several genes with functionality in cell elongation and cell cycle, including *alpha-expansin 10*, *CEN2 *and *CEN3*, were also highly enriched in RT.

To confirm the microarray data, a set of 21 tissue-enriched genes were selected for RT-PCR analysis. The RT-PCR expression pattern of 18 out of the 21 genes was consistent with that of the microarray experiments (Additional file 7). The RT-PCR profiles of the remaining three genes failed to confirm the microarray results. This inconsistency was likely due to the difference between the two methods in detecting different members of gene families. Semi-quantitative RT-PCR detects the expression patterns of individual genes characterized by a single peak in the melting curve, while microarray analysis cannot distinguish different members of the same gene family.

### Comparison between the differentially expressed genes in RT and ST

**Figure 2:**
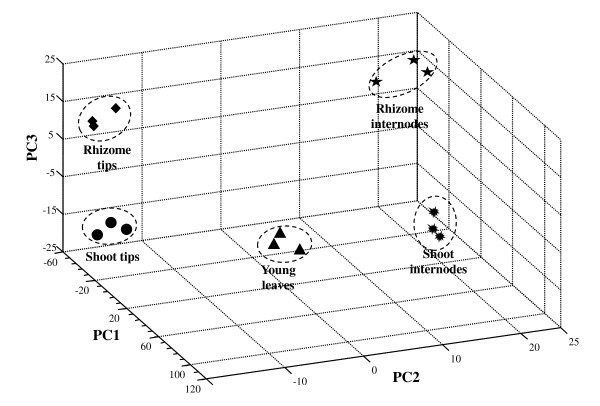
**The plot of the first principal components of the genome-wide gene expression profile of five tissues in *O. longistaminata *revealed by the microarray expression analysis**. PC1 is principal component 1, PC2 is principal component 2, and PC3 is principal component 3. Each type of tissue occupies a distinct location in the principal component space. PC1 separates leaves and shoot internodes from the other three organs. PC2 distinguishes among tips, internodes, and leaves. PC3 separates tips from internodes.

The principal components (PC) analysis based on the 10,801 genes that were expressed in all five tissues, which clearly differentiated the tissues from one another (Figure 2). Results from the three replicates of the microarray experiments were very consistent, indicating the high quality and consistency of the gene expression patterns in the five sampled tissues. Interestingly, PC1, which explained 63.7% of the total variation in expression level of this set of genes, did not contribute much to the differences between the five tissues. In contrast, PC2, which explained 17.5% of the expression variation of this set of genes, contributed greatly to the difference between RT/ST and RI, and between YL and SI, indicating that most genes contributing to PC2 are those differentiating leaves and internodes. PC3 explained 9.0% of the total expression variation of these genes and was primarily responsible for the difference between RT and ST. These results clearly indicate that there are significant quantitative differences in gene expression level among different tissues that contribute significantly to cell and tissue differentiation.

**Figure 3:**
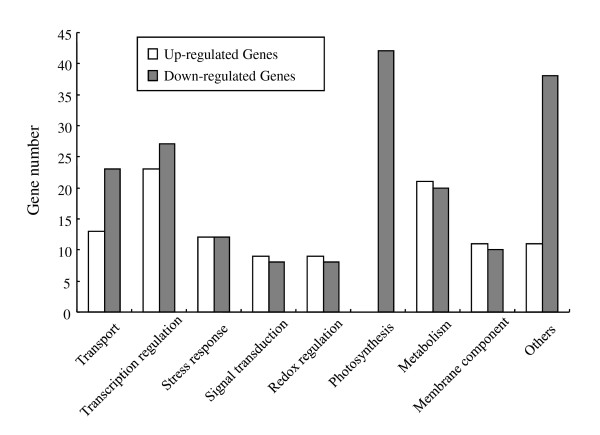
**Functional classification of the differentially expressed genes *O. longistaminata *with putative functions in the rhizome tips as compared with the shoot tips**. Up-regulated genes are shown in white bars, down-regulated genes in gray bars. Putative functions, taken from the Affymetrix annotation combined with the TIGR definition and NCBI database, are listed below the bars. Expression of genes involved in transport, transcription regulation, photosynthesis, and miscellaneous functions (labeled "others") is lower in rhizome tips than in shoot tips. Expression of genes involved in signal transduction, redox regulation, metabolism, and membrane components is higher in rhizome tips than in shoot tips.

Of the differentially expressed genes, 162 and 261 genes were up-regulated and down-regulated, respectively, in RT as compared to ST (Additional file 8). The function classification of all RT differentially expressed genes is shown in Figure 3. Many genes related to photosynthesis were greatly down-regulated and additional genes involved in transcription regulation and transport were repressed in RT. Of these, three auxin response-related genes were significantly down-regulated in RT as compared with ST. Several transcription factor genes related to shoot growth and development were also down-regulated in RT relative to ST (Additional file 7). These genes include TCP (*Os03g57190*), *SHOOT1*, *APETALA1, CONSTANS *(*Os04g42020*), *AGL19 *and a no-apical-meristem protein gene (*Os04g38720*). Among the down-regulated genes, several genes (*ARF8*, *Auxin Efflux Carrier 3*, *AS2*, and *SBP5*) with known functions were identified as ST-enriched ones.

The up-regulated genes in RT include those encoding two CEN-like proteins, two meiosis 5 proteins, two GA response proteins, and two auxin-responsive proteins. Also, the expression levels of two meiosis 5 protein genes (*Os06g35970 *and *Os02g13660*) were 8.0 and 14.0 times higher in RT than in ST. Twenty-four transcription factor genes encoding WRKY, NAC, bHLH, homeobox, flowering promoting factor-like 1, bZIP, AP2, and GBOF1 proteins, etc, were up-regulated. Seven genes encoding lipid transfer proteins (LTPs), which function as transporters, were highly up-regulated in the RT. In addition, five proline-rich protein (PRP) genes clustered on chromosome 10 were also up-regulated in RT relative to the ST.

### Identification of distinct cis-regulatory elements in the genes specifically expressed in particular tissues

Using the PLACE *cis*-element database, the *cis*-elements of the tissue-enriched genes were determined from both strands of their putative promoter sequences. We selected the top 65 genes from different gene sets for *cis-*element comparative analysis (Tables 2 and 3). Several distinct elements were found in significantly different proportions among different tissue-enriched gene sets (Table 2) and between RT up-regulated and down-regulated gene sets (Table 3).

**Table 2:** Four cis-elements abundant in genes specifically enriched in five tissues of *O. longistaminata *identified by bioinformatic analyses of the promoter regions of the genes involved.

Tissue type		RT	RI	ST	SI	YL
**No. of tested genes**		**56**	**57**	**61**	**27**	**64**
	**Total (%)**	**75.0 ± 11.3**	**98.2 ± 3.5 **^**a**^	**77.0 ± 10.6**	**77.8 ± 15.7**	**64.1 ± 11.8**
CGACG element (CGACG)	Single copy (%)	39.3	47.3	39.3	48.2	40.7
	Two or more copies (%)	35.7	50.9	37.7	29.6	23.4
	Total (%)	53.6 ± 13.1	73.7 ± 11.4^b^	59.0 ± 12.3	37.0 ± 18.2	39.1 ± 12.0
GCCCORE (GCCGCC)	Single copy (%)	39.3	45.6	24.6	29.6	25.0
	Two or more copies (%)	14.3	28.1	34.4	7.4	14.1
	Total (%)	98.2 ± 3.5 ^c^	78.9 ± 10.6	78.7 ± 10.3	88.9 ± 11.8 ^b^	78.1 ± 10.1
SURECOREATSULTR11 (GAGAC)	Single copy (%)	66.1	49.1	55.7	48.2	54.7
	Two or more copies (%)	32.1	29.8	23.0	40.7	23.4
	Total (%)	64.3 ± 12.5	86 ± 9.0 ^b^	83.6 ± 9.3 ^c^	66.7 ± 17.8	67.2 ± 11.5
Myb core (AACGG)	Single copy (%)	50.0	52.7	59.0	55.6	48.4
	Two or more copies (%)	14.3	33.3	24.6	11.1	18.8

^a ^The range about the average indicates 95% confidence limits for *p *among five treatments.

^b ^The range about the average indicates 95% confidence limits for *p *between RI and SI treatments.

^c ^The range about the average indicates 95% confidence limits for *p *between RT and ST treatments.

**Table 3:** Three c*is*-elements abundant in genes up-regulated and down-regulated in the rhizome tips (RT) of *O. longistaminata*

Gene set		RT Up-regulated	RT Down-regulated
**No. of tested genes**		**64**	**62**
	Total (%)	73.4 ± 11.6	91.9 ± 7.1*
CGACG element (CGACG)	Single copy (%)	31.2	37.1
	Two or more copies (%)	42.2	54.8
	Total (%)	82.8 ± 9.9*	58.1 ± 12.8
RY repeat (CATGCA)	Single copy (%)	50.0	42.0
	Two or more copies (%)	32.8	16.1
	Total (%)	96.9 ± 4.5*	79 ± 10.6
TAAAG motif (TAAAG)	Single copy (%)	59.4	43.5
	Two or more copies (%)	37.5	35.5

*The range about the average indicates 95% confidence limits for *p*.

Of the six tissue-enriched gene sets, a CGACG motif was the predominant *cis*-element in the RI-enriched genes relative to the other four tissues. This element was originally reported to function as a coupling element for the G box element [44]. An element of GCCGCC (GCCCORE, [45]) was found to be more abundant in RI than in SI. The SURECOREATSULTR11 element (GAGAC), which was reportedly conferring the sulfur deficiency response in Arabidopsis roots [46], showed significantly higher abundance in the RT than in other tissues. An AACGG (Myb core, [47]) element was enriched in RI and ST relative to the other tissues. Two additional *cis*-elements, the RY repeat (CATGCA, [48]) and TAAAG motif [49], were found to be significantly more abundant in the up-regulated genes set of RT as compared to other tissues.

### Co-localization of rhizome related QTLs and rhizome-specific expressed genes in rice and sorghum

In our previous study [11], we genetically identified the QTLs related to rhizome expression, abundance and growth related traits using an F_2 _population from the cross between RD23 and *Oryza longistaminata*. Sixteen QTLs were localized on 12 regions of the eight rice chromosomes that affected the nine rhizome traits. Of these, two dominant-complementary genes (*Rhz2 *and *Rhz3*) controlling the rhizomatous expression were mapped on chromosomes 3 and 4. Interestingly, many the RT- and RI-enriched genes and RT differentially regulated genes detected in the microarray experiments were mapped to the above-mentioned QTL likelihood intervals (Additional file 9).

Specifically, 34 of the RT- and RI-enriched genes were physically mapped on 11 rhizome-related QTL regions (Additional file 9). A gene encoding MATE-type transporter (*Os0311734*) associated with *Rhz2 *was highly repressed in RT relative to ST, while five RT up- or down-regulated genes were mapped on the *Rhz3 *region. Of these, a BADH gene (*Os0439020*) and a putative gene (*Os0436670*) of unknown function were highly up-regulated. Three other genes including a NAM transcription factor (*Os04g38720*) were down-regulated in RT. One gene encoding monosaccharide transporter 1 was down-regulated in RI as compared to SI. The homolog of this gene was also rhizome-specific expressed in sorghum [2]. Sixteen RT-specific expressed genes were identified in regions of five mapped QTLs (*QRn2*, *QRn3*, *QRn5*, *QRn6 *and *QRn10*) affecting rhizome number. Other positional candidate genes in these QTL regions include *MAP3K*, *Expansin S1*, *Hsp70*, *LTP1*, *SL-TPS/P*, and genes encoding gibberellin-regulated protein 2 (*Os06g51320*) and naringenin-chalcone synthase (*Os10g33370*). In the regions of three QTLs (*QRl1*, *QRl6 *and *QRl7*) controlling rhizome length, we identified nine RT-specific differentially regulated genes, which include a histone-like transcription factor (*Os07g41580*) and a homeodomain leucine zipper protein (*Os07g39320*).

We were able to align 26 of the rhizome-specific expressed genes on the sorghum genome using a comparative genomics tool, Phytozome v5.0 http://www.phytozome.net/, and found that 12 of these genes co-localize with the sorghum rhizome-related QTLs [1] (Additional file 9). All these genes will provide putative functional candidates for the identified rhizome-related QTLs and are worth of further study.

## Discussion

Annual upland rice grown in many hilly areas of tropical Asia provides essential food for poor sustainable farmers, but continuously growing this type of annual crops has caused severe soil erosion and environmental degradation in these areas [50]. Development of perennial grain crops with underground shoots (rhizomes) has been proposed as a vital alternative to solve the problem and to improve farm profitability in these areas [51]. Doing so requires full understanding of the genetic and molecular mechanisms underlying the growth and development of rhizomes, a key component of perenniality in many grass species. In this study, we used the Affymetrix oligomer microarray chips to profile the tissue-specific genome expression of *O. longistaminata *to discover and characterize genes and putative pathways responsible specifically for initiation and elongation of rhizomes in rice. As expected, we identified two distinct sets of genes that were differentially expressed in the two rhizome tissues. We realized that the Affymetrix oligomer microarray chips used in this study contain genes from *O. sativa*, but not from *O. longistaminata*. Thus, it is certain that some *O. longistaminata*-specific genes are missing in the chips and thus undetectable in this study. Nevertheless, the small set of rhizome specifically and differentially expressed genes detected in this study are, though incomplete, important in determining rhizome initiation and development in *O. longistaminata*. Detailed examination of the functions of this set of genes provides insights into molecular mechanisms associated with rhizome development and growth in *O. longistaminata.*

### Putative candidate genes for rhizome growth and development in O. longistaminata

RT is the most important tissue for rhizome development because they contain apical meristems consisting of pluripotent cells for rhizome initiation after embryogenesis. Thus, specifically and differentially expressed genes in RT are expected to be associated with early events in the rhizome development of *O. longistaminata *and thus are important candidates worthy of further study. Of particular interest is a group of regulatory genes that were highly enriched in RT. These include three homeobox genes of the *OSH1 *family, which is known to function as plant master regulators in the process of organ morphogenesis [52-54]. The TCP and YABBY genes of plant-specific transcription factor families are also important candidates, as they reportedly function in the development of plant lateral organs such as tiller initiation and elongation [36,55-57], suggesting the presence of overlapping regulatory mechanism(s) controlling plant underground rhizomes and aerial tillers. Additional candidates include *AGL12 *and *OsEXP10*. The former is known to be preferentially expressed in the primary root meristem and plays an important role in root development [58,59]. The latter is induced by GA and involved in cell elongation [60]. Two genes encoding CEN-like proteins 2 and 3 are also important candidates because they play distinct roles in regulating the activities of secondary meristem in the uppermost phytomeres [61].

### Genes with distinct expression patterns and functions differentiating RT and ST

Our results revealed very similar transcriptional programs between RT and ST. This is not surprising since the underground RT and aboveground ST are largely developed from homologous meristems [62]. However, a relatively small set of genes that were differentially expressed between RT and ST are of particular interest because they may have important molecular mechanism(s) for rhizomatousness in rice. For example, several auxin/IAA-related genes were greatly down-regulated in RT but highly enriched in ST. These include *ARF8 *and *Auxin Efflux Carrier 3 *which are known to play important roles in phytohormone signaling and control the activity of lateral meristems [63,64]. In contrast, several genes involved in GA biosynthesis were highly enriched in RT as compared to ST. These include genes encoding gibberellin 2-beta-dioxygenase (*Os01g55240*) and GA regulated protein (*Os06g51320*) [65]. These results suggest that auxin acts as a negative regulator in rhizome development and an activator for shoot growth, while GA acts as the activator in rhizome development. The suppression of genes encoding chlorophyll-binding and light-harvesting proteins for photosynthesis in RT was expected and consistent with the fact that the underground rhizomes do not have any functions in photosynthesis.

An interesting observation of this study was the significantly enhanced expression of genes in the gene families with "redundant" function(s) in RT. These include 2 CEN-like genes and 2 *Meiosis 5 *genes involved in apical meristem development [66], 5 genes encoding proline-rich proteins that are major components of plant cell walls [67,68], and seven lipid transfer proteins (LTPs) genes involved in cuticle synthesis and cell wall expansion [69]. All these results suggest that rhizome development tends to result from different members of large gene families with related but differentiated functions, consistent with a previous report that gene family members were frequently expressed with stage- or tissue-specific patterns [70].

### Important cis-regulatory elements in genes for rhizome development

In this study, several *cis*-elements were found overrepresented in one or more tissue-enriched gene sets. A core of sulfur-responsive element (SURE) containing an auxin response factor binding sequence [46] is enriched in RT-specifically expressed genes, suggesting that auxin may mediate gene regulation during rhizome development. Three *cis*-elements with motifs of CGACG, GCCGCC or AACGG were enriched in the 5' upstream regions of RI-enriched genes. These elements are involved in the cell cycle, jasminic acid (JA) responsiveness and sugar signaling [44,45,47], suggesting their possible functions in cell elongation, phytohormone regulation and metabolite regulation in the rhizome internodes. Two additional motifs, CATGCA and TAAAG, were in abundance in up- and down-regulated genes in RI and RT. The former was identified as an RY repeat in the RY/G-Box complex functioning in the abscisic acid (ABA) signaling pathway [48]. The latter was suggested as having a role for the Dof transcription factor in regulating guard cell-specific gene expression in ABA responsiveness [49,71]. All these results indicate that phytohormones such as auxin, JA and ABA play important roles in rhizome initiation and elongation, but details on how these phytohormones regulate rhizome initiation and elongation remains to be elucidated.

### QTL candidate genes associated with rhizome abundance and length

By aligning the functional candidate genes identified in the microarray analysis on the QTL regions associated with rhizome-related traits identified previously, we were able to identify a small number of QTL candidate genes for rhizomatousness in *O. longistaminata*. The most important one is a NAM transcription factor gene (*Oso4g38720*) in the *Rhz3 *interval, which was highly repressed in RT relative to ST. This kind of transcription factor gene is known to play crucial regulatory roles in rice growth and development. Importantly, the NAM proteins are involved in the formation of shoot apical meristem and lateral shoots [72]. Repressed expression of this gene in RT might reveal its negative regulation role in rhizome development. The *MAP3K *gene associated with *QRn2 *has been related to mediating the signal transduction of hormone and light, and required for regulating cell polarity and motility [73]. Enhanced activity of *MAP3K *in RT may be important to rhizome initiation as well as to the cell multiplication of rhizome apical meristem. The *Expansin S1 *on the *QRn3 *region is involved in enhancing growth by mediating cell wall loosening [74], so high abundance of Expansin S1 protein in RT should be responsible for rhizome elongation. LTPs are thought to function in lipid transfer between membranes as well as having other roles in plant development. *LTP1*, identified as a gene encoding calmodulin-binding protein [75], was mapped on the *QRn5 *locus. Enrichment of *LTP1 *transcripts in RT reveals its signal transduction role in rhizome development. These genes may be candidates for further function identification.

Comparative analysis indicated that 12 rhizome-specific expressed genes on the rhizome-related QTL intervals of *O. longistaminata *were aligned with similar genes in the sorghum genome, suggesting that functional conserved candidate genes across taxa could account for rhizome growth and development. With the accomplishment of sorghum genome sequencing [76], further comparative genomics study is necessary for dissecting the molecular role of these rhizome-related QTL-associated candidate genes.

## Conclusion

A whole rice genome oligonucleotide microarray was used to profile gene expression across five tissues of the perennial wild rice *O. longistaminata*. Results showed that a very complex gene regulatory network underlies rhizome development and growth, and there might be an overlapping regulatory mechanism in the establishment of rhizomes and tillers. Phytohormones such as IAA and GA are involved in the signaling pathway in determining rhizomes. Several *cis*-elements enriched in rhizome and the identified rhizome-specific genes co-localized on the rhizome-related QTL intervals provide a base for further dissection of the molecular regulatory mechanism of the rhizomatous trait in rice.

## Methods

### Plant materials and RNA sampling

The material used in this study was an unnamed wild rice accession of *O. longistaminata *originally collected from Niger [10]. It has long and strong rhizomes and has been maintained as a single plant in the greenhouse of the Food Crops Research Institute, Yunnan Academy of Agricultural Sciences, China, since it was provided by Dr. Hyakutake, the Institute of Physical and Chemical Research, Japan in 1999.

At the active tillering stage, five tissues of the *O. longistaminata *plant, including the rhizome tips (distal 1 cm of the young rhizomes), rhizome internodes, shoot tips (distal 5 mm of the tiller after removing all leaves), shoot internodes and young leaves were collected for total RNA extraction. Three independent biological replicates for each type of tissues were sampled, and all collected samples were snap-frozen in liquid nitrogen and kept in a -70°C freezer. Total RNA was extracted using TRIzol reagents according to the manufacturer's instructions, and then purified and concentrated using RNeasy MinElute Cleanup kit (Qiagen).

### Microarray hybridization and data analyses

All microarray experiments were performed using the Affymetrix GeneChip Rice Genome Array (Santa Clara, CA). The array contains 51,279 probe sets representing 48,564 *japonica *and 1,260 *indica *transcripts. Preparation of cDNA, cRNA, hybridization to the array and quality control checks were carried out by a specialized biotech company, CapitalBio Corporation, Beijing, China. Briefly, the biotin-labeled fragmented cRNA was hybridized to the array for 16 hours using GeneChip Hybridization Oven 640 (Affymetrix) according to the manufacturer's protocol, and then GeneChips were washed using Fluidics Station 450 and scanned using Gene Chip Scanner 3000. The Affymetrix GCOS software (version 1.4) was used to determine the total number of informative probe sets. The scanned images were firstly examined by visual inspection, and then processed to generate raw data saved as CEL files using the default setting of GCOS1.4. The normalization of all arrays was performed in a global scaling procedure by the dChip software. In the comparison analyses, a two class unpaired method in the Significant Analysis of Microarray software (SAM) was applied to identifying significantly differentially expressed genes between tissues. The whole set of original microarray data has been deposited in NCBI's Gene Expression Omnibus and can be freely accessed through GEO Series number GSE24228.

Tissue-enriched genes were identified by the following procedures: The microarray data were subjected to preliminary screening with a selection threshold of false discovery rate (FDR) less than 5% using a multiclass method in the SAM software. The resultant data then were further screened when the expression value of a tissue showed more than 1.5-fold change compared with other tissues using a significance level at 0.05 (*P *< 0.05) in Wilcoxon Rank-Sum tests. Differentially expressed genes between RT and ST were determined using the two-class unpaired method in the SAM software with more than two-fold change and a *q *value less than 0.05.

### Functional classification and prediction of cis-acting regulatory elements for the tissue-specific genes

The putative function of each tissue-specific gene corresponding to the probe set on the chip was predicted by the Affymetrix annotation combined with the TIGR definition and NCBI database. The 1 kb sequences upstream of the differentially expressed genes were downloaded from the TIGR rice genome database and used for predicting the *cis*-acting regulatory elements. The *cis*-element data was obtained from PLACE http://www.dna.go.jp/PLACE. The regulatory software developed by CapitalBio Corporation (Beijing) was used to perform the analysis of the enriched *cis*-regulatory elements for five different tissue-enriched genes sets and differentially regulated genes in RT. The confidence limit for a binomial proportion (*P *= 95%) was used to evaluate differences between identified *cis*-acting regulatory elements of the tissues.

### Physical mapping and alignment of the rhizome-specific expressed genes with genetically mapped rhizome-related QTLs

Physical mapping of the rhizome-specific expressed genes was performed by aligning each of the rhizome-related QTLs previously identified in the RD23-*O. Longistaminata *F_2 _population with the physical locations of the rhizome-specific expressed genes obtained from TIGR *japonica *rice assembly based on the chromosomal locations of the SSR markers flanking the rhizome-related QTLs [11].

### Semi-quantitative RT-PCR for confirmation of tissue-specific gene expression

A set of tissue-specific expressed genes identified from the microarray analysis were selected for confirmation using semi-quantitative RT-PCR. The gene sequences of the selected genes were obtained from NCBI database and the exon sequences from each gene were used for designing the primers with Primer 3 software http://frodo.wi.mit.edu/. The resulting primers sequences are listed in Additional file 10. RT-PCR was performed using the same RNA samples used for the microarray experiments. Again, three biological replicates for each sample were used. The first-strand cDNA was obtained from 1 μg of total RNA in a 50 μl reaction mixture, and 1 μl of synthesized cDNA was used as template for the PCR reaction (94°C for 2 min; then 26 cycles of 30 s at 94°C, 30 s at 52°C, 30 s at 72°C; followed at 72°C for 2 min). The reaction products were sized on 1.5% agarose gels stained with ethidium bromide.

## Authors' contributions

BF designed the experiments and drafted the manuscript. FH, DW, XZ, QL and LL performed the sample collection and microarray experiment. DW, HS, FZ and FH performed the data analyses of microarray data. TZ, LZ and DT did the RT-PCR analysis. ZL revised the final version of the manuscript. All authors have read and approved the final manuscript.

## Supplementary Material

Additional file 1 **Commonly and uniquely expressed genes in the five tissues of *O. longistaminata *detected by the Affymetrix oligomer chips**. Word file for the list of genes commonly and uniquely expressed in different tissues of *O. longistaminata* Click here for file

Additional file 2 **A complete list of 2567 differentially expressed genes in five tissues of *O. logistaminata***. Word file for the list of genes differentially expressed in five tissues of *Oryza longistaminata* Click here for file

Additional file 3 **The list of 61 genes specifically enriched in the rhizome internodes (RI) of *O. longistaminata *and their annotated functions detected by the Affymetrix GeneChip Rice Genome Array**. Word file for the list of genes enriched in the rhizome internode of *Oryza longistaminata *and their function annotation. Click here for file

Additional file 4 **The list of 299 genes specifically-enriched in the shoot tips (ST) of *O. longistaminata *and their annotated functions (TIGR) detected the Affymetrix GeneChip Rice Genome Array**. Word file for the list of genes enriched in the shoot tip of *Oryza longistaminata *and their function annotation. Click here for file

Additional file 5 **The list of 29 genes specifically enriched in the shoot internodes (SI) of *O. longistaminata *and their annotated functions detected by the Affymetrix GeneChip Rice Genome Array**. Word file for the list of genes enriched in the shoot internode of *Oryza longistaminata *and their function annotation. Click here for file

Additional file 6 **The list of 1974 genes specifically enriched in the young leaves (YL) of *O. longistaminata *and their annotated functions detected by the Affymetrix GeneChip Rice Genome Array**. Word file for the list of genes enriched in the young leaf of *Oryza longistaminata *and their function annotation. Click here for file

Additional file 7 **The RT-PCR profiles of 21 selected tissue-specifically expressed genes**. PPT file type, the RT-PCR profiles of the organ specific expressed genes Click here for file

Additional file 8 **The list of 424 genes up- and down-regulated in the rhizome tips (RT) relative to shoot tips (ST) of *O. longistaminata *and their annotated functions detected by the Affymetrix GeneChip Rice Genome Array**. Word file for the list of up- and down-regulated genes in rhizome tip compared with shoot tip in *Oryza longistaminata* Click here for file

Additional file 9 **Rhizome-specific genes located in the genomic regions of QTLs for rhizome-related traits identified in both rice and sorghum**. Word file for the list of rhizome-specific expressed genes associated with the previously mapped QTLs related to rhizome abundance and length. Click here for file

Additional file 10 **Primer list for the RT-PCR analysis used for identification of gene expression pattern detected by microarray analysis**. Word file for the list of PCR primers used for identification of gene expression pattern by semi-quantitative reverse transcription. Click here for file
