# Supplementary material for: Identification of rhizome-specific genes by genome-wide differential expression Analysis in Oryza longistaminata
Source: BMC Plant Biol. 2011 Jan 24;11:18. doi: 10.1186/1471-2229-11-18 (PMC3036607; doi:10.1186/1471-2229-11-18)
Supplement: Additional file 1 — Commonly and uniquely expressed genes in the five tissues of O. longistaminata detected by the Affymetrix oligomer chips. Word file for the list of genes commonly and uniquely expressed in different tissues of O. longistaminata [file 1471-2229-11-18-S1.DOC]

**Additional file 1.** Commonly and uniquely expressed genes in the five tissues of *O. longistaminata* detected by the Affymetrix oligomer chips

| **Tissue** | **N_1_**^a^ | **N_2_**^a^ | **vs RI** | **vs ST** | **vs SI** | **vs YL** |
| --- | --- | --- | --- | --- | --- | --- |
| Rhizome tips (RT) | 16,981 | 58 | 5,461^c^ | 447 | 5,405 | 9,149 |
| Rhizome internodes (RI) | 15,662 | 61 | - | 6,436 | 313 | 3,461 |
| Shoot tips (ST) | 16,026 | 299 | - | - | 5,636 | 8,988 |
| Shoot internodes (SI) | 15,732 | 29 | - | - | - | 3,168 |
| Young leaves (YL) | 15,294 | 1974 | - | - | - | - |
| Total | 21,372^b^ | 2566 | - | - | - | - |

^a^ N_1_ and N_2_ are the numbers of the total expressed genes and tissue-specifically expressed genes, respectively.

^b^ Total number of expressed genes in at least one tissue.

^c^ Total number of differentially expressed genes between two tissues.
