# Supplementary material for: Identification of rhizome-specific genes by genome-wide differential expression Analysis in Oryza longistaminata
Source: BMC Plant Biol. 2011 Jan 24;11:18. doi: 10.1186/1471-2229-11-18 (PMC3036607; doi:10.1186/1471-2229-11-18)
Supplement: Additional file 2 — A complete list of 2567 differentially expressed genes in five tissues of O. logistaminata. Word file for the list of genes differentially expressed in five tissues of Oryza longistaminata [file 1471-2229-11-18-S2.DOC]

**Additional file 2.** A complete list of 2567 differentially expressed genes in five tissues of *O. logistaminata*

| Gene name | q-value(%) | Ratio_1/2^a^ | Ratio_2/3^b^ | *p* values^c^ |
| --- | --- | --- | --- | --- |
| Os.20078.1.S1_at | 0.00 | 93.28 | 3.27 | 0.017 |
| Os.28424.1.A1_at | 0.00 | 75.83 | 1.33 | 0.016 |
| Os.34273.1.S1_at | 0.00 | 72.03 | 1.13 | 0.016 |
| Os.27592.1.A1_at | 0.00 | 70.78 | 1.19 | 0.040 |
| Os.47712.1.S1_at | 0.00 | 65.56 | 2.42 | 0.006 |
| Os.49185.1.S1_at | 0.00 | 57.59 | 2.34 | 0.021 |
| Os.11800.1.S1_at | 0.00 | 56.00 | 1.62 | 0.046 |
| Os.38152.1.S1_x_at | 0.00 | 52.29 | 1.16 | 0.015 |
| Os.10311.2.A1_x_at | 0.00 | 48.51 | 5.89 | 0.003 |
| Os.24876.1.S1_s_at | 0.00 | 47.68 | 1.95 | 0.011 |
| Os.11830.1.S1_at | 0.00 | 47.34 | 2.53 | 0.025 |
| Os.10311.1.S1_a_at | 0.00 | 45.17 | 4.92 | 0.002 |
| Os.14256.1.S1_a_at | 0.00 | 44.58 | 1.16 | 0.034 |
| Os.10150.1.S1_at | 0.00 | 43.73 | 1.08 | 0.021 |
| Os.15062.1.S1_a_at | 0.00 | 43.54 | 1.12 | 0.019 |
| OsAffx.1916.1.S1_s_at | 0.00 | 43.07 | 1.31 | 0.038 |
| Os.19896.2.S1_x_at | 0.00 | 42.08 | 1.13 | 0.041 |
| Os.10311.2.A1_s_at | 0.00 | 38.53 | 5.00 | 0.004 |
| Os.5438.1.S1_at | 0.00 | 37.96 | 1.49 | 0.027 |
| Os.37718.1.S1_at | 0.00 | 36.00 | 2.38 | 0.020 |
| OsAffx.19559.1.S1_at | 0.00 | 35.84 | 2.18 | 0.015 |
| Os.38760.1.S1_s_at | 0.00 | 35.14 | 3.43 | 0.019 |
| Os.1977.3.S1_x_at | 0.00 | 33.15 | 1.10 | 0.041 |
| Os.27709.1.S1_a_at | 0.00 | 32.77 | 2.04 | 0.019 |
| Os.49277.1.S1_at | 0.00 | 28.92 | 1.65 | 0.013 |
| Os.25496.1.S1_at | 0.00 | 28.10 | 1.05 | 0.021 |
| Os.3454.1.S1_at | 0.00 | 27.28 | 1.48 | 0.014 |
| Os.10597.1.S1_at | 0.00 | 25.75 | 1.09 | 0.047 |
| Os.46542.1.S1_at | 0.00 | 25.12 | 1.47 | 0.008 |
| Os.12089.1.S1_at | 0.00 | 24.83 | 1.14 | 0.024 |
| Os.32721.1.S1_at | 0.00 | 24.45 | 2.08 | 0.014 |
| Os.872.2.S1_at | 0.00 | 24.13 | 1.10 | 0.027 |
| Os.35224.1.A1_x_at | 0.00 | 23.06 | 1.31 | 0.000 |
| Os.3408.1.A2_a_at | 0.00 | 22.60 | 6.74 | 0.013 |
| Os.10146.1.S1_at | 0.00 | 21.88 | 5.37 | 0.002 |
| Os.19044.1.S1_at | 0.00 | 21.80 | 1.53 | 0.019 |
| Os.9332.1.S1_at | 0.00 | 21.38 | 1.24 | 0.009 |
| Os.13717.1.S1_at | 0.00 | 21.35 | 2.54 | 0.033 |
| Os.12037.1.S1_at | 0.00 | 21.29 | 7.52 | 0.029 |
| Os.38421.1.S1_at | 0.00 | 20.76 | 2.12 | 0.036 |
| Os.49996.1.S1_at | 0.00 | 20.75 | 1.86 | 0.020 |
| Os.3408.1.A1_x_at | 0.00 | 20.39 | 3.10 | 0.049 |
| OsAffx.19451.3.S1_x_at | 0.00 | 20.23 | 1.10 | 0.017 |
| Os.12112.1.S1_at | 0.00 | 20.14 | 4.24 | 0.011 |
| OsAffx.16825.1.S1_s_at | 0.00 | 19.99 | 1.49 | 0.000 |
| Os.14145.1.A1_at | 0.00 | 19.89 | 1.62 | 0.001 |
| Os.11674.1.S1_at | 0.00 | 19.60 | 1.84 | 0.000 |
| OsAffx.26509.1.S1_s_at | 0.00 | 19.54 | 1.18 | 0.044 |
| Os.11150.1.S1_at | 0.00 | 19.46 | 3.99 | 0.001 |
| Os.7527.1.S1_at | 0.00 | 19.39 | 1.04 | 0.000 |
| Os.54425.1.S1_at | 0.00 | 19.26 | 1.78 | 0.026 |
| OsAffx.17389.1.S1_s_at | 0.00 | 18.97 | 4.28 | 0.021 |
| OsAffx.28133.1.S1_at | 0.00 | 18.84 | 1.56 | 0.046 |
| Os.17111.2.S1_x_at | 0.00 | 18.53 | 1.06 | 0.025 |
| Os.23296.1.S1_x_at | 0.00 | 17.99 | 1.35 | 0.003 |
| Os.53427.1.S1_at | 0.00 | 17.65 | 1.45 | 0.018 |
| Os.10743.1.S1_at | 0.00 | 17.64 | 1.13 | 0.038 |
| Os.22651.1.S1_at | 0.00 | 17.57 | 2.06 | 0.042 |
| Os.51504.1.A1_at | 0.00 | 17.54 | 1.10 | 0.005 |
| Os.46638.1.S1_at | 0.00 | 17.44 | 1.04 | 0.005 |
| OsAffx.19451.3.S1_s_at | 0.00 | 17.37 | 1.02 | 0.030 |
| Os.35465.1.S1_at | 0.00 | 17.24 | 1.16 | 0.023 |
| Os.1193.1.S1_at | 0.00 | 16.76 | 1.97 | 0.012 |
| Os.8112.1.S1_at | 0.00 | 16.65 | 1.43 | 0.026 |
| Os.28404.1.A1_at | 0.00 | 16.51 | 1.09 | 0.026 |
| Os.46591.1.S1_at | 0.00 | 16.29 | 1.26 | 0.003 |
| Os.39243.1.A1_x_at | 0.00 | 15.83 | 1.38 | 0.005 |
| Os.38760.1.S1_at | 0.00 | 15.81 | 2.67 | 0.009 |
| Os.27346.1.S1_at | 0.00 | 15.55 | 1.00 | 0.001 |
| Os.39038.1.A1_at | 0.00 | 15.50 | 1.64 | 0.041 |
| Os.8012.1.S1_at | 0.00 | 15.37 | 6.05 | 0.033 |
| Os.5049.1.S1_at | 0.00 | 15.34 | 2.22 | 0.029 |
| OsAffx.4110.1.S1_at | 0.00 | 15.20 | 1.02 | 0.003 |
| Os.12371.1.S1_at | 0.00 | 14.89 | 3.39 | 0.008 |
| Os.11962.1.S1_at | 0.00 | 14.73 | 1.24 | 0.002 |
| Os.11602.1.S1_at | 0.00 | 14.41 | 15.44 | 0.002 |
| Os.15243.1.S1_a_at | 0.00 | 14.39 | 1.07 | 0.031 |
| Os.11807.1.S1_at | 0.00 | 14.23 | 1.32 | 0.000 |
| Os.9836.1.S1_at | 0.00 | 14.12 | 1.11 | 0.022 |
| Os.30998.1.S1_at | 0.00 | 13.75 | 1.12 | 0.009 |
| Os.36648.1.S1_x_at | 0.00 | 13.52 | 3.24 | 0.043 |
| Os.7695.1.S1_at | 0.00 | 13.32 | 1.11 | 0.031 |
| Os.30311.1.S1_at | 0.00 | 13.26 | 1.41 | 0.014 |
| Os.38848.1.S1_at | 0.00 | 13.18 | 1.67 | 0.000 |
| Os.54675.1.S1_x_at | 0.00 | 13.04 | 1.04 | 0.041 |
| Os.18856.1.S1_at | 0.00 | 12.88 | 1.20 | 0.025 |
| Os.11753.1.S1_at | 0.00 | 12.73 | 1.23 | 0.002 |
| OsAffx.13891.1.S1_s_at | 0.00 | 12.68 | 1.30 | 0.033 |
| Os.435.1.S1_at | 0.00 | 12.68 | 1.74 | 0.001 |
| Os.20203.1.S1_s_at | 0.00 | 12.63 | 2.45 | 0.023 |
| Os.17158.1.S1_at | 0.00 | 12.48 | 1.05 | 0.025 |
| Os.10113.1.S1_at | 0.00 | 12.44 | 3.91 | 0.005 |
| Os.18913.1.S1_x_at | 0.00 | 12.34 | 1.91 | 0.000 |
| Os.57062.1.S1_at | 0.00 | 12.25 | 1.05 | 0.013 |
| Os.11654.1.S1_at | 0.00 | 12.18 | 1.20 | 0.000 |
| Os.15641.1.S1_at | 0.00 | 12.08 | 2.35 | 0.041 |
| Os.46591.1.S1_x_at | 0.00 | 12.05 | 1.37 | 0.004 |
| Os.54675.1.S1_at | 0.00 | 12.02 | 1.05 | 0.038 |
| Os.12394.1.S1_at | 0.00 | 12.02 | 2.46 | 0.006 |
| OsAffx.14410.1.S1_s_at | 0.00 | 12.01 | 3.38 | 0.044 |
| Os.37955.2.S1_at | 0.00 | 12.00 | 1.06 | 0.046 |
| Os.23145.1.S1_at | 0.00 | 11.95 | 1.75 | 0.028 |
| Os.23056.2.S1_x_at | 0.00 | 11.82 | 4.84 | 0.005 |
| Os.9996.1.S1_at | 0.00 | 11.77 | 3.50 | 0.015 |
| Os.7868.1.S1_at | 0.00 | 11.72 | 1.62 | 0.017 |
| Os.11566.1.S1_at | 0.00 | 11.70 | 2.53 | 0.000 |
| Os.52278.1.S1_s_at | 0.00 | 11.67 | 6.07 | 0.005 |
| Os.49230.1.S1_at | 0.00 | 11.52 | 1.09 | 0.000 |
| Os.27513.1.A1_a_at | 0.00 | 11.48 | 1.92 | 0.001 |
| Os.5188.2.S1_s_at | 0.00 | 11.48 | 1.19 | 0.036 |
| Os.8468.1.S1_s_at | 0.00 | 11.35 | 1.01 | 0.000 |
| Os.20521.1.S1_at | 0.00 | 11.33 | 1.27 | 0.011 |
| Os.27835.1.S1_at | 0.00 | 11.32 | 1.78 | 0.000 |
| Os.4324.1.S1_at | 0.00 | 11.15 | 2.21 | 0.009 |
| Os.7907.1.S2_a_at | 0.00 | 11.02 | 1.28 | 0.011 |
| OsAffx.26321.1.S1_at | 0.00 | 10.98 | 1.32 | 0.017 |
| Os.7443.1.S1_s_at | 0.00 | 10.96 | 1.26 | 0.009 |
| OsAffx.20051.1.S1_at | 0.00 | 10.88 | 2.05 | 0.019 |
| OsAffx.32230.1.A1_x_at | 0.00 | 10.78 | 2.08 | 0.014 |
| Os.8888.1.S1_at | 0.00 | 10.77 | 1.92 | 0.002 |
| Os.7907.2.S1_x_at | 0.00 | 10.69 | 1.72 | 0.024 |
| OsAffx.12262.1.S1_at | 0.00 | 10.68 | 1.10 | 0.001 |
| Os.57530.1.S1_x_at | 0.00 | 10.51 | 1.03 | 0.047 |
| Os.10975.1.S1_at | 0.00 | 10.44 | 1.22 | 0.000 |
| Os.15243.2.S1_x_at | 0.00 | 10.42 | 7.53 | 0.008 |
| Os.24906.1.S1_at | 0.00 | 10.41 | 1.35 | 0.017 |
| Os.10031.1.S1_at | 0.00 | 10.39 | 4.31 | 0.002 |
| OsAffx.23932.1.S1_at | 0.00 | 10.37 | 3.65 | 0.020 |
| Os.26807.1.S1_at | 0.00 | 10.32 | 1.29 | 0.000 |
| Os.23745.1.S1_at | 0.00 | 10.27 | 1.10 | 0.010 |
| Os.8682.1.S1_a_at | 0.00 | 10.12 | 1.27 | 0.047 |
| Os.12594.1.S1_x_at | 0.00 | 10.09 | 3.43 | 0.007 |
| Os.21088.1.S1_at | 0.00 | 9.98 | 4.13 | 0.023 |
| Os.1411.1.S1_at | 0.00 | 9.96 | 5.19 | 0.034 |
| Os.19361.1.S2_at | 0.00 | 9.96 | 1.02 | 0.048 |
| Os.10200.1.S1_at | 0.00 | 9.96 | 1.16 | 0.001 |
| Os.26552.1.S1_at | 0.00 | 9.91 | 1.00 | 0.031 |
| Os.27730.1.S1_at | 0.00 | 9.88 | 1.11 | 0.004 |
| Os.8785.1.S1_at | 0.00 | 9.81 | 1.20 | 0.016 |
| Os.12771.1.S1_at | 0.00 | 9.76 | 1.19 | 0.021 |
| Os.25587.1.S1_at | 0.00 | 9.76 | 1.15 | 0.017 |
| Os.3496.1.S1_at | 0.00 | 9.56 | 2.68 | 0.016 |
| Os.21178.1.S1_at | 0.00 | 9.56 | 1.71 | 0.044 |
| Os.46071.1.S1_x_at | 0.00 | 9.53 | 6.96 | 0.023 |
| Os.19141.1.S1_at | 0.00 | 9.50 | 2.81 | 0.000 |
| OsAffx.13521.1.S1_at | 0.00 | 9.49 | 1.53 | 0.003 |
| Os.17768.1.S1_at | 0.01 | 9.49 | 1.73 | 0.004 |
| Os.3399.1.S1_at | 0.00 | 9.49 | 1.67 | 0.048 |
| OsAffx.6098.1.S1_at | 0.00 | 9.44 | 1.12 | 0.028 |
| Os.7662.1.S1_at | 0.00 | 9.43 | 8.39 | 0.006 |
| Os.7907.1.S2_at | 0.00 | 9.38 | 1.28 | 0.002 |
| Os.7137.1.S1_at | 0.00 | 9.34 | 2.30 | 0.044 |
| Os.35778.1.S1_at | 0.00 | 9.25 | 1.08 | 0.022 |
| Os.26798.1.S1_at | 0.00 | 9.23 | 1.61 | 0.007 |
| Os.56167.1.S1_at | 0.00 | 9.21 | 1.07 | 0.029 |
| OsAffx.12520.1.S1_s_at | 0.00 | 9.20 | 1.04 | 0.011 |
| Os.27334.1.S1_at | 0.00 | 9.13 | 1.93 | 0.019 |
| Os.46071.1.S1_at | 0.00 | 9.12 | 8.71 | 0.020 |
| Os.6448.1.S1_at | 0.00 | 9.11 | 4.61 | 0.001 |
| Os.46634.1.S1_x_at | 0.00 | 9.09 | 1.03 | 0.017 |
| OsAffx.30475.5.S1_x_at | 0.00 | 9.08 | 3.51 | 0.049 |
| Os.52161.1.S1_x_at | 0.00 | 9.07 | 2.36 | 0.008 |
| Os.28433.4.A1_at | 0.00 | 9.03 | 1.13 | 0.038 |
| Os.52224.1.S1_at | 0.00 | 9.03 | 1.00 | 0.000 |
| Os.22842.1.S1_at | 0.00 | 9.00 | 1.08 | 0.040 |
| Os.49149.1.S1_at | 0.00 | 8.95 | 1.05 | 0.005 |
| OsAffx.32207.1.A1_at | 0.00 | 8.94 | 2.17 | 0.002 |
| Os.18786.1.S1_at | 0.00 | 8.94 | 1.12 | 0.010 |
| Os.8120.1.S1_at | 0.00 | 8.88 | 1.04 | 0.000 |
| Os.7346.1.S1_at | 0.00 | 8.88 | 1.09 | 0.003 |
| Os.12025.1.S1_a_at | 0.00 | 8.82 | 1.39 | 0.036 |
| Os.50919.1.S1_at | 0.00 | 8.81 | 2.13 | 0.031 |
| Os.28433.5.S1_x_at | 0.00 | 8.81 | 1.40 | 0.024 |
| Os.18958.1.S1_at | 0.00 | 8.80 | 1.22 | 0.035 |
| Os.49107.1.A1_at | 0.00 | 8.79 | 1.45 | 0.015 |
| Os.10853.1.S1_at | 0.00 | 8.74 | 5.96 | 0.000 |
| OsAffx.14459.1.S1_at | 0.00 | 8.74 | 1.09 | 0.025 |
| Os.32279.1.S1_at | 0.00 | 8.74 | 1.05 | 0.002 |
| Os.11981.1.S1_x_at | 0.01 | 8.72 | 1.41 | 0.039 |
| Os.52657.1.S1_at | 0.00 | 8.71 | 1.41 | 0.020 |
| Os.19627.1.S1_a_at | 0.00 | 8.70 | 1.39 | 0.026 |
| Os.51130.1.S1_at | 0.00 | 8.64 | 2.77 | 0.015 |
| Os.57337.1.S1_at | 0.00 | 8.59 | 1.33 | 0.016 |
| Os.1229.2.S1_at | 0.00 | 8.52 | 1.01 | 0.026 |
| Os.14369.2.S1_at | 0.00 | 8.51 | 1.66 | 0.041 |
| Os.19100.1.S1_at | 0.00 | 8.51 | 1.88 | 0.046 |
| Os.14283.1.A1_at | 0.00 | 8.44 | 1.23 | 0.019 |
| Os.20203.1.S1_at | 0.00 | 8.44 | 1.49 | 0.038 |
| Os.16966.1.S1_s_at | 0.00 | 8.44 | 2.44 | 0.015 |
| Os.12126.2.S1_at | 0.00 | 8.43 | 2.09 | 0.001 |
| Os.27610.1.S1_at | 0.00 | 8.42 | 1.35 | 0.002 |
| OsAffx.13521.1.S1_x_at | 0.00 | 8.39 | 1.70 | 0.003 |
| Os.6340.1.S1_at | 0.00 | 8.35 | 1.57 | 0.023 |
| OsAffx.30475.1.S1_s_at | 0.00 | 8.35 | 3.27 | 0.013 |
| Os.22474.1.S1_at | 0.00 | 8.34 | 1.59 | 0.000 |
| Os.8682.2.S1_x_at | 0.00 | 8.31 | 1.55 | 0.037 |
| OsAffx.6713.1.S1_at | 0.00 | 8.30 | 2.76 | 0.042 |
| Os.53046.1.S1_s_at | 0.00 | 8.28 | 1.58 | 0.001 |
| Os.23808.1.S1_x_at | 0.00 | 8.27 | 2.79 | 0.004 |
| Os.27825.1.S1_at | 0.00 | 8.22 | 1.43 | 0.000 |
| Os.26818.1.S1_a_at | 0.00 | 8.21 | 1.41 | 0.001 |
| Os.20934.1.A1_at | 0.00 | 8.21 | 1.11 | 0.049 |
| Os.12100.1.S1_at | 0.00 | 8.20 | 3.95 | 0.004 |
| Os.24901.1.A1_at | 0.00 | 8.18 | 1.93 | 0.023 |
| Os.52283.1.S1_at | 0.00 | 8.16 | 2.47 | 0.002 |
| Os.11397.1.S1_at | 0.00 | 8.15 | 3.91 | 0.001 |
| Os.51052.1.S1_at | 0.00 | 8.15 | 1.11 | 0.024 |
| Os.45018.1.S1_x_at | 0.00 | 8.13 | 1.10 | 0.013 |
| Os.27634.1.S1_at | 0.00 | 8.09 | 2.59 | 0.005 |
| Os.37494.1.S1_x_at | 0.00 | 8.08 | 1.55 | 0.029 |
| Os.12102.1.S1_at | 0.00 | 8.07 | 1.30 | 0.000 |
| Os.17501.1.A1_at | 0.00 | 8.04 | 2.08 | 0.015 |
| Os.27627.1.S1_at | 0.02 | 8.02 | 1.30 | 0.046 |
| Os.27830.1.S1_at | 0.00 | 8.02 | 1.92 | 0.003 |
| Os.9828.1.S1_at | 0.00 | 7.97 | 1.26 | 0.023 |
| Os.11670.1.S1_at | 0.00 | 7.97 | 1.29 | 0.020 |
| Os.9756.1.S1_a_at | 0.00 | 7.94 | 3.97 | 0.001 |
| Os.11858.1.S1_at | 0.01 | 7.93 | 1.38 | 0.004 |
| Os.15618.1.S1_a_at | 0.00 | 7.93 | 1.07 | 0.010 |
| Os.10360.1.S1_at | 0.00 | 7.92 | 2.87 | 0.003 |
| Os.12699.1.S1_at | 0.00 | 7.92 | 2.01 | 0.000 |
| OsAffx.3615.1.S1_s_at | 0.00 | 7.91 | 1.15 | 0.048 |
| Os.172.1.S1_a_at | 0.03 | 7.88 | 1.63 | 0.038 |
| Os.11854.1.S1_at | 0.00 | 7.82 | 2.39 | 0.007 |
| Os.9792.1.S1_at | 0.00 | 7.80 | 1.79 | 0.008 |
| OsAffx.7294.1.S1_at | 0.00 | 7.79 | 1.67 | 0.039 |
| Os.18384.1.S1_at | 0.00 | 7.74 | 2.46 | 0.014 |
| OsAffx.28001.1.S1_s_at | 0.00 | 7.73 | 1.87 | 0.045 |
| Os.12967.2.A1_at | 0.00 | 7.73 | 1.00 | 0.031 |
| Os.35302.1.S1_at | 0.00 | 7.71 | 2.20 | 0.025 |
| Os.6172.1.S1_s_at | 0.00 | 7.69 | 1.06 | 0.028 |
| OsAffx.24550.1.S1_at | 0.00 | 7.68 | 1.62 | 0.001 |
| Os.17566.1.S1_at | 0.00 | 7.63 | 1.01 | 0.021 |
| Os.21073.1.S1_at | 0.00 | 7.63 | 1.54 | 0.023 |
| Os.24032.1.S1_at | 0.00 | 7.59 | 1.50 | 0.002 |
| Os.10292.1.S1_at | 0.00 | 7.59 | 1.85 | 0.011 |
| Os.51003.1.S1_at | 0.00 | 7.55 | 1.10 | 0.011 |
| Os.10454.1.S1_a_at | 0.00 | 7.53 | 1.04 | 0.000 |
| Os.27770.3.A1_a_at | 0.00 | 7.52 | 2.05 | 0.007 |
| OsAffx.6713.2.S1_at | 0.00 | 7.51 | 1.10 | 0.027 |
| Os.28305.1.S1_at | 0.00 | 7.51 | 1.57 | 0.033 |
| Os.25806.1.S1_at | 0.00 | 7.48 | 1.22 | 0.001 |
| OsAffx.32330.1.S1_x_at | 0.00 | 7.43 | 2.58 | 0.001 |
| Os.12608.1.S1_at | 0.00 | 7.41 | 2.08 | 0.000 |
| Os.16966.1.S1_at | 0.00 | 7.41 | 1.57 | 0.033 |
| Os.11387.1.S1_a_at | 0.00 | 7.40 | 1.39 | 0.011 |
| Os.3818.1.S1_a_at | 0.00 | 7.39 | 1.36 | 0.001 |
| Os.23808.3.S1_x_at | 0.00 | 7.38 | 2.42 | 0.007 |
| Os.19863.1.S1_a_at | 0.00 | 7.37 | 1.19 | 0.000 |
| Os.25613.1.A1_s_at | 0.00 | 7.35 | 2.34 | 0.050 |
| Os.10689.1.S1_at | 0.00 | 7.34 | 1.14 | 0.011 |
| Os.8521.2.S1_x_at | 0.00 | 7.28 | 1.20 | 0.032 |
| OsAffx.30475.9.S1_s_at | 0.00 | 7.23 | 3.24 | 0.001 |
| Os.23290.2.S1_x_at | 0.00 | 7.21 | 1.57 | 0.039 |
| OsAffx.4662.1.S1_at | 0.00 | 7.21 | 5.02 | 0.000 |
| Os.35688.1.S1_at | 0.00 | 7.21 | 1.29 | 0.024 |
| OsAffx.32241.1.S1_x_at | 0.00 | 7.20 | 1.43 | 0.017 |
| Os.55374.1.S1_s_at | 0.00 | 7.19 | 2.76 | 0.001 |
| Os.15142.1.S1_at | 0.00 | 7.15 | 1.62 | 0.019 |
| Os.1071.1.S1_at | 0.00 | 7.12 | 1.85 | 0.015 |
| Os.7931.1.S1_s_at | 0.00 | 7.11 | 2.51 | 0.000 |
| Os.12110.1.S1_at | 0.00 | 7.09 | 4.52 | 0.024 |
| Os.27837.2.S1_x_at | 0.00 | 7.07 | 1.46 | 0.017 |
| Os.52666.1.S1_at | 0.00 | 7.07 | 1.33 | 0.012 |
| Os.14793.1.S1_at | 0.00 | 7.05 | 1.23 | 0.020 |
| OsAffx.24657.1.S1_at | 0.00 | 7.04 | 1.11 | 0.023 |
| Os.7705.1.S1_at | 0.00 | 7.03 | 1.98 | 0.002 |
| Os.17002.1.S1_at | 0.00 | 7.01 | 1.02 | 0.001 |
| Os.50886.1.S1_at | 0.00 | 7.01 | 1.02 | 0.015 |
| Os.14877.1.S1_at | 0.00 | 6.97 | 2.80 | 0.034 |
| Os.5754.1.S1_at | 0.00 | 6.97 | 1.51 | 0.002 |
| Os.44735.1.S1_at | 0.00 | 6.96 | 1.14 | 0.003 |
| Os.26732.1.S1_at | 0.00 | 6.94 | 2.42 | 0.003 |
| Os.27754.1.S1_at | 0.00 | 6.94 | 1.13 | 0.000 |
| Os.17449.1.A1_at | 0.05 | 6.93 | 2.47 | 0.011 |
| Os.55033.1.A1_at | 0.00 | 6.92 | 3.15 | 0.012 |
| Os.52588.1.S1_at | 0.01 | 6.91 | 1.39 | 0.007 |
| OsAffx.3056.1.S1_at | 0.01 | 6.90 | 1.00 | 0.005 |
| Os.1385.2.S1_x_at | 0.00 | 6.90 | 1.37 | 0.007 |
| Os.53046.1.S1_at | 0.00 | 6.90 | 1.39 | 0.044 |
| Os.52934.1.S1_at | 0.00 | 6.90 | 1.06 | 0.024 |
| Os.15600.1.S1_a_at | 0.00 | 6.88 | 3.43 | 0.001 |
| Os.41694.1.S1_at | 0.01 | 6.87 | 1.04 | 0.020 |
| Os.27003.1.S2_a_at | 0.00 | 6.84 | 1.09 | 0.010 |
| Os.27416.1.S1_at | 0.00 | 6.82 | 1.29 | 0.041 |
| Os.11947.1.S1_a_at | 0.00 | 6.81 | 1.48 | 0.000 |
| Os.51464.1.A1_at | 0.00 | 6.78 | 1.40 | 0.005 |
| Os.2422.1.S1_a_at | 0.00 | 6.77 | 5.07 | 0.016 |
| Os.27770.1.A1_a_at | 0.00 | 6.74 | 1.48 | 0.001 |
| Os.27603.1.S1_at | 0.00 | 6.71 | 1.49 | 0.007 |
| Os.48107.1.A1_at | 0.01 | 6.69 | 1.21 | 0.000 |
| Os.38152.1.S1_at | 0.00 | 6.69 | 1.21 | 0.012 |
| Os.6379.1.S1_at | 0.00 | 6.67 | 1.01 | 0.008 |
| Os.27216.1.A1_at | 0.00 | 6.66 | 1.09 | 0.000 |
| OsAffx.3309.1.S1_s_at | 0.00 | 6.63 | 1.59 | 0.003 |
| Os.11303.1.S1_at | 0.00 | 6.62 | 1.36 | 0.050 |
| Os.22726.1.A1_at | 0.00 | 6.62 | 3.32 | 0.003 |
| Os.9913.1.S1_at | 0.00 | 6.59 | 3.40 | 0.004 |
| Os.26798.2.S1_a_at | 0.00 | 6.55 | 1.65 | 0.034 |
| Os.6375.1.S1_s_at | 0.00 | 6.54 | 1.37 | 0.029 |
| Os.50753.1.S1_at | 0.03 | 6.51 | 1.46 | 0.010 |
| Os.6998.1.S1_at | 0.00 | 6.51 | 2.81 | 0.023 |
| Os.7826.2.A1_at | 0.00 | 6.51 | 1.54 | 0.002 |
| Os.47301.1.A1_at | 0.00 | 6.50 | 4.16 | 0.003 |
| Os.51132.1.S1_at | 0.00 | 6.48 | 1.32 | 0.004 |
| Os.1899.1.S1_at | 0.00 | 6.47 | 2.10 | 0.015 |
| Os.5357.1.S1_at | 0.00 | 6.44 | 1.69 | 0.000 |
| Os.1229.2.S1_x_at | 0.00 | 6.43 | 1.05 | 0.012 |
| Os.11918.1.S1_at | 0.02 | 6.41 | 1.32 | 0.015 |
| Os.16838.1.S1_at | 0.00 | 6.41 | 1.97 | 0.026 |
| OsAffx.12824.1.S1_at | 0.01 | 6.40 | 1.06 | 0.004 |
| Os.15194.1.S1_at | 0.00 | 6.39 | 1.59 | 0.002 |
| Os.5643.1.S1_at | 0.07 | 6.38 | 1.09 | 0.005 |
| Os.25587.1.S1_s_at | 0.00 | 6.37 | 1.26 | 0.007 |
| Os.50917.1.S1_at | 0.00 | 6.36 | 1.41 | 0.000 |
| Os.17721.1.S1_at | 0.00 | 6.35 | 1.49 | 0.003 |
| Os.12313.1.S1_at | 0.00 | 6.33 | 6.35 | 0.046 |
| OsAffx.19331.1.S1_at | 0.00 | 6.31 | 5.09 | 0.019 |
| Os.39363.1.A1_x_at | 0.00 | 6.29 | 13.64 | 0.033 |
| Os.26965.1.S1_at | 0.00 | 6.28 | 1.56 | 0.040 |
| Os.38283.1.S2_a_at | 0.02 | 6.28 | 1.23 | 0.003 |
| Os.27006.1.S1_at | 0.00 | 6.27 | 1.47 | 0.043 |
| Os.5873.1.S1_at | 0.00 | 6.27 | 2.07 | 0.012 |
| OsAffx.14131.1.S1_at | 0.00 | 6.26 | 1.06 | 0.013 |
| Os.5613.1.S1_s_at | 0.00 | 6.24 | 1.56 | 0.036 |
| Os.27805.1.S1_at | 0.00 | 6.24 | 1.51 | 0.001 |
| Os.32447.1.S1_at | 0.00 | 6.23 | 1.16 | 0.001 |
| Os.11917.1.S1_at | 0.00 | 6.21 | 2.00 | 0.004 |
| OsAffx.24877.1.S1_at | 0.00 | 6.21 | 1.16 | 0.021 |
| Os.5412.1.S1_at | 0.00 | 6.19 | 4.15 | 0.014 |
| Os.23687.1.A1_at | 0.00 | 6.18 | 1.14 | 0.002 |
| Os.5010.1.S1_at | 0.00 | 6.18 | 1.02 | 0.037 |
| OsAffx.13950.2.S1_at | 0.00 | 6.18 | 1.33 | 0.000 |
| Os.3451.1.S1_at | 0.00 | 6.16 | 2.71 | 0.002 |
| Os.18211.1.S1_at | 0.02 | 6.16 | 1.12 | 0.023 |
| Os.52433.1.S1_at | 0.00 | 6.15 | 2.40 | 0.036 |
| Os.27510.1.A1_at | 0.00 | 6.15 | 1.92 | 0.005 |
| Os.26862.1.S1_s_at | 0.00 | 6.13 | 1.27 | 0.003 |
| Os.23773.1.A1_at | 0.00 | 6.11 | 1.11 | 0.001 |
| Os.18305.1.S1_at | 0.00 | 6.10 | 1.27 | 0.014 |
| Os.46397.1.S1_x_at | 0.00 | 6.07 | 1.64 | 0.015 |
| Os.20361.1.A1_at | 0.00 | 6.06 | 1.68 | 0.006 |
| Os.7712.1.S1_a_at | 0.00 | 6.04 | 1.64 | 0.003 |
| Os.11756.1.S1_s_at | 0.00 | 6.04 | 6.20 | 0.026 |
| Os.38283.1.S3_a_at | 0.01 | 6.03 | 1.26 | 0.002 |
| Os.20187.2.S1_at | 0.00 | 6.02 | 2.03 | 0.026 |
| OsAffx.9908.1.S1_x_at | 0.00 | 6.01 | 1.02 | 0.002 |
| Os.5363.1.S1_at | 0.03 | 6.00 | 1.19 | 0.049 |
| Os.52757.1.S2_at | 0.00 | 6.00 | 1.24 | 0.001 |
| OsAffx.30524.1.S1_x_at | 0.00 | 5.98 | 1.44 | 0.037 |
| OsAffx.24686.1.S1_at | 0.02 | 5.98 | 1.01 | 0.036 |
| OsAffx.23277.1.S1_at | 0.00 | 5.96 | 1.61 | 0.005 |
| Os.15071.2.S1_s_at | 0.00 | 5.95 | 2.60 | 0.050 |
| OsAffx.23156.1.S1_at | 0.00 | 5.94 | 1.21 | 0.008 |
| Os.23135.1.A1_at | 0.00 | 5.94 | 1.20 | 0.042 |
| Os.48545.1.S1_at | 0.00 | 5.93 | 1.44 | 0.004 |
| Os.7931.1.S1_a_at | 0.00 | 5.93 | 2.24 | 0.010 |
| Os.8554.2.S1_x_at | 0.00 | 5.93 | 1.14 | 0.001 |
| Os.52106.1.S1_at | 0.00 | 5.92 | 1.08 | 0.020 |
| Os.25529.1.S1_at | 0.00 | 5.92 | 1.16 | 0.000 |
| Os.286.1.S1_a_at | 0.02 | 5.91 | 1.44 | 0.026 |
| Os.31486.1.S1_at | 0.00 | 5.90 | 1.32 | 0.027 |
| Os.12977.1.S1_at | 0.00 | 5.89 | 1.05 | 0.000 |
| OsAffx.29987.1.S1_at | 0.00 | 5.88 | 1.03 | 0.011 |
| Os.17679.1.S1_at | 0.00 | 5.88 | 1.07 | 0.000 |
| Os.47706.1.S1_at | 0.00 | 5.87 | 2.36 | 0.007 |
| Os.8994.1.S1_at | 0.00 | 5.87 | 1.26 | 0.034 |
| Os.25530.1.S2_at | 0.00 | 5.86 | 1.14 | 0.007 |
| Os.3141.2.S1_at | 0.00 | 5.86 | 1.13 | 0.003 |
| Os.22767.1.S1_at | 0.00 | 5.85 | 2.01 | 0.038 |
| Os.11632.1.S1_at | 0.00 | 5.84 | 3.36 | 0.015 |
| OsAffx.21509.1.S1_at | 0.00 | 5.82 | 1.37 | 0.043 |
| Os.46404.1.S1_x_at | 0.00 | 5.81 | 6.32 | 0.003 |
| Os.50556.1.S1_at | 0.00 | 5.80 | 1.25 | 0.033 |
| OsAffx.4983.2.S1_x_at | 0.00 | 5.80 | 1.86 | 0.004 |
| Os.4608.3.A1_a_at | 0.00 | 5.80 | 1.15 | 0.005 |
| Os.2857.1.S1_at | 0.00 | 5.79 | 1.01 | 0.000 |
| Os.11756.1.S1_at | 0.00 | 5.77 | 8.11 | 0.023 |
| Os.19519.1.S1_s_at | 0.01 | 5.76 | 1.04 | 0.044 |
| Os.11824.1.S1_a_at | 0.00 | 5.75 | 1.89 | 0.004 |
| Os.5178.1.A1_s_at | 0.00 | 5.73 | 1.19 | 0.002 |
| Os.11725.1.S1_at | 0.00 | 5.73 | 1.14 | 0.007 |
| OsAffx.23830.2.S1_at | 0.00 | 5.73 | 1.56 | 0.006 |
| Os.23773.2.S1_at | 0.00 | 5.72 | 1.40 | 0.024 |
| Os.27650.1.S1_at | 0.00 | 5.69 | 2.87 | 0.004 |
| Os.16220.1.A1_at | 0.00 | 5.67 | 1.24 | 0.021 |
| Os.26991.1.S1_x_at | 0.01 | 5.64 | 1.02 | 0.024 |
| OsAffx.1782.2.S1_at | 0.00 | 5.64 | 1.19 | 0.021 |
| Os.13995.1.S1_at | 0.02 | 5.64 | 1.09 | 0.024 |
| Os.5449.1.S1_at | 0.00 | 5.62 | 1.39 | 0.001 |
| Os.27577.1.S1_at | 0.00 | 5.62 | 1.01 | 0.041 |
| Os.8399.1.S1_at | 0.00 | 5.62 | 1.69 | 0.002 |
| OsAffx.28769.2.S1_at | 0.00 | 5.60 | 1.09 | 0.028 |
| OsAffx.13950.2.S1_x_at | 0.00 | 5.59 | 1.35 | 0.000 |
| Os.20050.1.A1_at | 0.05 | 5.57 | 1.43 | 0.023 |
| Os.11300.2.S1_at | 0.00 | 5.56 | 1.94 | 0.004 |
| Os.10598.1.S1_at | 0.00 | 5.56 | 1.05 | 0.008 |
| Os.11888.4.S1_at | 0.00 | 5.55 | 1.38 | 0.014 |
| Os.9678.1.S1_at | 0.00 | 5.54 | 2.22 | 0.002 |
| Os.52346.1.S1_at | 0.01 | 5.52 | 1.28 | 0.007 |
| Os.5370.1.S1_at | 0.00 | 5.51 | 2.18 | 0.001 |
| Os.52757.1.S1_at | 0.00 | 5.50 | 1.12 | 0.000 |
| OsAffx.32230.1.A1_at | 0.00 | 5.49 | 2.65 | 0.010 |
| Os.9440.1.S1_a_at | 0.00 | 5.49 | 1.36 | 0.001 |
| Os.17502.1.S1_at | 0.01 | 5.49 | 1.08 | 0.003 |
| Os.7132.1.S1_at | 0.00 | 5.48 | 1.10 | 0.006 |
| Os.43896.1.S1_at | 0.02 | 5.48 | 1.41 | 0.003 |
| Os.773.1.S1_s_at | 0.00 | 5.47 | 1.13 | 0.044 |
| Os.8741.1.S1_at | 0.01 | 5.47 | 1.41 | 0.010 |
| Os.18305.1.S2_at | 0.00 | 5.47 | 1.18 | 0.001 |
| Os.21210.1.S1_at | 0.00 | 5.46 | 1.06 | 0.004 |
| Os.49797.1.A1_at | 0.00 | 5.46 | 1.18 | 0.001 |
| Os.8507.1.S1_at | 0.00 | 5.44 | 1.35 | 0.046 |
| Os.20638.1.S2_at | 0.00 | 5.43 | 1.18 | 0.018 |
| Os.46288.1.A1_at | 0.00 | 5.42 | 1.45 | 0.013 |
| Os.17369.1.S1_a_at | 0.00 | 5.41 | 1.53 | 0.000 |
| Os.17393.1.S1_at | 0.00 | 5.40 | 1.86 | 0.012 |
| Os.29051.1.S1_at | 0.00 | 5.39 | 1.37 | 0.002 |
| Os.4518.2.S1_x_at | 0.00 | 5.39 | 3.39 | 0.020 |
| Os.22699.1.S1_at | 0.00 | 5.35 | 1.01 | 0.026 |
| Os.27686.1.S1_at | 0.00 | 5.34 | 2.59 | 0.003 |
| Os.52507.1.S1_at | 0.00 | 5.34 | 1.08 | 0.002 |
| Os.47575.1.S1_at | 0.00 | 5.33 | 1.56 | 0.003 |
| Os.18168.1.S1_at | 0.27 | 5.33 | 1.00 | 0.004 |
| Os.7308.1.S2_x_at | 0.00 | 5.33 | 1.09 | 0.002 |
| OsAffx.2362.1.S1_at | 0.00 | 5.33 | 1.50 | 0.048 |
| Os.1505.1.S1_x_at | 0.00 | 5.32 | 1.15 | 0.030 |
| Os.55532.1.S1_at | 0.00 | 5.31 | 1.21 | 0.017 |
| OsAffx.21119.2.S1_at | 0.00 | 5.31 | 1.48 | 0.009 |
| Os.26206.1.S1_at | 0.00 | 5.31 | 1.38 | 0.004 |
| OsAffx.24076.1.S1_at | 0.00 | 5.30 | 1.09 | 0.034 |
| Os.5736.1.S1_s_at | 0.00 | 5.30 | 2.58 | 0.021 |
| Os.37204.1.S1_at | 0.00 | 5.29 | 2.85 | 0.017 |
| Os.50871.1.S1_at | 0.00 | 5.29 | 1.49 | 0.001 |
| Os.8686.1.S1_at | 0.01 | 5.29 | 1.01 | 0.001 |
| Os.46641.1.S1_x_at | 0.00 | 5.29 | 1.47 | 0.037 |
| Os.534.3.S1_x_at | 0.00 | 5.28 | 2.06 | 0.007 |
| Os.7872.1.S1_at | 0.00 | 5.28 | 12.97 | 0.008 |
| Os.20351.1.S1_at | 0.00 | 5.27 | 2.02 | 0.002 |
| Os.1149.1.S1_at | 0.00 | 5.27 | 1.07 | 0.002 |
| Os.18818.1.S1_a_at | 0.00 | 5.27 | 1.57 | 0.012 |
| Os.52647.1.S1_at | 0.01 | 5.27 | 1.03 | 0.023 |
| Os.54198.1.S1_at | 0.00 | 5.26 | 1.08 | 0.002 |
| Os.8465.1.S1_a_at | 0.00 | 5.26 | 1.23 | 0.001 |
| Os.17909.1.S1_a_at | 0.00 | 5.25 | 3.85 | 0.020 |
| OsAffx.32329.1.S1_x_at | 0.00 | 5.24 | 1.52 | 0.001 |
| Os.16309.1.S1_at | 0.00 | 5.24 | 1.25 | 0.030 |
| Os.4518.1.S1_a_at | 0.00 | 5.23 | 3.59 | 0.020 |
| Os.52535.1.S1_at | 0.00 | 5.23 | 7.71 | 0.001 |
| Os.50313.1.S1_at | 0.00 | 5.22 | 1.35 | 0.021 |
| Os.25561.1.S1_at | 0.00 | 5.22 | 2.54 | 0.001 |
| Os.27587.1.S1_at | 0.00 | 5.22 | 5.09 | 0.006 |
| Os.9105.1.S1_at | 0.00 | 5.22 | 1.15 | 0.042 |
| Os.12805.1.S1_at | 0.00 | 5.21 | 1.39 | 0.001 |
| Os.27417.1.S1_at | 0.00 | 5.21 | 2.03 | 0.006 |
| Os.54496.1.S1_x_at | 0.00 | 5.20 | 3.65 | 0.017 |
| Os.5335.1.S1_at | 0.02 | 5.20 | 2.70 | 0.031 |
| Os.6898.1.S1_at | 0.00 | 5.18 | 10.54 | 0.025 |
| Os.25510.1.S1_at | 0.00 | 5.18 | 1.07 | 0.036 |
| Os.49951.1.S1_at | 0.00 | 5.18 | 1.16 | 0.003 |
| Os.5442.1.S1_at | 0.00 | 5.18 | 1.29 | 0.002 |
| Os.8133.1.S1_at | 0.00 | 5.15 | 1.26 | 0.001 |
| Os.52515.1.S1_at | 0.00 | 5.15 | 2.50 | 0.014 |
| Os.53335.1.S1_at | 0.00 | 5.14 | 1.20 | 0.022 |
| Os.12347.1.S1_x_at | 0.00 | 5.14 | 1.70 | 0.006 |
| Os.22483.1.A1_at | 0.00 | 5.13 | 1.37 | 0.001 |
| Os.24834.1.S1_at | 0.01 | 5.12 | 2.25 | 0.035 |
| Os.13012.1.S1_at | 0.00 | 5.12 | 2.73 | 0.002 |
| Os.36779.1.S1_s_at | 0.00 | 5.11 | 1.60 | 0.001 |
| Os.25587.3.S1_x_at | 0.00 | 5.10 | 1.10 | 0.003 |
| Os.11573.2.A2_a_at | 0.00 | 5.08 | 12.83 | 0.007 |
| OsAffx.17491.1.S1_at | 0.00 | 5.07 | 1.58 | 0.014 |
| Os.18211.2.S1_x_at | 0.07 | 5.06 | 1.02 | 0.010 |
| Os.15428.1.S1_at | 0.00 | 5.04 | 2.42 | 0.021 |
| Os.12623.1.S1_at | 0.01 | 5.03 | 1.14 | 0.006 |
| Os.12615.1.A1_at | 0.00 | 5.03 | 1.98 | 0.040 |
| Os.2010.1.S1_at | 0.17 | 5.03 | 1.03 | 0.033 |
| Os.50823.1.S1_at | 0.00 | 5.02 | 1.26 | 0.006 |
| Os.54568.1.S1_at | 0.00 | 5.01 | 1.21 | 0.018 |
| Os.8149.1.S1_at | 0.17 | 4.99 | 1.43 | 0.014 |
| Os.53755.1.S1_at | 0.00 | 4.99 | 1.13 | 0.014 |
| Os.54895.1.S1_at | 0.00 | 4.99 | 2.48 | 0.015 |
| Os.17282.1.S1_at | 0.00 | 4.99 | 2.30 | 0.006 |
| Os.49423.1.S1_at | 0.00 | 4.98 | 1.08 | 0.002 |
| Os.6854.1.S1_at | 0.00 | 4.97 | 4.29 | 0.009 |
| Os.14444.1.S1_at | 0.00 | 4.96 | 2.06 | 0.046 |
| Os.27528.1.S1_x_at | 0.00 | 4.96 | 1.42 | 0.027 |
| Os.47358.1.A1_at | 0.00 | 4.96 | 2.33 | 0.017 |
| Os.17360.1.A1_at | 0.00 | 4.96 | 3.91 | 0.011 |
| Os.1605.1.S1_at | 0.01 | 4.95 | 1.21 | 0.000 |
| Os.10892.1.S1_at | 0.00 | 4.95 | 1.19 | 0.004 |
| Os.7451.1.S1_at | 0.00 | 4.94 | 1.28 | 0.000 |
| Os.49170.1.S1_at | 0.00 | 4.93 | 1.16 | 0.012 |
| Os.28814.1.S1_at | 0.00 | 4.92 | 1.05 | 0.003 |
| OsAffx.3560.1.S1_at | 0.00 | 4.92 | 2.26 | 0.008 |
| Os.13493.1.S1_at | 0.00 | 4.92 | 1.28 | 0.002 |
| Os.9466.1.S1_a_at | 0.00 | 4.92 | 1.07 | 0.034 |
| Os.27782.1.S1_at | 0.00 | 4.89 | 1.31 | 0.003 |
| OsAffx.32328.1.A1_at | 0.00 | 4.89 | 2.35 | 0.009 |
| Os.27454.1.S1_x_at | 0.00 | 4.88 | 1.61 | 0.044 |
| OsAffx.32313.1.S1_x_at | 0.01 | 4.87 | 1.11 | 0.023 |
| Os.9885.2.S1_x_at | 0.00 | 4.87 | 2.70 | 0.049 |
| Os.27477.1.S1_at | 0.00 | 4.84 | 1.02 | 0.027 |
| Os.11841.1.S1_at | 0.00 | 4.84 | 6.64 | 0.004 |
| Os.28976.1.S1_at | 0.00 | 4.84 | 1.49 | 0.023 |
| Os.9752.2.S1_x_at | 0.00 | 4.83 | 1.11 | 0.018 |
| Os.7679.1.S1_at | 0.00 | 4.82 | 1.66 | 0.019 |
| Os.49705.1.S1_at | 0.00 | 4.82 | 1.13 | 0.004 |
| Os.6294.1.S1_s_at | 0.00 | 4.81 | 1.01 | 0.007 |
| Os.47438.1.A1_at | 0.00 | 4.81 | 3.11 | 0.027 |
| Os.21318.1.S1_at | 0.00 | 4.80 | 1.89 | 0.014 |
| Os.8138.1.S1_at | 0.01 | 4.80 | 1.02 | 0.015 |
| Os.16324.1.S1_at | 0.01 | 4.80 | 1.11 | 0.043 |
| Os.46869.2.S1_x_at | 0.02 | 4.80 | 3.82 | 0.010 |
| Os.11736.1.S1_at | 0.00 | 4.80 | 4.91 | 0.026 |
| Os.25261.1.S1_at | 0.00 | 4.79 | 1.00 | 0.001 |
| OsAffx.18004.1.S1_x_at | 0.00 | 4.78 | 6.74 | 0.010 |
| Os.50299.1.S1_at | 0.01 | 4.78 | 1.05 | 0.002 |
| Os.21230.1.S1_at | 0.00 | 4.78 | 1.07 | 0.018 |
| Os.11844.1.S1_at | 0.00 | 4.77 | 1.61 | 0.013 |
| Os.6165.1.S1_a_at | 0.00 | 4.76 | 3.32 | 0.020 |
| Os.52229.1.S1_at | 0.00 | 4.76 | 1.01 | 0.008 |
| Os.51043.1.S1_at | 0.00 | 4.75 | 1.30 | 0.000 |
| Os.52535.1.S1_x_at | 0.00 | 4.74 | 5.88 | 0.007 |
| Os.9145.1.S1_at | 0.00 | 4.74 | 1.17 | 0.002 |
| Os.27642.1.S1_at | 0.00 | 4.72 | 3.46 | 0.007 |
| Os.12993.1.S1_at | 0.00 | 4.72 | 1.03 | 0.016 |
| Os.8900.1.S1_a_at | 0.01 | 4.72 | 1.16 | 0.005 |
| Os.53808.1.S1_x_at | 0.00 | 4.72 | 1.43 | 0.009 |
| Os.17490.1.A1_at | 0.00 | 4.71 | 1.25 | 0.001 |
| Os.5562.1.S1_at | 0.00 | 4.71 | 1.60 | 0.000 |
| Os.23613.1.S1_at | 0.07 | 4.71 | 1.11 | 0.027 |
| Os.8117.2.S1_at | 0.00 | 4.70 | 2.40 | 0.003 |
| Os.48079.1.S1_at | 0.00 | 4.69 | 1.05 | 0.001 |
| Os.12127.2.S1_x_at | 0.00 | 4.69 | 5.29 | 0.000 |
| Os.6207.1.S1_s_at | 0.01 | 4.69 | 2.00 | 0.048 |
| OsAffx.12071.1.S1_s_at | 0.00 | 4.69 | 1.51 | 0.023 |
| Os.5736.2.S1_x_at | 0.00 | 4.69 | 3.51 | 0.031 |
| Os.51804.1.S1_at | 0.00 | 4.69 | 1.40 | 0.021 |
| Os.15570.1.S1_at | 0.00 | 4.68 | 1.34 | 0.000 |
| Os.5229.1.S1_at | 0.00 | 4.67 | 4.17 | 0.024 |
| Os.11687.1.S1_a_at | 0.00 | 4.66 | 1.17 | 0.015 |
| OsAffx.23959.2.S1_at | 0.01 | 4.66 | 1.10 | 0.001 |
| Os.51211.1.S1_at | 0.00 | 4.65 | 1.32 | 0.001 |
| Os.17279.1.S1_s_at | 0.00 | 4.65 | 1.26 | 0.000 |
| Os.32177.1.S1_at | 0.00 | 4.64 | 2.80 | 0.006 |
| Os.13735.1.S1_at | 0.00 | 4.63 | 1.54 | 0.014 |
| OsAffx.32220.1.A1_s_at | 0.00 | 4.62 | 1.02 | 0.016 |
| Os.51896.1.S1_at | 0.00 | 4.61 | 1.17 | 0.001 |
| Os.9226.1.S1_a_at | 0.00 | 4.60 | 1.83 | 0.013 |
| Os.49855.1.S1_at | 0.00 | 4.60 | 1.64 | 0.005 |
| Os.17368.1.S1_at | 0.00 | 4.59 | 1.16 | 0.025 |
| Os.5261.1.S1_a_at | 0.00 | 4.58 | 1.62 | 0.003 |
| Os.50778.1.S1_at | 0.01 | 4.58 | 1.10 | 0.012 |
| Os.49087.1.A1_at | 0.00 | 4.58 | 1.57 | 0.001 |
| Os.15427.1.S1_at | 0.00 | 4.57 | 4.07 | 0.012 |
| Os.14274.1.S1_at | 0.00 | 4.57 | 1.91 | 0.011 |
| Os.46698.2.S1_x_at | 0.00 | 4.57 | 1.03 | 0.022 |
| Os.27632.1.S1_at | 0.00 | 4.56 | 1.46 | 0.000 |
| Os.7866.1.S1_at | 0.00 | 4.55 | 1.02 | 0.001 |
| Os.16724.1.S1_at | 0.00 | 4.55 | 1.22 | 0.000 |
| Os.12932.1.S1_at | 0.00 | 4.55 | 1.04 | 0.027 |
| Os.32113.1.S1_at | 0.00 | 4.55 | 1.06 | 0.001 |
| Os.6340.1.S2_s_at | 0.00 | 4.54 | 1.00 | 0.000 |
| Os.20717.1.S1_at | 0.01 | 4.53 | 1.00 | 0.038 |
| OsAffx.32324.1.A1_at | 0.00 | 4.53 | 1.23 | 0.002 |
| Os.23916.1.A1_at | 0.01 | 4.52 | 1.04 | 0.004 |
| Os.48438.1.S1_at | 0.01 | 4.51 | 1.09 | 0.036 |
| OsAffx.7764.1.S1_at | 0.00 | 4.50 | 1.03 | 0.013 |
| Os.17076.1.S1_at | 0.00 | 4.50 | 1.77 | 0.004 |
| Os.3397.1.S1_at | 0.00 | 4.50 | 1.51 | 0.003 |
| Os.15221.1.S1_a_at | 0.02 | 4.49 | 1.21 | 0.004 |
| Os.7284.1.S1_a_at | 0.00 | 4.49 | 1.56 | 0.023 |
| Os.17125.1.S1_at | 0.00 | 4.49 | 1.08 | 0.022 |
| Os.23808.2.S1_at | 0.00 | 4.49 | 1.13 | 0.018 |
| Os.17122.1.S1_at | 0.00 | 4.48 | 1.50 | 0.003 |
| Os.26570.1.S1_s_at | 0.00 | 4.48 | 1.90 | 0.028 |
| Os.35573.1.S1_at | 0.00 | 4.48 | 1.26 | 0.002 |
| Os.7029.1.S1_at | 0.00 | 4.48 | 1.07 | 0.005 |
| Os.15283.1.S1_at | 0.00 | 4.48 | 1.28 | 0.001 |
| OsAffx.24022.1.S1_x_at | 0.00 | 4.48 | 1.16 | 0.003 |
| Os.49580.1.S2_x_at | 0.00 | 4.47 | 1.07 | 0.022 |
| Os.10427.1.S1_a_at | 0.00 | 4.47 | 1.23 | 0.002 |
| Os.53789.1.S1_at | 0.00 | 4.46 | 1.16 | 0.035 |
| Os.51473.2.S1_x_at | 0.00 | 4.46 | 1.32 | 0.016 |
| Os.4859.1.S1_at | 0.00 | 4.46 | 1.50 | 0.008 |
| Os.14245.1.S1_s_at | 0.00 | 4.46 | 1.77 | 0.003 |
| Os.11824.1.S1_x_at | 0.00 | 4.46 | 1.74 | 0.002 |
| OsAffx.24269.1.S1_at | 0.00 | 4.45 | 2.74 | 0.002 |
| Os.20541.1.S1_at | 0.00 | 4.45 | 2.59 | 0.009 |
| Os.12799.1.S1_s_at | 0.00 | 4.45 | 2.23 | 0.024 |
| Os.29068.1.S1_at | 0.00 | 4.45 | 3.59 | 0.012 |
| Os.26592.2.A1_x_at | 0.00 | 4.45 | 3.16 | 0.018 |
| OsAffx.4145.1.S1_x_at | 0.02 | 4.44 | 1.06 | 0.001 |
| Os.46623.1.S1_at | 0.01 | 4.44 | 2.16 | 0.035 |
| Os.51804.2.S1_x_at | 0.01 | 4.44 | 1.24 | 0.001 |
| Os.7140.1.S1_at | 0.00 | 4.44 | 5.22 | 0.005 |
| Os.14935.1.S1_at | 0.00 | 4.43 | 5.47 | 0.010 |
| OsAffx.4527.1.S1_s_at | 0.00 | 4.43 | 2.73 | 0.025 |
| Os.17779.1.S1_at | 0.00 | 4.43 | 1.19 | 0.009 |
| Os.49814.1.S1_at | 0.00 | 4.43 | 1.25 | 0.030 |
| Os.25062.1.A1_a_at | 0.00 | 4.43 | 2.04 | 0.008 |
| Os.9279.1.S1_at | 0.00 | 4.43 | 1.44 | 0.010 |
| Os.46437.1.S1_s_at | 0.03 | 4.42 | 1.05 | 0.000 |
| Os.49474.1.S1_at | 0.00 | 4.42 | 1.12 | 0.006 |
| Os.6452.2.A1_a_at | 0.00 | 4.42 | 1.52 | 0.007 |
| Os.12371.1.S2_at | 0.00 | 4.42 | 3.69 | 0.004 |
| OsAffx.24076.1.S1_s_at | 0.00 | 4.41 | 1.12 | 0.022 |
| Os.47679.1.S1_at | 0.00 | 4.41 | 10.26 | 0.003 |
| OsAffx.2971.1.S1_at | 0.00 | 4.41 | 1.27 | 0.030 |
| Os.10011.1.S1_at | 0.00 | 4.40 | 1.15 | 0.002 |
| Os.27626.1.S1_at | 0.00 | 4.40 | 2.14 | 0.035 |
| Os.52897.1.S1_at | 0.00 | 4.40 | 1.09 | 0.019 |
| OsAffx.22147.1.S1_x_at | 0.00 | 4.40 | 1.87 | 0.022 |
| Os.26786.1.S1_at | 0.00 | 4.39 | 1.18 | 0.001 |
| Os.53213.1.S1_x_at | 0.00 | 4.38 | 1.23 | 0.007 |
| Os.17754.1.S1_at | 0.00 | 4.38 | 1.05 | 0.024 |
| Os.9818.1.S1_at | 0.00 | 4.38 | 1.58 | 0.006 |
| OsAffx.27606.1.S1_at | 0.00 | 4.37 | 1.14 | 0.011 |
| Os.12844.1.A1_a_at | 0.01 | 4.37 | 1.11 | 0.005 |
| Os.7218.1.S1_at | 0.00 | 4.37 | 2.15 | 0.001 |
| Os.50204.1.S1_at | 0.00 | 4.35 | 2.75 | 0.009 |
| Os.24860.1.S1_at | 0.00 | 4.35 | 1.14 | 0.034 |
| Os.36388.2.S1_x_at | 0.00 | 4.35 | 1.48 | 0.000 |
| Os.51208.1.S1_s_at | 0.00 | 4.34 | 2.51 | 0.013 |
| OsAffx.5257.1.S1_at | 0.00 | 4.34 | 4.48 | 0.020 |
| Os.11987.1.S1_at | 0.00 | 4.33 | 3.26 | 0.036 |
| Os.27449.1.S1_at | 0.00 | 4.33 | 1.82 | 0.001 |
| Os.35230.1.S1_at | 0.01 | 4.32 | 2.39 | 0.021 |
| Os.8479.1.S1_s_at | 0.00 | 4.32 | 1.06 | 0.017 |
| Os.26876.1.S1_at | 0.02 | 4.31 | 1.05 | 0.023 |
| Os.21784.1.S1_at | 0.00 | 4.31 | 1.54 | 0.002 |
| Os.53475.1.A1_at | 0.00 | 4.29 | 1.06 | 0.013 |
| Os.46598.1.S1_a_at | 0.00 | 4.29 | 1.89 | 0.021 |
| Os.10003.1.S1_at | 0.00 | 4.27 | 1.16 | 0.029 |
| Os.27837.1.S1_at | 0.00 | 4.27 | 1.20 | 0.041 |
| Os.27353.1.S1_at | 0.00 | 4.27 | 1.00 | 0.005 |
| Os.19165.1.S1_at | 0.00 | 4.27 | 1.17 | 0.019 |
| Os.28094.1.S1_at | 0.00 | 4.26 | 1.22 | 0.001 |
| Os.49694.1.S1_at | 0.00 | 4.26 | 1.09 | 0.016 |
| Os.5581.1.S1_at | 0.00 | 4.26 | 1.55 | 0.000 |
| Os.14817.1.S1_a_at | 0.00 | 4.26 | 1.75 | 0.004 |
| Os.5332.1.S1_at | 0.00 | 4.25 | 1.56 | 0.006 |
| Os.11990.1.S2_at | 0.00 | 4.24 | 1.02 | 0.032 |
| Os.16772.1.S1_at | 0.00 | 4.23 | 4.18 | 0.006 |
| Os.49239.1.S1_at | 0.01 | 4.23 | 1.09 | 0.011 |
| Os.5682.1.S1_at | 0.00 | 4.23 | 3.13 | 0.012 |
| Os.12696.1.S1_at | 0.00 | 4.22 | 13.62 | 0.024 |
| Os.51462.1.S1_at | 0.01 | 4.22 | 1.66 | 0.013 |
| Os.6764.2.S1_at | 0.00 | 4.22 | 1.36 | 0.003 |
| Os.46267.1.S1_x_at | 0.11 | 4.22 | 1.22 | 0.022 |
| Os.51349.1.S1_at | 0.00 | 4.22 | 1.23 | 0.030 |
| OsAffx.25398.1.S1_x_at | 0.00 | 4.22 | 1.33 | 0.032 |
| Os.36369.1.S1_at | 0.00 | 4.21 | 1.54 | 0.001 |
| Os.26549.1.S1_at | 0.00 | 4.20 | 2.49 | 0.019 |
| Os.15065.1.S1_at | 0.00 | 4.20 | 1.49 | 0.046 |
| OsAffx.18601.1.S1_at | 0.00 | 4.20 | 1.88 | 0.015 |
| Os.42075.1.A1_x_at | 0.00 | 4.19 | 1.09 | 0.000 |
| Os.27438.2.S1_x_at | 0.00 | 4.19 | 1.54 | 0.006 |
| Os.7707.1.S1_at | 0.01 | 4.19 | 1.74 | 0.013 |
| Os.19131.1.A1_at | 0.00 | 4.19 | 1.64 | 0.006 |
| Os.17917.1.S1_at | 0.00 | 4.18 | 1.71 | 0.002 |
| Os.5351.2.S1_s_at | 0.00 | 4.18 | 1.35 | 0.042 |
| Os.11573.2.A1_a_at | 0.00 | 4.18 | 18.76 | 0.004 |
| OsAffx.27597.1.S1_at | 0.00 | 4.17 | 1.29 | 0.006 |
| Os.9506.1.S1_a_at | 0.00 | 4.17 | 4.74 | 0.017 |
| Os.16121.1.S1_at | 0.01 | 4.17 | 1.43 | 0.040 |
| Os.51478.1.S1_at | 0.00 | 4.17 | 1.53 | 0.009 |
| Os.6046.1.S2_at | 0.41 | 4.17 | 1.03 | 0.009 |
| Os.7986.1.S1_at | 0.00 | 4.16 | 15.78 | 0.022 |
| Os.51926.1.S1_at | 0.00 | 4.16 | 2.08 | 0.001 |
| Os.22452.1.S1_at | 0.00 | 4.16 | 1.25 | 0.003 |
| Os.26592.2.A1_s_at | 0.00 | 4.15 | 3.67 | 0.017 |
| Os.27831.1.S1_at | 0.01 | 4.15 | 1.22 | 0.011 |
| Os.23936.1.S1_at | 0.01 | 4.14 | 1.10 | 0.007 |
| OsAffx.1926.1.S1_at | 0.03 | 4.14 | 1.11 | 0.018 |
| Os.14648.1.S1_at | 0.00 | 4.13 | 1.20 | 0.003 |
| OsAffx.16049.1.S1_at | 0.00 | 4.12 | 1.04 | 0.001 |
| OsAffx.29627.2.S1_at | 0.00 | 4.12 | 1.22 | 0.021 |
| Os.3710.1.S1_at | 0.01 | 4.12 | 1.33 | 0.018 |
| OsAffx.5582.1.S1_at | 0.00 | 4.12 | 1.34 | 0.000 |
| OsAffx.21616.1.S1_s_at | 0.00 | 4.11 | 2.71 | 0.035 |
| Os.26587.1.A1_at | 0.01 | 4.11 | 1.85 | 0.016 |
| Os.18572.1.S1_at | 0.00 | 4.10 | 3.72 | 0.001 |
| Os.9824.1.S1_at | 0.00 | 4.10 | 1.27 | 0.003 |
| Os.9752.3.S1_x_at | 0.05 | 4.10 | 1.30 | 0.019 |
| Os.49556.1.S1_at | 0.00 | 4.10 | 1.13 | 0.025 |
| Os.17268.1.S1_at | 0.00 | 4.09 | 1.90 | 0.002 |
| Os.50251.1.S1_at | 0.00 | 4.09 | 1.36 | 0.027 |
| OsAffx.11871.1.S1_s_at | 0.00 | 4.09 | 1.52 | 0.027 |
| Os.27944.1.S1_at | 0.00 | 4.08 | 1.58 | 0.003 |
| Os.13927.1.S1_at | 0.00 | 4.08 | 4.49 | 0.015 |
| Os.51374.1.S1_at | 0.00 | 4.08 | 1.24 | 0.028 |
| Os.46288.1.A1_x_at | 0.00 | 4.07 | 7.57 | 0.014 |
| Os.9765.1.S1_a_at | 0.00 | 4.07 | 1.01 | 0.010 |
| Os.7718.1.S1_at | 0.00 | 4.07 | 1.24 | 0.014 |
| Os.52538.1.S1_at | 0.00 | 4.07 | 1.02 | 0.022 |
| Os.53135.1.S1_at | 0.00 | 4.06 | 2.10 | 0.007 |
| Os.11998.1.S1_at | 0.00 | 4.06 | 1.29 | 0.004 |
| Os.17708.1.S1_at | 0.02 | 4.06 | 1.01 | 0.001 |
| OsAffx.15124.1.S1_at | 0.00 | 4.06 | 1.71 | 0.012 |
| Os.22795.1.S1_at | 0.00 | 4.05 | 1.49 | 0.027 |
| Os.38764.1.S1_x_at | 0.00 | 4.05 | 1.14 | 0.008 |
| Os.27679.1.A1_at | 0.00 | 4.05 | 3.05 | 0.001 |
| Os.9767.1.S1_at | 0.00 | 4.04 | 2.15 | 0.046 |
| Os.47826.1.S1_at | 0.00 | 4.04 | 1.31 | 0.010 |
| OsAffx.28195.1.S1_x_at | 0.00 | 4.04 | 1.12 | 0.005 |
| Os.9086.1.S1_at | 0.00 | 4.04 | 2.25 | 0.004 |
| Os.52459.1.S1_at | 0.00 | 4.03 | 2.16 | 0.000 |
| Os.9354.2.S1_at | 0.00 | 4.03 | 1.16 | 0.021 |
| Os.10849.1.S1_at | 0.00 | 4.03 | 1.99 | 0.002 |
| OsAffx.23277.2.S1_at | 0.00 | 4.03 | 2.38 | 0.028 |
| OsAffx.32210.1.A1_at | 0.00 | 4.03 | 2.12 | 0.005 |
| OsAffx.27742.1.S1_at | 0.11 | 4.02 | 1.08 | 0.014 |
| Os.14496.1.S1_at | 0.11 | 4.01 | 1.04 | 0.025 |
| Os.6225.2.S1_at | 0.05 | 4.01 | 1.27 | 0.029 |
| Os.9780.1.S1_at | 0.00 | 4.01 | 1.18 | 0.002 |
| Os.25955.1.S1_at | 0.00 | 4.01 | 1.13 | 0.015 |
| Os.24927.1.S1_at | 0.02 | 4.00 | 1.57 | 0.021 |
| Os.15734.1.S1_at | 0.00 | 4.00 | 1.08 | 0.006 |
| Os.534.2.S1_x_at | 0.00 | 4.00 | 1.98 | 0.001 |
| Os.19378.1.S1_at | 0.02 | 4.00 | 1.32 | 0.022 |
| Os.12008.1.S1_at | 0.00 | 3.99 | 2.68 | 0.003 |
| Os.21880.2.S1_x_at | 0.00 | 3.99 | 1.06 | 0.002 |
| Os.2467.1.S1_at | 0.00 | 3.99 | 1.55 | 0.010 |
| OsAffx.23277.2.S1_x_at | 0.00 | 3.99 | 2.15 | 0.033 |
| Os.27357.1.A1_at | 0.00 | 3.99 | 1.89 | 0.005 |
| Os.20465.1.S1_at | 0.00 | 3.98 | 7.55 | 0.006 |
| Os.6073.1.S1_at | 0.00 | 3.98 | 1.22 | 0.027 |
| Os.7969.2.S1_at | 0.00 | 3.98 | 1.54 | 0.026 |
| Os.5577.2.S1_a_at | 0.05 | 3.97 | 1.38 | 0.043 |
| Os.49718.1.S1_at | 0.01 | 3.97 | 1.09 | 0.003 |
| Os.44710.1.S1_at | 0.00 | 3.96 | 1.45 | 0.007 |
| Os.7304.1.S1_at | 0.00 | 3.96 | 1.34 | 0.047 |
| Os.23303.1.A1_at | 0.27 | 3.96 | 1.24 | 0.041 |
| Os.11573.5.A1_x_at | 0.00 | 3.96 | 20.24 | 0.003 |
| Os.19755.2.S1_a_at | 0.00 | 3.96 | 1.77 | 0.001 |
| Os.19539.1.S1_at | 0.00 | 3.96 | 1.70 | 0.001 |
| Os.46632.1.S1_at | 0.00 | 3.96 | 1.78 | 0.039 |
| Os.17759.1.A1_s_at | 0.00 | 3.95 | 1.08 | 0.011 |
| Os.15183.1.S1_at | 0.00 | 3.95 | 2.58 | 0.007 |
| Os.5136.1.S1_at | 0.00 | 3.94 | 1.29 | 0.001 |
| Os.16582.1.S1_at | 0.00 | 3.93 | 1.29 | 0.007 |
| Os.6595.1.S1_a_at | 0.00 | 3.93 | 2.38 | 0.021 |
| Os.33770.1.S1_at | 0.03 | 3.93 | 1.20 | 0.005 |
| OsAffx.11847.1.S1_at | 0.00 | 3.92 | 1.03 | 0.045 |
| Os.27862.1.S1_at | 0.00 | 3.92 | 3.39 | 0.001 |
| Os.10141.1.S1_at | 0.00 | 3.92 | 1.14 | 0.013 |
| Os.12134.1.S1_at | 0.00 | 3.92 | 1.71 | 0.000 |
| Os.5986.1.S1_at | 0.00 | 3.91 | 1.96 | 0.009 |
| Os.27967.1.A1_at | 0.00 | 3.90 | 1.03 | 0.043 |
| Os.7691.1.S1_s_at | 0.00 | 3.89 | 1.14 | 0.003 |
| Os.27322.1.S1_x_at | 0.01 | 3.89 | 2.00 | 0.026 |
| Os.23434.1.S1_s_at | 0.00 | 3.89 | 1.15 | 0.028 |
| Os.1606.2.S1_a_at | 0.00 | 3.89 | 1.30 | 0.010 |
| Os.14870.1.S1_at | 0.00 | 3.89 | 1.89 | 0.034 |
| Os.24711.1.A1_at | 0.00 | 3.89 | 1.22 | 0.006 |
| Os.13493.1.S1_s_at | 0.00 | 3.89 | 1.35 | 0.001 |
| Os.11281.1.S2_at | 0.00 | 3.88 | 1.85 | 0.037 |
| Os.8611.1.S1_at | 0.01 | 3.88 | 1.66 | 0.010 |
| Os.23606.1.S1_at | 0.00 | 3.88 | 1.15 | 0.028 |
| Os.5377.1.S1_at | 0.00 | 3.87 | 2.78 | 0.008 |
| Os.37894.1.S1_at | 0.00 | 3.87 | 1.27 | 0.004 |
| Os.33788.1.S1_at | 0.00 | 3.87 | 1.19 | 0.004 |
| Os.3507.1.S1_at | 0.00 | 3.87 | 1.11 | 0.006 |
| Os.33770.1.S1_s_at | 0.00 | 3.86 | 1.18 | 0.002 |
| Os.46548.2.S1_at | 0.00 | 3.86 | 1.05 | 0.005 |
| Os.17435.2.S1_at | 0.01 | 3.86 | 1.16 | 0.048 |
| Os.27079.1.A1_at | 0.00 | 3.86 | 1.73 | 0.001 |
| Os.57558.1.S1_at | 0.00 | 3.86 | 1.94 | 0.026 |
| Os.11429.1.S1_at | 0.00 | 3.86 | 1.12 | 0.002 |
| OsAffx.11955.1.S1_at | 0.00 | 3.85 | 1.75 | 0.023 |
| Os.10727.1.S1_at | 0.00 | 3.84 | 1.18 | 0.003 |
| Os.5191.1.S1_at | 0.00 | 3.84 | 1.14 | 0.006 |
| OsAffx.7038.1.S1_s_at | 0.00 | 3.83 | 1.47 | 0.014 |
| OsAffx.14661.1.S1_s_at | 0.01 | 3.83 | 1.58 | 0.032 |
| Os.27742.2.S1_a_at | 0.02 | 3.83 | 1.04 | 0.006 |
| Os.18850.1.S1_at | 0.00 | 3.83 | 1.02 | 0.019 |
| Os.46591.2.S1_x_at | 0.00 | 3.83 | 1.19 | 0.001 |
| Os.27127.1.S1_a_at | 0.05 | 3.82 | 1.04 | 0.014 |
| Os.5632.1.S1_a_at | 0.00 | 3.82 | 2.23 | 0.034 |
| Os.11927.1.S1_x_at | 0.00 | 3.81 | 4.12 | 0.007 |
| Os.5518.1.S1_at | 0.00 | 3.81 | 2.87 | 0.002 |
| Os.10120.1.S2_a_at | 0.00 | 3.79 | 1.01 | 0.031 |
| Os.12738.1.S2_a_at | 0.11 | 3.78 | 1.01 | 0.013 |
| Os.32506.1.S1_at | 0.00 | 3.78 | 1.44 | 0.020 |
| Os.35827.1.S1_at | 0.01 | 3.78 | 1.03 | 0.031 |
| Os.38378.1.S1_a_at | 0.00 | 3.77 | 2.16 | 0.049 |
| Os.6248.1.S1_s_at | 0.00 | 3.77 | 1.10 | 0.003 |
| Os.27085.1.A1_at | 0.01 | 3.77 | 1.43 | 0.048 |
| Os.12865.1.S1_at | 0.00 | 3.77 | 1.14 | 0.048 |
| Os.8014.1.S1_at | 0.00 | 3.77 | 4.99 | 0.045 |
| Os.5960.1.S1_at | 0.00 | 3.76 | 1.80 | 0.009 |
| Os.15722.1.S1_s_at | 0.02 | 3.76 | 1.39 | 0.009 |
| OsAffx.16018.1.S1_at | 0.00 | 3.76 | 1.29 | 0.013 |
| Os.52476.1.S1_at | 0.01 | 3.76 | 1.19 | 0.010 |
| OsAffx.19737.1.S1_x_at | 0.00 | 3.75 | 1.24 | 0.009 |
| Os.27663.1.A1_at | 0.00 | 3.75 | 1.09 | 0.002 |
| Os.53108.1.S1_at | 0.00 | 3.75 | 1.11 | 0.009 |
| OsAffx.32256.1.S1_x_at | 0.00 | 3.75 | 2.93 | 0.039 |
| OsAffx.26103.1.S1_at | 0.00 | 3.75 | 1.72 | 0.022 |
| Os.27520.1.S1_at | 0.00 | 3.74 | 2.15 | 0.015 |
| OsAffx.17904.1.S1_at | 0.00 | 3.74 | 1.05 | 0.011 |
| Os.52104.1.S1_at | 0.01 | 3.74 | 1.40 | 0.031 |
| Os.29736.1.S1_at | 0.00 | 3.74 | 1.05 | 0.017 |
| Os.46548.1.S1_at | 0.05 | 3.74 | 1.35 | 0.002 |
| Os.17091.1.S1_at | 0.00 | 3.74 | 1.75 | 0.002 |
| Os.46383.2.A1_s_at | 0.00 | 3.74 | 14.56 | 0.007 |
| Os.51222.1.S1_at | 0.00 | 3.73 | 1.20 | 0.031 |
| Os.47913.1.A1_at | 0.00 | 3.73 | 1.36 | 0.001 |
| OsAffx.4648.1.S1_at | 0.03 | 3.72 | 1.17 | 0.023 |
| Os.1150.1.S1_at | 0.00 | 3.72 | 1.19 | 0.031 |
| Os.12484.1.S1_at | 0.00 | 3.72 | 1.27 | 0.040 |
| Os.27773.1.S1_at | 0.01 | 3.72 | 1.03 | 0.046 |
| Os.8081.1.A1_at | 0.00 | 3.72 | 1.71 | 0.012 |
| Os.23146.1.S1_at | 0.01 | 3.72 | 1.37 | 0.014 |
| Os.27425.1.S1_at | 0.00 | 3.72 | 1.02 | 0.001 |
| Os.26592.3.S1_x_at | 0.00 | 3.71 | 3.47 | 0.027 |
| Os.17487.1.S1_at | 0.00 | 3.71 | 3.05 | 0.006 |
| Os.5311.1.S1_at | 0.00 | 3.71 | 2.01 | 0.015 |
| Os.54895.1.S1_x_at | 0.01 | 3.71 | 1.38 | 0.034 |
| Os.52775.1.S1_at | 0.00 | 3.71 | 1.38 | 0.002 |
| Os.26537.2.S1_x_at | 0.00 | 3.71 | 1.81 | 0.030 |
| Os.38048.1.S1_a_at | 0.00 | 3.70 | 1.18 | 0.014 |
| OsAffx.32225.1.S1_x_at | 0.05 | 3.70 | 1.29 | 0.038 |
| Os.17959.1.S1_a_at | 0.00 | 3.70 | 1.46 | 0.022 |
| Os.11805.1.A1_s_at | 0.00 | 3.69 | 1.77 | 0.024 |
| Os.50976.1.S1_at | 0.00 | 3.69 | 1.27 | 0.026 |
| Os.17549.1.S1_at | 0.00 | 3.69 | 1.26 | 0.014 |
| Os.7914.2.S1_at | 0.00 | 3.68 | 1.25 | 0.002 |
| OsAffx.29059.1.S1_s_at | 0.00 | 3.68 | 1.23 | 0.030 |
| Os.27221.1.A1_at | 0.00 | 3.68 | 1.38 | 0.002 |
| Os.11990.1.S1_a_at | 0.00 | 3.68 | 1.91 | 0.032 |
| Os.24327.1.A1_at | 0.01 | 3.67 | 1.25 | 0.039 |
| Os.54722.1.S1_at | 0.11 | 3.67 | 1.20 | 0.018 |
| Os.9167.1.A1_at | 0.03 | 3.67 | 1.01 | 0.001 |
| Os.27146.1.A1_at | 0.03 | 3.66 | 1.19 | 0.031 |
| Os.7760.1.S1_at | 0.00 | 3.66 | 1.47 | 0.033 |
| Os.17778.1.S1_at | 0.03 | 3.66 | 1.18 | 0.020 |
| Os.33534.1.S1_s_at | 0.02 | 3.66 | 1.81 | 0.010 |
| Os.21398.1.S1_at | 0.00 | 3.66 | 1.06 | 0.038 |
| Os.26654.1.S1_at | 0.00 | 3.65 | 1.02 | 0.047 |
| Os.28203.1.S1_a_at | 0.00 | 3.65 | 5.70 | 0.049 |
| OsAffx.26382.1.S1_at | 0.00 | 3.65 | 1.25 | 0.004 |
| Os.22029.1.S1_a_at | 0.00 | 3.65 | 2.07 | 0.000 |
| Os.4917.1.S1_at | 0.00 | 3.65 | 1.20 | 0.001 |
| Os.54897.1.S1_at | 0.05 | 3.65 | 1.03 | 0.000 |
| Os.5401.1.S1_at | 0.00 | 3.65 | 2.63 | 0.002 |
| Os.53453.1.S1_at | 0.00 | 3.64 | 7.10 | 0.002 |
| Os.13874.1.S1_x_at | 0.00 | 3.64 | 1.13 | 0.004 |
| Os.12065.1.S1_at | 0.00 | 3.64 | 1.05 | 0.007 |
| Os.32172.1.S1_at | 0.00 | 3.64 | 1.13 | 0.045 |
| Os.6271.1.S1_at | 0.00 | 3.63 | 1.82 | 0.002 |
| OsAffx.3006.1.S1_at | 0.17 | 3.63 | 1.08 | 0.039 |
| Os.24334.1.A1_at | 0.02 | 3.63 | 1.44 | 0.009 |
| OsAffx.5487.1.S1_at | 0.00 | 3.63 | 1.49 | 0.016 |
| Os.21192.2.S1_at | 0.00 | 3.63 | 1.48 | 0.010 |
| OsAffx.22476.1.S1_x_at | 0.05 | 3.62 | 1.36 | 0.043 |
| Os.20911.1.S1_at | 0.01 | 3.62 | 1.07 | 0.009 |
| Os.6830.1.S1_at | 0.00 | 3.62 | 2.21 | 0.008 |
| Os.11860.1.S1_at | 0.00 | 3.61 | 1.15 | 0.030 |
| Os.26271.1.A1_s_at | 0.01 | 3.61 | 1.32 | 0.004 |
| Os.23673.1.S1_at | 0.00 | 3.61 | 1.79 | 0.026 |
| Os.35409.1.A1_a_at | 0.00 | 3.61 | 1.32 | 0.021 |
| Os.38365.1.S1_a_at | 0.00 | 3.60 | 2.56 | 0.022 |
| Os.9367.1.S1_a_at | 0.03 | 3.60 | 1.03 | 0.001 |
| Os.27322.2.S1_at | 0.00 | 3.60 | 1.78 | 0.044 |
| Os.15436.1.S1_at | 0.00 | 3.60 | 1.00 | 0.023 |
| Os.5136.1.S1_a_at | 0.00 | 3.60 | 1.44 | 0.002 |
| Os.26832.1.S1_at | 0.00 | 3.60 | 1.01 | 0.001 |
| OsAffx.11954.1.S1_s_at | 0.00 | 3.60 | 1.95 | 0.013 |
| Os.26535.1.S1_x_at | 0.03 | 3.59 | 1.63 | 0.017 |
| Os.11136.1.S1_at | 0.00 | 3.59 | 1.25 | 0.000 |
| OsAffx.16049.1.S1_x_at | 0.00 | 3.59 | 1.11 | 0.000 |
| OsAffx.4277.1.S1_s_at | 0.02 | 3.58 | 1.25 | 0.033 |
| Os.20476.2.S1_a_at | 0.41 | 3.58 | 1.04 | 0.013 |
| OsAffx.7876.1.S1_s_at | 0.07 | 3.58 | 1.18 | 0.042 |
| Os.18955.1.S1_at | 0.05 | 3.57 | 1.25 | 0.013 |
| Os.1798.1.S1_at | 0.01 | 3.56 | 1.03 | 0.038 |
| OsAffx.32268.1.A1_x_at | 0.01 | 3.56 | 1.04 | 0.033 |
| Os.53776.1.S1_at | 0.00 | 3.56 | 1.02 | 0.010 |
| Os.10087.1.S1_at | 0.05 | 3.56 | 1.07 | 0.009 |
| Os.10700.1.S1_at | 0.00 | 3.55 | 1.55 | 0.014 |
| Os.28045.1.S1_at | 0.00 | 3.55 | 1.26 | 0.002 |
| Os.26040.1.S1_at | 0.00 | 3.54 | 1.85 | 0.004 |
| Os.11970.2.S1_x_at | 0.00 | 3.54 | 1.05 | 0.027 |
| Os.45906.1.S1_at | 0.00 | 3.54 | 1.02 | 0.002 |
| Os.51391.1.S1_at | 0.00 | 3.54 | 1.10 | 0.021 |
| Os.19752.1.S1_at | 0.11 | 3.54 | 1.07 | 0.044 |
| Os.14058.1.S1_at | 0.00 | 3.54 | 1.73 | 0.005 |
| Os.5136.2.S1_x_at | 0.00 | 3.53 | 1.26 | 0.000 |
| Os.52821.1.S1_at | 0.00 | 3.53 | 1.90 | 0.016 |
| OsAffx.16139.1.S1_at | 0.00 | 3.52 | 1.33 | 0.000 |
| Os.7165.1.S1_at | 0.01 | 3.52 | 1.15 | 0.004 |
| Os.28089.1.S2_at | 0.00 | 3.52 | 1.73 | 0.023 |
| Os.34459.1.S1_at | 0.00 | 3.52 | 1.68 | 0.020 |
| Os.20642.1.S1_at | 0.00 | 3.52 | 1.32 | 0.028 |
| Os.26592.1.S1_s_at | 0.00 | 3.52 | 2.88 | 0.035 |
| Os.27914.1.S1_at | 0.00 | 3.52 | 1.28 | 0.008 |
| Os.26686.1.S1_a_at | 0.00 | 3.51 | 1.29 | 0.002 |
| Os.10613.1.S1_at | 0.00 | 3.51 | 1.57 | 0.003 |
| Os.36435.2.S1_x_at | 0.00 | 3.51 | 1.51 | 0.016 |
| OsAffx.19033.1.S1_at | 0.01 | 3.51 | 1.22 | 0.021 |
| Os.4291.1.S1_at | 0.17 | 3.51 | 1.13 | 0.044 |
| Os.11630.1.S1_at | 0.03 | 3.51 | 1.02 | 0.004 |
| Os.36346.2.S1_at | 0.00 | 3.51 | 1.09 | 0.014 |
| Os.25202.1.S1_at | 0.00 | 3.51 | 1.49 | 0.025 |
| OsAffx.32195.1.S1_x_at | 0.05 | 3.50 | 1.36 | 0.032 |
| Os.46883.1.S1_at | 0.00 | 3.50 | 1.12 | 0.013 |
| Os.8712.1.S2_at | 0.01 | 3.50 | 1.01 | 0.008 |
| Os.10501.1.S1_at | 0.11 | 3.50 | 1.13 | 0.005 |
| Os.21254.1.S1_at | 0.01 | 3.49 | 1.23 | 0.013 |
| OsAffx.4104.1.S1_at | 0.00 | 3.49 | 1.17 | 0.038 |
| Os.9908.1.S1_at | 0.03 | 3.49 | 1.12 | 0.045 |
| Os.23847.1.S1_at | 0.00 | 3.49 | 1.99 | 0.021 |
| Os.7020.1.S1_x_at | 0.01 | 3.48 | 1.10 | 0.013 |
| OsAffx.32240.1.A1_at | 0.07 | 3.48 | 1.24 | 0.025 |
| Os.20461.1.S2_s_at | 0.00 | 3.48 | 1.36 | 0.049 |
| Os.6384.1.S1_at | 0.00 | 3.48 | 1.00 | 0.031 |
| Os.27635.1.S1_at | 0.01 | 3.48 | 1.25 | 0.026 |
| Os.7994.1.S1_at | 0.00 | 3.48 | 2.43 | 0.007 |
| Os.19155.1.S1_at | 0.07 | 3.47 | 1.25 | 0.029 |
| Os.27652.1.S1_at | 0.00 | 3.47 | 1.69 | 0.019 |
| Os.8571.1.S1_s_at | 0.00 | 3.47 | 2.93 | 0.003 |
| Os.55208.1.S1_at | 0.00 | 3.46 | 1.33 | 0.017 |
| Os.15711.1.S1_at | 0.27 | 3.46 | 1.41 | 0.012 |
| OsAffx.11628.1.S1_x_at | 0.00 | 3.46 | 1.46 | 0.012 |
| OsAffx.26722.2.S1_s_at | 0.00 | 3.46 | 1.12 | 0.035 |
| Os.24660.1.S1_at | 0.01 | 3.46 | 1.19 | 0.000 |
| Os.47073.1.S1_at | 0.00 | 3.45 | 1.10 | 0.031 |
| Os.26688.1.S1_s_at | 0.01 | 3.45 | 1.03 | 0.002 |
| Os.55007.1.S1_x_at | 0.02 | 3.45 | 1.13 | 0.010 |
| Os.11935.1.S1_at | 0.02 | 3.44 | 1.64 | 0.016 |
| OsAffx.5460.1.S1_at | 0.00 | 3.44 | 1.33 | 0.049 |
| Os.16076.1.S1_s_at | 0.01 | 3.44 | 1.00 | 0.012 |
| Os.37047.1.S1_at | 0.00 | 3.44 | 1.28 | 0.003 |
| Os.6334.1.S1_at | 0.01 | 3.44 | 1.19 | 0.001 |
| Os.14404.1.S1_at | 0.01 | 3.44 | 1.06 | 0.004 |
| Os.11458.1.S1_at | 0.00 | 3.44 | 1.28 | 0.028 |
| Os.7626.1.S1_at | 0.01 | 3.44 | 1.11 | 0.021 |
| Os.11364.1.S1_at | 0.00 | 3.43 | 1.26 | 0.008 |
| Os.46739.1.A1_x_at | 0.17 | 3.43 | 1.39 | 0.027 |
| Os.35811.1.S1_at | 0.01 | 3.42 | 1.05 | 0.009 |
| Os.53486.1.S1_at | 0.00 | 3.42 | 1.07 | 0.046 |
| Os.19014.1.S1_at | 0.00 | 3.41 | 1.09 | 0.010 |
| Os.6230.1.S1_at | 0.02 | 3.41 | 1.18 | 0.001 |
| Os.53001.1.S1_at | 0.00 | 3.41 | 1.44 | 0.043 |
| Os.7948.1.S1_a_at | 0.00 | 3.41 | 2.15 | 0.006 |
| Os.33708.1.S1_s_at | 0.00 | 3.40 | 1.05 | 0.011 |
| Os.52385.1.S1_at | 0.01 | 3.40 | 1.21 | 0.036 |
| Os.46321.1.A1_s_at | 0.00 | 3.40 | 1.24 | 0.001 |
| Os.14271.1.S1_at | 0.00 | 3.40 | 1.61 | 0.023 |
| Os.10862.1.S1_at | 0.00 | 3.40 | 1.28 | 0.018 |
| Os.28030.2.A1_at | 0.00 | 3.40 | 1.14 | 0.050 |
| Os.11222.1.S1_at | 0.00 | 3.40 | 1.04 | 0.006 |
| Os.52679.1.S1_at | 0.27 | 3.40 | 1.11 | 0.032 |
| Os.24683.1.A1_s_at | 0.00 | 3.40 | 1.84 | 0.005 |
| Os.55604.1.S1_at | 0.00 | 3.39 | 1.34 | 0.007 |
| Os.21367.1.S1_at | 0.00 | 3.39 | 1.29 | 0.028 |
| Os.21406.1.A1_at | 0.02 | 3.39 | 1.59 | 0.045 |
| Os.10410.1.S1_at | 0.00 | 3.39 | 10.63 | 0.000 |
| Os.32449.1.S1_at | 0.00 | 3.39 | 2.84 | 0.002 |
| Os.7969.1.S1_x_at | 0.00 | 3.39 | 1.26 | 0.032 |
| Os.32072.1.S1_at | 0.00 | 3.38 | 1.20 | 0.002 |
| Os.26642.1.S1_a_at | 0.00 | 3.38 | 1.72 | 0.000 |
| Os.27759.1.S1_at | 0.00 | 3.37 | 1.34 | 0.008 |
| Os.3407.1.S1_a_at | 0.00 | 3.37 | 3.96 | 0.050 |
| Os.50857.1.S1_at | 0.02 | 3.37 | 1.11 | 0.004 |
| Os.11041.1.S1_s_at | 0.00 | 3.37 | 1.26 | 0.009 |
| Os.8457.1.S1_at | 0.00 | 3.37 | 2.36 | 0.026 |
| Os.17490.2.A1_a_at | 0.00 | 3.36 | 1.52 | 0.012 |
| Os.54097.1.A1_at | 0.02 | 3.36 | 1.24 | 0.012 |
| Os.8213.1.S1_at | 0.00 | 3.36 | 1.57 | 0.012 |
| Os.51057.1.S1_at | 0.01 | 3.35 | 1.52 | 0.030 |
| Os.5651.1.S1_a_at | 0.00 | 3.34 | 1.17 | 0.011 |
| Os.27454.2.S1_at | 0.00 | 3.34 | 1.10 | 0.032 |
| Os.5739.1.S1_at | 0.00 | 3.34 | 1.60 | 0.012 |
| Os.51926.1.S1_x_at | 0.00 | 3.34 | 1.82 | 0.004 |
| Os.7282.1.S1_at | 0.05 | 3.34 | 1.05 | 0.016 |
| Os.20540.1.S1_at | 0.00 | 3.34 | 1.34 | 0.010 |
| OsAffx.13195.1.S1_at | 0.00 | 3.33 | 1.28 | 0.002 |
| Os.28094.1.S1_s_at | 0.00 | 3.33 | 1.43 | 0.003 |
| Os.10320.1.S1_x_at | 0.00 | 3.33 | 1.08 | 0.019 |
| OsAffx.31580.1.S1_x_at | 0.00 | 3.33 | 1.59 | 0.001 |
| Os.46634.1.S1_at | 0.00 | 3.33 | 1.17 | 0.024 |
| Os.7977.1.S1_at | 0.00 | 3.33 | 1.01 | 0.020 |
| Os.53702.1.S1_x_at | 0.00 | 3.32 | 2.69 | 0.001 |
| Os.22472.1.S1_x_at | 0.01 | 3.32 | 1.01 | 0.012 |
| Os.11810.2.S1_a_at | 0.00 | 3.32 | 1.68 | 0.014 |
| Os.17625.1.S1_at | 0.05 | 3.32 | 1.23 | 0.014 |
| Os.19335.1.S1_at | 0.00 | 3.32 | 1.64 | 0.006 |
| OsAffx.32221.1.A1_s_at | 0.02 | 3.32 | 1.70 | 0.050 |
| Os.26397.1.S1_at | 0.01 | 3.31 | 1.13 | 0.002 |
| OsAffx.32206.1.S1_at | 0.05 | 3.31 | 1.09 | 0.018 |
| Os.23349.2.S1_at | 0.00 | 3.31 | 1.20 | 0.008 |
| OsAffx.32257.1.A1_at | 0.00 | 3.30 | 1.55 | 0.010 |
| Os.14875.1.S1_at | 0.00 | 3.30 | 1.04 | 0.009 |
| Os.11622.1.S1_at | 0.01 | 3.29 | 1.42 | 0.032 |
| Os.26761.1.S1_s_at | 0.00 | 3.29 | 2.56 | 0.014 |
| Os.26974.1.S1_at | 0.00 | 3.29 | 1.40 | 0.012 |
| Os.11866.1.S1_at | 0.00 | 3.29 | 1.08 | 0.041 |
| Os.49832.1.S1_at | 0.01 | 3.29 | 1.16 | 0.022 |
| Os.12363.1.S1_at | 0.05 | 3.28 | 1.11 | 0.023 |
| Os.11929.1.S1_at | 0.00 | 3.28 | 1.92 | 0.041 |
| Os.14151.1.S1_at | 0.00 | 3.28 | 1.51 | 0.007 |
| Os.46386.1.S1_at | 0.00 | 3.28 | 2.21 | 0.045 |
| Os.24684.1.S1_at | 0.00 | 3.28 | 1.32 | 0.022 |
| Os.26761.2.S1_x_at | 0.00 | 3.28 | 2.62 | 0.012 |
| Os.34818.1.S1_x_at | 0.07 | 3.27 | 1.13 | 0.012 |
| Os.55007.1.S1_at | 0.01 | 3.27 | 1.11 | 0.004 |
| OsAffx.12906.1.S1_at | 0.00 | 3.27 | 1.32 | 0.017 |
| Os.38048.2.S1_x_at | 0.00 | 3.26 | 1.12 | 0.004 |
| Os.28459.1.S1_at | 0.01 | 3.26 | 1.32 | 0.026 |
| Os.9104.1.S1_at | 0.03 | 3.26 | 1.12 | 0.024 |
| Os.27758.1.S1_at | 0.00 | 3.26 | 1.74 | 0.008 |
| Os.39020.1.S1_at | 0.05 | 3.26 | 1.34 | 0.019 |
| Os.14762.1.S1_x_at | 0.01 | 3.25 | 1.07 | 0.002 |
| Os.10220.1.S1_at | 0.01 | 3.25 | 1.31 | 0.009 |
| Os.50951.1.S1_at | 0.01 | 3.24 | 2.17 | 0.010 |
| Os.37415.1.S1_at | 0.05 | 3.24 | 1.50 | 0.004 |
| Os.38257.1.S1_x_at | 0.00 | 3.23 | 5.56 | 0.012 |
| Os.22579.1.S1_at | 0.00 | 3.23 | 1.94 | 0.001 |
| OsAffx.31942.1.S1_at | 0.00 | 3.23 | 1.17 | 0.017 |
| Os.57099.1.S1_at | 0.00 | 3.23 | 1.34 | 0.031 |
| Os.11995.1.S1_at | 0.00 | 3.22 | 1.12 | 0.000 |
| Os.49228.1.S1_at | 0.00 | 3.22 | 1.55 | 0.012 |
| Os.20068.1.S1_at | 0.07 | 3.22 | 1.00 | 0.021 |
| Os.38416.1.A1_at | 0.00 | 3.21 | 1.42 | 0.014 |
| Os.52574.1.S1_at | 0.00 | 3.21 | 1.62 | 0.009 |
| Os.43898.1.S1_x_at | 0.11 | 3.21 | 1.12 | 0.028 |
| OsAffx.17904.1.S1_s_at | 0.00 | 3.21 | 1.19 | 0.001 |
| Os.52427.1.S1_at | 0.01 | 3.21 | 1.18 | 0.004 |
| Os.16067.1.S1_at | 0.00 | 3.20 | 1.01 | 0.024 |
| Os.54727.1.S1_s_at | 0.01 | 3.20 | 1.40 | 0.045 |
| Os.7752.1.S1_at | 0.00 | 3.20 | 1.12 | 0.003 |
| Os.7308.1.S1_x_at | 0.03 | 3.20 | 1.06 | 0.008 |
| Os.5694.1.S1_at | 0.00 | 3.20 | 2.87 | 0.004 |
| Os.8554.1.S1_at | 0.00 | 3.20 | 1.20 | 0.000 |
| Os.33670.3.S1_s_at | 0.01 | 3.20 | 1.32 | 0.002 |
| Os.10696.1.S1_at | 0.00 | 3.20 | 2.34 | 0.014 |
| Os.14941.2.S1_a_at | 0.00 | 3.19 | 1.23 | 0.007 |
| Os.35677.1.S1_at | 0.01 | 3.19 | 1.31 | 0.048 |
| OsAffx.27291.1.S1_at | 0.00 | 3.19 | 2.39 | 0.003 |
| Os.12837.1.S1_at | 0.00 | 3.19 | 3.32 | 0.044 |
| Os.27322.3.S1_x_at | 0.00 | 3.19 | 1.35 | 0.047 |
| Os.22472.2.S1_at | 0.01 | 3.19 | 1.33 | 0.019 |
| Os.46548.2.S1_x_at | 0.00 | 3.19 | 1.09 | 0.003 |
| Os.54151.1.S1_x_at | 0.00 | 3.19 | 1.03 | 0.026 |
| Os.20461.1.S1_at | 0.00 | 3.18 | 1.36 | 0.045 |
| Os.28450.1.S1_at | 0.01 | 3.18 | 1.06 | 0.029 |
| Os.11652.1.S1_at | 0.17 | 3.17 | 1.33 | 0.017 |
| OsAffx.17919.1.S1_at | 0.00 | 3.17 | 1.44 | 0.028 |
| Os.51371.1.S1_at | 0.00 | 3.17 | 5.47 | 0.015 |
| Os.19035.1.S1_at | 0.00 | 3.17 | 1.49 | 0.003 |
| Os.53764.1.S1_at | 0.01 | 3.17 | 1.13 | 0.007 |
| Os.1148.1.S1_at | 0.00 | 3.17 | 1.08 | 0.020 |
| Os.9021.1.S1_at | 0.00 | 3.17 | 1.25 | 0.001 |
| Os.34151.1.S2_a_at | 0.00 | 3.16 | 1.40 | 0.017 |
| Os.46525.1.S1_at | 0.01 | 3.16 | 1.60 | 0.023 |
| Os.11976.1.S1_a_at | 0.00 | 3.16 | 4.09 | 0.019 |
| Os.49084.1.S1_at | 0.05 | 3.15 | 1.11 | 0.018 |
| Os.28438.4.S1_x_at | 0.00 | 3.15 | 4.20 | 0.001 |
| Os.49726.1.S1_at | 0.00 | 3.15 | 2.05 | 0.006 |
| Os.6636.1.S1_at | 0.00 | 3.15 | 3.01 | 0.001 |
| Os.23416.1.S1_x_at | 0.00 | 3.14 | 1.37 | 0.041 |
| Os.23313.1.S1_x_at | 0.00 | 3.14 | 1.10 | 0.024 |
| Os.10779.1.S1_at | 0.11 | 3.14 | 1.04 | 0.001 |
| Os.46382.3.S1_at | 0.00 | 3.14 | 1.14 | 0.016 |
| Os.26770.2.S1_x_at | 0.00 | 3.13 | 2.21 | 0.024 |
| Os.18608.1.S1_at | 0.00 | 3.13 | 1.05 | 0.002 |
| Os.52638.1.S1_at | 0.00 | 3.12 | 1.28 | 0.002 |
| Os.49624.1.S1_at | 0.00 | 3.12 | 1.59 | 0.000 |
| Os.12053.1.S1_at | 0.00 | 3.12 | 1.15 | 0.006 |
| Os.19474.1.S1_a_at | 0.00 | 3.12 | 1.43 | 0.029 |
| Os.14286.1.S1_at | 0.17 | 3.12 | 1.06 | 0.045 |
| Os.34982.1.A1_at | 0.00 | 3.12 | 1.54 | 0.010 |
| Os.19844.1.S1_at | 0.00 | 3.11 | 2.28 | 0.047 |
| OsAffx.17231.1.S1_at | 0.00 | 3.11 | 1.29 | 0.014 |
| Os.1500.2.S1_x_at | 0.01 | 3.11 | 1.12 | 0.013 |
| Os.27024.1.S1_at | 0.00 | 3.11 | 1.51 | 0.001 |
| Os.8921.1.S1_at | 0.00 | 3.11 | 1.47 | 0.001 |
| Os.4148.1.S2_a_at | 0.00 | 3.10 | 1.73 | 0.003 |
| OsAffx.27242.1.S1_x_at | 0.01 | 3.10 | 1.08 | 0.011 |
| Os.7631.1.S1_at | 0.00 | 3.10 | 1.72 | 0.019 |
| Os.17390.1.S1_at | 0.01 | 3.10 | 1.03 | 0.016 |
| Os.50865.1.S1_at | 0.00 | 3.10 | 1.50 | 0.018 |
| Os.10068.1.S1_at | 0.00 | 3.10 | 1.39 | 0.005 |
| Os.46361.1.A1_at | 0.00 | 3.09 | 1.33 | 0.024 |
| OsAffx.4156.1.S1_at | 0.00 | 3.09 | 1.06 | 0.005 |
| Os.17692.1.S1_at | 0.01 | 3.09 | 1.16 | 0.017 |
| Os.11313.1.S1_at | 0.05 | 3.09 | 1.18 | 0.045 |
| Os.4180.1.S1_at | 0.00 | 3.09 | 1.43 | 0.019 |
| Os.34206.1.S1_at | 0.00 | 3.08 | 1.57 | 0.027 |
| Os.56323.1.S1_at | 0.01 | 3.08 | 1.07 | 0.001 |
| Os.28462.1.S1_s_at | 0.00 | 3.08 | 5.75 | 0.041 |
| Os.7756.2.S1_x_at | 0.17 | 3.07 | 1.08 | 0.046 |
| Os.3400.1.S1_s_at | 0.07 | 3.07 | 1.01 | 0.035 |
| Os.6824.1.S1_at | 0.00 | 3.07 | 1.14 | 0.008 |
| Os.10080.1.S1_at | 0.01 | 3.07 | 1.26 | 0.021 |
| Os.2914.1.S1_at | 0.00 | 3.07 | 2.74 | 0.026 |
| Os.51732.1.S1_at | 0.00 | 3.06 | 3.31 | 0.003 |
| Os.11253.1.S1_at | 0.00 | 3.06 | 2.06 | 0.014 |
| Os.13734.1.S1_s_at | 0.00 | 3.06 | 1.20 | 0.036 |
| Os.26825.1.S1_at | 0.00 | 3.06 | 1.19 | 0.009 |
| Os.39065.1.A1_s_at | 0.00 | 3.06 | 1.10 | 0.004 |
| Os.47399.1.A1_at | 0.00 | 3.05 | 1.01 | 0.001 |
| Os.9166.4.S1_x_at | 0.17 | 3.05 | 1.44 | 0.039 |
| Os.26408.3.S1_x_at | 0.01 | 3.05 | 1.38 | 0.036 |
| OsAffx.24885.1.S1_at | 0.07 | 3.05 | 1.04 | 0.012 |
| Os.9097.1.A1_at | 0.01 | 3.04 | 1.17 | 0.012 |
| Os.50763.1.S1_at | 0.00 | 3.04 | 3.75 | 0.038 |
| OsAffx.2388.1.S1_at | 0.02 | 3.04 | 1.44 | 0.010 |
| Os.7552.1.S1_at | 0.00 | 3.04 | 1.27 | 0.003 |
| Os.35613.1.S1_at | 0.01 | 3.04 | 1.63 | 0.024 |
| Os.52416.1.S1_at | 0.03 | 3.03 | 1.63 | 0.003 |
| Os.25250.1.S1_at | 0.07 | 3.03 | 1.02 | 0.011 |
| Os.27654.1.S1_at | 0.01 | 3.03 | 1.12 | 0.012 |
| Os.47708.1.A1_at | 0.17 | 3.02 | 1.27 | 0.018 |
| Os.8936.1.S1_at | 0.05 | 3.02 | 1.13 | 0.018 |
| OsAffx.25889.1.S1_at | 0.00 | 3.01 | 1.45 | 0.000 |
| Os.17377.1.S1_at | 0.01 | 3.01 | 1.21 | 0.042 |
| Os.4633.1.S1_at | 0.00 | 3.01 | 2.48 | 0.000 |
| Os.7934.1.S1_at | 0.03 | 3.01 | 1.33 | 0.026 |
| OsAffx.13616.1.S1_x_at | 0.03 | 3.01 | 1.12 | 0.014 |
| Os.2293.1.S1_at | 0.00 | 3.01 | 1.30 | 0.004 |
| Os.4177.1.S1_at | 0.00 | 3.00 | 1.65 | 0.016 |
| Os.27861.1.S1_a_at | 0.00 | 3.00 | 1.24 | 0.009 |
| Os.54564.1.S1_at | 0.07 | 3.00 | 1.10 | 0.024 |
| Os.19036.1.S1_at | 0.07 | 3.00 | 1.19 | 0.015 |
| Os.17227.1.S1_at | 0.00 | 3.00 | 1.45 | 0.038 |
| OsAffx.32256.1.A1_x_at | 0.01 | 3.00 | 1.20 | 0.021 |
| Os.20397.1.S1_a_at | 0.00 | 2.99 | 2.21 | 0.018 |
| Os.16692.1.S1_x_at | 0.00 | 2.99 | 1.49 | 0.042 |
| Os.53348.2.S1_at | 0.00 | 2.99 | 2.11 | 0.005 |
| Os.50588.1.S1_at | 0.01 | 2.99 | 1.07 | 0.010 |
| OsAffx.31174.3.S1_x_at | 0.01 | 2.99 | 1.26 | 0.008 |
| Os.10235.1.S1_at | 0.01 | 2.99 | 1.11 | 0.050 |
| OsAffx.7704.1.S1_at | 0.02 | 2.98 | 1.02 | 0.023 |
| Os.5054.1.S1_at | 0.00 | 2.98 | 1.47 | 0.027 |
| OsAffx.32262.1.S1_x_at | 0.00 | 2.98 | 2.07 | 0.009 |
| Os.49838.1.S1_at | 0.00 | 2.98 | 2.45 | 0.030 |
| Os.17652.1.S1_s_at | 0.00 | 2.97 | 1.08 | 0.031 |
| Os.8666.1.S1_at | 0.00 | 2.97 | 1.40 | 0.025 |
| Os.8655.1.S1_at | 0.05 | 2.97 | 1.02 | 0.049 |
| Os.16968.1.S1_at | 0.00 | 2.97 | 1.32 | 0.008 |
| Os.18054.1.S1_s_at | 0.03 | 2.97 | 1.25 | 0.008 |
| Os.24026.1.S1_at | 0.01 | 2.97 | 1.73 | 0.023 |
| Os.11845.1.S1_a_at | 0.01 | 2.97 | 1.07 | 0.000 |
| Os.27175.1.S1_a_at | 0.00 | 2.96 | 1.17 | 0.007 |
| OsAffx.24765.1.S1_at | 0.01 | 2.95 | 1.40 | 0.024 |
| Os.3774.2.S1_x_at | 0.07 | 2.95 | 1.40 | 0.015 |
| OsAffx.31788.1.S1_s_at | 0.00 | 2.95 | 1.42 | 0.016 |
| OsAffx.28980.1.S1_at | 0.00 | 2.94 | 1.26 | 0.022 |
| Os.30608.1.S1_x_at | 0.00 | 2.94 | 5.68 | 0.006 |
| Os.24034.2.S1_x_at | 0.05 | 2.94 | 1.15 | 0.019 |
| Os.14585.1.S1_s_at | 0.01 | 2.94 | 1.36 | 0.000 |
| Os.20084.1.S1_at | 0.00 | 2.94 | 1.32 | 0.000 |
| Os.15022.1.S1_at | 0.00 | 2.94 | 1.32 | 0.007 |
| OsAffx.27460.1.S1_at | 0.02 | 2.94 | 1.05 | 0.011 |
| Os.2244.1.S1_at | 0.00 | 2.94 | 2.19 | 0.002 |
| Os.26818.2.S1_at | 0.07 | 2.93 | 1.25 | 0.006 |
| Os.9768.1.S1_a_at | 0.00 | 2.93 | 1.31 | 0.013 |
| Os.57028.1.S1_at | 0.00 | 2.93 | 1.56 | 0.001 |
| Os.10129.1.S1_at | 0.00 | 2.93 | 1.49 | 0.029 |
| Os.14960.1.S1_at | 0.00 | 2.93 | 1.08 | 0.002 |
| Os.54060.1.S1_at | 0.02 | 2.93 | 1.03 | 0.006 |
| OsAffx.24035.1.S1_s_at | 0.00 | 2.93 | 1.05 | 0.008 |
| OsAffx.32313.1.A1_at | 0.01 | 2.93 | 1.13 | 0.018 |
| Os.49267.1.S1_at | 0.05 | 2.92 | 1.02 | 0.020 |
| Os.11970.1.S1_at | 0.00 | 2.92 | 2.26 | 0.007 |
| OsAffx.30652.1.S1_at | 0.00 | 2.92 | 2.05 | 0.027 |
| Os.25239.1.S1_a_at | 0.00 | 2.92 | 1.24 | 0.004 |
| Os.50298.1.S1_at | 0.00 | 2.91 | 1.27 | 0.014 |
| OsAffx.24761.1.S1_at | 0.00 | 2.91 | 1.05 | 0.013 |
| Os.7676.1.S1_at | 0.00 | 2.91 | 2.67 | 0.025 |
| Os.52130.1.S1_at | 0.00 | 2.91 | 1.38 | 0.007 |
| Os.7933.1.S1_at | 0.00 | 2.90 | 1.50 | 0.002 |
| Os.46208.1.S1_x_at | 0.00 | 2.90 | 1.81 | 0.016 |
| Os.18927.1.S1_at | 0.00 | 2.90 | 1.31 | 0.004 |
| Os.1983.1.S1_at | 0.01 | 2.89 | 1.10 | 0.012 |
| Os.37075.1.S1_at | 0.00 | 2.88 | 1.22 | 0.001 |
| OsAffx.32195.1.A1_at | 0.27 | 2.88 | 1.37 | 0.033 |
| Os.6369.1.S1_at | 0.01 | 2.88 | 1.15 | 0.029 |
| Os.26386.1.S1_a_at | 0.00 | 2.88 | 1.03 | 0.028 |
| Os.7312.1.S1_a_at | 0.00 | 2.87 | 1.27 | 0.016 |
| OsAffx.2611.1.S1_at | 0.01 | 2.87 | 1.27 | 0.032 |
| Os.51206.1.S1_at | 0.00 | 2.87 | 1.08 | 0.010 |
| OsAffx.32206.1.S1_x_at | 0.17 | 2.87 | 1.06 | 0.025 |
| Os.5377.2.S1_x_at | 0.00 | 2.87 | 2.77 | 0.005 |
| Os.9750.1.S1_at | 0.00 | 2.87 | 1.00 | 0.003 |
| Os.7859.1.S1_at | 0.00 | 2.86 | 1.08 | 0.003 |
| Os.47912.1.S1_at | 0.01 | 2.86 | 1.57 | 0.026 |
| Os.16333.1.S1_at | 0.03 | 2.86 | 1.04 | 0.000 |
| OsAffx.26740.1.S1_at | 0.00 | 2.86 | 1.20 | 0.002 |
| Os.52947.1.S1_x_at | 0.00 | 2.86 | 1.18 | 0.008 |
| Os.24428.1.S1_at | 0.01 | 2.85 | 1.03 | 0.003 |
| Os.54411.1.A1_at | 0.00 | 2.85 | 1.25 | 0.006 |
| Os.52226.1.S1_at | 0.00 | 2.85 | 1.86 | 0.017 |
| Os.53148.1.S1_at | 0.00 | 2.85 | 1.13 | 0.002 |
| Os.4603.1.S1_at | 0.02 | 2.85 | 1.01 | 0.035 |
| Os.4948.1.S1_x_at | 0.00 | 2.84 | 1.38 | 0.003 |
| OsAffx.2490.1.S1_at | 0.00 | 2.84 | 1.38 | 0.009 |
| Os.38386.1.A1_at | 0.01 | 2.84 | 1.78 | 0.016 |
| Os.7759.1.S1_at | 0.00 | 2.84 | 1.31 | 0.017 |
| Os.10128.1.S1_at | 0.03 | 2.84 | 1.23 | 0.006 |
| Os.27933.1.S1_at | 0.00 | 2.84 | 1.05 | 0.001 |
| Os.7988.1.S1_s_at | 0.00 | 2.84 | 10.41 | 0.006 |
| Os.10370.2.S1_x_at | 0.00 | 2.84 | 11.01 | 0.004 |
| Os.15007.1.S1_at | 0.02 | 2.84 | 1.40 | 0.047 |
| Os.5170.1.S1_at | 0.01 | 2.83 | 1.09 | 0.042 |
| Os.10872.1.S1_at | 0.07 | 2.83 | 1.33 | 0.011 |
| Os.46728.1.S1_at | 0.00 | 2.83 | 12.41 | 0.045 |
| Os.15247.1.S1_s_at | 0.03 | 2.82 | 1.19 | 0.042 |
| Os.35065.1.S1_at | 0.01 | 2.82 | 1.41 | 0.005 |
| OsAffx.24834.1.S1_at | 0.01 | 2.82 | 1.28 | 0.005 |
| Os.51057.1.S1_s_at | 0.00 | 2.82 | 1.15 | 0.014 |
| Os.11184.1.S1_at | 0.00 | 2.82 | 1.33 | 0.024 |
| Os.22374.1.S2_a_at | 0.01 | 2.82 | 1.05 | 0.010 |
| OsAffx.32323.1.A1_at | 0.03 | 2.82 | 1.07 | 0.044 |
| Os.10144.1.S1_at | 0.00 | 2.82 | 1.42 | 0.000 |
| OsAffx.6135.1.S1_s_at | 0.17 | 2.81 | 1.20 | 0.031 |
| Os.14216.1.S1_at | 0.07 | 2.81 | 1.31 | 0.025 |
| OsAffx.24153.1.S1_s_at | 0.00 | 2.81 | 1.00 | 0.019 |
| Os.48834.1.S1_at | 0.00 | 2.81 | 1.09 | 0.001 |
| Os.28394.1.S1_s_at | 0.01 | 2.81 | 1.41 | 0.018 |
| Os.54437.1.S1_at | 0.07 | 2.81 | 1.28 | 0.013 |
| Os.27670.1.S1_at | 0.00 | 2.81 | 1.20 | 0.007 |
| Os.26783.1.S1_at | 1.46 | 2.81 | 1.14 | 0.005 |
| Os.7747.1.S1_at | 0.01 | 2.81 | 1.04 | 0.011 |
| Os.37762.1.S1_a_at | 0.07 | 2.80 | 1.14 | 0.012 |
| Os.8549.1.S1_at | 0.07 | 2.80 | 1.04 | 0.000 |
| OsAffx.12252.1.S1_at | 0.00 | 2.80 | 1.92 | 0.022 |
| Os.50806.1.A1_at | 0.07 | 2.80 | 1.49 | 0.002 |
| OsAffx.14480.1.S1_at | 0.00 | 2.80 | 2.68 | 0.029 |
| Os.20473.1.S1_s_at | 0.00 | 2.80 | 3.68 | 0.033 |
| Os.2330.1.S1_at | 0.00 | 2.79 | 1.25 | 0.001 |
| OsAffx.16875.1.S1_x_at | 0.11 | 2.79 | 1.15 | 0.020 |
| Os.17741.1.S1_at | 0.02 | 2.79 | 1.49 | 0.024 |
| Os.51086.1.S1_x_at | 0.00 | 2.79 | 4.16 | 0.001 |
| OsAffx.2808.1.S1_s_at | 0.00 | 2.79 | 1.72 | 0.032 |
| Os.14831.1.S1_at | 0.00 | 2.79 | 1.25 | 0.005 |
| Os.16806.1.S1_at | 0.03 | 2.79 | 1.01 | 0.026 |
| Os.5567.1.S1_at | 0.00 | 2.79 | 1.13 | 0.035 |
| Os.10352.1.S1_at | 0.01 | 2.78 | 1.35 | 0.029 |
| Os.27942.1.A1_at | 0.00 | 2.78 | 1.43 | 0.039 |
| Os.26758.1.A1_a_at | 0.02 | 2.78 | 1.06 | 0.044 |
| Os.49795.1.S1_at | 0.00 | 2.78 | 1.79 | 0.029 |
| Os.11664.1.S1_at | 0.00 | 2.77 | 2.35 | 0.009 |
| Os.9230.1.S1_x_at | 0.00 | 2.77 | 2.57 | 0.013 |
| Os.52400.1.S1_at | 0.00 | 2.77 | 1.37 | 0.047 |
| Os.49670.1.S1_at | 0.00 | 2.77 | 3.38 | 0.011 |
| Os.49486.1.S1_at | 0.01 | 2.77 | 1.13 | 0.014 |
| Os.19310.1.S1_at | 0.03 | 2.76 | 1.05 | 0.015 |
| Os.48148.1.S1_at | 0.00 | 2.76 | 1.76 | 0.035 |
| OsAffx.2572.1.S1_at | 0.00 | 2.76 | 1.31 | 0.019 |
| Os.20603.1.S1_at | 0.07 | 2.76 | 1.24 | 0.005 |
| Os.26967.1.S2_at | 0.01 | 2.76 | 1.14 | 0.026 |
| Os.20810.4.S1_at | 0.01 | 2.76 | 1.02 | 0.008 |
| Os.16429.1.S1_at | 0.01 | 2.75 | 1.07 | 0.020 |
| Os.46525.2.S1_x_at | 0.01 | 2.75 | 1.65 | 0.001 |
| Os.32618.1.S1_at | 0.00 | 2.75 | 5.78 | 0.017 |
| Os.16119.1.S1_at | 0.01 | 2.75 | 1.03 | 0.029 |
| Os.39063.1.A1_s_at | 0.01 | 2.74 | 1.01 | 0.011 |
| Os.48911.1.A1_s_at | 0.01 | 2.74 | 1.40 | 0.025 |
| Os.47908.1.S1_at | 0.11 | 2.74 | 1.29 | 0.002 |
| OsAffx.15190.1.S1_at | 0.11 | 2.74 | 1.36 | 0.043 |
| Os.11673.2.S1_x_at | 0.00 | 2.74 | 1.25 | 0.002 |
| Os.15041.1.S1_a_at | 0.00 | 2.74 | 1.74 | 0.024 |
| Os.14820.1.S2_s_at | 0.03 | 2.74 | 1.38 | 0.049 |
| Os.26588.1.S1_x_at | 0.11 | 2.74 | 1.05 | 0.003 |
| Os.12261.1.S1_a_at | 0.00 | 2.74 | 1.03 | 0.003 |
| Os.36529.1.S1_at | 0.07 | 2.73 | 1.04 | 0.008 |
| Os.7754.1.S1_at | 0.00 | 2.73 | 1.20 | 0.013 |
| Os.27933.2.S1_s_at | 0.00 | 2.73 | 1.02 | 0.007 |
| Os.20603.1.S1_s_at | 0.07 | 2.73 | 1.03 | 0.040 |
| Os.20075.1.S1_at | 0.03 | 2.73 | 1.13 | 0.023 |
| OsAffx.32208.1.S1_x_at | 0.00 | 2.73 | 2.50 | 0.019 |
| Os.2362.2.S1_x_at | 0.11 | 2.72 | 1.36 | 0.013 |
| Os.9779.1.S1_at | 0.01 | 2.72 | 1.16 | 0.003 |
| Os.19007.1.S1_at | 0.00 | 2.72 | 1.04 | 0.021 |
| Os.12312.1.S1_at | 0.41 | 2.72 | 1.11 | 0.017 |
| Os.22638.1.S1_x_at | 0.00 | 2.72 | 1.08 | 0.001 |
| OsAffx.26669.1.S1_at | 0.11 | 2.71 | 1.27 | 0.012 |
| OsAffx.3106.1.S1_x_at | 0.00 | 2.71 | 1.43 | 0.029 |
| Os.51361.1.S1_at | 0.00 | 2.71 | 1.12 | 0.000 |
| Os.27635.3.A1_x_at | 0.07 | 2.71 | 1.36 | 0.034 |
| Os.8014.2.S1_x_at | 0.00 | 2.71 | 7.47 | 0.027 |
| Os.12141.1.S1_at | 0.00 | 2.71 | 1.57 | 0.007 |
| Os.47971.1.A1_at | 0.00 | 2.71 | 1.60 | 0.013 |
| Os.11994.1.S1_at | 0.17 | 2.71 | 1.09 | 0.030 |
| Os.13844.1.S1_at | 0.00 | 2.70 | 6.04 | 0.048 |
| Os.8987.1.S1_at | 0.00 | 2.70 | 1.44 | 0.031 |
| Os.20757.1.S1_a_at | 0.07 | 2.70 | 1.34 | 0.047 |
| Os.27821.1.S1_s_at | 0.00 | 2.70 | 1.04 | 0.019 |
| Os.9536.1.S1_at | 0.00 | 2.70 | 1.02 | 0.036 |
| Os.3739.1.S1_at | 0.02 | 2.70 | 1.14 | 0.014 |
| Os.37603.1.S1_at | 0.03 | 2.70 | 1.04 | 0.001 |
| Os.27717.1.S1_a_at | 0.00 | 2.69 | 1.70 | 0.048 |
| Os.56238.1.S1_at | 0.00 | 2.69 | 1.48 | 0.005 |
| Os.19308.1.S1_x_at | 0.17 | 2.69 | 1.18 | 0.036 |
| Os.14750.1.S1_x_at | 0.07 | 2.69 | 1.12 | 0.007 |
| Os.54064.1.S1_at | 0.00 | 2.69 | 1.28 | 0.027 |
| Os.11573.7.A1_x_at | 0.00 | 2.69 | 9.37 | 0.001 |
| OsAffx.14067.1.S1_at | 0.03 | 2.69 | 1.48 | 0.015 |
| Os.57565.1.S1_at | 0.02 | 2.68 | 1.49 | 0.001 |
| Os.28255.1.S1_at | 0.00 | 2.68 | 1.52 | 0.002 |
| Os.51989.1.S2_at | 0.01 | 2.68 | 1.12 | 0.021 |
| Os.26359.3.S1_at | 0.07 | 2.68 | 1.33 | 0.001 |
| Os.48074.1.A1_at | 0.00 | 2.68 | 2.62 | 0.040 |
| Os.1191.1.S1_at | 0.01 | 2.68 | 1.65 | 0.026 |
| Os.52297.1.S1_at | 0.01 | 2.68 | 1.10 | 0.000 |
| Os.5577.2.S1_at | 0.00 | 2.68 | 1.23 | 0.009 |
| Os.27519.1.S1_at | 0.17 | 2.68 | 1.14 | 0.010 |
| Os.20829.1.S1_at | 0.01 | 2.68 | 1.10 | 0.038 |
| Os.9448.1.A2_at | 0.07 | 2.67 | 1.01 | 0.007 |
| Os.27426.1.S1_at | 0.98 | 2.67 | 1.11 | 0.000 |
| Os.9563.1.S1_at | 0.00 | 2.67 | 2.60 | 0.038 |
| Os.12622.1.S1_at | 0.00 | 2.67 | 1.66 | 0.008 |
| Os.15707.1.S1_at | 0.01 | 2.67 | 1.25 | 0.012 |
| OsAffx.6438.1.S1_at | 0.00 | 2.66 | 1.86 | 0.004 |
| Os.13014.1.S1_a_at | 0.00 | 2.66 | 1.30 | 0.030 |
| Os.27314.1.S1_at | 0.02 | 2.66 | 1.16 | 0.007 |
| OsAffx.26436.1.S1_at | 0.01 | 2.66 | 1.10 | 0.003 |
| OsAffx.25555.1.S1_s_at | 0.00 | 2.66 | 4.16 | 0.005 |
| Os.33271.1.S1_a_at | 0.00 | 2.66 | 1.29 | 0.007 |
| Os.17547.1.S1_at | 0.00 | 2.66 | 1.13 | 0.047 |
| Os.53196.1.S1_at | 0.00 | 2.65 | 1.12 | 0.001 |
| Os.27502.1.S1_at | 0.07 | 2.65 | 1.19 | 0.030 |
| Os.17149.1.S1_at | 0.64 | 2.65 | 1.22 | 0.035 |
| Os.3838.1.S1_at | 0.00 | 2.64 | 1.74 | 0.010 |
| Os.26990.1.S1_at | 0.00 | 2.64 | 3.03 | 0.041 |
| Os.46403.1.S1_at | 0.02 | 2.64 | 1.15 | 0.011 |
| Os.21061.1.S1_at | 0.07 | 2.64 | 1.05 | 0.027 |
| Os.5697.1.S1_at | 0.01 | 2.64 | 1.12 | 0.031 |
| Os.27508.1.S2_a_at | 0.00 | 2.64 | 1.08 | 0.015 |
| OsAffx.24834.1.S1_x_at | 0.00 | 2.63 | 1.33 | 0.003 |
| Os.21038.1.S1_s_at | 0.05 | 2.63 | 1.35 | 0.021 |
| OsAffx.16965.1.S1_x_at | 0.00 | 2.63 | 1.06 | 0.003 |
| Os.53291.1.S1_at | 0.01 | 2.62 | 1.01 | 0.003 |
| Os.8938.1.S1_at | 0.00 | 2.62 | 2.14 | 0.008 |
| Os.14762.2.S1_s_at | 0.17 | 2.62 | 1.51 | 0.014 |
| Os.5206.1.S1_at | 0.00 | 2.62 | 1.83 | 0.008 |
| Os.21364.2.S1_x_at | 0.00 | 2.62 | 1.05 | 0.017 |
| Os.36418.1.S1_at | 0.17 | 2.62 | 1.13 | 0.038 |
| Os.46774.1.S1_at | 0.05 | 2.62 | 1.06 | 0.001 |
| Os.25324.1.A1_at | 0.00 | 2.61 | 1.22 | 0.036 |
| OsAffx.32314.1.A1_at | 0.01 | 2.61 | 1.12 | 0.022 |
| Os.10595.1.S1_at | 0.00 | 2.61 | 1.05 | 0.005 |
| Os.8865.2.S1_x_at | 0.05 | 2.61 | 1.35 | 0.005 |
| Os.8444.1.S1_s_at | 0.17 | 2.61 | 1.14 | 0.038 |
| OsAffx.11002.1.S1_at | 0.00 | 2.61 | 1.38 | 0.033 |
| Os.14915.1.S1_a_at | 0.00 | 2.61 | 1.61 | 0.012 |
| Os.6371.1.S1_at | 0.00 | 2.61 | 1.27 | 0.002 |
| Os.14176.1.S1_at | 0.00 | 2.61 | 1.02 | 0.002 |
| Os.9620.1.S1_at | 0.00 | 2.60 | 1.10 | 0.001 |
| OsAffx.32336.1.A1_at | 0.03 | 2.60 | 1.27 | 0.034 |
| Os.23792.2.S1_x_at | 0.00 | 2.60 | 1.50 | 0.011 |
| OsAffx.25078.1.S1_s_at | 0.27 | 2.60 | 1.25 | 0.020 |
| Os.49184.1.S1_at | 0.01 | 2.60 | 1.32 | 0.004 |
| Os.52809.1.S1_at | 0.00 | 2.60 | 1.07 | 0.039 |
| Os.50483.1.S1_at | 0.00 | 2.60 | 3.01 | 0.016 |
| Os.11399.1.S1_at | 0.00 | 2.60 | 1.13 | 0.004 |
| Os.48352.1.A1_at | 0.00 | 2.59 | 1.53 | 0.004 |
| OsAffx.16693.1.S1_x_at | 0.17 | 2.59 | 1.09 | 0.016 |
| Os.52470.1.S1_at | 0.03 | 2.59 | 1.17 | 0.019 |
| Os.38045.1.S1_at | 0.05 | 2.58 | 1.42 | 0.015 |
| Os.33786.1.S1_at | 0.01 | 2.58 | 1.46 | 0.034 |
| Os.49216.1.S1_at | 0.17 | 2.58 | 1.02 | 0.030 |
| Os.51212.1.S1_at | 0.00 | 2.58 | 1.12 | 0.009 |
| Os.14992.1.S1_x_at | 0.00 | 2.58 | 1.23 | 0.000 |
| Os.17876.1.S1_at | 0.05 | 2.58 | 1.09 | 0.010 |
| Os.4329.1.S1_at | 0.00 | 2.57 | 3.45 | 0.019 |
| Os.17149.2.S1_x_at | 0.41 | 2.57 | 1.02 | 0.016 |
| Os.12611.1.S1_at | 0.00 | 2.57 | 1.07 | 0.002 |
| Os.27433.1.S1_at | 0.03 | 2.57 | 1.57 | 0.007 |
| Os.54427.1.A1_at | 0.00 | 2.57 | 2.28 | 0.029 |
| OsAffx.7704.1.S1_s_at | 0.01 | 2.57 | 1.09 | 0.015 |
| Os.15138.1.S1_at | 0.02 | 2.56 | 1.25 | 0.020 |
| Os.11874.1.S1_s_at | 0.00 | 2.56 | 1.01 | 0.009 |
| Os.27646.1.A1_at | 0.01 | 2.56 | 1.07 | 0.027 |
| Os.50876.1.S1_at | 0.00 | 2.56 | 2.34 | 0.005 |
| Os.17419.1.S1_at | 0.07 | 2.56 | 1.76 | 0.040 |
| OsAffx.26462.1.S1_at | 0.01 | 2.56 | 1.05 | 0.009 |
| Os.7860.1.S1_at | 0.00 | 2.56 | 1.41 | 0.010 |
| Os.11972.1.S1_s_at | 0.41 | 2.56 | 1.09 | 0.018 |
| Os.10227.1.S1_at | 0.11 | 2.56 | 1.04 | 0.039 |
| Os.16854.1.S1_at | 0.00 | 2.56 | 1.34 | 0.025 |
| Os.20204.2.S1_a_at | 0.00 | 2.56 | 1.59 | 0.003 |
| Os.11117.1.S2_at | 0.02 | 2.56 | 1.02 | 0.010 |
| Os.33271.2.S1_x_at | 0.05 | 2.55 | 1.05 | 0.021 |
| Os.8970.1.S1_at | 0.00 | 2.55 | 1.14 | 0.002 |
| Os.9247.2.S1_x_at | 0.07 | 2.55 | 1.13 | 0.046 |
| Os.17198.1.S1_at | 0.00 | 2.54 | 1.11 | 0.029 |
| Os.9880.1.S1_a_at | 0.00 | 2.54 | 2.86 | 0.015 |
| Os.21849.1.S1_at | 0.01 | 2.54 | 1.08 | 0.014 |
| Os.5364.1.S1_at | 0.07 | 2.54 | 1.01 | 0.031 |
| Os.5424.1.S1_at | 0.02 | 2.54 | 1.33 | 0.012 |
| Os.52523.1.S1_at | 0.01 | 2.54 | 1.30 | 0.030 |
| OsAffx.19268.1.S1_at | 0.01 | 2.54 | 1.01 | 0.004 |
| Os.7230.1.S1_at | 0.00 | 2.54 | 1.38 | 0.001 |
| Os.8508.1.S1_at | 0.00 | 2.53 | 2.17 | 0.014 |
| Os.9134.1.S1_at | 0.00 | 2.53 | 1.30 | 0.002 |
| OsAffx.23740.1.S1_at | 0.05 | 2.53 | 1.01 | 0.015 |
| Os.52467.1.S1_at | 0.05 | 2.53 | 1.25 | 0.028 |
| Os.3167.1.S1_at | 0.00 | 2.53 | 1.85 | 0.002 |
| Os.1665.1.S1_a_at | 0.00 | 2.52 | 1.02 | 0.010 |
| Os.53782.1.S1_at | 0.01 | 2.52 | 1.03 | 0.036 |
| Os.51258.1.S1_at | 0.00 | 2.52 | 1.27 | 0.016 |
| Os.10271.2.S1_x_at | 0.00 | 2.52 | 1.60 | 0.002 |
| Os.57294.1.S1_at | 0.03 | 2.52 | 1.06 | 0.006 |
| Os.16765.1.S1_at | 0.00 | 2.52 | 1.84 | 0.050 |
| Os.8758.1.S1_s_at | 0.02 | 2.51 | 1.01 | 0.009 |
| OsAffx.18947.1.S1_x_at | 0.00 | 2.51 | 10.39 | 0.000 |
| Os.18552.1.S1_at | 0.00 | 2.51 | 4.37 | 0.003 |
| Os.51807.1.S1_at | 0.98 | 2.51 | 1.01 | 0.037 |
| Os.54942.1.S1_at | 0.11 | 2.50 | 1.04 | 0.042 |
| Os.5992.1.S1_at | 0.00 | 2.50 | 1.04 | 0.016 |
| Os.11724.2.S2_s_at | 0.00 | 2.50 | 1.87 | 0.013 |
| Os.28133.1.S1_s_at | 0.00 | 2.50 | 16.16 | 0.003 |
| Os.46062.1.S1_at | 0.01 | 2.50 | 1.21 | 0.039 |
| Os.5619.1.A1_at | 0.00 | 2.50 | 1.65 | 0.017 |
| Os.12056.1.S1_at | 0.00 | 2.50 | 7.16 | 0.009 |
| Os.15238.1.S1_at | 0.01 | 2.50 | 1.19 | 0.027 |
| Os.13937.1.S1_at | 0.00 | 2.50 | 1.01 | 0.023 |
| Os.38256.1.S1_at | 0.64 | 2.49 | 1.01 | 0.041 |
| OsAffx.32220.1.S1_s_at | 0.00 | 2.49 | 1.07 | 0.015 |
| Os.26733.3.S1_x_at | 0.27 | 2.49 | 1.15 | 0.011 |
| Os.17783.1.S1_at | 0.01 | 2.49 | 1.44 | 0.034 |
| Os.17640.1.S1_at | 0.02 | 2.49 | 1.09 | 0.005 |
| Os.16918.1.S1_at | 0.01 | 2.49 | 1.02 | 0.022 |
| Os.17074.1.S1_at | 0.98 | 2.49 | 1.29 | 0.042 |
| Os.30724.1.S1_at | 0.02 | 2.49 | 1.18 | 0.045 |
| Os.47951.1.S1_at | 0.00 | 2.49 | 1.35 | 0.039 |
| Os.8406.1.S1_at | 0.00 | 2.48 | 1.04 | 0.002 |
| OsAffx.23944.2.S1_at | 0.00 | 2.48 | 1.61 | 0.028 |
| Os.18663.1.S1_at | 0.03 | 2.48 | 1.02 | 0.023 |
| Os.6831.1.S1_a_at | 0.02 | 2.48 | 1.10 | 0.036 |
| Os.46070.1.S1_x_at | 0.01 | 2.47 | 1.11 | 0.011 |
| Os.11787.1.S1_at | 0.00 | 2.47 | 1.67 | 0.015 |
| Os.28438.4.S1_s_at | 0.00 | 2.47 | 4.75 | 0.003 |
| Os.14762.3.S1_x_at | 0.02 | 2.47 | 1.69 | 0.005 |
| Os.17898.1.S1_at | 0.00 | 2.47 | 1.25 | 0.041 |
| Os.15117.1.S1_at | 0.02 | 2.47 | 1.47 | 0.021 |
| Os.10120.2.S1_at | 0.00 | 2.47 | 1.03 | 0.008 |
| Os.15295.3.S1_x_at | 0.64 | 2.46 | 1.01 | 0.017 |
| Os.5571.1.S1_at | 0.17 | 2.46 | 1.14 | 0.027 |
| Os.10132.1.S1_at | 0.01 | 2.46 | 1.11 | 0.002 |
| Os.53014.1.S1_at | 0.00 | 2.46 | 1.11 | 0.035 |
| Os.28438.2.S1_a_at | 0.00 | 2.46 | 3.57 | 0.002 |
| Os.14874.1.S1_at | 0.01 | 2.46 | 1.03 | 0.005 |
| OsAffx.32337.1.S1_x_at | 0.03 | 2.46 | 1.11 | 0.043 |
| Os.48332.1.A1_s_at | 0.00 | 2.46 | 1.29 | 0.016 |
| Os.26548.1.S1_at | 0.03 | 2.45 | 1.70 | 0.045 |
| Os.33127.2.S1_x_at | 0.00 | 2.45 | 1.14 | 0.049 |
| Os.18825.1.S1_at | 0.01 | 2.45 | 1.01 | 0.024 |
| Os.6566.1.S1_at | 0.00 | 2.45 | 1.56 | 0.026 |
| Os.12607.1.S1_s_at | 0.00 | 2.44 | 1.17 | 0.008 |
| OsAffx.12830.1.S1_at | 0.27 | 2.44 | 1.04 | 0.035 |
| Os.23028.1.S1_s_at | 0.00 | 2.44 | 1.66 | 0.040 |
| Os.5439.1.S1_a_at | 0.00 | 2.44 | 1.03 | 0.008 |
| Os.47600.1.S1_s_at | 0.00 | 2.43 | 1.31 | 0.010 |
| Os.46208.1.S1_s_at | 0.00 | 2.43 | 1.94 | 0.024 |
| Os.53012.1.S1_at | 0.00 | 2.43 | 1.10 | 0.007 |
| Os.5307.1.S1_s_at | 0.00 | 2.43 | 1.23 | 0.004 |
| Os.37762.1.S1_at | 0.07 | 2.42 | 1.24 | 0.002 |
| Os.10136.1.S1_at | 0.00 | 2.42 | 7.60 | 0.001 |
| Os.5961.1.S1_at | 0.07 | 2.42 | 1.06 | 0.009 |
| Os.34151.1.S1_a_at | 0.00 | 2.42 | 1.42 | 0.001 |
| Os.57006.1.S1_at | 0.00 | 2.42 | 2.83 | 0.027 |
| OsAffx.28136.1.S1_at | 0.05 | 2.42 | 1.04 | 0.013 |
| OsAffx.25184.1.S1_at | 0.03 | 2.41 | 1.11 | 0.025 |
| OsAffx.15782.1.S1_at | 0.00 | 2.41 | 1.08 | 0.000 |
| OsAffx.11154.2.S1_s_at | 0.02 | 2.41 | 1.05 | 0.044 |
| Os.10447.1.S1_at | 0.01 | 2.40 | 1.34 | 0.039 |
| Os.54620.1.A1_at | 0.00 | 2.40 | 10.33 | 0.024 |
| Os.16894.1.S1_at | 0.03 | 2.40 | 1.03 | 0.042 |
| Os.18863.1.S1_at | 0.03 | 2.40 | 1.08 | 0.014 |
| Os.49711.1.S1_at | 0.27 | 2.40 | 1.20 | 0.040 |
| Os.53716.1.S1_at | 0.00 | 2.40 | 2.02 | 0.042 |
| Os.15134.1.S1_at | 0.00 | 2.40 | 1.02 | 0.015 |
| Os.8865.4.S1_x_at | 0.05 | 2.39 | 1.31 | 0.007 |
| Os.3395.1.S1_at | 0.00 | 2.39 | 1.34 | 0.043 |
| Os.35543.1.S1_at | 0.00 | 2.39 | 1.26 | 0.016 |
| Os.19325.1.S1_at | 0.00 | 2.39 | 1.22 | 0.004 |
| OsAffx.27388.1.S1_at | 0.01 | 2.39 | 1.14 | 0.002 |
| Os.36108.2.S1_x_at | 0.03 | 2.39 | 1.01 | 0.024 |
| OsAffx.6114.1.S1_at | 0.01 | 2.39 | 1.04 | 0.001 |
| Os.33604.1.S1_a_at | 0.00 | 2.39 | 1.26 | 0.045 |
| Os.39257.1.S1_at | 0.00 | 2.38 | 1.25 | 0.023 |
| OsAffx.32196.1.A1_at | 0.07 | 2.38 | 1.07 | 0.042 |
| Os.9118.1.S1_at | 0.00 | 2.38 | 1.26 | 0.018 |
| Os.10776.1.S1_at | 0.01 | 2.37 | 1.12 | 0.008 |
| Os.11629.1.S1_at | 0.11 | 2.37 | 1.55 | 0.043 |
| Os.28218.1.S1_x_at | 0.00 | 2.37 | 17.37 | 0.028 |
| Os.8858.1.S1_a_at | 0.01 | 2.37 | 1.24 | 0.010 |
| OsAffx.32264.1.S1_x_at | 0.00 | 2.37 | 2.29 | 0.023 |
| Os.48062.1.S1_s_at | 0.00 | 2.37 | 1.05 | 0.009 |
| Os.28032.1.A1_at | 0.00 | 2.37 | 8.50 | 0.034 |
| Os.46988.1.A1_at | 0.00 | 2.37 | 3.49 | 0.029 |
| Os.11230.1.S1_s_at | 0.00 | 2.37 | 1.34 | 0.016 |
| Os.44471.2.S1_at | 0.05 | 2.37 | 1.01 | 0.011 |
| Os.14872.1.S1_at | 0.00 | 2.36 | 1.19 | 0.001 |
| Os.20693.1.S1_at | 0.64 | 2.36 | 1.07 | 0.033 |
| Os.6336.1.S1_at | 0.00 | 2.36 | 1.92 | 0.034 |
| OsAffx.21812.1.S1_at | 0.11 | 2.36 | 1.17 | 0.039 |
| OsAffx.24672.1.S1_at | 0.00 | 2.36 | 1.16 | 0.013 |
| Os.30608.1.S1_at | 0.00 | 2.36 | 6.01 | 0.005 |
| Os.22483.1.A1_s_at | 0.00 | 2.36 | 1.04 | 0.034 |
| Os.12277.1.S1_at | 0.17 | 2.36 | 1.06 | 0.009 |
| Os.17210.1.S1_at | 0.17 | 2.35 | 1.01 | 0.001 |
| Os.2248.1.S1_at | 0.00 | 2.35 | 3.38 | 0.022 |
| Os.10001.1.S1_at | 0.00 | 2.35 | 1.38 | 0.041 |
| Os.10609.1.S1_at | 0.00 | 2.35 | 1.26 | 0.002 |
| OsAffx.31358.1.S1_at | 0.00 | 2.35 | 1.23 | 0.009 |
| Os.3774.1.S1_at | 0.02 | 2.35 | 1.60 | 0.023 |
| Os.8520.1.S1_at | 0.00 | 2.35 | 1.52 | 0.013 |
| Os.9650.1.A1_at | 0.00 | 2.35 | 1.20 | 0.033 |
| Os.15189.1.S1_at | 0.00 | 2.35 | 1.04 | 0.002 |
| Os.7873.1.S1_x_at | 0.00 | 2.34 | 1.44 | 0.038 |
| OsAffx.29152.1.S1_s_at | 0.02 | 2.34 | 1.26 | 0.010 |
| Os.7971.2.S1_s_at | 0.00 | 2.34 | 1.62 | 0.007 |
| Os.4661.1.S1_at | 0.01 | 2.34 | 1.23 | 0.002 |
| Os.22263.1.S1_a_at | 0.01 | 2.34 | 1.12 | 0.011 |
| Os.53308.1.A1_at | 0.00 | 2.34 | 1.16 | 0.001 |
| Os.5874.1.S1_at | 0.00 | 2.34 | 1.03 | 0.041 |
| OsAffx.20119.1.S1_at | 0.07 | 2.34 | 1.29 | 0.020 |
| Os.17440.1.S1_at | 0.05 | 2.34 | 1.20 | 0.035 |
| OsAffx.30042.1.S1_at | 0.03 | 2.34 | 1.22 | 0.041 |
| Os.23802.1.A1_at | 0.00 | 2.34 | 1.09 | 0.026 |
| Os.49212.1.S1_a_at | 0.11 | 2.34 | 1.01 | 0.005 |
| Os.8907.1.S1_at | 0.00 | 2.33 | 1.09 | 0.020 |
| Os.16261.1.S1_at | 0.05 | 2.33 | 1.00 | 0.017 |
| Os.27516.1.A1_at | 0.00 | 2.33 | 1.12 | 0.010 |
| Os.19622.1.S1_at | 0.07 | 2.33 | 1.15 | 0.011 |
| Os.5631.1.S1_s_at | 0.01 | 2.33 | 1.08 | 0.000 |
| Os.11014.1.S1_at | 0.00 | 2.33 | 1.24 | 0.015 |
| Os.24869.1.A1_at | 0.00 | 2.33 | 1.33 | 0.022 |
| Os.15729.1.S1_at | 0.11 | 2.33 | 1.47 | 0.025 |
| Os.19268.1.S1_at | 2.14 | 2.32 | 1.09 | 0.049 |
| Os.45486.1.S1_x_at | 0.00 | 2.32 | 2.05 | 0.021 |
| Os.9619.1.S1_at | 0.00 | 2.32 | 1.40 | 0.023 |
| OsAffx.31341.1.S1_s_at | 0.00 | 2.32 | 1.41 | 0.007 |
| OsAffx.24694.1.S1_x_at | 0.01 | 2.32 | 1.09 | 0.038 |
| Os.11693.1.S1_at | 0.07 | 2.32 | 1.88 | 0.018 |
| Os.11619.1.A1_at | 0.64 | 2.31 | 1.44 | 0.030 |
| OsAffx.14819.1.S1_at | 0.11 | 2.31 | 1.55 | 0.024 |
| Os.47784.1.S1_at | 0.00 | 2.31 | 1.28 | 0.010 |
| Os.10078.1.S1_at | 0.27 | 2.31 | 1.14 | 0.043 |
| Os.4642.1.A1_s_at | 0.11 | 2.31 | 1.10 | 0.001 |
| Os.27275.1.S1_at | 0.17 | 2.31 | 1.03 | 0.039 |
| Os.14148.1.S1_at | 0.00 | 2.31 | 1.12 | 0.010 |
| Os.9116.1.S1_at | 0.00 | 2.30 | 1.98 | 0.029 |
| Os.15991.1.S1_at | 0.00 | 2.30 | 1.51 | 0.026 |
| OsAffx.11050.1.S1_x_at | 0.00 | 2.30 | 1.44 | 0.026 |
| Os.26509.1.S1_x_at | 0.00 | 2.30 | 1.45 | 0.042 |
| Os.26522.1.S1_at | 0.00 | 2.30 | 2.06 | 0.044 |
| OsAffx.22431.1.S1_s_at | 0.01 | 2.30 | 1.17 | 0.001 |
| Os.7011.1.S1_at | 0.00 | 2.30 | 1.49 | 0.016 |
| Os.14762.2.S1_x_at | 0.11 | 2.30 | 1.35 | 0.020 |
| Os.47372.1.S1_x_at | 0.00 | 2.30 | 1.90 | 0.044 |
| Os.18459.2.S1_x_at | 0.00 | 2.30 | 5.99 | 0.003 |
| Os.25449.1.S1_at | 0.01 | 2.29 | 1.00 | 0.004 |
| Os.9354.1.S1_at | 0.00 | 2.29 | 1.17 | 0.004 |
| Os.27826.1.S1_at | 0.00 | 2.29 | 1.07 | 0.011 |
| Os.12376.1.S1_at | 0.00 | 2.29 | 1.39 | 0.034 |
| Os.7431.1.S1_a_at | 1.46 | 2.29 | 1.08 | 0.035 |
| Os.39989.1.S1_s_at | 0.03 | 2.29 | 1.87 | 0.039 |
| Os.23932.1.A1_at | 0.02 | 2.29 | 1.20 | 0.044 |
| Os.52545.1.S1_at | 0.01 | 2.29 | 1.19 | 0.015 |
| Os.11999.1.S1_at | 0.01 | 2.28 | 1.29 | 0.021 |
| OsAffx.25620.1.S1_x_at | 0.03 | 2.28 | 1.37 | 0.050 |
| Os.11222.2.S1_x_at | 0.00 | 2.28 | 1.19 | 0.004 |
| Os.14956.1.S1_at | 0.05 | 2.28 | 1.07 | 0.030 |
| Os.13253.1.S1_at | 0.11 | 2.28 | 1.39 | 0.047 |
| Os.24631.1.A1_at | 0.00 | 2.28 | 1.34 | 0.017 |
| Os.19563.1.S1_at | 0.02 | 2.28 | 1.04 | 0.001 |
| Os.20835.1.S1_at | 0.00 | 2.28 | 1.16 | 0.044 |
| Os.10275.1.S1_at | 0.00 | 2.27 | 2.15 | 0.002 |
| Os.8203.1.S1_at | 0.01 | 2.27 | 1.94 | 0.004 |
| OsAffx.17468.1.S1_s_at | 0.00 | 2.27 | 4.94 | 0.028 |
| Os.54773.1.S1_at | 0.00 | 2.27 | 1.90 | 0.027 |
| Os.16757.1.S1_s_at | 0.07 | 2.27 | 1.25 | 0.011 |
| Os.38791.1.A1_s_at | 0.03 | 2.27 | 1.19 | 0.019 |
| OsAffx.30737.1.S1_at | 0.00 | 2.26 | 1.12 | 0.029 |
| Os.49099.1.S1_at | 0.00 | 2.26 | 1.05 | 0.033 |
| Os.7873.1.S1_at | 0.00 | 2.26 | 1.37 | 0.043 |
| Os.46498.1.S1_at | 0.00 | 2.26 | 1.27 | 0.033 |
| Os.9773.1.S1_at | 0.00 | 2.26 | 1.80 | 0.003 |
| Os.10314.1.S1_a_at | 0.17 | 2.25 | 1.08 | 0.027 |
| Os.52420.1.A1_at | 0.00 | 2.25 | 1.52 | 0.016 |
| Os.52426.1.S1_at | 0.01 | 2.25 | 1.07 | 0.012 |
| Os.834.1.S1_a_at | 0.00 | 2.25 | 1.96 | 0.026 |
| Os.48588.1.A1_at | 0.00 | 2.25 | 1.11 | 0.001 |
| Os.27779.1.S1_at | 0.01 | 2.25 | 1.15 | 0.041 |
| OsAffx.4181.1.S1_at | 0.00 | 2.25 | 2.62 | 0.008 |
| Os.17540.1.S1_at | 0.01 | 2.25 | 1.09 | 0.036 |
| OsAffx.32335.1.A1_at | 0.03 | 2.25 | 1.38 | 0.049 |
| Os.29958.2.S1_x_at | 0.00 | 2.25 | 1.47 | 0.016 |
| Os.27354.1.S1_at | 0.00 | 2.25 | 1.79 | 0.020 |
| Os.37384.1.S1_x_at | 0.07 | 2.25 | 1.11 | 0.015 |
| Os.21805.1.S1_s_at | 0.03 | 2.24 | 2.29 | 0.013 |
| Os.49621.1.S1_at | 0.00 | 2.24 | 1.28 | 0.013 |
| Os.32622.1.S1_at | 0.00 | 2.24 | 3.88 | 0.001 |
| Os.8553.1.S1_at | 0.00 | 2.24 | 3.49 | 0.028 |
| Os.14173.1.S1_at | 0.98 | 2.23 | 1.06 | 0.034 |
| Os.49739.1.S2_at | 0.01 | 2.23 | 1.20 | 0.008 |
| Os.11134.1.S1_at | 0.01 | 2.23 | 1.03 | 0.001 |
| Os.4999.1.S1_at | 0.00 | 2.23 | 1.73 | 0.036 |
| Os.13481.1.S1_at | 0.00 | 2.23 | 1.12 | 0.031 |
| Os.1521.1.S1_at | 0.05 | 2.23 | 1.11 | 0.036 |
| Os.9996.2.S1_at | 0.00 | 2.23 | 1.79 | 0.006 |
| Os.10822.1.S1_at | 0.01 | 2.22 | 1.09 | 0.047 |
| Os.25299.3.A1_at | 0.01 | 2.22 | 1.13 | 0.009 |
| Os.5388.1.S1_at | 0.00 | 2.22 | 1.50 | 0.044 |
| OsAffx.32263.1.S1_x_at | 0.00 | 2.22 | 2.04 | 0.025 |
| Os.51056.1.S1_at | 0.01 | 2.22 | 1.60 | 0.014 |
| OsAffx.25646.1.S1_at | 0.00 | 2.22 | 1.27 | 0.014 |
| Os.54353.1.S1_at | 0.00 | 2.22 | 21.46 | 0.007 |
| Os.46223.1.S1_s_at | 0.00 | 2.22 | 1.21 | 0.002 |
| Os.33607.1.S1_at | 1.46 | 2.22 | 1.03 | 0.039 |
| Os.23349.1.S1_x_at | 0.00 | 2.22 | 1.40 | 0.017 |
| Os.9688.1.S1_at | 0.05 | 2.22 | 1.03 | 0.001 |
| Os.2353.1.S1_at | 0.64 | 2.22 | 1.03 | 0.041 |
| Os.9272.1.S1_at | 0.02 | 2.22 | 1.15 | 0.014 |
| Os.6126.1.S1_at | 0.01 | 2.22 | 1.04 | 0.037 |
| Os.2243.2.S1_at | 0.00 | 2.22 | 1.34 | 0.009 |
| Os.31875.1.S1_at | 0.00 | 2.22 | 1.34 | 0.002 |
| Os.24044.1.S1_at | 0.07 | 2.22 | 1.01 | 0.015 |
| Os.10050.1.S1_at | 0.00 | 2.21 | 1.38 | 0.011 |
| Os.53103.1.S1_at | 0.17 | 2.21 | 1.10 | 0.045 |
| OsAffx.32229.1.S1_x_at | 0.00 | 2.21 | 1.24 | 0.005 |
| OsAffx.27959.1.S1_x_at | 0.00 | 2.20 | 1.57 | 0.035 |
| Os.4831.1.S1_a_at | 0.00 | 2.20 | 1.25 | 0.005 |
| Os.16631.1.S1_x_at | 0.05 | 2.20 | 1.07 | 0.037 |
| Os.26751.2.S1_at | 0.00 | 2.20 | 3.54 | 0.025 |
| OsAffx.29695.1.S1_s_at | 0.07 | 2.20 | 1.49 | 0.046 |
| Os.5486.1.S1_at | 0.02 | 2.20 | 1.06 | 0.001 |
| Os.49406.1.S1_at | 0.02 | 2.20 | 1.08 | 0.014 |
| Os.57130.1.S1_at | 0.01 | 2.20 | 1.37 | 0.023 |
| Os.5616.1.S1_at | 0.01 | 2.20 | 1.02 | 0.021 |
| Os.8353.1.S1_at | 0.02 | 2.20 | 1.17 | 0.012 |
| Os.17380.4.A1_x_at | 0.01 | 2.19 | 1.19 | 0.006 |
| Os.46070.1.S1_at | 0.00 | 2.19 | 1.20 | 0.014 |
| OsAffx.12107.1.S1_at | 0.64 | 2.19 | 1.06 | 0.044 |
| Os.48036.1.A1_at | 0.00 | 2.19 | 3.82 | 0.036 |
| Os.1182.1.S1_x_at | 0.00 | 2.19 | 1.98 | 0.042 |
| Os.25065.3.S1_at | 0.01 | 2.19 | 1.11 | 0.046 |
| Os.7567.1.S1_at | 0.00 | 2.19 | 1.30 | 0.026 |
| Os.27469.2.S1_at | 0.01 | 2.19 | 1.46 | 0.028 |
| OsAffx.14582.1.S1_at | 0.01 | 2.19 | 1.49 | 0.007 |
| Os.12101.2.A1_a_at | 0.02 | 2.19 | 1.68 | 0.013 |
| Os.53358.1.S1_at | 0.07 | 2.18 | 1.32 | 0.003 |
| Os.13102.1.S1_at | 0.64 | 2.18 | 1.04 | 0.008 |
| Os.10823.2.S1_at | 0.00 | 2.18 | 1.02 | 0.047 |
| Os.27377.1.S1_at | 0.05 | 2.18 | 1.21 | 0.034 |
| OsAffx.11906.1.S1_at | 0.02 | 2.18 | 1.26 | 0.007 |
| Os.14719.1.S1_at | 0.00 | 2.18 | 1.36 | 0.031 |
| OsAffx.32268.1.S1_x_at | 0.07 | 2.18 | 1.12 | 0.013 |
| OsAffx.5213.1.S1_x_at | 0.00 | 2.18 | 2.42 | 0.039 |
| Os.25169.1.S1_at | 0.98 | 2.18 | 1.01 | 0.005 |
| Os.22796.1.S1_at | 0.00 | 2.17 | 12.55 | 0.002 |
| Os.18335.3.S1_x_at | 0.00 | 2.17 | 8.10 | 0.010 |
| Os.52207.1.S1_at | 0.03 | 2.17 | 1.12 | 0.018 |
| Os.46498.2.S1_x_at | 0.00 | 2.17 | 1.29 | 0.046 |
| Os.12115.1.S1_at | 0.00 | 2.17 | 1.28 | 0.003 |
| Os.17714.1.S1_at | 0.00 | 2.17 | 1.10 | 0.022 |
| OsAffx.3180.1.S1_at | 0.00 | 2.16 | 1.62 | 0.003 |
| Os.18774.1.S1_s_at | 0.00 | 2.16 | 1.33 | 0.038 |
| Os.11889.1.S2_at | 0.07 | 2.16 | 1.14 | 0.027 |
| Os.47644.1.S1_at | 0.00 | 2.16 | 1.25 | 0.008 |
| Os.23664.1.S1_at | 0.07 | 2.16 | 1.11 | 0.027 |
| Os.47852.1.S1_at | 0.01 | 2.16 | 1.26 | 0.038 |
| OsAffx.32257.1.S1_x_at | 0.00 | 2.16 | 1.50 | 0.049 |
| Os.10084.1.A1_at | 0.64 | 2.16 | 1.06 | 0.041 |
| Os.17937.1.S1_a_at | 0.02 | 2.16 | 1.36 | 0.003 |
| Os.18490.3.S1_at | 0.00 | 2.15 | 1.29 | 0.015 |
| OsAffx.26050.37.S1_s_at | 0.00 | 2.15 | 1.79 | 0.021 |
| Os.17351.1.A1_at | 0.01 | 2.15 | 1.00 | 0.014 |
| Os.39984.1.S1_at | 0.00 | 2.15 | 1.54 | 0.005 |
| Os.2367.1.S1_at | 0.01 | 2.14 | 1.42 | 0.025 |
| Os.55328.1.S1_at | 0.03 | 2.14 | 1.26 | 0.005 |
| Os.7613.1.S1_at | 0.01 | 2.14 | 1.22 | 0.049 |
| Os.27196.1.S1_x_at | 0.00 | 2.14 | 1.10 | 0.001 |
| Os.52820.1.S1_at | 0.00 | 2.14 | 1.20 | 0.013 |
| Os.8003.1.S1_at | 0.00 | 2.14 | 1.15 | 0.028 |
| Os.27520.2.S1_x_at | 0.00 | 2.14 | 1.80 | 0.043 |
| Os.20538.2.S1_x_at | 0.03 | 2.14 | 1.03 | 0.013 |
| Os.50421.1.S1_at | 0.07 | 2.14 | 1.05 | 0.047 |
| Os.17841.2.S1_x_at | 0.00 | 2.14 | 1.18 | 0.030 |
| Os.33894.2.A1_at | 0.00 | 2.14 | 1.02 | 0.001 |
| OsAffx.19773.1.S1_at | 0.17 | 2.14 | 1.21 | 0.044 |
| Os.11888.1.S1_s_at | 0.00 | 2.14 | 1.17 | 0.012 |
| Os.14087.1.S1_at | 0.00 | 2.14 | 1.12 | 0.049 |
| Os.18600.1.S1_at | 0.03 | 2.13 | 1.30 | 0.040 |
| Os.7177.2.S1_a_at | 0.00 | 2.13 | 2.05 | 0.004 |
| Os.57181.1.S1_at | 0.00 | 2.13 | 1.17 | 0.036 |
| Os.16014.1.S1_at | 0.00 | 2.13 | 1.55 | 0.005 |
| Os.49393.1.S1_at | 0.00 | 2.13 | 1.55 | 0.022 |
| Os.50420.1.S1_s_at | 0.00 | 2.13 | 1.44 | 0.022 |
| Os.26783.2.S1_x_at | 0.07 | 2.13 | 1.01 | 0.005 |
| Os.5878.1.S1_at | 0.64 | 2.13 | 1.06 | 0.010 |
| Os.11193.1.S1_at | 0.00 | 2.13 | 1.16 | 0.037 |
| Os.12449.1.A1_at | 0.00 | 2.12 | 1.20 | 0.026 |
| Os.27656.1.S1_at | 0.05 | 2.12 | 1.15 | 0.017 |
| Os.26510.1.S1_at | 1.46 | 2.12 | 1.02 | 0.022 |
| OsAffx.3193.1.S1_at | 0.07 | 2.12 | 1.42 | 0.042 |
| OsAffx.32212.1.A1_at | 0.01 | 2.12 | 1.20 | 0.006 |
| Os.16883.1.S1_a_at | 0.02 | 2.12 | 1.22 | 0.044 |
| Os.34507.1.A1_at | 0.00 | 2.12 | 1.17 | 0.005 |
| Os.48020.1.S1_x_at | 0.00 | 2.12 | 1.45 | 0.040 |
| Os.15338.1.S1_a_at | 0.00 | 2.12 | 2.01 | 0.030 |
| Os.42960.1.S1_x_at | 0.00 | 2.12 | 1.27 | 0.022 |
| Os.8649.1.S1_at | 0.00 | 2.12 | 2.94 | 0.031 |
| Os.12125.1.S1_at | 0.11 | 2.12 | 1.01 | 0.021 |
| Os.27724.2.A1_a_at | 0.01 | 2.11 | 1.38 | 0.009 |
| Os.4730.1.S1_at | 0.03 | 2.11 | 1.69 | 0.032 |
| Os.8037.1.S2_at | 2.14 | 2.11 | 1.03 | 0.034 |
| OsAffx.12689.1.S1_s_at | 0.27 | 2.11 | 1.22 | 0.006 |
| Os.24093.2.S1_x_at | 0.03 | 2.11 | 1.17 | 0.023 |
| Os.21030.1.S1_at | 0.05 | 2.11 | 1.04 | 0.003 |
| Os.47398.2.A1_at | 0.00 | 2.11 | 1.66 | 0.009 |
| Os.5457.1.S1_at | 0.00 | 2.11 | 1.06 | 0.011 |
| Os.15454.2.S1_at | 0.00 | 2.11 | 2.33 | 0.047 |
| Os.9142.1.S1_at | 0.00 | 2.10 | 1.52 | 0.024 |
| Os.26362.1.S1_at | 0.64 | 2.10 | 1.05 | 0.041 |
| OsAffx.14122.1.S1_at | 0.00 | 2.10 | 1.11 | 0.025 |
| Os.24663.1.S1_s_at | 0.07 | 2.10 | 1.42 | 0.005 |
| Os.5619.1.A1_s_at | 0.01 | 2.10 | 1.76 | 0.006 |
| Os.47946.1.S1_s_at | 0.11 | 2.10 | 1.57 | 0.040 |
| Os.22967.1.S1_s_at | 0.00 | 2.10 | 6.08 | 0.049 |
| Os.20570.1.S1_x_at | 0.27 | 2.10 | 1.41 | 0.031 |
| Os.10901.1.S1_a_at | 0.00 | 2.09 | 1.37 | 0.017 |
| Os.6220.1.S1_at | 0.01 | 2.09 | 1.16 | 0.003 |
| Os.46776.1.S1_s_at | 0.00 | 2.09 | 1.45 | 0.001 |
| Os.55527.1.S1_at | 0.00 | 2.09 | 1.51 | 0.025 |
| Os.53316.1.S1_at | 0.00 | 2.09 | 1.38 | 0.035 |
| OsAffx.15319.1.S1_at | 0.64 | 2.09 | 1.21 | 0.013 |
| Os.57347.1.S1_at | 0.00 | 2.09 | 2.46 | 0.017 |
| Os.17618.2.S1_x_at | 0.01 | 2.09 | 1.12 | 0.012 |
| Os.9633.1.S1_a_at | 0.00 | 2.08 | 1.05 | 0.039 |
| OsAffx.14273.1.S1_at | 0.00 | 2.08 | 3.54 | 0.015 |
| OsAffx.4028.1.S1_x_at | 0.02 | 2.08 | 1.11 | 0.009 |
| Os.11344.1.S1_s_at | 0.01 | 2.08 | 1.14 | 0.039 |
| Os.54905.1.S1_at | 0.00 | 2.08 | 1.45 | 0.008 |
| Os.23819.1.S1_s_at | 0.00 | 2.08 | 1.54 | 0.024 |
| Os.53265.1.S1_at | 0.01 | 2.08 | 1.00 | 0.009 |
| Os.49163.1.S1_at | 0.00 | 2.08 | 2.10 | 0.015 |
| Os.8504.1.S1_at | 0.00 | 2.08 | 1.38 | 0.002 |
| Os.5771.1.S1_a_at | 0.00 | 2.08 | 1.15 | 0.007 |
| OsAffx.28880.1.S1_s_at | 0.00 | 2.08 | 2.03 | 0.035 |
| Os.2476.1.S1_at | 0.00 | 2.07 | 1.77 | 0.007 |
| Os.24424.1.S1_at | 0.05 | 2.07 | 1.05 | 0.039 |
| Os.7962.1.S1_a_at | 0.00 | 2.07 | 7.49 | 0.019 |
| Os.11111.1.S2_at | 0.01 | 2.07 | 1.05 | 0.004 |
| Os.12795.1.S1_at | 0.02 | 2.07 | 1.16 | 0.009 |
| Os.22623.1.S1_at | 0.17 | 2.07 | 1.11 | 0.026 |
| Os.50774.1.S1_at | 0.11 | 2.06 | 1.40 | 0.031 |
| Os.37955.1.S2_at | 0.00 | 2.06 | 1.01 | 0.006 |
| Os.16976.1.S1_at | 0.00 | 2.06 | 1.54 | 0.041 |
| Os.50938.1.S1_at | 0.00 | 2.06 | 8.89 | 0.011 |
| OsAffx.29101.1.S1_at | 0.00 | 2.06 | 1.07 | 0.000 |
| Os.9351.1.S1_at | 0.00 | 2.06 | 4.23 | 0.019 |
| Os.15717.1.S1_at | 0.01 | 2.06 | 1.03 | 0.046 |
| OsAffx.5226.1.S1_at | 0.03 | 2.06 | 1.23 | 0.019 |
| Os.27118.1.A1_at | 0.00 | 2.06 | 1.84 | 0.018 |
| Os.12728.1.S1_at | 0.03 | 2.06 | 1.48 | 0.040 |
| Os.8381.1.S1_at | 0.01 | 2.05 | 1.13 | 0.019 |
| Os.32970.1.S1_at | 0.05 | 2.05 | 1.02 | 0.043 |
| OsAffx.30394.1.S1_at | 0.00 | 2.05 | 1.86 | 0.007 |
| Os.12196.1.S1_at | 0.00 | 2.05 | 1.39 | 0.017 |
| Os.9125.1.S1_at | 0.07 | 2.05 | 1.00 | 0.026 |
| Os.8495.1.S1_at | 0.41 | 2.05 | 1.07 | 0.009 |
| Os.52168.1.S1_at | 0.01 | 2.05 | 1.02 | 0.007 |
| Os.14046.1.S1_at | 2.14 | 2.05 | 1.00 | 0.044 |
| Os.49742.1.S1_at | 2.14 | 2.04 | 1.11 | 0.017 |
| Os.38110.1.S1_at | 0.00 | 2.04 | 1.10 | 0.041 |
| Os.53698.1.S1_at | 0.00 | 2.04 | 1.38 | 0.001 |
| Os.49419.1.S1_at | 0.01 | 2.04 | 1.16 | 0.032 |
| OsAffx.17386.1.S1_at | 2.14 | 2.04 | 1.03 | 0.048 |
| Os.6265.1.S1_at | 0.27 | 2.04 | 1.29 | 0.008 |
| Os.9224.1.S1_at | 0.17 | 2.04 | 1.17 | 0.010 |
| Os.13481.1.S1_s_at | 0.01 | 2.04 | 1.13 | 0.026 |
| Os.7589.1.S1_at | 0.00 | 2.04 | 1.57 | 0.003 |
| Os.54791.1.S1_at | 0.00 | 2.04 | 6.40 | 0.042 |
| OsAffx.32221.1.S1_s_at | 0.00 | 2.04 | 1.05 | 0.024 |
| OsAffx.31482.1.S1_s_at | 0.01 | 2.04 | 1.03 | 0.049 |
| Os.33341.2.S1_x_at | 0.00 | 2.04 | 1.10 | 0.048 |
| Os.16884.1.S1_at | 0.00 | 2.04 | 1.24 | 0.010 |
| Os.12218.1.S1_at | 0.00 | 2.03 | 1.25 | 0.022 |
| Os.28438.1.S1_x_at | 0.00 | 2.03 | 3.60 | 0.003 |
| Os.20864.1.A1_at | 0.00 | 2.03 | 1.16 | 0.007 |
| Os.54742.1.S1_at | 0.02 | 2.03 | 1.14 | 0.008 |
| Os.53598.1.S1_at | 0.00 | 2.03 | 4.11 | 0.033 |
| OsAffx.24863.1.S1_x_at | 0.00 | 2.03 | 2.29 | 0.045 |
| OsAffx.29871.1.S1_x_at | 0.00 | 2.03 | 2.67 | 0.042 |
| Os.54673.1.S1_at | 0.11 | 2.03 | 1.01 | 0.021 |
| OsAffx.16345.1.S1_s_at | 0.05 | 2.03 | 1.07 | 0.005 |
| Os.21192.3.S1_x_at | 0.01 | 2.03 | 1.12 | 0.020 |
| OsAffx.29501.1.S1_at | 0.00 | 2.03 | 1.01 | 0.002 |
| Os.54799.1.A1_at | 0.11 | 2.02 | 1.11 | 0.034 |
| Os.7971.1.S1_x_at | 0.00 | 2.02 | 1.73 | 0.045 |
| Os.46065.1.S1_at | 0.27 | 2.02 | 1.08 | 0.026 |
| Os.24019.1.S1_at | 0.05 | 2.02 | 1.05 | 0.010 |
| Os.23178.1.S1_at | 0.00 | 2.02 | 1.08 | 0.009 |
| Os.54960.1.S1_at | 0.02 | 2.02 | 1.22 | 0.042 |
| Os.11637.1.S1_at | 0.05 | 2.02 | 1.11 | 0.037 |
| OsAffx.26273.1.S1_at | 0.00 | 2.02 | 1.53 | 0.005 |
| Os.12052.1.S1_at | 0.02 | 2.01 | 1.23 | 0.012 |
| Os.32455.1.S1_at | 0.00 | 2.01 | 1.55 | 0.027 |
| Os.52358.1.S1_at | 0.05 | 2.01 | 1.15 | 0.017 |
| Os.10076.1.S1_at | 0.01 | 2.01 | 1.04 | 0.018 |
| Os.17162.2.S1_x_at | 0.41 | 2.01 | 1.02 | 0.030 |
| Os.6257.1.S1_at | 0.00 | 2.01 | 1.02 | 0.005 |
| Os.17597.1.S1_at | 0.01 | 2.01 | 1.29 | 0.039 |
| Os.52497.1.S1_at | 0.01 | 2.01 | 1.47 | 0.004 |
| Os.1316.1.S1_a_at | 0.00 | 2.01 | 1.17 | 0.047 |
| OsAffx.32258.1.S1_x_at | 0.00 | 2.01 | 1.40 | 0.048 |
| Os.27452.1.S1_at | 0.17 | 2.00 | 1.01 | 0.046 |
| Os.10378.2.A1_s_at | 0.17 | 2.00 | 1.09 | 0.021 |
| Os.46626.1.S1_x_at | 0.01 | 2.00 | 1.21 | 0.012 |
| Os.1070.1.S1_at | 0.01 | 2.00 | 1.24 | 0.047 |
| Os.18321.1.S1_at | 0.00 | 2.00 | 1.38 | 0.000 |
| Os.32503.1.S1_x_at | 0.27 | 2.00 | 1.01 | 0.026 |
| OsAffx.19795.1.S1_s_at | 0.27 | 2.00 | 1.57 | 0.040 |
| Os.37961.1.S1_s_at | 0.03 | 2.00 | 1.01 | 0.008 |
| Os.11988.1.S1_at | 0.00 | 2.00 | 1.30 | 0.027 |
| Os.18653.1.S1_at | 0.01 | 2.00 | 1.27 | 0.000 |
| Os.8103.1.S1_at | 0.02 | 2.00 | 1.13 | 0.000 |
| Os.47322.1.A1_at | 0.00 | 2.00 | 5.28 | 0.002 |
| OsAffx.17638.1.S1_at | 0.41 | 1.99 | 1.11 | 0.009 |
| Os.11407.1.S1_at | 0.05 | 1.99 | 1.18 | 0.044 |
| OsAffx.30149.1.S1_s_at | 0.00 | 1.99 | 5.87 | 0.015 |
| Os.2657.1.S1_x_at | 0.01 | 1.99 | 1.50 | 0.049 |
| Os.8347.1.S1_at | 0.00 | 1.99 | 1.10 | 0.012 |
| Os.7864.1.S1_at | 0.00 | 1.99 | 1.55 | 0.041 |
| OsAffx.23225.1.S1_x_at | 0.00 | 1.99 | 1.48 | 0.003 |
| Os.4387.1.S1_at | 0.01 | 1.99 | 1.84 | 0.006 |
| Os.22623.1.S1_x_at | 0.07 | 1.99 | 1.08 | 0.007 |
| OsAffx.26590.1.S1_at | 0.02 | 1.99 | 1.01 | 0.037 |
| Os.10800.1.S1_at | 0.00 | 1.99 | 1.28 | 0.024 |
| Os.24556.1.S1_at | 0.64 | 1.98 | 1.13 | 0.002 |
| OsAffx.28387.1.S1_s_at | 0.00 | 1.98 | 1.36 | 0.038 |
| Os.46329.1.S1_at | 0.02 | 1.98 | 1.03 | 0.044 |
| Os.33610.3.S1_s_at | 0.01 | 1.98 | 1.04 | 0.018 |
| Os.47949.1.S1_a_at | 0.02 | 1.98 | 1.41 | 0.000 |
| OsAffx.11057.1.S1_x_at | 0.00 | 1.98 | 1.32 | 0.042 |
| Os.50583.1.S1_at | 0.00 | 1.98 | 3.72 | 0.020 |
| Os.36706.1.S1_at | 0.00 | 1.98 | 1.46 | 0.026 |
| Os.16018.1.S1_at | 0.00 | 1.98 | 2.46 | 0.014 |
| Os.27212.1.A1_at | 0.11 | 1.98 | 1.08 | 0.024 |
| Os.10452.1.S1_at | 0.00 | 1.97 | 1.19 | 0.013 |
| Os.16460.1.S1_at | 0.07 | 1.97 | 1.00 | 0.025 |
| Os.20299.4.S1_x_at | 0.01 | 1.97 | 1.02 | 0.004 |
| Os.37384.1.S1_s_at | 0.03 | 1.97 | 1.39 | 0.021 |
| Os.2242.1.S1_at | 0.00 | 1.97 | 1.55 | 0.010 |
| Os.15099.1.S1_at | 0.01 | 1.97 | 1.19 | 0.025 |
| Os.50488.1.S1_at | 0.00 | 1.97 | 2.17 | 0.001 |
| Os.32071.2.S1_x_at | 0.00 | 1.97 | 1.11 | 0.009 |
| Os.37562.1.A1_x_at | 0.00 | 1.97 | 9.80 | 0.043 |
| Os.37370.1.S1_at | 0.05 | 1.97 | 1.02 | 0.001 |
| OsAffx.32204.1.S1_x_at | 0.11 | 1.97 | 1.11 | 0.013 |
| OsAffx.27244.1.S1_x_at | 0.00 | 1.96 | 12.00 | 0.025 |
| Os.26978.1.S1_a_at | 0.41 | 1.96 | 1.01 | 0.046 |
| Os.12645.1.S1_at | 0.00 | 1.96 | 1.02 | 0.041 |
| Os.18589.1.S1_at | 0.01 | 1.96 | 1.21 | 0.016 |
| Os.11921.1.S1_at | 0.00 | 1.96 | 1.27 | 0.015 |
| Os.11007.1.S1_at | 0.00 | 1.96 | 1.21 | 0.040 |
| Os.56996.1.S1_at | 0.00 | 1.96 | 3.19 | 0.026 |
| Os.9077.1.S1_at | 0.00 | 1.96 | 1.21 | 0.044 |
| Os.5729.1.S1_at | 0.00 | 1.96 | 1.66 | 0.010 |
| Os.6264.1.S1_at | 0.02 | 1.96 | 1.49 | 0.037 |
| Os.23131.1.S1_at | 0.00 | 1.95 | 1.29 | 0.008 |
| OsAffx.21381.1.S1_s_at | 0.27 | 1.95 | 1.02 | 0.043 |
| Os.52658.1.S1_at | 0.00 | 1.95 | 1.52 | 0.030 |
| Os.20681.1.S1_x_at | 0.00 | 1.95 | 1.23 | 0.037 |
| Os.46592.2.S1_x_at | 0.05 | 1.95 | 1.06 | 0.030 |
| OsAffx.24325.1.S1_at | 0.17 | 1.95 | 1.14 | 0.042 |
| OsAffx.24693.1.S1_at | 0.00 | 1.95 | 1.24 | 0.007 |
| Os.12627.1.S1_at | 0.00 | 1.95 | 3.68 | 0.008 |
| OsAffx.3602.1.S1_at | 0.41 | 1.95 | 1.07 | 0.013 |
| Os.10240.1.S1_at | 0.00 | 1.95 | 2.75 | 0.022 |
| Os.4647.1.S1_at | 0.98 | 1.95 | 1.14 | 0.021 |
| Os.24561.1.S1_s_at | 0.07 | 1.95 | 1.17 | 0.025 |
| OsAffx.26384.8.S1_x_at | 0.00 | 1.95 | 1.33 | 0.010 |
| Os.7865.1.S1_at | 0.00 | 1.95 | 5.48 | 0.027 |
| Os.4174.1.S1_at | 0.07 | 1.95 | 1.29 | 0.029 |
| OsAffx.22955.1.S1_x_at | 0.00 | 1.95 | 1.46 | 0.034 |
| Os.137.1.S1_at | 0.00 | 1.95 | 1.25 | 0.021 |
| Os.11875.1.S1_a_at | 0.00 | 1.94 | 1.08 | 0.034 |
| Os.18923.1.S1_a_at | 0.01 | 1.94 | 1.01 | 0.008 |
| Os.12024.1.S1_at | 0.00 | 1.94 | 1.71 | 0.032 |
| Os.4898.1.S1_at | 0.98 | 1.94 | 1.05 | 0.047 |
| OsAffx.6960.1.S1_s_at | 0.07 | 1.94 | 1.11 | 0.009 |
| Os.50756.1.S1_at | 0.05 | 1.94 | 1.15 | 0.005 |
| Os.14423.1.S1_x_at | 0.01 | 1.94 | 1.53 | 0.006 |
| Os.1229.1.S1_at | 0.01 | 1.94 | 1.65 | 0.030 |
| Os.27038.1.S1_at | 0.05 | 1.94 | 1.46 | 0.009 |
| Os.20299.1.S1_a_at | 0.01 | 1.94 | 1.02 | 0.010 |
| Os.24631.1.A1_s_at | 0.03 | 1.94 | 1.14 | 0.018 |
| Os.54619.1.S1_at | 0.64 | 1.94 | 1.06 | 0.023 |
| Os.8913.1.S1_at | 0.01 | 1.94 | 1.25 | 0.003 |
| Os.24760.1.A1_at | 0.41 | 1.93 | 1.12 | 0.016 |
| Os.9484.1.S2_at | 0.00 | 1.93 | 5.75 | 0.002 |
| Os.8455.1.S1_at | 0.00 | 1.93 | 3.05 | 0.032 |
| Os.11634.1.S1_at | 0.98 | 1.93 | 1.17 | 0.003 |
| OsAffx.15320.1.S1_x_at | 0.27 | 1.93 | 1.05 | 0.019 |
| Os.52409.1.S1_at | 0.01 | 1.93 | 1.06 | 0.033 |
| OsAffx.14786.1.S1_s_at | 0.00 | 1.93 | 1.12 | 0.033 |
| Os.13649.1.S1_at | 0.17 | 1.93 | 1.24 | 0.010 |
| Os.27630.1.A1_at | 0.00 | 1.93 | 1.01 | 0.042 |
| Os.8168.1.S1_at | 0.00 | 1.93 | 1.04 | 0.005 |
| Os.23164.1.S1_a_at | 0.00 | 1.93 | 1.12 | 0.003 |
| Os.6868.1.S1_at | 0.00 | 1.93 | 1.98 | 0.017 |
| Os.23868.1.S1_at | 0.00 | 1.93 | 6.22 | 0.034 |
| Os.50609.1.S1_at | 0.00 | 1.93 | 4.75 | 0.002 |
| Os.8756.2.A1_at | 0.00 | 1.93 | 1.13 | 0.016 |
| Os.11704.1.S1_at | 0.01 | 1.92 | 1.09 | 0.020 |
| Os.40415.1.A1_s_at | 0.00 | 1.92 | 1.42 | 0.045 |
| Os.17432.1.S1_at | 0.01 | 1.92 | 1.23 | 0.044 |
| Os.20672.1.S2_at | 0.02 | 1.92 | 1.15 | 0.004 |
| Os.9144.1.S1_a_at | 0.00 | 1.92 | 2.06 | 0.002 |
| Os.24421.1.S1_at | 0.00 | 1.92 | 1.81 | 0.049 |
| Os.9889.1.S1_at | 0.02 | 1.92 | 1.04 | 0.021 |
| OsAffx.17671.1.S1_at | 0.07 | 1.91 | 1.05 | 0.035 |
| Os.5331.1.S1_at | 0.00 | 1.91 | 1.45 | 0.004 |
| Os.53214.1.S1_at | 0.00 | 1.91 | 1.08 | 0.027 |
| Os.54612.1.A1_at | 0.00 | 1.91 | 1.16 | 0.014 |
| Os.12148.1.S1_at | 0.00 | 1.91 | 1.18 | 0.007 |
| Os.609.1.S2_a_at | 0.00 | 1.91 | 1.24 | 0.018 |
| Os.24545.2.S1_x_at | 0.00 | 1.91 | 1.12 | 0.030 |
| Os.7502.1.S1_at | 0.03 | 1.91 | 1.02 | 0.035 |
| Os.19279.2.S1_x_at | 0.07 | 1.91 | 1.22 | 0.025 |
| Os.35797.1.S1_at | 0.64 | 1.91 | 1.40 | 0.027 |
| Os.3372.1.S1_at | 0.01 | 1.91 | 1.36 | 0.017 |
| Os.6262.1.S1_at | 0.00 | 1.91 | 2.37 | 0.034 |
| Os.49353.1.S1_at | 0.05 | 1.91 | 1.06 | 0.039 |
| Os.9262.1.S1_at | 0.17 | 1.91 | 1.10 | 0.037 |
| Os.11943.1.S1_at | 0.17 | 1.90 | 1.00 | 0.038 |
| Os.27729.1.S1_x_at | 0.17 | 1.90 | 1.46 | 0.037 |
| Os.15545.1.S1_at | 0.64 | 1.90 | 1.12 | 0.006 |
| Os.5151.1.S1_at | 0.00 | 1.90 | 1.20 | 0.041 |
| Os.4531.1.S1_at | 0.01 | 1.90 | 1.21 | 0.031 |
| Os.9235.1.S1_at | 0.03 | 1.90 | 1.04 | 0.001 |
| Os.46919.1.S1_at | 0.00 | 1.90 | 1.67 | 0.010 |
| Os.43375.1.S1_at | 0.00 | 1.90 | 1.50 | 0.035 |
| Os.9948.1.S1_at | 0.17 | 1.90 | 1.10 | 0.017 |
| Os.1445.1.S1_at | 0.41 | 1.90 | 1.16 | 0.025 |
| OsAffx.17869.1.S1_at | 0.41 | 1.90 | 1.10 | 0.007 |
| Os.13478.1.S1_at | 0.00 | 1.90 | 1.69 | 0.026 |
| Os.24852.1.A1_at | 0.00 | 1.90 | 2.03 | 0.032 |
| OsAffx.14759.1.S1_s_at | 0.05 | 1.90 | 1.13 | 0.015 |
| Os.21627.1.S1_at | 0.07 | 1.90 | 1.25 | 0.028 |
| Os.12703.1.S1_at | 0.00 | 1.90 | 1.41 | 0.006 |
| Os.17919.1.S1_at | 0.05 | 1.90 | 1.10 | 0.023 |
| Os.49148.2.S1_at | 0.00 | 1.89 | 1.30 | 0.019 |
| Os.20884.1.S1_at | 0.64 | 1.89 | 1.08 | 0.045 |
| Os.50557.1.S1_at | 0.00 | 1.89 | 5.41 | 0.030 |
| Os.11888.3.S1_x_at | 0.00 | 1.89 | 1.34 | 0.010 |
| Os.9902.1.S1_at | 0.27 | 1.89 | 1.04 | 0.006 |
| Os.49187.1.S1_at | 0.17 | 1.89 | 1.03 | 0.000 |
| Os.51983.1.A1_x_at | 0.07 | 1.89 | 1.02 | 0.027 |
| Os.46545.2.S1_x_at | 1.46 | 1.89 | 1.05 | 0.018 |
| Os.53543.1.A1_at | 0.07 | 1.89 | 1.19 | 0.011 |
| Os.41793.1.S1_at | 0.01 | 1.89 | 1.19 | 0.001 |
| Os.53726.1.S1_at | 0.01 | 1.88 | 1.72 | 0.039 |
| Os.7525.1.S1_at | 0.00 | 1.88 | 1.42 | 0.046 |
| OsAffx.19845.1.S1_at | 0.05 | 1.88 | 1.28 | 0.031 |
| Os.11166.1.S1_s_at | 0.07 | 1.88 | 1.20 | 0.028 |
| Os.54191.1.S1_at | 0.00 | 1.88 | 4.74 | 0.004 |
| Os.10786.1.S1_at | 0.03 | 1.88 | 1.44 | 0.029 |
| Os.35005.1.S1_x_at | 0.00 | 1.88 | 1.31 | 0.036 |
| Os.21835.1.S1_at | 0.02 | 1.88 | 1.26 | 0.028 |
| Os.11138.2.S1_x_at | 0.41 | 1.88 | 1.01 | 0.027 |
| Os.17226.1.S1_s_at | 0.00 | 1.88 | 1.06 | 0.010 |
| Os.46065.2.S1_x_at | 0.41 | 1.88 | 1.02 | 0.035 |
| Os.54601.1.S1_s_at | 0.17 | 1.88 | 1.05 | 0.030 |
| Os.8200.2.S1_x_at | 1.46 | 1.88 | 1.16 | 0.020 |
| Os.50932.1.S1_at | 0.01 | 1.87 | 1.17 | 0.023 |
| Os.10548.1.S1_s_at | 0.00 | 1.87 | 7.10 | 0.005 |
| Os.33336.1.S1_at | 0.01 | 1.87 | 2.09 | 0.018 |
| Os.52929.1.S1_x_at | 0.27 | 1.87 | 1.09 | 0.020 |
| Os.5399.1.S1_at | 0.03 | 1.87 | 1.04 | 0.021 |
| Os.16983.1.S1_at | 0.17 | 1.87 | 1.08 | 0.012 |
| Os.30055.1.S1_x_at | 0.17 | 1.87 | 1.01 | 0.040 |
| Os.10370.2.S1_at | 0.00 | 1.87 | 9.31 | 0.008 |
| Os.19817.1.S1_at | 0.00 | 1.87 | 1.38 | 0.008 |
| Os.53527.1.S1_at | 0.00 | 1.87 | 1.07 | 0.044 |
| Os.33220.1.S1_at | 0.00 | 1.87 | 1.57 | 0.020 |
| Os.4701.1.S1_at | 0.01 | 1.87 | 1.31 | 0.029 |
| Os.9954.1.S1_at | 0.07 | 1.86 | 1.15 | 0.031 |
| Os.12283.1.S1_at | 0.00 | 1.86 | 3.00 | 0.002 |
| Os.9487.1.S1_a_at | 0.00 | 1.86 | 1.04 | 0.024 |
| Os.49426.1.S1_at | 0.07 | 1.86 | 1.15 | 0.007 |
| Os.5433.1.S1_at | 0.05 | 1.86 | 1.06 | 0.002 |
| Os.12713.1.S1_at | 0.00 | 1.86 | 5.09 | 0.035 |
| OsAffx.26746.1.S1_at | 0.00 | 1.86 | 1.14 | 0.016 |
| Os.23940.1.A1_at | 0.00 | 1.86 | 2.23 | 0.002 |
| Os.27235.2.S1_x_at | 0.41 | 1.86 | 1.26 | 0.023 |
| Os.47600.2.S1_x_at | 0.00 | 1.86 | 1.42 | 0.003 |
| Os.38774.1.S1_s_at | 0.27 | 1.86 | 1.10 | 0.044 |
| Os.27832.1.A1_s_at | 0.11 | 1.86 | 1.10 | 0.028 |
| Os.32953.1.S1_at | 0.02 | 1.86 | 1.05 | 0.021 |
| Os.6086.2.S1_x_at | 3.01 | 1.86 | 1.02 | 0.048 |
| Os.49151.1.S1_at | 0.00 | 1.86 | 6.90 | 0.037 |
| Os.8776.1.S1_at | 0.05 | 1.86 | 1.16 | 0.034 |
| Os.52602.1.S1_x_at | 0.01 | 1.86 | 1.19 | 0.018 |
| Os.7593.1.S1_at | 0.00 | 1.86 | 1.54 | 0.003 |
| Os.11185.1.S1_a_at | 0.41 | 1.85 | 1.11 | 0.011 |
| Os.50945.1.S1_at | 0.00 | 1.85 | 2.01 | 0.006 |
| Os.37320.2.S1_x_at | 0.00 | 1.85 | 1.34 | 0.015 |
| Os.47368.1.S1_x_at | 0.00 | 1.85 | 6.96 | 0.016 |
| Os.45953.1.S1_at | 0.00 | 1.85 | 11.87 | 0.013 |
| Os.8788.1.S1_at | 0.11 | 1.85 | 1.03 | 0.026 |
| Os.40410.1.A1_s_at | 0.27 | 1.85 | 1.03 | 0.020 |
| Os.22668.1.A1_at | 0.05 | 1.85 | 1.26 | 0.012 |
| Os.15988.1.S1_at | 0.01 | 1.85 | 1.25 | 0.017 |
| Os.46422.1.S1_at | 0.00 | 1.85 | 4.79 | 0.007 |
| OsAffx.2480.1.S1_at | 0.03 | 1.85 | 1.24 | 0.050 |
| Os.12165.1.S1_at | 0.27 | 1.85 | 1.12 | 0.047 |
| Os.9242.1.A1_at | 0.41 | 1.85 | 1.02 | 0.016 |
| Os.4995.1.S1_at | 0.00 | 1.85 | 1.74 | 0.041 |
| Os.18590.1.S1_a_at | 0.00 | 1.85 | 3.61 | 0.001 |
| Os.19777.1.A1_at | 0.00 | 1.85 | 1.87 | 0.013 |
| Os.47958.1.A1_x_at | 0.17 | 1.85 | 1.09 | 0.039 |
| OsAffx.14496.1.S1_at | 0.00 | 1.85 | 1.32 | 0.008 |
| Os.8200.1.S1_s_at | 0.07 | 1.85 | 1.09 | 0.005 |
| Os.30049.1.S1_at | 0.03 | 1.84 | 1.10 | 0.021 |
| Os.15725.1.S1_at | 0.00 | 1.84 | 2.30 | 0.013 |
| Os.37742.1.S1_at | 0.02 | 1.84 | 1.02 | 0.000 |
| OsAffx.3776.1.S1_at | 0.00 | 1.84 | 1.90 | 0.045 |
| Os.7961.1.S1_s_at | 0.64 | 1.84 | 1.02 | 0.028 |
| Os.20424.1.S1_s_at | 0.00 | 1.84 | 1.16 | 0.012 |
| Os.50643.2.S1_x_at | 0.00 | 1.84 | 2.98 | 0.041 |
| OsAffx.26050.2.S2_x_at | 0.00 | 1.84 | 1.49 | 0.023 |
| Os.14353.1.S1_s_at | 0.00 | 1.84 | 1.25 | 0.018 |
| Os.53309.1.S1_at | 0.07 | 1.84 | 1.12 | 0.036 |
| Os.12420.1.S1_at | 0.00 | 1.84 | 1.00 | 0.023 |
| OsAffx.26779.2.S1_at | 0.00 | 1.84 | 7.90 | 0.034 |
| Os.8476.1.S1_at | 0.00 | 1.84 | 1.23 | 0.019 |
| Os.26998.1.S2_at | 0.11 | 1.84 | 1.02 | 0.049 |
| Os.54106.1.S1_at | 0.00 | 1.84 | 1.22 | 0.043 |
| Os.24268.1.S1_x_at | 0.03 | 1.84 | 2.43 | 0.012 |
| Os.7972.1.S2_at | 0.01 | 1.83 | 1.21 | 0.050 |
| Os.25806.1.S1_s_at | 0.27 | 1.83 | 1.02 | 0.010 |
| Os.16326.1.S1_at | 0.00 | 1.83 | 2.39 | 0.035 |
| OsAffx.28068.1.S1_at | 0.11 | 1.83 | 1.04 | 0.018 |
| Os.33110.1.S1_at | 0.01 | 1.83 | 1.10 | 0.050 |
| Os.9262.1.S2_at | 0.41 | 1.83 | 1.26 | 0.011 |
| Os.12931.1.S1_at | 0.27 | 1.83 | 1.01 | 0.024 |
| Os.50910.1.S1_at | 0.64 | 1.83 | 1.02 | 0.034 |
| OsAffx.32204.1.A1_x_at | 0.02 | 1.83 | 1.03 | 0.021 |
| Os.17828.1.S1_s_at | 0.41 | 1.83 | 1.08 | 0.045 |
| Os.10279.1.S1_at | 0.41 | 1.83 | 1.05 | 0.021 |
| Os.49095.1.S1_at | 0.00 | 1.83 | 2.79 | 0.011 |
| Os.48038.1.S1_at | 0.03 | 1.82 | 1.29 | 0.008 |
| Os.353.1.S1_at | 0.02 | 1.82 | 1.33 | 0.018 |
| Os.28133.1.S1_x_at | 0.00 | 1.82 | 9.47 | 0.004 |
| Os.31693.3.S1_at | 0.98 | 1.82 | 1.04 | 0.022 |
| Os.17072.1.S1_at | 0.00 | 1.82 | 1.13 | 0.003 |
| Os.7057.1.S1_at | 0.03 | 1.82 | 1.11 | 0.004 |
| Os.50942.1.S1_at | 0.00 | 1.82 | 2.28 | 0.023 |
| Os.10154.1.S1_at | 0.00 | 1.82 | 1.81 | 0.043 |
| Os.14911.1.S1_at | 0.01 | 1.82 | 1.13 | 0.000 |
| Os.8136.1.A1_at | 0.00 | 1.82 | 1.45 | 0.023 |
| Os.52838.1.S1_at | 0.00 | 1.82 | 3.15 | 0.003 |
| Os.49455.1.S1_at | 0.00 | 1.81 | 1.12 | 0.019 |
| OsAffx.27550.1.S1_at | 0.00 | 1.81 | 2.68 | 0.000 |
| Os.2131.1.S1_s_at | 0.07 | 1.81 | 1.38 | 0.020 |
| Os.6590.1.S1_at | 0.00 | 1.81 | 4.36 | 0.012 |
| Os.17380.1.S1_x_at | 0.27 | 1.81 | 1.46 | 0.016 |
| Os.23200.1.S1_x_at | 0.03 | 1.81 | 1.44 | 0.034 |
| Os.12216.1.S1_at | 0.64 | 1.81 | 1.01 | 0.038 |
| Os.50813.1.S1_at | 0.11 | 1.81 | 1.12 | 0.016 |
| Os.11152.1.S1_at | 0.00 | 1.80 | 1.33 | 0.020 |
| Os.10817.1.S1_a_at | 0.07 | 1.80 | 1.24 | 0.022 |
| Os.23200.2.S1_x_at | 0.00 | 1.80 | 1.13 | 0.021 |
| Os.11808.1.S1_at | 0.00 | 1.80 | 1.60 | 0.017 |
| Os.49626.1.S1_at | 0.00 | 1.80 | 5.41 | 0.008 |
| Os.44676.1.S1_at | 0.00 | 1.80 | 1.05 | 0.019 |
| OsAffx.32326.1.S1_x_at | 0.27 | 1.80 | 1.34 | 0.034 |
| Os.3278.1.S1_at | 0.17 | 1.80 | 1.03 | 0.046 |
| Os.15037.1.S2_at | 0.01 | 1.80 | 1.55 | 0.022 |
| Os.52981.1.S1_at | 0.00 | 1.80 | 1.15 | 0.033 |
| OsAffx.27399.1.S1_at | 0.03 | 1.80 | 1.13 | 0.022 |
| Os.47853.1.A1_at | 0.27 | 1.79 | 1.24 | 0.035 |
| Os.50236.1.S1_at | 1.46 | 1.79 | 1.03 | 0.045 |
| Os.19006.1.S1_a_at | 0.01 | 1.79 | 1.06 | 0.049 |
| Os.22453.1.S1_at | 0.01 | 1.79 | 1.38 | 0.009 |
| Os.27164.1.A1_a_at | 0.03 | 1.79 | 1.16 | 0.011 |
| Os.46135.1.S1_x_at | 0.00 | 1.79 | 1.87 | 0.010 |
| Os.17215.1.S2_at | 0.98 | 1.79 | 1.03 | 0.015 |
| OsAffx.18040.1.S1_at | 0.00 | 1.79 | 5.18 | 0.015 |
| Os.7396.2.S1_a_at | 0.05 | 1.79 | 1.73 | 0.029 |
| OsAffx.7765.1.S1_at | 0.03 | 1.79 | 1.02 | 0.005 |
| Os.7974.1.S1_at | 0.00 | 1.79 | 3.53 | 0.036 |
| Os.8453.1.S1_a_at | 0.01 | 1.78 | 1.00 | 0.021 |
| OsAffx.26050.8.S1_x_at | 0.01 | 1.78 | 1.29 | 0.032 |
| OsAffx.28174.1.S1_x_at | 0.03 | 1.78 | 1.16 | 0.026 |
| Os.24275.1.S2_at | 0.05 | 1.78 | 1.75 | 0.041 |
| Os.15046.1.S1_at | 0.00 | 1.78 | 1.00 | 0.013 |
| Os.34638.1.S1_at | 0.07 | 1.78 | 1.13 | 0.014 |
| Os.16736.1.S1_s_at | 0.00 | 1.78 | 1.22 | 0.048 |
| OsAffx.14846.1.S1_at | 0.00 | 1.78 | 2.15 | 0.014 |
| Os.50548.2.S1_x_at | 0.00 | 1.78 | 2.15 | 0.024 |
| OsAffx.25952.1.S1_x_at | 0.11 | 1.78 | 1.09 | 0.032 |
| Os.3354.1.S1_at | 0.01 | 1.78 | 1.19 | 0.022 |
| Os.5082.1.S1_a_at | 0.00 | 1.78 | 1.35 | 0.007 |
| Os.48707.1.S1_at | 0.00 | 1.78 | 2.57 | 0.003 |
| Os.50766.1.S1_at | 0.00 | 1.78 | 1.33 | 0.026 |
| OsAffx.11589.1.S1_at | 0.00 | 1.78 | 1.99 | 0.012 |
| Os.7896.2.S1_at | 3.01 | 1.78 | 1.05 | 0.045 |
| OsAffx.28461.1.S1_at | 0.05 | 1.77 | 1.29 | 0.016 |
| Os.17219.1.S1_at | 0.01 | 1.77 | 1.78 | 0.014 |
| Os.47323.1.S1_x_at | 0.00 | 1.77 | 4.80 | 0.035 |
| Os.53086.1.S1_at | 0.01 | 1.77 | 1.36 | 0.039 |
| Os.20672.1.S1_s_at | 0.64 | 1.77 | 1.10 | 0.013 |
| Os.32192.1.S1_at | 0.00 | 1.77 | 4.79 | 0.025 |
| Os.7790.1.S1_at | 0.01 | 1.77 | 1.14 | 0.024 |
| Os.51228.3.S1_x_at | 0.07 | 1.77 | 1.08 | 0.045 |
| Os.19307.1.S1_at | 0.00 | 1.77 | 1.09 | 0.010 |
| Os.28769.1.S2_at | 0.00 | 1.77 | 3.19 | 0.031 |
| Os.46398.1.S1_at | 0.17 | 1.77 | 1.07 | 0.017 |
| Os.31545.1.S1_at | 0.00 | 1.77 | 13.31 | 0.029 |
| Os.41841.1.S1_at | 0.00 | 1.77 | 2.46 | 0.000 |
| Os.28409.20.A1_a_at | 0.02 | 1.77 | 1.14 | 0.044 |
| OsAffx.5881.1.S1_s_at | 0.07 | 1.77 | 1.02 | 0.017 |
| Os.52584.1.S1_at | 0.03 | 1.77 | 1.43 | 0.049 |
| Os.7317.2.S1_at | 0.00 | 1.77 | 7.34 | 0.002 |
| Os.11702.1.S1_at | 0.01 | 1.76 | 1.34 | 0.027 |
| Os.7497.1.S1_at | 0.11 | 1.76 | 1.12 | 0.042 |
| Os.19084.1.S1_at | 0.27 | 1.76 | 1.22 | 0.048 |
| Os.14949.1.S1_at | 0.00 | 1.76 | 1.73 | 0.035 |
| Os.5160.1.S1_at | 0.00 | 1.76 | 1.32 | 0.000 |
| Os.2698.3.S1_x_at | 0.27 | 1.76 | 1.26 | 0.044 |
| Os.20538.1.S1_at | 0.17 | 1.76 | 1.01 | 0.029 |
| Os.37320.2.S1_at | 0.05 | 1.76 | 1.27 | 0.035 |
| Os.49864.2.S1_x_at | 0.27 | 1.76 | 1.09 | 0.049 |
| Os.53380.1.S1_at | 0.00 | 1.76 | 1.58 | 0.009 |
| Os.23694.1.S1_at | 0.00 | 1.76 | 5.60 | 0.017 |
| Os.7999.1.S1_at | 0.07 | 1.76 | 1.10 | 0.005 |
| OsAffx.16346.1.S1_at | 0.00 | 1.76 | 1.47 | 0.008 |
| Os.45975.1.S1_a_at | 0.17 | 1.76 | 1.08 | 0.031 |
| Os.12020.1.S1_at | 0.03 | 1.76 | 1.10 | 0.011 |
| OsAffx.18792.1.S1_x_at | 0.11 | 1.76 | 1.07 | 0.020 |
| Os.52847.1.S1_at | 0.00 | 1.75 | 1.55 | 0.031 |
| Os.4851.1.S1_at | 0.01 | 1.75 | 1.59 | 0.043 |
| Os.11943.1.S1_s_at | 0.05 | 1.75 | 1.08 | 0.013 |
| Os.5888.1.S1_at | 0.17 | 1.75 | 1.23 | 0.016 |
| Os.116.1.S1_at | 0.00 | 1.75 | 1.44 | 0.016 |
| Os.21457.1.S1_at | 0.01 | 1.75 | 1.52 | 0.045 |
| Os.5780.1.S1_at | 0.00 | 1.75 | 1.78 | 0.030 |
| OsAffx.18836.1.S1_x_at | 0.00 | 1.75 | 5.28 | 0.015 |
| Os.8708.1.S1_at | 0.00 | 1.75 | 1.53 | 0.009 |
| Os.18704.1.S1_a_at | 0.01 | 1.74 | 1.53 | 0.019 |
| Os.37320.1.S1_at | 0.00 | 1.74 | 1.55 | 0.022 |
| Os.24789.1.A1_at | 0.00 | 1.74 | 4.29 | 0.035 |
| Os.51242.1.S1_at | 0.11 | 1.74 | 1.03 | 0.049 |
| Os.5527.1.S1_a_at | 0.02 | 1.74 | 1.11 | 0.003 |
| Os.11078.1.S1_a_at | 0.00 | 1.74 | 4.26 | 0.008 |
| Os.14984.1.S1_a_at | 0.00 | 1.74 | 1.76 | 0.019 |
| OsAffx.12501.1.S1_at | 0.01 | 1.74 | 1.08 | 0.004 |
| Os.31936.1.S1_at | 0.01 | 1.74 | 1.51 | 0.006 |
| Os.46426.1.S1_at | 0.03 | 1.74 | 1.20 | 0.008 |
| Os.4899.1.S1_a_at | 0.02 | 1.74 | 1.23 | 0.023 |
| Os.5656.1.S1_at | 0.00 | 1.73 | 1.34 | 0.032 |
| Os.12669.1.S1_at | 0.07 | 1.73 | 1.12 | 0.041 |
| Os.40106.1.A1_s_at | 0.00 | 1.73 | 9.33 | 0.001 |
| Os.11431.1.S1_at | 0.00 | 1.73 | 2.43 | 0.048 |
| Os.20595.1.S1_a_at | 0.07 | 1.73 | 1.18 | 0.047 |
| Os.4689.1.S1_at | 0.02 | 1.73 | 1.09 | 0.021 |
| Os.35754.1.S1_at | 0.17 | 1.73 | 1.18 | 0.020 |
| Os.52479.1.S1_at | 0.00 | 1.73 | 1.91 | 0.030 |
| Os.16927.1.S1_at | 0.00 | 1.73 | 1.44 | 0.031 |
| Os.34902.1.S1_at | 2.14 | 1.73 | 1.06 | 0.045 |
| Os.7465.1.S1_at | 0.03 | 1.72 | 1.14 | 0.006 |
| Os.7742.1.S1_a_at | 0.64 | 1.72 | 1.00 | 0.002 |
| Os.54123.1.S1_at | 0.00 | 1.72 | 2.56 | 0.010 |
| Os.4853.1.S2_at | 0.11 | 1.72 | 1.07 | 0.005 |
| Os.32071.1.S1_at | 0.00 | 1.72 | 1.02 | 0.005 |
| Os.21578.1.S1_at | 0.01 | 1.72 | 1.00 | 0.026 |
| Os.47323.1.S1_at | 0.00 | 1.72 | 4.85 | 0.007 |
| Os.27893.1.S2_at | 0.00 | 1.72 | 1.23 | 0.029 |
| OsAffx.12732.1.S1_s_at | 0.00 | 1.72 | 2.48 | 0.042 |
| Os.57379.1.S1_at | 0.00 | 1.72 | 1.12 | 0.035 |
| Os.24616.1.S1_s_at | 0.00 | 1.72 | 2.02 | 0.029 |
| Os.24797.1.A1_at | 0.01 | 1.72 | 1.18 | 0.005 |
| Os.12788.1.S1_at | 1.46 | 1.72 | 1.26 | 0.035 |
| Os.49861.1.S1_at | 0.00 | 1.72 | 6.55 | 0.033 |
| Os.57063.1.S1_at | 0.00 | 1.72 | 3.15 | 0.010 |
| Os.27745.1.A1_at | 0.05 | 1.72 | 1.21 | 0.049 |
| OsAffx.6884.1.S1_at | 0.11 | 1.72 | 1.27 | 0.017 |
| Os.7816.1.S1_at | 0.00 | 1.72 | 1.32 | 0.041 |
| Os.27664.1.S1_at | 0.01 | 1.72 | 1.12 | 0.050 |
| Os.6219.1.S1_at | 0.00 | 1.71 | 33.70 | 0.006 |
| Os.12967.4.S1_x_at | 3.01 | 1.71 | 1.01 | 0.013 |
| Os.53781.1.S1_at | 0.01 | 1.71 | 1.88 | 0.018 |
| OsAffx.14179.1.S1_s_at | 0.07 | 1.71 | 1.37 | 0.011 |
| OsAffx.3283.1.S1_at | 0.00 | 1.71 | 4.60 | 0.005 |
| Os.24660.2.S1_at | 0.07 | 1.71 | 1.11 | 0.013 |
| Os.32680.1.S1_at | 0.00 | 1.71 | 8.44 | 0.001 |
| Os.14251.1.S2_at | 0.01 | 1.71 | 1.38 | 0.010 |
| Os.19279.1.S1_at | 0.07 | 1.71 | 1.06 | 0.015 |
| Os.38157.1.S1_s_at | 0.01 | 1.71 | 1.28 | 0.030 |
| Os.5292.1.S1_at | 0.98 | 1.71 | 1.00 | 0.039 |
| Os.17666.1.S1_at | 0.00 | 1.71 | 1.41 | 0.001 |
| Os.11802.1.S1_at | 0.11 | 1.71 | 1.11 | 0.037 |
| Os.25486.1.S1_at | 0.00 | 1.71 | 1.00 | 0.025 |
| Os.7170.1.S1_at | 0.00 | 1.71 | 1.17 | 0.004 |
| Os.4951.1.S1_at | 0.07 | 1.71 | 1.02 | 0.005 |
| Os.19862.1.S1_at | 0.00 | 1.71 | 6.09 | 0.030 |
| Os.12333.1.S1_at | 0.98 | 1.71 | 1.05 | 0.039 |
| Os.53618.1.S1_at | 0.00 | 1.71 | 1.74 | 0.001 |
| OsAffx.12962.1.S1_at | 0.27 | 1.71 | 1.80 | 0.014 |
| Os.23245.1.A1_at | 0.00 | 1.71 | 3.82 | 0.018 |
| Os.11096.1.S1_at | 0.00 | 1.71 | 3.43 | 0.039 |
| Os.27708.1.S1_at | 0.00 | 1.71 | 1.08 | 0.031 |
| Os.53894.1.S1_x_at | 0.64 | 1.71 | 1.20 | 0.011 |
| OsAffx.11589.1.S1_x_at | 0.00 | 1.71 | 2.27 | 0.017 |
| Os.17893.1.S1_at | 0.02 | 1.70 | 1.17 | 0.005 |
| Os.5378.1.S1_at | 0.00 | 1.70 | 2.09 | 0.017 |
| Os.14409.1.S1_at | 3.01 | 1.70 | 1.00 | 0.018 |
| Os.8816.1.S1_at | 0.00 | 1.70 | 32.77 | 0.006 |
| Os.23927.1.S1_at | 0.17 | 1.70 | 1.03 | 0.040 |
| Os.53778.1.A1_s_at | 0.41 | 1.70 | 1.26 | 0.038 |
| Os.7317.1.S1_a_at | 0.07 | 1.70 | 1.04 | 0.030 |
| Os.14555.1.S1_at | 0.00 | 1.70 | 2.21 | 0.045 |
| Os.51121.1.S1_x_at | 0.64 | 1.70 | 1.08 | 0.013 |
| Os.14200.2.S1_x_at | 0.98 | 1.70 | 1.01 | 0.001 |
| OsAffx.22013.1.S1_at | 0.41 | 1.69 | 1.05 | 0.017 |
| Os.37570.1.S1_s_at | 0.05 | 1.69 | 1.18 | 0.012 |
| Os.4813.1.S1_at | 0.03 | 1.69 | 1.05 | 0.010 |
| Os.16087.1.S1_at | 0.03 | 1.69 | 1.02 | 0.002 |
| Os.25231.1.S1_x_at | 0.03 | 1.69 | 1.74 | 0.006 |
| Os.16318.1.S1_at | 0.07 | 1.69 | 1.43 | 0.010 |
| Os.18583.1.S1_at | 0.00 | 1.69 | 18.70 | 0.004 |
| Os.9023.1.S1_at | 0.01 | 1.69 | 1.15 | 0.012 |
| Os.35193.1.S1_at | 0.05 | 1.69 | 1.13 | 0.024 |
| Os.46545.1.S1_at | 0.07 | 1.69 | 1.10 | 0.003 |
| Os.27641.1.A1_at | 0.00 | 1.68 | 1.16 | 0.016 |
| OsAffx.12465.1.S1_at | 0.00 | 1.68 | 10.20 | 0.001 |
| OsAffx.16462.1.S1_at | 0.01 | 1.68 | 1.91 | 0.003 |
| Os.32481.1.S1_at | 0.00 | 1.68 | 1.29 | 0.032 |
| Os.11591.1.S1_x_at | 0.03 | 1.68 | 1.35 | 0.042 |
| Os.3381.1.S1_a_at | 0.00 | 1.68 | 1.95 | 0.023 |
| Os.51025.1.S1_at | 0.00 | 1.68 | 3.10 | 0.001 |
| Os.51949.1.S1_at | 0.11 | 1.68 | 1.02 | 0.034 |
| OsAffx.19468.2.S1_at | 0.02 | 1.68 | 1.55 | 0.017 |
| Os.7418.1.S1_a_at | 0.41 | 1.68 | 1.05 | 0.032 |
| Os.11949.1.S1_at | 0.17 | 1.68 | 1.23 | 0.014 |
| Os.46999.1.S1_at | 0.00 | 1.67 | 4.01 | 0.034 |
| OsAffx.29804.1.S1_at | 0.00 | 1.67 | 2.15 | 0.008 |
| Os.16282.1.A1_at | 0.00 | 1.67 | 2.95 | 0.027 |
| Os.54185.1.S1_at | 0.01 | 1.67 | 1.74 | 0.031 |
| Os.18195.1.S1_at | 0.00 | 1.67 | 1.53 | 0.003 |
| Os.46305.1.S1_at | 0.07 | 1.67 | 1.41 | 0.043 |
| Os.23659.1.S1_at | 0.02 | 1.67 | 1.39 | 0.048 |
| Os.23212.1.S1_at | 0.00 | 1.67 | 13.87 | 0.021 |
| Os.17618.1.S1_at | 0.02 | 1.67 | 1.15 | 0.007 |
| Os.6180.1.S1_at | 0.05 | 1.67 | 1.12 | 0.000 |
| OsAffx.27508.126.S1_x_at | 0.00 | 1.67 | 3.75 | 0.004 |
| Os.10156.1.S1_a_at | 0.00 | 1.67 | 1.48 | 0.007 |
| Os.15977.1.S1_at | 0.07 | 1.67 | 1.15 | 0.001 |
| Os.17851.1.S1_at | 0.11 | 1.67 | 1.03 | 0.008 |
| Os.3445.1.S1_at | 0.00 | 1.67 | 1.01 | 0.001 |
| Os.2347.1.S1_a_at | 0.00 | 1.67 | 4.23 | 0.020 |
| Os.54394.1.S1_at | 0.00 | 1.67 | 3.85 | 0.035 |
| Os.17219.2.S1_s_at | 0.00 | 1.66 | 1.88 | 0.001 |
| Os.10043.1.S1_at | 0.00 | 1.66 | 1.82 | 0.003 |
| Os.18333.1.S1_at | 0.00 | 1.66 | 1.05 | 0.009 |
| Os.5342.2.S1_x_at | 0.41 | 1.66 | 1.04 | 0.042 |
| Os.12295.1.S1_at | 0.00 | 1.66 | 6.12 | 0.027 |
| OsAffx.23918.1.S1_x_at | 0.03 | 1.66 | 1.00 | 0.014 |
| Os.48919.1.S1_at | 0.41 | 1.66 | 1.10 | 0.013 |
| Os.49535.1.A1_at | 0.17 | 1.66 | 1.04 | 0.027 |
| Os.25157.1.A1_at | 0.00 | 1.66 | 2.08 | 0.010 |
| Os.32013.1.S1_a_at | 0.01 | 1.66 | 1.05 | 0.020 |
| Os.16079.1.S1_at | 0.00 | 1.66 | 2.01 | 0.021 |
| Os.24642.1.S1_at | 0.64 | 1.66 | 1.04 | 0.028 |
| OsAffx.13800.1.S1_s_at | 2.14 | 1.66 | 1.11 | 0.010 |
| Os.47818.1.A1_at | 0.00 | 1.66 | 1.27 | 0.034 |
| OsAffx.23795.1.S1_at | 0.07 | 1.66 | 1.25 | 0.005 |
| Os.27375.1.A1_at | 0.00 | 1.66 | 1.44 | 0.017 |
| Os.4627.1.S1_x_at | 0.01 | 1.66 | 1.36 | 0.007 |
| Os.51682.1.S1_at | 0.00 | 1.66 | 14.39 | 0.009 |
| Os.9751.1.S1_at | 0.00 | 1.66 | 1.16 | 0.001 |
| Os.31252.1.S1_at | 0.02 | 1.66 | 1.05 | 0.026 |
| Os.14652.1.S1_at | 0.00 | 1.66 | 3.46 | 0.036 |
| Os.32564.1.S2_at | 0.00 | 1.66 | 3.89 | 0.001 |
| Os.51161.2.S1_at | 0.00 | 1.66 | 3.10 | 0.026 |
| Os.35440.1.S1_a_at | 0.01 | 1.66 | 1.04 | 0.028 |
| Os.35685.1.S1_at | 0.00 | 1.66 | 2.03 | 0.010 |
| Os.50217.1.S1_at | 0.00 | 1.65 | 1.85 | 0.043 |
| Os.28012.1.S1_at | 0.00 | 1.65 | 2.26 | 0.049 |
| Os.25060.1.S1_at | 3.01 | 1.65 | 1.02 | 0.024 |
| Os.48959.1.S1_at | 0.00 | 1.65 | 4.80 | 0.015 |
| Os.11567.1.S2_a_at | 0.01 | 1.65 | 1.25 | 0.003 |
| Os.46546.1.S1_at | 0.01 | 1.65 | 1.21 | 0.024 |
| OsAffx.11862.1.S1_at | 0.00 | 1.65 | 2.22 | 0.024 |
| Os.22749.1.S1_at | 0.41 | 1.65 | 1.02 | 0.004 |
| Os.32500.2.A1_at | 0.01 | 1.65 | 1.02 | 0.013 |
| Os.33081.1.S1_at | 0.00 | 1.65 | 3.63 | 0.045 |
| Os.15622.1.S1_at | 2.14 | 1.65 | 1.17 | 0.029 |
| Os.52754.1.S1_x_at | 0.00 | 1.65 | 5.27 | 0.023 |
| Os.51821.1.S1_at | 0.41 | 1.65 | 1.31 | 0.043 |
| Os.50931.1.S1_at | 0.00 | 1.64 | 4.65 | 0.008 |
| Os.49745.1.S1_at | 0.41 | 1.64 | 1.18 | 0.042 |
| Os.46513.1.S1_at | 0.00 | 1.64 | 1.60 | 0.021 |
| OsAffx.12382.1.S1_at | 0.00 | 1.64 | 2.58 | 0.006 |
| Os.12960.1.S1_at | 0.00 | 1.64 | 3.80 | 0.030 |
| Os.27530.1.S1_at | 0.01 | 1.64 | 1.27 | 0.008 |
| Os.11985.2.A1_a_at | 0.11 | 1.64 | 1.10 | 0.032 |
| OsAffx.24075.1.S1_at | 0.00 | 1.64 | 2.14 | 0.001 |
| Os.24984.1.A1_at | 0.00 | 1.64 | 1.93 | 0.013 |
| Os.9033.1.S1_at | 0.00 | 1.64 | 1.12 | 0.001 |
| Os.14558.1.S1_at | 0.03 | 1.64 | 1.32 | 0.008 |
| Os.47349.1.S1_at | 0.03 | 1.64 | 1.17 | 0.004 |
| Os.51175.1.S1_x_at | 0.11 | 1.64 | 1.08 | 0.014 |
| Os.11306.1.S1_at | 0.11 | 1.64 | 1.00 | 0.044 |
| Os.51844.1.S1_at | 0.00 | 1.64 | 1.58 | 0.030 |
| Os.49557.1.S1_at | 0.00 | 1.64 | 3.37 | 0.029 |
| Os.15789.1.S1_at | 0.01 | 1.63 | 1.55 | 0.049 |
| Os.5795.1.S1_at | 0.05 | 1.63 | 1.03 | 0.018 |
| Os.18089.1.S1_at | 3.01 | 1.63 | 1.01 | 0.042 |
| OsAffx.11145.1.S1_s_at | 0.00 | 1.63 | 1.35 | 0.026 |
| Os.5059.1.S1_at | 0.00 | 1.63 | 8.98 | 0.046 |
| Os.8999.3.S1_x_at | 0.27 | 1.63 | 1.44 | 0.017 |
| Os.47919.1.A1_at | 0.00 | 1.63 | 4.71 | 0.008 |
| Os.27881.1.S1_s_at | 1.46 | 1.63 | 1.12 | 0.048 |
| OsAffx.9982.1.S1_at | 0.01 | 1.63 | 1.11 | 0.004 |
| Os.5884.1.S1_at | 0.01 | 1.63 | 1.00 | 0.009 |
| Os.52551.1.S1_at | 0.00 | 1.63 | 1.16 | 0.012 |
| Os.54130.1.S1_at | 0.01 | 1.63 | 2.46 | 0.005 |
| OsAffx.27916.1.S1_at | 0.01 | 1.63 | 2.09 | 0.025 |
| Os.24775.1.A1_s_at | 0.01 | 1.63 | 1.60 | 0.038 |
| Os.20329.1.S1_at | 0.00 | 1.63 | 6.62 | 0.044 |
| Os.4635.1.S1_at | 0.00 | 1.63 | 1.33 | 0.022 |
| Os.8281.1.S1_at | 0.01 | 1.63 | 1.67 | 0.007 |
| Os.9892.1.S1_a_at | 0.00 | 1.62 | 2.94 | 0.021 |
| Os.4887.1.S1_at | 0.17 | 1.62 | 1.24 | 0.041 |
| OsAffx.11640.1.S1_at | 0.00 | 1.62 | 7.06 | 0.022 |
| OsAffx.15187.1.S1_at | 0.01 | 1.62 | 1.95 | 0.017 |
| OsAffx.32175.1.S1_at | 0.01 | 1.62 | 1.09 | 0.011 |
| Os.16709.1.S1_at | 0.98 | 1.62 | 1.25 | 0.032 |
| Os.28216.2.S1_s_at | 0.00 | 1.62 | 4.27 | 0.008 |
| Os.25215.1.A1_at | 0.02 | 1.62 | 1.27 | 0.038 |
| Os.19016.1.S1_at | 0.05 | 1.62 | 1.49 | 0.023 |
| Os.12628.1.S1_at | 0.01 | 1.62 | 1.56 | 0.033 |
| Os.22619.1.S2_at | 0.27 | 1.62 | 1.14 | 0.008 |
| Os.9240.1.S1_at | 0.27 | 1.62 | 1.16 | 0.017 |
| Os.12994.1.S1_at | 0.00 | 1.62 | 1.95 | 0.005 |
| Os.46297.1.S1_at | 0.00 | 1.62 | 3.21 | 0.024 |
| Os.11176.1.S1_at | 0.05 | 1.62 | 1.60 | 0.020 |
| OsAffx.3874.1.S1_s_at | 0.41 | 1.62 | 1.29 | 0.041 |
| Os.26508.2.S1_a_at | 0.01 | 1.62 | 1.16 | 0.007 |
| Os.24324.2.S1_at | 0.00 | 1.62 | 3.35 | 0.004 |
| Os.7504.2.S1_at | 0.64 | 1.61 | 1.23 | 0.050 |
| Os.27911.1.S1_at | 0.00 | 1.61 | 2.85 | 0.011 |
| Os.14955.1.S1_at | 2.14 | 1.61 | 1.09 | 0.042 |
| OsAffx.26288.1.S1_at | 0.27 | 1.61 | 1.20 | 0.024 |
| Os.11316.1.S1_at | 0.00 | 1.61 | 1.47 | 0.034 |
| Os.18469.1.S1_at | 0.01 | 1.61 | 1.38 | 0.023 |
| Os.45934.3.S1_x_at | 0.00 | 1.61 | 2.03 | 0.035 |
| Os.47392.1.A1_x_at | 0.27 | 1.61 | 1.00 | 0.022 |
| OsAffx.13048.1.S1_at | 0.07 | 1.61 | 1.13 | 0.016 |
| Os.53732.1.S1_a_at | 0.01 | 1.61 | 1.33 | 0.009 |
| Os.8207.1.S1_at | 0.00 | 1.61 | 2.08 | 0.009 |
| Os.47660.1.S1_s_at | 0.07 | 1.61 | 1.25 | 0.035 |
| Os.27778.3.S1_x_at | 0.27 | 1.61 | 1.04 | 0.023 |
| Os.26502.1.S1_a_at | 0.00 | 1.61 | 1.31 | 0.015 |
| Os.7575.1.S1_at | 0.00 | 1.61 | 1.15 | 0.023 |
| Os.46456.1.S1_at | 0.00 | 1.60 | 1.11 | 0.004 |
| Os.7285.1.S1_at | 0.98 | 1.60 | 1.03 | 0.034 |
| OsAffx.4083.1.S1_at | 0.00 | 1.60 | 1.06 | 0.017 |
| Os.32625.1.S1_s_at | 0.02 | 1.60 | 1.06 | 0.023 |
| Os.26579.1.S1_at | 0.00 | 1.60 | 1.49 | 0.033 |
| Os.12653.1.S1_at | 0.07 | 1.60 | 1.04 | 0.001 |
| Os.50286.1.S1_at | 0.41 | 1.60 | 1.05 | 0.016 |
| Os.53379.1.S1_at | 0.00 | 1.60 | 1.02 | 0.016 |
| Os.51135.1.S1_at | 0.05 | 1.60 | 1.50 | 0.025 |
| OsAffx.24895.1.S1_at | 0.41 | 1.60 | 1.02 | 0.000 |
| Os.12658.1.S1_at | 0.00 | 1.60 | 1.65 | 0.014 |
| Os.31693.4.S1_x_at | 0.27 | 1.60 | 1.21 | 0.049 |
| Os.2431.1.S1_at | 3.01 | 1.60 | 1.26 | 0.037 |
| Os.15219.1.S1_at | 0.00 | 1.60 | 3.08 | 0.028 |
| Os.9782.1.S1_at | 0.00 | 1.60 | 4.98 | 0.027 |
| Os.19179.1.S1_at | 0.64 | 1.60 | 1.02 | 0.006 |
| Os.7316.1.S1_at | 0.00 | 1.60 | 13.90 | 0.001 |
| Os.19541.1.S1_at | 0.00 | 1.60 | 2.32 | 0.017 |
| Os.12724.1.S1_a_at | 0.00 | 1.60 | 2.69 | 0.015 |
| Os.35219.1.S1_a_at | 0.01 | 1.60 | 1.05 | 0.015 |
| Os.52927.1.S1_at | 0.01 | 1.59 | 2.07 | 0.000 |
| Os.15458.1.S1_at | 0.00 | 1.59 | 1.47 | 0.030 |
| Os.51953.1.S1_at | 0.17 | 1.59 | 1.20 | 0.020 |
| Os.151.2.S1_at | 0.00 | 1.59 | 1.54 | 0.036 |
| Os.7890.2.S1_x_at | 0.00 | 1.59 | 5.78 | 0.035 |
| OsAffx.14620.1.S1_at | 0.01 | 1.59 | 1.13 | 0.011 |
| Os.11010.1.S1_at | 0.00 | 1.59 | 1.15 | 0.003 |
| Os.17391.1.S1_at | 0.00 | 1.59 | 1.35 | 0.029 |
| Os.14326.1.S1_at | 1.46 | 1.59 | 1.04 | 0.030 |
| Os.33983.1.S1_at | 0.00 | 1.59 | 15.92 | 0.029 |
| Os.5660.1.S1_at | 2.14 | 1.59 | 1.24 | 0.035 |
| OsAffx.27316.1.S1_at | 0.00 | 1.59 | 3.95 | 0.002 |
| OsAffx.26381.1.S1_at | 1.46 | 1.59 | 1.10 | 0.045 |
| Os.34341.1.S1_at | 0.05 | 1.59 | 1.12 | 0.036 |
| Os.2601.1.S1_at | 0.07 | 1.59 | 1.09 | 0.021 |
| Os.55062.1.S1_at | 0.00 | 1.59 | 1.29 | 0.008 |
| Os.50833.1.S2_at | 0.03 | 1.59 | 1.04 | 0.001 |
| Os.23677.1.S1_at | 0.41 | 1.59 | 1.43 | 0.034 |
| Os.55135.1.S1_at | 0.02 | 1.59 | 1.39 | 0.019 |
| Os.53590.1.S1_at | 0.05 | 1.59 | 1.19 | 0.004 |
| Os.53455.1.S1_at | 0.00 | 1.58 | 2.11 | 0.005 |
| Os.24603.1.A1_at | 0.00 | 1.58 | 4.15 | 0.019 |
| Os.26696.1.S1_at | 0.01 | 1.58 | 1.23 | 0.033 |
| Os.53622.1.S1_at | 0.00 | 1.58 | 1.23 | 0.030 |
| OsAffx.3080.1.S1_at | 0.00 | 1.58 | 1.46 | 0.013 |
| Os.14983.1.S2_a_at | 0.00 | 1.58 | 1.80 | 0.023 |
| Os.8764.1.A1_a_at | 0.00 | 1.58 | 2.30 | 0.011 |
| Os.53537.1.S1_at | 0.00 | 1.58 | 2.00 | 0.038 |
| Os.36729.1.A1_at | 0.05 | 1.58 | 1.12 | 0.015 |
| Os.15559.1.A1_at | 0.00 | 1.58 | 2.05 | 0.003 |
| OsAffx.14221.1.S1_x_at | 0.27 | 1.58 | 1.29 | 0.032 |
| Os.27508.1.S1_a_at | 0.41 | 1.57 | 1.20 | 0.010 |
| Os.47087.1.S1_at | 0.01 | 1.57 | 1.25 | 0.044 |
| OsAffx.12351.1.S1_at | 0.05 | 1.57 | 1.09 | 0.024 |
| Os.9023.1.S1_x_at | 0.05 | 1.57 | 1.14 | 0.008 |
| Os.19048.1.S1_s_at | 0.01 | 1.57 | 1.05 | 0.049 |
| Os.18412.1.S1_a_at | 0.17 | 1.57 | 1.01 | 0.046 |
| Os.2517.1.S1_a_at | 0.05 | 1.57 | 1.01 | 0.041 |
| OsAffx.23268.1.S1_at | 2.14 | 1.57 | 1.00 | 0.004 |
| Os.9476.1.S1_at | 0.01 | 1.57 | 1.19 | 0.033 |
| Os.25542.1.S1_at | 0.07 | 1.57 | 1.08 | 0.050 |
| Os.51837.1.S1_at | 0.00 | 1.57 | 2.87 | 0.024 |
| Os.51650.1.S1_at | 0.00 | 1.57 | 6.87 | 0.028 |
| Os.9834.1.S1_a_at | 0.07 | 1.57 | 1.06 | 0.026 |
| Os.51112.1.S1_at | 0.00 | 1.57 | 1.63 | 0.037 |
| Os.12078.1.S1_at | 1.46 | 1.57 | 1.21 | 0.048 |
| Os.7967.1.S1_at | 0.00 | 1.57 | 11.84 | 0.006 |
| Os.10810.1.S1_at | 0.01 | 1.57 | 1.17 | 0.020 |
| OsAffx.18767.1.S1_at | 0.00 | 1.57 | 13.87 | 0.010 |
| Os.3377.1.S1_at | 0.03 | 1.57 | 1.07 | 0.015 |
| Os.12691.1.A1_x_at | 0.98 | 1.56 | 1.01 | 0.030 |
| Os.53632.1.S1_at | 3.01 | 1.56 | 1.09 | 0.025 |
| Os.49738.1.S1_at | 0.05 | 1.56 | 1.46 | 0.032 |
| Os.7402.1.S1_at | 0.00 | 1.56 | 2.55 | 0.005 |
| Os.14445.1.S1_at | 0.98 | 1.56 | 1.23 | 0.029 |
| Os.4742.1.S2_at | 0.00 | 1.56 | 1.11 | 0.013 |
| OsAffx.31629.1.S1_at | 0.17 | 1.56 | 1.07 | 0.036 |
| Os.51948.1.S1_at | 0.00 | 1.56 | 2.09 | 0.033 |
| Os.10686.1.S1_at | 0.03 | 1.56 | 1.21 | 0.011 |
| Os.46398.1.S1_s_at | 0.00 | 1.56 | 1.10 | 0.013 |
| Os.423.1.S1_a_at | 0.00 | 1.56 | 1.53 | 0.023 |
| Os.49214.1.S1_at | 0.00 | 1.56 | 3.84 | 0.012 |
| Os.54247.1.S1_at | 0.00 | 1.56 | 2.96 | 0.003 |
| Os.7890.1.S1_x_at | 0.00 | 1.56 | 4.38 | 0.034 |
| Os.52477.1.S1_at | 0.00 | 1.56 | 1.47 | 0.034 |
| Os.5347.1.S1_x_at | 0.01 | 1.56 | 2.38 | 0.040 |
| Os.5008.1.S1_at | 0.01 | 1.56 | 1.05 | 0.027 |
| Os.49780.1.S1_x_at | 0.01 | 1.56 | 1.07 | 0.002 |
| OsAffx.12064.1.S1_at | 0.03 | 1.56 | 1.87 | 0.013 |
| Os.26779.1.S1_at | 0.02 | 1.56 | 2.24 | 0.039 |
| Os.18308.1.S1_at | 0.01 | 1.56 | 1.18 | 0.024 |
| Os.32569.1.S1_at | 0.00 | 1.56 | 2.11 | 0.047 |
| Os.17444.1.S1_a_at | 0.01 | 1.55 | 1.57 | 0.029 |
| Os.20211.1.S1_at | 0.00 | 1.55 | 2.53 | 0.014 |
| Os.10726.1.S1_a_at | 0.02 | 1.55 | 1.45 | 0.042 |
| Os.12592.1.S1_at | 0.64 | 1.55 | 1.06 | 0.034 |
| Os.48553.2.S1_x_at | 0.41 | 1.55 | 1.06 | 0.047 |
| Os.12967.1.A1_a_at | 0.07 | 1.55 | 1.05 | 0.041 |
| Os.28403.1.S1_a_at | 0.00 | 1.55 | 4.59 | 0.006 |
| Os.18577.1.S1_at | 0.00 | 1.55 | 11.52 | 0.017 |
| OsAffx.543.1.S1_at | 0.11 | 1.55 | 1.01 | 0.002 |
| Os.30681.1.S1_at | 0.03 | 1.55 | 1.30 | 0.025 |
| Os.25378.1.S1_at | 0.11 | 1.55 | 1.00 | 0.006 |
| Os.38167.1.S1_at | 0.00 | 1.55 | 2.20 | 0.026 |
| Os.22658.1.S1_at | 0.01 | 1.54 | 1.41 | 0.029 |
| Os.50597.1.S1_at | 0.01 | 1.54 | 1.10 | 0.041 |
| Os.49493.1.S1_at | 0.27 | 1.54 | 1.11 | 0.027 |
| Os.10504.1.S1_at | 0.00 | 1.54 | 1.12 | 0.047 |
| Os.27424.1.S1_at | 0.98 | 1.54 | 1.33 | 0.002 |
| Os.9205.1.S1_x_at | 0.00 | 1.54 | 1.28 | 0.008 |
| Os.14690.1.S1_at | 0.11 | 1.54 | 1.07 | 0.027 |
| Os.6080.1.S1_at | 0.03 | 1.54 | 1.06 | 0.013 |
| OsAffx.7660.1.S1_at | 0.00 | 1.54 | 1.91 | 0.039 |
| Os.54640.1.S1_at | 0.00 | 1.54 | 4.71 | 0.005 |
| Os.52749.1.S1_at | 0.02 | 1.54 | 2.71 | 0.043 |
| Os.17777.1.S1_at | 0.98 | 1.54 | 1.05 | 0.043 |
| Os.53870.1.A1_at | 0.01 | 1.54 | 1.54 | 0.046 |
| Os.27278.1.S1_s_at | 0.27 | 1.54 | 1.17 | 0.037 |
| Os.7243.1.S1_s_at | 0.00 | 1.54 | 1.02 | 0.016 |
| Os.36264.1.S1_x_at | 0.00 | 1.54 | 15.39 | 0.007 |
| Os.8150.1.S1_at | 0.05 | 1.54 | 1.10 | 0.036 |
| Os.50790.1.S1_at | 0.00 | 1.54 | 42.85 | 0.015 |
| Os.38092.1.S1_at | 0.00 | 1.54 | 1.54 | 0.002 |
| Os.5845.1.S1_at | 0.11 | 1.54 | 1.11 | 0.015 |
| Os.26476.1.S1_at | 0.03 | 1.53 | 1.27 | 0.048 |
| Os.10274.3.S1_at | 0.41 | 1.53 | 1.00 | 0.046 |
| Os.7915.1.S1_a_at | 0.00 | 1.53 | 3.11 | 0.007 |
| Os.52203.1.S1_at | 0.00 | 1.53 | 1.35 | 0.017 |
| OsAffx.16533.1.S1_at | 0.00 | 1.53 | 1.76 | 0.020 |
| Os.4459.2.S1_x_at | 0.02 | 1.53 | 1.16 | 0.004 |
| Os.47356.1.A1_at | 0.00 | 1.53 | 4.85 | 0.024 |
| Os.27785.1.A1_at | 0.01 | 1.53 | 1.84 | 0.022 |
| Os.10274.1.S1_a_at | 0.05 | 1.53 | 1.20 | 0.001 |
| Os.53073.1.S1_at | 0.00 | 1.53 | 1.28 | 0.005 |
| Os.151.1.S1_x_at | 0.00 | 1.53 | 1.28 | 0.013 |
| OsAffx.24929.1.S1_at | 0.00 | 1.53 | 1.83 | 0.035 |
| Os.48829.1.A1_at | 0.00 | 1.53 | 1.66 | 0.023 |
| Os.5417.3.S1_x_at | 0.07 | 1.53 | 1.16 | 0.050 |
| Os.50904.1.S1_at | 0.00 | 1.53 | 1.27 | 0.005 |
| Os.22617.1.S1_at | 0.01 | 1.53 | 1.17 | 0.006 |
| Os.7167.1.S1_at | 0.00 | 1.53 | 1.43 | 0.027 |
| Os.26702.1.A1_s_at | 0.00 | 1.53 | 1.31 | 0.013 |
| Os.49593.1.S1_x_at | 0.00 | 1.53 | 1.49 | 0.005 |
| Os.8323.1.S1_a_at | 0.41 | 1.53 | 1.07 | 0.042 |
| Os.49732.1.S1_at | 0.01 | 1.52 | 1.19 | 0.036 |
| Os.22281.1.S1_at | 0.17 | 1.52 | 1.60 | 0.011 |
| Os.8773.1.S1_a_at | 0.00 | 1.52 | 1.18 | 0.002 |
| OsAffx.7241.1.S1_at | 0.00 | 1.52 | 1.58 | 0.028 |
| Os.5799.1.S1_s_at | 0.00 | 1.52 | 1.72 | 0.034 |
| Os.51846.1.S1_x_at | 0.98 | 1.52 | 1.05 | 0.010 |
| Os.55991.1.S1_at | 0.05 | 1.52 | 1.05 | 0.022 |
| Os.27778.2.S1_at | 0.07 | 1.52 | 1.22 | 0.004 |
| OsAffx.25768.1.S1_x_at | 0.00 | 1.52 | 3.01 | 0.039 |
| Os.48917.1.A1_at | 0.00 | 1.52 | 4.37 | 0.048 |
| OsAffx.25081.1.S1_at | 0.11 | 1.52 | 1.07 | 0.028 |
| Os.22812.1.S1_a_at | 0.01 | 1.52 | 1.60 | 0.033 |
| Os.12701.1.S2_at | 0.00 | 1.52 | 1.02 | 0.021 |
| Os.23153.1.S1_s_at | 0.00 | 1.52 | 6.62 | 0.018 |
| Os.8946.1.S1_at | 0.01 | 1.52 | 1.20 | 0.008 |
| Os.54379.1.S1_at | 0.01 | 1.52 | 1.50 | 0.018 |
| Os.37673.1.S1_at | 0.11 | 1.52 | 1.02 | 0.003 |
| Os.54112.1.S1_at | 0.27 | 1.52 | 1.28 | 0.006 |
| OsAffx.7922.1.S1_at | 0.01 | 1.52 | 1.39 | 0.050 |
| Os.22569.1.S1_at | 0.00 | 1.52 | 1.15 | 0.025 |
| OsAffx.14597.1.S1_at | 0.00 | 1.52 | 3.48 | 0.013 |
| Os.5869.1.S1_a_at | 0.00 | 1.52 | 3.20 | 0.024 |
| Os.554.1.S1_at | 0.00 | 1.52 | 11.54 | 0.023 |
| OsAffx.30188.1.S1_s_at | 0.03 | 1.52 | 1.04 | 0.005 |
| Os.28814.2.S1_x_at | 0.01 | 1.51 | 1.09 | 0.046 |
| Os.49596.1.S1_at | 0.64 | 1.51 | 1.38 | 0.013 |
| Os.16530.1.S1_at | 0.98 | 1.51 | 1.02 | 0.049 |
| Os.49281.1.S1_s_at | 0.07 | 1.51 | 2.79 | 0.049 |
| Os.35377.1.S1_a_at | 0.00 | 1.51 | 1.99 | 0.012 |
| Os.54305.1.S1_at | 0.00 | 1.51 | 2.46 | 0.046 |
| OsAffx.2017.1.S1_s_at | 0.00 | 1.51 | 2.28 | 0.035 |
| Os.56014.1.S1_at | 0.01 | 1.51 | 1.85 | 0.044 |
| Os.54092.1.S1_at | 0.00 | 1.51 | 2.00 | 0.012 |
| Os.9596.1.S1_at | 0.05 | 1.51 | 1.17 | 0.005 |
| Os.27768.1.S1_at | 0.00 | 1.51 | 1.17 | 0.018 |
| Os.54657.1.S1_at | 0.07 | 1.51 | 1.06 | 0.039 |
| Os.12049.1.S1_at | 0.11 | 1.51 | 1.35 | 0.030 |
| Os.42377.1.S1_x_at | 0.05 | 1.51 | 1.09 | 0.003 |
| Os.47919.1.A1_s_at | 0.00 | 1.51 | 7.41 | 0.017 |
| Os.48636.1.S1_at | 0.00 | 1.51 | 1.00 | 0.018 |
| OsAffx.21707.2.S1_x_at | 0.00 | 1.51 | 2.87 | 0.020 |
| Os.16867.1.S1_at | 0.05 | 1.51 | 1.40 | 0.026 |
| Os.34512.1.S1_at | 0.17 | 1.51 | 1.11 | 0.025 |
| OsAffx.30015.1.S1_at | 0.00 | 1.51 | 1.21 | 0.041 |
| Os.18149.2.S1_at | 0.02 | 1.50 | 1.86 | 0.045 |
| Os.54006.1.S1_at | 0.05 | 1.50 | 1.01 | 0.038 |
| Os.24947.1.S1_at | 0.00 | 1.50 | 2.88 | 0.022 |
| Os.47700.1.A1_at | 0.00 | 1.50 | 5.11 | 0.002 |
| Os.54315.1.S1_at | 0.00 | 1.50 | 12.96 | 0.006 |
| Os.35567.1.S1_at | 0.17 | 1.50 | 1.12 | 0.007 |
| Os.11689.1.S1_at | 0.03 | 1.50 | 1.08 | 0.009 |
| Os.2708.3.S1_at | 0.00 | 1.50 | 1.47 | 0.004 |

Note:

a. Ratio1/2 = signal1(avg)/signal2(avg) from Wilcoxon Rank-Sum tests

b. Ratio2/3 = signal2(avg)/signal3(avg) from Wilcoxon Rank-Sum tests

c. The probability associated with the *t*-tests.
