# Supplementary material for: Identification of rhizome-specific genes by genome-wide differential expression Analysis in Oryza longistaminata
Source: BMC Plant Biol. 2011 Jan 24;11:18. doi: 10.1186/1471-2229-11-18 (PMC3036607; doi:10.1186/1471-2229-11-18)
Supplement: Additional file 3 — The list of 61 genes specifically enriched in the rhizome internodes (RI) of O. longistaminata and their annotated functions detected by the Affymetrix GeneChip Rice Genome Array. Word file for the list of genes enriched in the rhizome internode of Oryza longistaminata and their function annotation. [file 1471-2229-11-18-S3.DOC]

**Additional file 3**. The list of 61 genes specifically enriched in the rhizome internodes (RI) of *O. longistaminata* and their annotated functions detected by the Affymetrix GeneChip Rice Genome Array

| **Name** | **Ratio*** | ***p* value** | **OsGI** | **Function Annotation (NCBI)** |
| --- | --- | --- | --- | --- |
| Os.10240.1.S1 | 1.95 | 0.0221 | LOC_Os01g65680 | Catalytic LigB subunit of aromatic ring-opening dioxygenase family protein, expressed |
| Os.11166.1.S1_s | 1.88 | 0.0282 | LOC_Os01g09830 | Glutaredoxin-like family protein, expressed |
| Os.11313.1.S1 | 3.09 | 0.0453 | LOC_Os03g18850 | Pathogenesis-related protein 1 |
| Os.13844.1.S1 | 2.70 | 0.0482 | LOC_Os06g04200 | unspliced-genomic starch synthase, putative,expressed |
| Os.15138.1.S1 | 2.56 | 0.0197 | LOC_Os01g41900 | MCB2 protein, putative, expressed |
| Os.15717.1.S1 | 2.06 | 0.0464 | Os08g0566900 | Mpv17/PMP22 family protein. |
| Os.16018.1.S1 | 1.98 | 0.0137 | LOC_Os01g63010 | ENOD18 protein, putative, expressed |
| Os.16121.1.S1 | 4.17 | 0.0400 | Os01g0327400 | Peroxidase 1. |
| Os.16582.1.S1 | 3.93 | 0.0071 | LOC_Os07g42910 | Cytochrome c oxidase subunit, putative, expressed |
| Os.172.1.S1_a | 7.88 | 0.0380 | LOC_Os11g47500 | Xylanase inhibitor protein 1 precursor, putative, expressed |
| Os.22967.1.S1_s | 2.10 | 0.0492 | LOC_Os03g02070 | heavy-metal-associated domain-containing protein, putative, expressed |
| Os.2362.2.S1_x | 2.72 | 0.0126 | LOC_Os02g35770 | Homeobox domain containing protein, expressed |
| Os.23792.2.S1_x | 2.60 | 0.0111 | LOC_Os08g06450 | Cytidylyltransferase family protein, expressed |
| Os.25202.1.S1 | 3.51 | 0.0249 | LOC_Os05g32710 | isoamylase-type starch debranching enzyme ISO2, putative, expressed |
| Os.25449.1.S1 | 2.29 | 0.0040 | LOC_Os07g24230 | folate/biopterin transporter family protein, expressed |
| Os.26502.1.S1_a | 1.61 | 0.0147 | LOC_Os04g35520 | L-ascorbate peroxidase 7, chloroplast precursor, putative, expressed |
| Os.27967.1.A1 | 3.90 | 0.0428 | LOC_Os01g63620 | expressed protein |
| Os.28433.4.A1 | 9.03 | 0.0385 | LOC_Os01g58290 | Subtilisin N-terminal Region family protein |
| Os.35005.1.S1_x | 1.88 | 0.0357 | Os01g0149800 | Metallothionein-like protein type 2 |
| Os.36346.2.S1 | 3.51 | 0.0137 | LOC_Os01g67030 | membrane protein, putative, expressed |
| Os.37603.1.S1 | 2.70 | 0.0006 | LOC_Os01g63480 | AER, putative, expressed |
| Os.37718.1.S1 | 36.00 | 0.0200 | LOC_Os10g26940 | BURP domain containing protein, expressed |
| Os.41841.1.S1 | 1.77 | 0.0004 | LOC_Os01g52130 | sulfate transporter 3.5, putative, expressed |
| Os.46398.1.S1_s | 1.56 | 0.0129 | LOC_Os01g05650 | Metallothionein-like protein type 2, putative |
| Os.46728.1.S1 | 2.83 | 0.0450 | LOC_Os10g04800 | hypothetical protein |
| Os.46869.2.S1_x | 4.80 | 0.0098 | LOC_Os11g42390 | Serine carboxypeptidase family protein |
| Os.47946.1.S1_s | 2.10 | 0.0404 | LOC_Os07g33910 | Glucose-6-phosphate/phosphate translocator 2, chloroplast precursor, putative, expressed |
| Os.47949.1.S1_a | 1.98 | 0.0002 | LOC_Os05g51470 | expressed protein |
| Os.48074.1.A1 | 2.68 | 0.0397 | LOC_Os05g19910 | Transferase family protein, expressed |
| Os.49099.1.S1 | 2.26 | 0.0327 | LOC_Os03g63760 | xyloglucan endotransglucosylase/hydrolase protein 28 precursor, putative, expressed |
| Os.49151.1.S1 | 1.86 | 0.0373 | LOC_Os11g44800 | expressed protein |
| Os.49281.1.S1_s | 1.51 | 0.0487 | LOC_Os06g21240 | Glycine rich protein family protein, expressed |
| Os.49795.1.S1 | 2.78 | 0.0290 | LOC_Os07g41580 | CCAAT-binding transcription factor subunit A, putative, expressed |
| Os.4999.1.S1 | 2.23 | 0.0357 | Os06g0716100 | Heat shock protein DnaJ family protein. |
| Os.50583.1.S1 | 1.98 | 0.0195 | LOC_Os03g60260 | Transmembrane amino acid transporter protein |
| Os.52574.1.S1 | 3.21 | 0.0086 | LOC_Os04g41450 | POT family protein, expressed |
| Os.52981.1.S1 | 1.80 | 0.0335 | LOC_Os11g28910 | transposon protein, putative, CACTA, En/Spm sub-class, expressed |
| Os.53214.1.S1 | 1.91 | 0.0270 | Os01g0223000 | Lipolytic enzyme, G-D-S-L family protein. |
| Os.53622.1.S1 | 1.58 | 0.0301 | LOC_Os04g44100 | expressed protein |
| Os.53698.1.S1 | 2.04 | 0.0009 | LOC_Os11g43420 | NB-ARC domain containing protein, expressed |
| Os.54620.1.A1 | 2.40 | 0.0239 | LOC_Os12g38810 | expressed protein |
| Os.55527.1.S1 | 2.09 | 0.0252 | LOC_Os08g31340 | heavy metal-associated domain containing protein, expressed |
| Os.55532.1.S1 | 5.31 | 0.0169 | LOC_Os03g02300 | expressed protein |
| Os.5616.1.S1 | 2.20 | 0.0213 | LOC_Os03g18740 | Sex determination protein tasselseed 2 |
| Os.5780.1.S1 | 1.75 | 0.0296 | LOC_Os08g43290 | Protease inhibitor/seed storage/LTP family protein, expressed |
| Os.6764.2.S1 | 4.22 | 0.0026 | Os04g0635400 | Conserved hypothetical protein |
| Os.7631.1.S1 | 3.10 | 0.0191 | Os08g0476300 | Short-chain dehydrogenase/reductase SDR family protein. |
| Os.7705.1.S1 | 7.03 | 0.0015 | LOC_Os04g54300 | wound induced protein, putative, expressed |
| Os.7756.2.S1_x | 3.07 | 0.0464 | LOC_Os10g36500 | pectinesterase inhibitor domain containing protein, expressed |
| Os.8508.1.S1 | 2.53 | 0.0142 | LOC_Os10g38880 | uncharacterized plant-specific domain TIGR01568 family protein, expressed |
| Os.8999.3.S1_x | 1.63 | 0.0167 | LOC_Os01g67860 | Fructose-bisphosphate aldolase, cytoplasmic isozyme, putative, expressed |
| Os.9828.1.S1 | 7.97 | 0.0234 | LOC_Os07g44920 | Dirigent-like protein |
| Os.9913.1.S1 | 6.59 | 0.0040 | LOC_Os04g54230 | expressed protein |
| OsAffx.14273.1.S1 | 2.08 | 0.0153 | LOC_Os04g45130 | heavy-metal-associated domain-containing protein, putative, expressed |
| OsAffx.14410.1.S1_s | 12.01 | 0.0442 | LOC_Os04g54230 | expressed protein |
| OsAffx.14846.1.S1 | 1.78 | 0.0136 | Os05g0338900 | TGF-beta receptor, type I/II extracellular region family protein. |
| OsAffx.20051.1.S1 | 10.88 | 0.0188 | LOC_Os12g40330 | expressed protein |
| OsAffx.30015.1.S1 | 1.51 | 0.0411 | LOC_Os09g25420 | Zinc finger, C2H2 type family protein, expressed |
| OsAffx.30737.1.S1 | 2.26 | 0.0293 | LOC_Os10g40610 | Flavin-binding monooxygenase-like family protein, expressed |
| OsAffx.31482.1.S1_s | 2.04 | 0.0488 | LOC_Os11g44940 | seed maturation protein PM27, putative, expressed |
| OsAffx.32175.1.S1 | 1.62 | 0.0109 | LOC_Os12g43620 | Helix-loop-helix DNA-binding domain containing protein |

* Ratio indicates signal1(avg)/signal2(avg) from Wilcoxon Rank-Sum tests

*P* is the probability associated with the *t*-tests.
