# Supplementary material for: Identification of rhizome-specific genes by genome-wide differential expression Analysis in Oryza longistaminata
Source: BMC Plant Biol. 2011 Jan 24;11:18. doi: 10.1186/1471-2229-11-18 (PMC3036607; doi:10.1186/1471-2229-11-18)
Supplement: Additional file 4 — The list of 299 genes specifically-enriched in the shoot tips (ST) of O. longistaminata and their annotated functions (TIGR) detected the Affymetrix GeneChip Rice Genome Array. Word file for the list of genes enriched in the shoot tip of Oryza longistaminata and their function annotation. [file 1471-2229-11-18-S4.DOC]

**Additional file 4**. The list of 299 genes specifically-enriched in the shoot tips (ST) of *O. longistaminata* and their annotated functions (TIGR) detected by the Affymetrix GeneChip Rice Genome Array

| **Name** | **Ratio_1/2** | ***p* value** | **OsGI** | **Function Annotation** |
| --- | --- | --- | --- | --- |
| Os.10043.1.S1_at | 1.66 | 0.0027 | LOC_Os11g38920 | TB2/DP1, HVA22 family protein |
| Os.10452.1.S1_at | 1.97 | 0.0126 | LOC_Os03g13720 | expressed protein |
| Os.10548.1.S1_s_at | 1.87 | 0.0051 | LOC_Os02g55130 | Serine carboxypeptidase II precursor |
| Os.10726.1.S1_a_at | 1.55 | 0.0418 | LOC_Os06g16430 | FHA domain containing protein |
| Os.10817.1.S1_a_at | 1.80 | 0.0219 | Os11g0523800 | Transcriptional factor B3 family protein. |
| Os.11096.1.S1_at | 1.71 | 0.0388 | LOC_Os05g34240 | expressed protein |
| Os.11176.1.S1_at | 1.62 | 0.0198 | LOC_Os02g57400 | 2-phosphoglycerate kinase |
| Os.11689.1.S1_at | 1.50 | 0.0094 | Os01g0527400 | Bromo adjacent region domain containing protein. |
| Os.12658.1.S1_at | 1.60 | 0.0140 | LOC_Os03g55620 | Serine/threonine-protein kinase 12 |
| Os.12960.1.S1_at | 1.64 | 0.0298 | LOC_Os12g06740 | F-box protein interaction domain containing protein |
| Os.14423.1.S1_x_at | 1.94 | 0.0063 | LOC_Os06g11400 | expressed protein |
| Os.14555.1.S1_at | 1.70 | 0.0448 | LOC_Os10g27990 | exocyst complex component Sec15 |
| Os.14652.1.S1_at | 1.66 | 0.0362 | LOC_Os05g30750 | C2 domain-containing protein |
| Os.14949.1.S1_at | 1.76 | 0.0355 | LOC_Os04g12480 | pumilio/Puf RNA-binding domain-containing protein |
| Os.14983.1.S2_a_at | 1.58 | 0.0227 | LOC_Os05g51790 | structural maintenance of chromosomes family protein |
| Os.15037.1.S2_at | 1.80 | 0.0221 | Os08g0175300 | PolI-like DNA polymerase. |
| Os.15458.1.S1_at | 1.59 | 0.0305 | LOC_Os06g40600 | 116 kDa U5 small nuclear ribonucleoprotein component |
| Os.15559.1.A1_at | 1.58 | 0.0028 | LOC_Os11g45240 | Ribonucleases P/MRP protein subunit POP1 containing protein |
| Os.16079.1.S1_at | 1.66 | 0.0209 | LOC_Os05g06260 | Gamma-tubulin complex component 4 homolog |
| Os.16087.1.S1_at | 1.69 | 0.0024 | LOC_Os02g47940 | Histidinol-phosphate aminotransferase, chloroplast precursor |
| Os.16282.1.A1_at | 1.67 | 0.0265 | LOC_Os06g37080 | L-ascorbate oxidase precursor |
| Os.16326.1.S1_at | 1.83 | 0.0346 | LOC_Os02g17780 | Ent-kaurene synthase A, chloroplast precursor |
| Os.17219.1.S1_at | 1.77 | 0.0141 | LOC_Os11g09960 | hypothetical protein |
| Os.17219.2.S1_s_at | 1.66 | 0.0007 | LOC_Os11g09960 | hypothetical protein |
| Os.17777.1.S1_at | 1.54 | 0.0433 | Os01g0521400 | Conserved hypothetical protein. |
| Os.17841.2.S1_x_at | 2.14 | 0.0303 | LOC_Os07g06940 | Valyl-tRNA synthetase |
| Os.18149.2.S1_at | 1.50 | 0.0451 | LOC_Os04g40740 | Kelch motif family protein |
| Os.18195.1.S1_at | 1.67 | 0.0031 | LOC_Os02g58210 | jmjC domain containing protein |
| Os.18308.1.S1_at | 1.56 | 0.0239 | LOC_Os12g29520 | Auxin response factor 2 |
| Os.18412.1.S1_a_at | 1.57 | 0.0456 | LOC_Os10g27340 | prolyl 4-hydroxylase |
| Os.18459.2.S1_x_at | 2.30 | 0.0027 | LOC_Os01g66590 | AS2 |
| Os.18552.1.S1_at | 2.51 | 0.0030 | LOC_Os11g31980 | Serine carboxypeptidase family protein |
| Os.18572.1.S1_at | 4.10 | 0.0010 | LOC_Os04g52310 | ZIP zinc/iron transport family protein |
| Os.18577.1.S1_at | 1.55 | 0.0174 | Os03g0577100 | Nuf2 family protein. |
| Os.18583.1.S1_at | 1.69 | 0.0041 | LOC_Os01g07260 | expressed protein |
| Os.18590.1.S1_a_at | 1.85 | 0.0014 | LOC_Os06g51310 | PINHEAD protein |
| Os.18704.1.S1_a_at | 1.74 | 0.0191 | LOC_Os02g28830 | TPR Domain containing protein |
| Os.19006.1.S1_a_at | 1.79 | 0.0495 | LOC_Os07g48350 | expressed protein |
| Os.19048.1.S1_s_at | 1.57 | 0.0489 | LOC_Os08g24760 | Pre-mRNA splicing factor RNA helicase |
| Os.19179.1.S1_at | 1.60 | 0.0063 | LOC_Os08g38570 | transducin family protein |
| Os.19541.1.S1_at | 1.60 | 0.0165 | Os12g0178700 | Dynamin-like protein. |
| Os.19844.1.S1_at | 3.11 | 0.0474 | LOC_Os03g57190 | TCP family transcription factor containing protein |
| Os.19862.1.S1_at | 1.71 | 0.0295 | LOC_Os05g41750 | SMC family, C-terminal domain containing protein |
| Os.20211.1.S1_at | 1.55 | 0.0142 | LOC_Os10g36710 | CDPK-related protein kinase |
| Os.20329.1.S1_at | 1.63 | 0.0440 | LOC_Os05g38480 | Kinesin motor domain containing protein |
| Os.2131.1.S1_s_at | 1.81 | 0.0198 | LOC_Os01g54370 | Dihydroorotase, mitochondrial precursor |
| Os.22617.1.S1_at | 1.53 | 0.0063 | LOC_Os05g48860 | Guanylate-binding protein, N-terminal domain containing protein |
| Os.22658.1.S1_at | 1.54 | 0.0289 | LOC_Os01g27740 | DnaJ domain containing protein |
| Os.22796.1.S1_at | 2.17 | 0.0024 | LOC_Os03g47140 | expressed protein |
| Os.2293.1.S1_at | 3.01 | 0.0040 | LOC_Os03g44710 | YABBY protein |
| Os.23153.1.S1_s_at | 1.52 | 0.0176 | LOC_Os09g15170 | xanthine/uracil permease family protein |
| Os.23164.1.S1_a_at | 1.93 | 0.0028 | LOC_Os07g39980 | expressed protein |
| Os.23212.1.S1_at | 1.67 | 0.0212 | LOC_Os12g44090 | leucine-rich repeat family protein |
| Os.23245.1.A1_at | 1.71 | 0.0184 | LOC_Os10g39850 | Zinc finger, C3HC4 type family protein |
| Os.2347.1.S1_a_at | 1.67 | 0.0197 | LOC_Os02g53690 | expressed protein |
| Os.23694.1.S1_at | 1.76 | 0.0170 | LOC_Os01g42060 | Ubiquitin-conjugating enzyme family protein |
| Os.23868.1.S1_at | 1.93 | 0.0344 | LOC_Os01g68870 | Leucine Rich Repeat family protein |
| Os.23927.1.S1_at | 1.70 | 0.0404 | LOC_Os08g40630 | mTERF family protein |
| Os.23940.1.A1_at | 1.86 | 0.0017 | LOC_Os08g01680 | Chromatin assembly factor 1 subunit B |
| Os.24268.1.S1_x_at | 1.84 | 0.0121 | LOC_Os11g06680 | expressed protein |
| Os.24275.1.S2_at | 1.78 | 0.0410 | LOC_Os02g35100 | DDT domain containing protein |
| Os.2431.1.S1_at | 1.60 | 0.0367 | LOC_Os11g10340 | F-box domain containing protein |
| Os.24324.2.S1_at | 1.62 | 0.0044 | LOC_Os03g27030 | Arginine/serine-rich splicing factor |
| Os.24545.2.S1_x_at | 1.91 | 0.0301 | LOC_Os03g30300 | 6-phosphogluconolactonase family protein |
| Os.24603.1.A1_at | 1.58 | 0.0189 | LOC_Os10g42960 | SSS sodium solute transporter superfamily protein |
| Os.24616.1.S1_s_at | 1.72 | 0.0291 | LOC_Os04g46310 | HEAT repeat family protein |
| Os.24775.1.A1_s_at | 1.63 | 0.0375 | LOC_Os01g16220 | expressed protein |
| Os.24789.1.A1_at | 1.74 | 0.0347 | LOC_Os11g10040 | mTERF family protein |
| Os.24797.1.A1_at | 1.72 | 0.0046 | LOC_Os02g55180 | Ubiquitin carboxyl-terminal hydrolase family protein |
| Os.24852.1.A1_at | 1.90 | 0.0321 | LOC_Os01g24940 | Poly synthetase 2 |
| Os.24947.1.S1_at | 1.50 | 0.0222 | LOC_Os04g55620 | Protein kinase domain containing protein |
| Os.24984.1.A1_at | 1.64 | 0.0129 | LOC_Os01g12430 | D-mannose binding lectin family protein |
| Os.25157.1.A1_at | 1.66 | 0.0097 | LOC_Os05g08450 | expressed protein |
| Os.25231.1.S1_x_at | 1.69 | 0.0055 | LOC_Os10g35750 | pentatricopeptide |
| Os.25542.1.S1_at | 1.57 | 0.0497 | Os03g0646800 | RPB140 (Fragment). |
| Os.26779.1.S1_at | 1.56 | 0.0391 | LOC_Os05g07960 | F-box protein interaction domain containing protein |
| Os.26978.1.S1_a_at | 1.96 | 0.0461 | LOC_Os11g40610 | early flowering 4 |
| Os.2708.3.S1_at | 1.50 | 0.0036 | LOC_Os03g37270 | RNA recognition motif family protein |
| Os.27118.1.A1_at | 2.06 | 0.0180 | LOC_Os06g44050 | Methyladenine glycosylase family protein |
| Os.27424.1.S1_at | 1.54 | 0.0015 | LOC_Os05g18660 | F-box domain containing protein |
| Os.27778.2.S1_at | 1.52 | 0.0039 | Os01g0581900 | Hypothetical protein. |
| Os.27778.3.S1_x_at | 1.61 | 0.0229 | LOC_Os01g40000 | expressed protein |
| Os.27785.1.A1_at | 1.53 | 0.0221 | LOC_Os01g74330 | pectinacetylesterase family protein |
| Os.27911.1.S1_at | 1.61 | 0.0115 | LOC_Os01g54210 | GATA zinc finger family protein |
| Os.28012.1.S1_at | 1.65 | 0.0493 | LOC_Os03g57870 | Activator 1 38 kDa subunit |
| Os.28032.1.A1_at | 2.37 | 0.0338 | LOC_Os01g21070 | Endoglucanase 1 precursor |
| Os.28769.1.S2_at | 1.77 | 0.0314 | LOC_Os01g34610 | expressed protein |
| Os.29068.1.S1_at | 4.45 | 0.0123 | LOC_Os03g56200 | expressed protein |
| Os.30681.1.S1_at | 1.55 | 0.0254 | LOC_Os07g03130 | expressed protein |
| Os.31252.1.S1_at | 1.66 | 0.0264 | LOC_Os11g18830 | Survival motor neuron containing protein |
| Os.31545.1.S1_at | 1.77 | 0.0287 | LOC_Os07g46560 | Ubiquitin ligase SINAT5 |
| Os.31936.1.S1_at | 1.74 | 0.0058 | LOC_Os10g05220 | tRNA-guanine transglycosylases, various specificities family protein |
| Os.32455.1.S1_at | 2.01 | 0.0273 | LOC_Os01g54670 | expressed protein |
| Os.32564.1.S2_at | 1.66 | 0.0008 | LOC_Os01g54420 | dynamin family protein |
| Os.32569.1.S1_at | 1.56 | 0.0470 | LOC_Os03g03560 | FHA domain containing protein |
| Os.32618.1.S1_at | 2.75 | 0.0174 | LOC_Os06g02560 | growth-regulating factor |
| Os.32622.1.S1_at | 2.24 | 0.0010 | LOC_Os01g01170 | expressed protein |
| Os.32680.1.S1_at | 1.71 | 0.0005 | LOC_Os07g05190 | leucine-rich repeat family protein |
| Os.3278.1.S1_at | 1.80 | 0.0456 | LOC_Os10g40130 | UDP-N-acetylmuramyl-tripeptide synthetases family protein |
| Os.32970.1.S1_at | 2.05 | 0.0434 | LOC_Os03g37100 | DTW domain containing protein |
| Os.33081.1.S1_at | 1.65 | 0.0453 | LOC_Os03g39740 | expressed protein |
| Os.33127.2.S1_x_at | 2.45 | 0.0488 | LOC_Os01g07940 | Protein kinase domain containing protein |
| Os.33220.1.S1_at | 1.87 | 0.0202 | LOC_Os01g70260 | Protein kinase domain containing protein |
| Os.3354.1.S1_at | 1.78 | 0.0215 | LOC_Os10g25110 | Pumilio-family RNA binding repeat containing protein |
| Os.3381.1.S1_a_at | 1.68 | 0.0228 | LOC_Os05g28510 | Importin-beta N-terminal domain containing protein |
| Os.33894.2.A1_at | 2.14 | 0.0011 | Os07g0534400 | Hypothetical protein. |
| Os.33983.1.S1_at | 1.59 | 0.0291 | LOC_Os01g09790 | IQ calmodulin-binding motif family protein |
| Os.34206.1.S1_at | 3.08 | 0.0270 | Os03g0162000 | Dimethylaniline monooxygenase-like protein (Flavin-containing monooxygenase YUCCA). |
| Os.35377.1.S1_a_at | 1.51 | 0.0115 | LOC_Os02g45600 | rac GTPase activating protein |
| Os.35440.1.S1_a_at | 1.66 | 0.0282 | LOC_Os01g55090 | transposon protein , Mutator sub-class |
| Os.35685.1.S1_at | 1.66 | 0.0098 | LOC_Os01g56510 | Glycosyl hydrolase family 3 N terminal domain containing protein |
| Os.36264.1.S1_x_at | 1.54 | 0.0067 | LOC_Os01g53750 | Glycosyl hydrolases family 17 protein |
| Os.38092.1.S1_at | 1.54 | 0.0018 | LOC_Os03g12320 | UDP-3-0-acyl N-acetylglucosamine deacetylase family protein |
| Os.38110.1.S1_at | 2.04 | 0.0409 | LOC_Os10g37660 | Trehalase family protein |
| Os.38167.1.S1_at | 1.55 | 0.0259 | LOC_Os04g31000 | expressed protein |
| Os.40106.1.A1_s_at | 1.73 | 0.0010 | LOC_Os08g42600 | retinoblastoma |
| Os.423.1.S1_a_at | 1.56 | 0.0228 | LOC_Os02g33430 | E2F protein |
| Os.42377.1.S1_x_at | 1.51 | 0.0025 | LOC_Os01g64280 | sorting nexin 1 |
| Os.44676.1.S1_at | 1.80 | 0.0194 | LOC_Os07g37640 | IKI3 family protein |
| Os.45934.3.S1_x_at | 1.61 | 0.0347 | LOC_Os01g09760 | DIRP family protein |
| Os.45953.1.S1_at | 1.85 | 0.0130 | LOC_Os01g39600 | Leucine Rich Repeat family protein |
| Os.46297.1.S1_at | 1.62 | 0.0244 | LOC_Os07g33360 | chromosoma associate protein subunit H2 |
| Os.46397.1.S1_x_at | 6.07 | 0.0147 | LOC_Os10g35490 | epoxide hydrolase |
| Os.46426.1.S1_at | 1.74 | 0.0082 | LOC_Os10g03910 | F-box domain containing protein |
| Os.46456.1.S1_at | 1.60 | 0.0036 | LOC_Os10g29310 | BTB/POZ domain containing protein |
| Os.46513.1.S1_at | 1.64 | 0.0213 | LOC_Os10g34580 | FHA domain containing protein |
| Os.46919.1.S1_at | 1.90 | 0.0096 | LOC_Os05g39880 | CBL-interacting serine/threonine-protein kinase 15 |
| Os.46988.1.A1_at | 2.37 | 0.0286 | LOC_Os07g45250 | transposon protein , Mutator sub-class |
| Os.46999.1.S1_at | 1.67 | 0.0339 | LOC_Os10g42520 | expressed protein |
| Os.47322.1.A1_at | 2.00 | 0.0023 | LOC_Os09g30380 | expressed protein |
| Os.47368.1.S1_x_at | 1.85 | 0.0161 | LOC_Os07g46540 | HEAT repeat family protein |
| Os.4742.1.S2_at | 1.56 | 0.0133 | LOC_Os07g04530 | GYF domain containing protein |
| Os.47700.1.A1_at | 1.50 | 0.0019 | Os05g0394200 | Protein of unknown function DUF630 domain containing protein. |
| Os.47853.1.A1_at | 1.79 | 0.0353 | LOC_Os05g49680 | F-box domain containing protein |
| Os.47919.1.A1_at | 1.63 | 0.0080 | LOC_Os05g33030 | Kinesin motor domain containing protein |
| Os.48036.1.A1_at | 2.19 | 0.0361 | LOC_Os09g15700 | Leucine Rich Repeat family protein |
| Os.4813.1.S1_at | 1.69 | 0.0096 | LOC_Os12g42570 | expressed protein |
| Os.48553.2.S1_x_at | 1.55 | 0.0472 | LOC_Os04g26260 | F-box domain containing protein |
| Os.48636.1.S1_at | 1.51 | 0.0177 | LOC_Os08g41400 | expressed protein |
| Os.48707.1.S1_at | 1.78 | 0.0026 | LOC_Os06g48650 | subtilase family protein |
| Os.48917.1.A1_at | 1.52 | 0.0483 | Os01g0973200 | Conserved hypothetical protein. |
| Os.48959.1.S1_at | 1.65 | 0.0153 | LOC_Os03g21510 | Protein kinase domain containing protein |
| Os.4898.1.S1_at | 1.94 | 0.0467 | LOC_Os04g52290 | pentatricopeptide |
| Os.49095.1.S1_at | 1.83 | 0.0108 | LOC_Os03g21140 | Zinc finger C-x8-C-x5-C-x3-H type family protein |
| Os.49163.1.S1_at | 2.08 | 0.0153 | LOC_Os03g47730 | Associated with HOX family protein |
| Os.49214.1.S1_at | 1.56 | 0.0116 | LOC_Os11g07050 | FHA domain containing protein |
| Os.49426.1.S1_at | 1.86 | 0.0068 | LOC_Os08g39160 | Phosphoribosylglycinamide formyltransferase, chloroplast precursor |
| Os.49455.1.S1_at | 1.81 | 0.0186 | Os04g0507000 | DNA mismatch repair protein. |
| Os.49557.1.S1_at | 1.64 | 0.0288 | LOC_Os07g12630 | transcription elongation factor S-II family protein |
| Os.49593.1.S1_x_at | 1.53 | 0.0048 | LOC_Os09g29060 | expressed protein |
| Os.49596.1.S1_at | 1.51 | 0.0133 | LOC_Os03g10460 | expressed protein |
| Os.49626.1.S1_at | 1.80 | 0.0077 | LOC_Os03g57260 | GRF zinc finger family protein |
| Os.49670.1.S1_at | 2.77 | 0.0112 | LOC_Os02g02500 | Remorin, C-terminal region family protein |
| Os.49732.1.S1_at | 1.52 | 0.0362 | LOC_Os06g23370 | expressed protein |
| Os.49738.1.S1_at | 1.56 | 0.0322 | LOC_Os06g01640 | Protein arginine N-methyltransferase |
| Os.50217.1.S1_at | 1.65 | 0.0428 | LOC_Os04g01480 | zinc finger family protein |
| Os.50286.1.S1_at | 1.60 | 0.0162 | LOC_Os02g14120 | BRASSINOSTEROID INSENSITIVE 1-associated receptor kinase 1 precursor |
| Os.50488.1.S1_at | 1.97 | 0.0013 | LOC_Os02g13950 | NHL repeat-containing protein |
| Os.50557.1.S1_at | 1.89 | 0.0297 | LOC_Os11g02270 | expressed protein |
| Os.5059.1.S1_at | 1.63 | 0.0457 | LOC_Os07g44400 | kinesin motor protein |
| Os.50597.1.S1_at | 1.54 | 0.0408 | LOC_Os11g04930 | SPRY domain containing protein |
| Os.50609.1.S1_at | 1.93 | 0.0019 | LOC_Os01g08540 | MutS domain III family protein |
| Os.50643.2.S1_x_at | 1.84 | 0.0413 | LOC_Os07g44690 | HSF-type DNA-binding domain containing protein |
| Os.50763.1.S1_at | 3.04 | 0.0379 | LOC_Os03g30260 | COBRA-like protein 2 precursor |
| Os.50790.1.S1_at | 1.54 | 0.0150 | LOC_Os03g52320 | SSXT protein containing protein |
| Os.50833.1.S2_at | 1.59 | 0.0014 | LOC_Os07g16370 | transposon protein , CACTA, En/Spm sub-class |
| Os.50904.1.S1_at | 1.53 | 0.0050 | LOC_Os04g46320 | Protein kinase domain containing protein |
| Os.50931.1.S1_at | 1.64 | 0.0082 | LOC_Os02g44860 | GDSL-like Lipase/Acylhydrolase family protein |
| Os.50938.1.S1_at | 2.06 | 0.0109 | LOC_Os12g04000 | Auxin efflux carrier component 3 |
| Os.50942.1.S1_at | 1.82 | 0.0231 | LOC_Os12g03360 | Nse1 non-SMC component of SMC5-6 complex family protein |
| Os.50945.1.S1_at | 1.85 | 0.0057 | LOC_Os06g39480 | transcription elongation factor |
| Os.51025.1.S1_at | 1.68 | 0.0009 | LOC_Os08g38310 | expressed protein |
| Os.51056.1.S1_at | 2.22 | 0.0136 | LOC_Os11g14390 | expressed protein |
| Os.51112.1.S1_at | 1.57 | 0.0368 | LOC_Os03g50860 | cytokinin receptor CRE1b |
| Os.51135.1.S1_at | 1.60 | 0.0255 | LOC_Os11g38930 | tRNA intron endonuclease, catalytic C-terminal domain containing protein |
| Os.51161.2.S1_at | 1.66 | 0.0262 | LOC_Os06g04190 | Rad1 |
| Os.51371.1.S1_at | 3.17 | 0.0146 | LOC_Os02g40410 | expressed protein |
| Os.51650.1.S1_at | 1.57 | 0.0280 | LOC_Os01g56020 | expressed protein |
| Os.51682.1.S1_at | 1.66 | 0.0085 | LOC_Os03g02290 | kinesin motor protein |
| Os.51732.1.S1_at | 3.06 | 0.0034 | LOC_Os09g31438 | unspliced-genomic OsSPL17 - SBP-box gene family member, expressed |
| Os.51821.1.S1_at | 1.65 | 0.0433 | LOC_Os04g40130 | Rf1 protein, mitochondrial precursor |
| Os.51837.1.S1_at | 1.57 | 0.0240 | LOC_Os06g36220 | F-box domain containing protein |
| Os.51844.1.S1_at | 1.64 | 0.0298 | LOC_Os05g39840 | DNA replication licensing factor MCM3 homolog |
| Os.51948.1.S1_at | 1.56 | 0.0332 | LOC_Os01g44180 | hypothetical protein |
| Os.51949.1.S1_at | 1.68 | 0.0335 | Os04g0465700 | RNA dependent RNA polymerase family protein. |
| Os.51953.1.S1_at | 1.59 | 0.0201 | LOC_Os12g39830 | Cyclin, N-terminal domain containing protein |
| Os.52203.1.S1_at | 1.53 | 0.0168 | LOC_Os12g36100 | kinesin motor protein |
| Os.52477.1.S1_at | 1.56 | 0.0338 | LOC_Os03g19190 | BRCA1 C Terminus domain containing protein |
| Os.52749.1.S1_at | 1.54 | 0.0431 | LOC_Os04g42110 | Cell division control protein 48 homolog B |
| Os.52754.1.S1_x_at | 1.65 | 0.0225 | LOC_Os03g50220 | Tat binding protein 1 containing protein |
| Os.52838.1.S1_at | 1.82 | 0.0027 | LOC_Os03g59650 | BRCA1 C Terminus domain containing protein |
| Os.52927.1.S1_at | 1.59 | 0.0004 | LOC_Os05g22990 | expressed protein |
| Os.53073.1.S1_at | 1.53 | 0.0053 | LOC_Os02g51880 | amine oxidase family protein |
| Os.53379.1.S1_at | 1.60 | 0.0165 | LOC_Os05g30240 | pentatricopeptide |
| Os.53380.1.S1_at | 1.76 | 0.0089 | LOC_Os05g38980 | respiratory burst oxidase protein F |
| Os.53453.1.S1_at | 3.64 | 0.0020 | LOC_Os12g43940 | TPR Domain containing protein |
| Os.53455.1.S1_at | 1.58 | 0.0051 | LOC_Os12g44130 | expressed protein |
| Os.53486.1.S1_at | 3.42 | 0.0458 | LOC_Os02g43560 | WRKY family transcription factor |
| Os.53527.1.S1_at | 1.87 | 0.0440 | LOC_Os09g33600 | ARF-GAP |
| Os.53537.1.S1_at | 1.58 | 0.0381 | LOC_Os03g17610 | DNA topoisomerase VIA |
| Os.53598.1.S1_at | 2.03 | 0.0333 | LOC_Os07g48370 | Glycosyl transferase family 8 protein |
| Os.53618.1.S1_at | 1.71 | 0.0013 | LOC_Os05g02040 | replication factor-a protein 1 containing protein |
| Os.53632.1.S1_at | 1.56 | 0.0249 | LOC_Os05g13370 | rhomboid family protein |
| Os.53781.1.S1_at | 1.71 | 0.0176 | Os07g0184900 | ATP-dependent DNA helicase, 70 kDa subunit family protein. |
| Os.54092.1.S1_at | 1.51 | 0.0121 | LOC_Os03g60180 | expressed protein |
| Os.54112.1.S1_at | 1.52 | 0.0064 | Os02g0247200 | T-complex protein 1, gamma subunit (TCP-1-gamma) |
| Os.54123.1.S1_at | 1.72 | 0.0102 | LOC_Os07g05650 | MAGE-8 antigen |
| Os.54130.1.S1_at | 1.63 | 0.0051 | LOC_Os04g40050 | expressed protein |
| Os.54185.1.S1_at | 1.67 | 0.0312 | Os01g0949200 | Protein of unknown function DUF731 family protein. |
| Os.54191.1.S1_at | 1.88 | 0.0036 | LOC_Os05g43610 | BRCA1 C Terminus domain containing protein |
| Os.54247.1.S1_at | 1.56 | 0.0035 | LOC_Os01g67550 | FAD binding domain containing protein |
| Os.54315.1.S1_at | 1.50 | 0.0065 | LOC_Os02g07180 | expressed protein |
| Os.54353.1.S1_at | 2.22 | 0.0066 | LOC_Os06g04370 | leucine-rich repeat transmembrane protein kinase |
| Os.54394.1.S1_at | 1.67 | 0.0347 | LOC_Os02g52460 | Zinc knuckle family protein |
| Os.54640.1.S1_at | 1.54 | 0.0054 | LOC_Os04g40290 | ATPase, AAA family protein |
| Os.54657.1.S1_at | 1.51 | 0.0390 | LOC_Os05g01190 | hAT family dimerisation domain containing protein |
| Os.54791.1.S1_at | 2.04 | 0.0425 | LOC_Os02g53000 | LysM domain GPI-anchored protein 1 precursor |
| Os.55062.1.S1_at | 1.59 | 0.0083 | LOC_Os12g07350 | tRNA-nucleotidyltransferase |
| Os.55135.1.S1_at | 1.59 | 0.0188 | LOC_Os06g43280 | elongator component |
| Os.55328.1.S1_at | 2.14 | 0.0050 | LOC_Os07g14890 | expressed protein |
| Os.554.1.S1_at | 1.52 | 0.0232 | LOC_Os01g08150 | expressed protein |
| Os.55604.1.S1_at | 3.39 | 0.0072 | LOC_Os03g24070 | hydrolase |
| Os.56996.1.S1_at | 1.96 | 0.0264 | LOC_Os03g49200 | Cell division cycle protein cdt2 |
| Os.57063.1.S1_at | 1.72 | 0.0100 | LOC_Os11g14240 | expressed protein |
| Os.57347.1.S1_at | 2.09 | 0.0175 | LOC_Os05g50310 | ZF-HD protein dimerisation region containing protein |
| Os.57558.1.S1_at | 3.86 | 0.0260 | LOC_Os04g51000 | Transcription factor FL |
| Os.5771.1.S1_a_at | 2.08 | 0.0070 | Os09g0441900 | Hypothetical protein. |
| Os.5799.1.S1_s_at | 1.52 | 0.0336 | LOC_Os03g58810 | ATP-dependent RNA helicase C1F7.02c |
| Os.6080.1.S1_at | 1.54 | 0.0128 | LOC_Os06g04380 | Glycine cleavage T-protein containing protein |
| Os.6180.1.S1_at | 1.67 | 0.0003 | LOC_Os07g14100 | pentatricopeptide |
| Os.6219.1.S1_at | 1.71 | 0.0061 | LOC_Os04g45330 | yabby14 protein |
| Os.6262.1.S1_at | 1.91 | 0.0341 | LOC_Os07g41180 | Nuclear transport factor 2 domain containing protein |
| Os.6636.1.S1_at | 3.15 | 0.0006 | LOC_Os08g39890 | SBP-domain protein 5 |
| Os.7177.2.S1_a_at | 2.13 | 0.0038 | LOC_Os04g57610 | Auxin response factor 8 |
| Os.7316.1.S1_at | 1.60 | 0.0007 | LOC_Os03g17164 | 125 kDa kinesin-related protein, putative, expressed |
| Os.7402.1.S1_at | 1.56 | 0.0054 | LOC_Os05g08010 | F-box domain containing protein |
| Os.7593.1.S1_at | 1.86 | 0.0033 | LOC_Os08g12410 | Pectinesterase family protein |
| Os.7613.1.S1_at | 2.14 | 0.0489 | LOC_Os06g47830 | OB-fold nucleic acid binding domain containing protein |
| Os.7742.1.S1_a_at | 1.72 | 0.0018 | LOC_Os03g49420 | HEAT repeat family protein |
| Os.7790.1.S1_at | 1.77 | 0.0238 | LOC_Os05g02490 | Adenosine 3'-phospho 5'-phosphosulfate transporter 1 |
| Os.7967.1.S1_at | 1.57 | 0.0056 | LOC_Os02g56540 | Kinesin motor domain containing protein |
| Os.8207.1.S1_at | 1.61 | 0.0093 | LOC_Os04g34670 | tRNA-splicing endonuclease positive effector |
| Os.8281.1.S1_at | 1.63 | 0.0069 | LOC_Os12g41620 | transducin family protein |
| Os.8649.1.S1_at | 2.12 | 0.0315 | LOC_Os11g43600 | Peptide chain release factor 1 |
| Os.8764.1.A1_a_at | 1.58 | 0.0108 | LOC_Os11g42420 | Nuclear pore protein 84/107 containing protein |
| Os.8816.1.S1_at | 1.70 | 0.0061 | Os02g0621300 | Sterol desaturase family protein. |
| Os.9033.1.S1_at | 1.64 | 0.0015 | LOC_Os03g44610 | IQ calmodulin-binding motif family protein |
| Os.9144.1.S1_a_at | 1.92 | 0.0020 | LOC_Os06g05820 | DNA repair protein RadA containing protein |
| Os.9351.1.S1_at | 2.06 | 0.0189 | LOC_Os06g39640 | PINHEAD protein |
| Os.9484.1.S2_at | 1.93 | 0.0019 | LOC_Os12g09580 | expressed protein |
| Os.9596.1.S1_at | 1.51 | 0.0051 | LOC_Os02g51180 | Cullin family protein |
| Os.9751.1.S1_at | 1.66 | 0.0009 | LOC_Os11g40090 | expressed protein |
| Os.9782.1.S1_at | 1.60 | 0.0267 | LOC_Os09g19930 | HOTHEAD protein precursor |
| Os.9892.1.S1_a_at | 1.62 | 0.0214 | LOC_Os01g65460 | Beta-galactosidase precursor |
| OsAffx.11589.1.S1_at | 1.78 | 0.0125 | Os01g0737300 | Diphosphonucleotide phosphatase 2. |
| OsAffx.11589.1.S1_x_at | 1.71 | 0.0166 | LOC_Os01g53560 | phosphoesterase |
| OsAffx.11640.1.S1_at | 1.62 | 0.0219 | LOC_Os01g57920 | F-box domain containing protein |
| OsAffx.11862.1.S1_at | 1.65 | 0.0244 | LOC_Os02g01940 | jmjC domain containing protein |
| OsAffx.12064.1.S1_at | 1.56 | 0.0127 | LOC_Os02g15980 | expressed protein |
| OsAffx.12351.1.S1_at | 1.57 | 0.0239 | LOC_Os02g34750 | Protein kinase domain containing protein |
| OsAffx.12465.1.S1_at | 1.68 | 0.0012 | LOC_Os02g42910 | expressed protein |
| OsAffx.13048.1.S1_at | 1.61 | 0.0161 | LOC_Os03g26220 | expressed protein |
| OsAffx.14221.1.S1_x_at | 1.58 | 0.0316 | LOC_Os04g40720 | expressed protein |
| OsAffx.14597.1.S1_at | 1.52 | 0.0134 | LOC_Os05g08790 | MAR binding filament-like protein 1-1 |
| OsAffx.14759.1.S1_s_at | 1.90 | 0.0151 | LOC_Os05g19270 | DNA mismatch repair protein MSH2 |
| OsAffx.16346.1.S1_at | 1.76 | 0.0076 | LOC_Os07g20420 | phosphoglycerate/bisphosphoglycerate mutase family protein |
| OsAffx.16533.1.S1_at | 1.53 | 0.0198 | LOC_Os07g32890 | 3'-5' exonuclease family protein |
| OsAffx.18040.1.S1_at | 1.79 | 0.0151 | LOC_Os09g37920 | DNA repair helicase family protein |
| OsAffx.18767.1.S1_at | 1.57 | 0.0103 | Os11g0202200 | Cyclin-like F-box domain containing protein. |
| OsAffx.18947.1.S1_x_at | 2.51 | 0.0001 | LOC_Os11g19730 | expressed protein |
| OsAffx.19468.2.S1_at | 1.68 | 0.0173 | LOC_Os11g03670 | Mpv17/PMP22 family protein |
| OsAffx.19795.1.S1_s_at | 2.00 | 0.0402 | LOC_Os12g24240 | expressed protein |
| OsAffx.19845.1.S1_at | 1.88 | 0.0306 | LOC_Os12g27060 | hypothetical protein |
| OsAffx.2017.1.S1_s_at | 1.51 | 0.0351 | LOC_Os01g16100 | Translin family protein |
| OsAffx.21707.2.S1_x_at | 1.51 | 0.0195 | LOC_Os01g64900 | expressed protein |
| OsAffx.24075.1.S1_at | 1.64 | 0.0012 | LOC_Os02g03000 | division protein |
| OsAffx.24672.1.S1_at | 2.36 | 0.0134 | LOC_Os02g41770 | hypothetical protein |
| OsAffx.24863.1.S1_x_at | 2.03 | 0.0449 | LOC_Os02g54170 | Leucine Rich Repeat family protein |
| OsAffx.24895.1.S1_at | 1.60 | 0.0003 | LOC_Os02g56560 | Casein kinase I |
| OsAffx.24929.1.S1_at | 1.53 | 0.0349 | LOC_Os03g01160 | U-box domain containing protein |
| OsAffx.25555.1.S1_s_at | 2.66 | 0.0049 | LOC_Os03g45270 | expressed protein |
| OsAffx.25768.1.S1_x_at | 1.52 | 0.0390 | LOC_Os03g59480 | expressed protein |
| OsAffx.26779.2.S1_at | 1.84 | 0.0339 | LOC_Os05g16640 | expressed protein |
| OsAffx.27244.1.S1_x_at | 1.96 | 0.0249 | LOC_Os05g39850 | DNA replication licensing factor MCM3 homolog |
| OsAffx.27316.1.S1_at | 1.59 | 0.0020 | LOC_Os05g45340 | DNA polymerase III, delta subunit family protein |
| OsAffx.27550.1.S1_at | 1.81 | 0.0000 | LOC_Os06g10410 | Mechanosensitive ion channel family protein |
| OsAffx.27916.1.S1_at | 1.63 | 0.0254 | LOC_Os06g33520 | DEAD box RNA helicase |
| OsAffx.28461.1.S1_at | 1.77 | 0.0158 | LOC_Os07g15500 | OB-fold nucleic acid binding domain containing protein |
| OsAffx.29501.1.S1_at | 2.03 | 0.0025 | LOC_Os08g34050 | phosphofructokinase family protein |
| OsAffx.29804.1.S1_at | 1.67 | 0.0080 | LOC_Os09g11440 | hypothetical protein |
| OsAffx.30394.1.S1_at | 2.05 | 0.0074 | LOC_Os10g11820 | expressed protein |
| OsAffx.3080.1.S1_at | 1.58 | 0.0129 | LOC_Os02g55570 | Shugoshin C terminus family protein |
| OsAffx.31174.3.S1_x_at | 2.99 | 0.0078 | LOC_Os04g51490 | hypothetical protein |
| OsAffx.3283.1.S1_at | 1.71 | 0.0053 | LOC_Os03g20580 | expressed protein |
| OsAffx.4181.1.S1_at | 2.25 | 0.0080 | LOC_Os04g55130 | expressed protein |
| OsAffx.5213.1.S1_x_at | 2.18 | 0.0389 | LOC_Os07g04980 | hypothetical protein |
| OsAffx.6884.1.S1_at | 1.72 | 0.0172 | LOC_Os10g36190 | pentatricopeptide |
| OsAffx.7241.1.S1_at | 1.52 | 0.0279 | LOC_Os11g28980 | Gpi16 subunit, GPI transamidase component family protein |
| OsAffx.7660.1.S1_at | 1.54 | 0.0388 | LOC_Os12g22620 | Endonuclease/Exonuclease/phosphatase family protein |
