# Supplementary material for: Identification of rhizome-specific genes by genome-wide differential expression Analysis in Oryza longistaminata
Source: BMC Plant Biol. 2011 Jan 24;11:18. doi: 10.1186/1471-2229-11-18 (PMC3036607; doi:10.1186/1471-2229-11-18)
Supplement: Additional file 5 — The list of 29 genes specifically enriched in the shoot internodes (SI) of O. longistaminata and their annotated functions detected by the Affymetrix GeneChip Rice Genome Array. Word file for the list of genes enriched in the shoot internode of Oryza longistaminata and their function annotation. [file 1471-2229-11-18-S5.DOC]

**Additional file 5**. The list of 29 genes specifically enriched in the shoot internodes (SI) of *O. longistaminata* and their annotated functions detected by the Affymetrix GeneChip Rice Genome Array

| **Name** | **Ratio_1/2** | ***p* value** | **OsGI** | **Function Annotation** |
| --- | --- | --- | --- | --- |
| Os.116.1.S1_at | 1.75 | 0.0158 | LOC_Os01g15610 | hypothetical protein |
| Os.1182.1.S1_x_at | 2.19 | 0.0422 | Os01g0121700 | ABC transporter related domain containing protein. |
| Os.12363.1.S1_at | 3.28 | 0.0233 | LOC_Os04g58200 | Protochlorophyllide reductase A, |
| Os.12420.1.S1_at | 1.84 | 0.0234 | LOC_Os08g27720 | Pirin, putative, expressed |
| Os.12703.1.S1_at | 1.90 | 0.0063 | LOC_Os03g10090 | mannitol transporter, putative, expressed |
| Os.12724.1.S1_a_at | 1.60 | 0.0152 | LOC_Os08g36320 | Glutamate decarboxylase, putative, expressed |
| Os.15545.1.S1_at | 1.90 | 0.0059 | LOC_Os05g43300 | H1flk, putative, expressed |
| Os.20681.1.S1_x_at | 1.95 | 0.0371 | LOC_Os01g61500 | BCL-2 binding anthanogene-1, putative, expressed |
| Os.22281.1.S1_at | 1.52 | 0.0106 | Os03g0186600 | Transcription factor, MADS-box domain containing protein. |
| Os.32192.1.S1_at | 1.77 | 0.0245 | LOC_Os01g67090 | IQ calmodulin-binding motif family protein |
| Os.45486.1.S1_x_at | 2.32 | 0.0212 | Os01g0121700 | ABC transporter related domain containing protein. |
| Os.46776.1.S1_s_at | 2.09 | 0.0006 | LOC_Os09g26960 | Cytochrome P450 family protein, expressed |
| Os.47852.1.S1_at | 2.16 | 0.0380 | LOC_Os05g40220 | retrotransposon protein, putative, unclassified, |
| Os.47971.1.A1_at | 2.71 | 0.0128 | LOC_Os05g49350 | expressed protein |
| Os.51846.1.S1_x_at | 1.52 | 0.0100 | Os06g0677400 | 3-hydroxyisobutyrate dehydrogenase |
| Os.52847.1.S1_at | 1.75 | 0.0308 | LOC_Os09g11170 | IBR domain containing protein, expressed |
| Os.53086.1.S1_at | 1.77 | 0.0385 | LOC_Os03g59570 | cytokinin synthase, putative, expressed |
| Os.53348.2.S1_at | 2.99 | 0.0054 | Os11g0270500 | Disease resistance protein family protein. |
| Os.5347.1.S1_x_at | 1.56 | 0.0405 | LOC_Os01g52010 | Allinase, C-terminal domain containing protein, |
| Os.54942.1.S1_at | 2.50 | 0.0418 | Os09g0482000 | UBX domain containing protein. |
| Os.54960.1.S1_at | 2.02 | 0.0423 | LOC_Os05g25540 | Protein kinase domain containing protein, |
| Os.56014.1.S1_at | 1.51 | 0.0438 | LOC_Os01g40190 | transposon protein, putative, CACTA, En/Spm sub- |
| Os.5660.1.S1_at | 1.59 | 0.0350 | LOC_Os05g04170 | AMP-binding enzyme family protein, expressed |
| Os.57130.1.S1_at | 2.20 | 0.0228 | Os06g0681300 | PAK-box/P21-Rho-binding domain containing protein. |
| Os.9563.1.S1_at | 2.67 | 0.0382 | LOC_Os08g32160 | oxidoreductase, 2OG-Fe oxygenase family protein, |
| OsAffx.12732.1.S1_s_at | 1.72 | 0.0419 | LOC_Os03g04660 | Cytochrome P450 family protein, expressed |
| OsAffx.12962.1.S1_at | 1.71 | 0.0144 | LOC_Os03g21000 | Thioredoxin-like 1, putative, expressed |
| OsAffx.16462.1.S1_at | 1.68 | 0.0031 | LOC_Os07g28890 | hypothetical protein |
| OsAffx.7922.1.S1_at | 1.52 | 0.0498 | LOC_Os10g40230 | hypothetical protein |

Ratio 1/2 indicates signal1(avg)/signal2(avg) from Wilcoxon Rank-Sum tests
