# Supplementary material for: Identification of rhizome-specific genes by genome-wide differential expression Analysis in Oryza longistaminata
Source: BMC Plant Biol. 2011 Jan 24;11:18. doi: 10.1186/1471-2229-11-18 (PMC3036607; doi:10.1186/1471-2229-11-18)
Supplement: Additional file 6 — The list of 1974 genes specifically enriched in the young leaves (YL) of O. longistaminata and their annotated functions detected by the Affymetrix GeneChip Rice Genome Array. Word file for the list of genes enriched in the young leaf of Oryza longistaminata and their function annotation. [file 1471-2229-11-18-S6.DOC]

**Additional file 6**. The list of 1974 genes specifically enriched in the young leaves (YL) of *O. longistaminata* and their annotated functions detected by the Affymetrix GeneChip Rice Genome Array

| **Name** | **Ratio_1/2** | ***p* value** | **OsGI** | **Function Annotation** |
| --- | --- | --- | --- | --- |
| Os.10001.1.S1_at | 2.35 | 0.0414 | Os02g0326000 | Conserved hypothetical protein. |
| Os.10003.1.S1_at | 4.27 | 0.0285 | LOC_Os01g13390 | expressed protein |
| Os.10011.1.S1_at | 4.40 | 0.0022 | LOC_Os06g49530 | F-box domain containing protein, expressed |
| Os.10031.1.S1_at | 10.39 | 0.0018 | LOC_Os05g35140 | MtN3/saliva family protein, expressed |
| Os.10068.1.S1_at | 3.10 | 0.0051 | Os11g0604600 | Conserved hypothetical protein. |
| Os.10076.1.S1_at | 2.01 | 0.0177 | LOC_Os08g33830 | Polypyrimidine tract-binding protein homolog 1, putative, |
| Os.10078.1.S1_at | 2.31 | 0.0434 | LOC_Os09g314 | 70 DNA binding protein, putative, expressed |
| Os.10080.1.S1_at | 3.07 | 0.0211 | LOC_Os02g03070 | shrunken seed protein, putative, expressed |
| Os.10084.1.A1_at | 2.16 | 0.0414 | LOC_Os11g16290 | RWP-RK domain containing protein, expressed |
| Os.10087.1.S1_at | 3.56 | 0.0086 | LOC_Os04g51290 | expressed protein |
| Os.10113.1.S1_at | 12.44 | 0.0047 | LOC_Os08g39300 | Serine-glyoxylate aminotransferase, putative, expressed |
| Os.10120.1.S2_a_at | 3.79 | 0.0312 | LOC_Os12g02340 | Nonspecific lipid-transfer protein 3 precursor |
| Os.10120.2.S1_at | 2.47 | 0.0075 | LOC_Os12g02350 | Zinc finger, C3HC4 type family protein, expressed |
| Os.10128.1.S1_at | 2.84 | 0.0056 | LOC_Os08g43190 | oxidoreductase, zinc-binding dehydrogenase family protein, |
| Os.10129.1.S1_at | 2.93 | 0.0291 | LOC_Os04g01540 | expressed protein |
| Os.10132.1.S1_at | 2.46 | 0.0021 | LOC_Os09g36300 | Lon protease homolog 1, mitochondrial precursor, putative, |
| Os.10136.1.S1_at | 2.42 | 0.0005 | LOC_Os05g48630 | Photosystem I reaction center subunit VI |
| Os.10141.1.S1_at | 3.92 | 0.0125 | LOC_Os03g53750 | Narf, putative, expressed |
| Os.10144.1.S1_at | 2.82 | 0.0002 | LOC_Os08g41990 | Glutamate-1-semialdehyde 2,1-aminomutase |
| Os.10146.1.S1_at | 21.88 | 0.0020 | LOC_Os03g59320 | expressed protein |
| Os.10150.1.S1_at | 43.73 | 0.0206 | LOC_Os03g12890 | Branched-chain-amino-acid aminotransferase 5 |
| Os.10154.1.S1_at | 1.82 | 0.0432 | LOC_Os10g32540 | SAP domain containing protein, expressed |
| Os.10156.1.S1_a_at | 1.67 | 0.0067 | LOC_Os03g61260 | 50S ribosomal protein L18, chloroplast precursor, putative |
| Os.10200.1.S1_at | 9.96 | 0.0012 | LOC_Os08g05620 | Cytochrome P450 89A2, putative, expressed |
| Os.10220.1.S1_at | 3.25 | 0.0087 | LOC_Os07g48820 | Transcription factor HBP-1b, putative, expressed |
| Os.10227.1.S1_at | 2.56 | 0.0385 | LOC_Os01g54880 | prkcsh-prov protein, putative, expressed |
| Os.10235.1.S1_at | 2.99 | 0.0496 | LOC_Os07g48570 | Dof domain, zinc finger family protein, expressed |
| Os.10271.2.S1_x_at | 2.52 | 0.0017 | LOC_Os01g15860 | PRP1 splicing factor, N-terminal family protein, expressed |
| Os.10274.1.S1_a_at | 1.53 | 0.0014 | LOC_Os11g03970 | CIPK-like protein 1, putative, expressed |
| Os.10274.3.S1_at | 1.53 | 0.0458 | LOC_Os11g03980 | Calmodulin-6, putative |
| Os.10275.1.S1_at | 2.27 | 0.0020 | LOC_Os02g51080 | Geranylgeranyl hydrogenase, putative, expressed |
| Os.10279.1.S1_at | 1.83 | 0.0213 | LOC_Os01g64520 | Uricase, putative, expressed |
| Os.10292.1.S1_at | 7.59 | 0.0107 | LOC_Os01g16260 | transporter, putative, expressed |
| Os.10311.1.S1_a_at | 45.17 | 0.0015 | Os06g0254300 | Caleosin related family protein. |
| Os.10311.2.A1_x_at | 48.51 | 0.0033 | LOC_Os06g14350 | Calcium binding EF-hand protein, putative, expressed |
| Os.10314.1.S1_a_at | 2.25 | 0.0267 | LOC_Os02g55200 | expressed protein |
| Os.10320.1.S1_x_at | 3.33 | 0.0186 | LOC_Os01g15490 | phosphoadenosine phosphosulfate, putative, expressed |
| Os.10352.1.S1_at | 2.78 | 0.0286 | LOC_Os05g01470 | Methionine S-methyltransferase, putative, expressed |
| Os.10360.1.S1_at | 7.92 | 0.0031 | LOC_Os10g38110 | Cytochrome P450 family protein, expressed |
| Os.10370.2.S1_at | 1.87 | 0.0076 | LOC_Os07g47640 | Photosystem II reaction centre X protein |
| Os.10370.2.S1_x_at | 2.84 | 0.0039 | LOC_Os07g47640 | Photosystem II reaction centre X protein |
| Os.10378.2.A1_s_at | 2.00 | 0.0207 | LOC_Os01g72570 | expressed protein |
| Os.10410.1.S1_at | 3.39 | 0.0003 | LOC_Os08g02210 | expressed protein |
| Os.10427.1.S1_a_at | 4.47 | 0.0023 | LOC_Os12g04260 | saccharopine dehydrogenase family protein, expressed |
| Os.10447.1.S1_at | 2.40 | 0.0390 | LOC_Os05g03550 | Myb-like DNA-binding domain containing protein, expressed |
| Os.10454.1.S1_a_at | 7.53 | 0.0000 | LOC_Os08g27840 | Phosphoenolpyruvate carboxylase 2, putative, expressed |
| Os.10501.1.S1_at | 3.50 | 0.0050 | LOC_Os07g39430 | mTERF family protein, expressed |
| Os.10504.1.S1_at | 1.54 | 0.0469 | LOC_Os05g43540 | expressed protein |
| Os.10597.1.S1_at | 25.75 | 0.0467 | LOC_Os01g53350 | UDP-glucoronosyl and UDP-glucosyl transferase family protein |
| Os.10598.1.S1_at | 5.56 | 0.0080 | LOC_Os12g44310 | 9,10-9',10' carotenoid cleavage dioxygenase 1 |
| Os.10609.1.S1_at | 2.35 | 0.0025 | LOC_Os08g43560 | L-ascorbate peroxidase 4, putative, expressed |
| Os.10613.1.S1_at | 3.51 | 0.0035 | LOC_Os04g25400 | Coenzyme F420 hydrogenase/dehydrogenase |
| Os.10686.1.S1_at | 1.56 | 0.0108 | LOC_Os06g41770 | bZIP transcription factor family protein, expressed |
| Os.10689.1.S1_at | 7.34 | 0.0109 | Os06g0110000 | Ent-kaurenoic acid oxidase 2 |
| Os.10696.1.S1_at | 3.20 | 0.0142 | LOC_Os06g01850 | Ferredoxin-NADP reductase, leaf isozyme |
| Os.1070.1.S1_at | 2.00 | 0.0475 | LOC_Os01g03020 | Leucyl-tRNA synthetase, putative, expressed |
| Os.10700.1.S1_at | 3.55 | 0.0137 | LOC_Os05g05480 | DegP2 protease, putative, expressed |
| Os.1071.1.S1_at | 7.12 | 0.0152 | LOC_Os01g03040 | expressed protein |
| Os.10727.1.S1_at | 3.84 | 0.0034 | LOC_Os04g52100 | Methionine aminopeptidase, putative, expressed |
| Os.10743.1.S1_at | 17.64 | 0.0378 | LOC_Os06g33200 | expressed protein |
| Os.10776.1.S1_at | 2.37 | 0.0084 | LOC_Os02g40030 | UDP-sugar transporter sqv-7, putative, expressed |
| Os.10779.1.S1_at | 3.14 | 0.0010 | LOC_Os03g14690 | V-type ATPase 116kDa subunit family protein, expressed |
| Os.10786.1.S1_at | 1.88 | 0.0292 | LOC_Os03g31550 | Aldehyde oxidase and xanthine dehydrogenase |
| Os.10800.1.S1_at | 1.99 | 0.0243 | LOC_Os03g22060 | Uroporphyrinogen decarboxylase, chloroplast precursor |
| Os.10810.1.S1_at | 1.57 | 0.0199 | LOC_Os06g40630 | Protein SFT2, putative, expressed |
| Os.10822.1.S1_at | 2.22 | 0.0469 | LOC_Os11g40070 | expressed protein |
| Os.10823.2.S1_at | 2.18 | 0.0473 | LOC_Os05g40890 | expressed protein |
| Os.10849.1.S1_at | 4.03 | 0.0022 | LOC_Os03g62490 | Mitochondrial prohibitin complex protein 2, |
| Os.10853.1.S1_at | 8.74 | 0.0001 | LOC_Os02g39930 | Outer membrane lipoprotein blc precursor |
| Os.10862.1.S1_at | 3.40 | 0.0184 | LOC_Os06g05410 | expressed protein |
| Os.10872.1.S1_at | 2.83 | 0.0114 | LOC_Os07g09420 | ATPase, AAA family protein, expressed |
| Os.10892.1.S1_at | 4.95 | 0.0036 | Os05g0146100 | Peptidase, trypsin-like serine and cysteine proteases domain containing protein. |
| Os.10901.1.S1_a_at | 2.09 | 0.0174 | LOC_Os08g06110 | myb-like DNA-binding domain, SHAQKYF class family protein, |
| Os.10975.1.S1_at | 10.44 | 0.0003 | LOC_Os11g39020 | ABC transporter family protein, expressed |
| Os.11007.1.S1_at | 1.96 | 0.0404 | Os12g0538500 | PGPD14 protein. |
| Os.11010.1.S1_at | 1.59 | 0.0027 | LOC_Os05g22970 | Lactoylglutathione lyase, putative, expressed |
| Os.11014.1.S1_at | 2.33 | 0.0152 | LOC_Os05g08730 | expressed protein |
| Os.11041.1.S1_s_at | 3.37 | 0.0091 | LOC_Os11g26890 | expressed protein |
| Os.11078.1.S1_a_at | 1.74 | 0.0080 | LOC_Os07g36080 | Oxygen-evolving enhancer protein 3-1 |
| Os.11111.1.S2_at | 2.07 | 0.0036 | LOC_Os05g08810 | Phosphatidylinositol 3-kinase, root isoform |
| Os.11117.1.S2_at | 2.56 | 0.0103 | LOC_Os07g43050 | RNA recognition motif family protein, expressed |
| Os.11134.1.S1_at | 2.23 | 0.0013 | LOC_Os06g44150 | magnesium transporter CorA-like family protein |
| Os.11136.1.S1_at | 3.59 | 0.0002 | LOC_Os01g44210 | ribosomal protein L31 containing protein, expressed |
| Os.11138.2.S1_x_at | 1.88 | 0.0268 | LOC_Os07g46590 | Chromodomain-helicase-DNA-binding protein |
| Os.11150.1.S1_at | 19.46 | 0.0006 | LOC_Os03g32490 | expressed protein |
| Os.11152.1.S1_at | 1.80 | 0.0202 | LOC_Os01g54540 | 50S ribosomal protein L13, chloroplast precursor |
| Os.11184.1.S1_at | 2.82 | 0.0239 | LOC_Os04g31690 | expressed protein |
| Os.11185.1.S1_a_at | 1.85 | 0.0110 | LOC_Os02g17500 | Sugar transporter family protein, expressed |
| Os.11193.1.S1_at | 2.13 | 0.0367 | LOC_Os03g55240 | Cytochrome P450 family protein, expressed |
| Os.11222.1.S1_at | 3.40 | 0.0063 | LOC_Os01g40630 | Lysine Decarboxylase, putative, expressed |
| Os.11230.1.S1_s_at | 2.37 | 0.0159 | LOC_Os05g03040 | floral homeotic protein, putative, expressed |
| Os.11253.1.S1_at | 3.06 | 0.0140 | LOC_Os01g63220 | pfkB-type carbohydrate kinase family protein |
| Os.11281.1.S2_at | 3.88 | 0.0365 | LOC_Os06g48770 | beta-lactamase family protein, expressed |
| Os.11300.2.S1_at | 5.56 | 0.0042 | LOC_Os10g38910 | expressed protein |
| Os.11303.1.S1_at | 6.62 | 0.0498 | Os01g0950300 | Glutathione S-transferase GST 29 |
| Os.11306.1.S1_at | 1.64 | 0.0438 | LOC_Os08g37350 | Ubiquitin carboxyl-terminal hydrolase family protein |
| Os.11316.1.S1_at | 1.61 | 0.0337 | LOC_Os01g68020 | BTB/POZ domain containing protein, expressed |
| Os.11364.1.S1_at | 3.43 | 0.0082 | LOC_Os12g33080 | 2-oxoglutarate/malate translocator, chloroplast precursor, |
| Os.11387.1.S1_a_at | 7.40 | 0.0109 | LOC_Os01g73580 | Beta-fructofuranosidase, insoluble isoenzyme 4 precursor, |
| Os.11397.1.S1_at | 8.15 | 0.0010 | LOC_Os02g51570 | peptidyl-prolyl cis-trans isomerase, FKBP-type family |
| Os.11399.1.S1_at | 2.60 | 0.0041 | LOC_Os05g28200 | chlorophyll synthase, ChlG family protein, expressed |
| Os.11407.1.S1_at | 1.99 | 0.0438 | LOC_Os04g57550 | amine oxidase, flavin-containing family protein, expressed |
| Os.11429.1.S1_at | 3.86 | 0.0021 | LOC_Os01g05060 | Mitochondrial glycoprotein, expressed |
| Os.11431.1.S1_at | 1.73 | 0.0482 | LOC_Os04g49990 | AT hook motif family protein, expressed |
| Os.11458.1.S1_at | 3.44 | 0.0280 | LOC_Os08g04840 | myb-like DNA-binding domain, SHAQKYF class family protein, |
| Os.1148.1.S1_at | 3.17 | 0.0196 | LOC_Os03g03020 | 50S ribosomal protein L11, chloroplast precursor |
| Os.1149.1.S1_at | 5.27 | 0.0016 | LOC_Os03g22160 | RelA/SpoT protein, putative, expressed |
| Os.1150.1.S1_at | 3.72 | 0.0308 | LOC_Os05g06890 | RelA-SpoT like protein RSH4, putative, expressed |
| Os.11566.1.S1_at | 11.70 | 0.0001 | LOC_Os02g47620 | hydrolase, alpha/beta fold family protein, expressed |
| Os.11567.1.S2_a_at | 1.65 | 0.0029 | LOC_Os02g34590 | transposon protein, putative, unclassified, expressed |
| Os.11573.2.A1_a_at | 4.18 | 0.0039 | LOC_Os11g47970 | Ribulose bisphosphate carboxylase/oxygenase activase |
| Os.11573.2.A2_a_at | 5.08 | 0.0068 | LOC_Os11g47970 | Ribulose bisphosphate carboxylase/oxygenase activase |
| Os.11591.1.S1_x_at | 1.68 | 0.0420 | LOC_Os01g56580 | protein kinase family protein, putative, expressed |
| Os.11602.1.S1_at | 14.41 | 0.0022 | LOC_Os09g38090 | expressed protein |
| Os.11619.1.A1_at | 2.31 | 0.0304 | LOC_Os02g57160 | ABC1 family protein, expressed |
| Os.11622.1.S1_at | 3.29 | 0.0323 | LOC_Os06g01360 | Homogentisate 1,2-dioxygenase, putative, expressed |
| Os.11629.1.S1_at | 2.37 | 0.0427 | LOC_Os09g02710 | CBS domain containing protein, expressed |
| Os.11630.1.S1_at | 3.51 | 0.0036 | LOC_Os01g73450 | Uridylate kinase, putative, expressed |
| Os.11632.1.S1_at | 5.84 | 0.0145 | LOC_Os08g42590 | MtN19, putative, expressed |
| Os.11634.1.S1_at | 1.93 | 0.0030 | LOC_Os02g05630 | Protein phosphatase 2C containing protein, expressed |
| Os.11637.1.S1_at | 2.02 | 0.0365 | LOC_Os04g39270 | Indole-3-glycerol phosphate synthase, chloroplast precursor |
| Os.11652.1.S1_at | 3.17 | 0.0167 | LOC_Os08g15230 | heat shock protein, putative, expressed |
| Os.11654.1.S1_at | 12.18 | 0.0003 | LOC_Os06g39690 | SOUL heme-binding protein, expressed |
| Os.11664.1.S1_at | 2.77 | 0.0089 | LOC_Os08g01770 | Adenylate kinase, chloroplast, putative, expressed |
| Os.11673.2.S1_x_at | 2.74 | 0.0022 | LOC_Os01g56780 | Plus-3 domain containing protein, expressed |
| Os.11674.1.S1_at | 19.60 | 0.0003 | LOC_Os07g05000 | oxidoreductase, aldo/keto reductase family protein, expressed |
| Os.11687.1.S1_a_at | 4.66 | 0.0150 | LOC_Os03g61220 | DEAD/DEAH box helicase family protein, expressed |
| Os.11702.1.S1_at | 1.76 | 0.0266 | LOC_Os04g31900 | CG-1 domain containing protein, expressed |
| Os.11704.1.S1_at | 1.92 | 0.0199 | LOC_Os03g58090 | expressed protein |
| Os.11724.2.S2_s_at | 2.50 | 0.0131 | LOC_Os06g24730 | hydrolase, alpha/beta fold family protein, expressed |
| Os.11725.1.S1_at | 5.73 | 0.0073 | LOC_Os09g13940 | AP2 domain containing protein, expressed |
| Os.11736.1.S1_at | 4.80 | 0.0261 | LOC_Os02g22100 | Rhomboid family protein, expressed |
| Os.11753.1.S1_at | 12.73 | 0.0021 | LOC_Os07g06660 | lactoylglutathione lyase family protein, putative, expressed |
| Os.11756.1.S1_at | 5.77 | 0.0234 | LOC_Os03g16050 | Fructose-1,6-bisphosphatase, chloroplast precursor, putatitive |
| Os.11787.1.S1_at | 2.47 | 0.0153 | LOC_Os07g01020 | pyridoxin biosynthesis protein ER1, putative, expressed |
| Os.11800.1.S1_at | 56.00 | 0.0459 | LOC_Os01g50100 | multidrug resistance protein 1 homolog, putative, expressed |
| Os.11802.1.S1_at | 1.71 | 0.0371 | LOC_Os03g51650 | expressed protein |
| Os.11805.1.A1_s_at | 3.69 | 0.0239 | LOC_Os11g06020 | Associated with HOX family protein, expressed |
| Os.11807.1.S1_at | 14.23 | 0.0004 | Os06g0581000 | TGF-beta receptor, type I/II extracellular region family protein. |
| Os.11808.1.S1_at | 1.80 | 0.0167 | Os03g0161500 | Conserved hypothetical protein. |
| Os.11810.2.S1_a_at | 3.32 | 0.0140 | LOC_Os09g04790 | Light-induced protein, chloroplast precursor, putative |
| Os.11824.1.S1_x_at | 4.46 | 0.0020 | LOC_Os07g01760 | Alanine aminotransferase 2, putative, expressed |
| Os.11830.1.S1_at | 47.34 | 0.0250 | LOC_Os05g30350 | Glycosyl hydrolase family 1 protein, expressed |
| Os.11841.1.S1_at | 4.84 | 0.0044 | LOC_Os08g45190 | PGR5, putative, expressed |
| Os.11844.1.S1_at | 4.77 | 0.0134 | LOC_Os01g37040 | expressed protein |
| Os.11845.1.S1_a_at | 2.97 | 0.0004 | LOC_Os12g42550 | Methyl-CpG binding domain containing protein, expressed |
| Os.11854.1.S1_at | 7.82 | 0.0066 | LOC_Os03g49720 | plastid-lipid associated protein PAP, putative, expressed |
| Os.11858.1.S1_at | 7.93 | 0.0042 | LOC_Os03g58080 | folate/biopterin transporter family protein, expressed |
| Os.11860.1.S1_at | 3.61 | 0.0301 | LOC_Os05g05600 | ATA15 protein, putative, expressed |
| Os.11866.1.S1_at | 3.29 | 0.0406 | LOC_Os01g62460 | SCARECROW, putative, expressed |
| Os.11874.1.S1_s_at | 2.56 | 0.0088 | LOC_Os07g18720 | expressed protein |
| Os.11875.1.S1_a_at | 1.94 | 0.0342 | LOC_Os12g18650 | regulator of chromosome condensation, putative, expressed |
| Os.11888.3.S1_x_at | 1.89 | 0.0104 | LOC_Os03g08270 | Ataxin-2 C-terminal region family protein, expressed |
| Os.11889.1.S2_at | 2.16 | 0.0266 | LOC_Os02g39010 | PITSLRE serine/threonine-protein kinase CDC2L2, putative, |
| Os.1191.1.S1_at | 2.68 | 0.0257 | LOC_Os03g04060 | Basic endochitinase precursor, putative, expressed |
| Os.11917.1.S1_at | 6.21 | 0.0039 | LOC_Os06g47970 | expressed protein |
| Os.11918.1.S1_at | 6.41 | 0.0152 | LOC_Os10g34400 | expressed protein |
| Os.11927.1.S1_x_at | 3.81 | 0.0065 | LOC_Os03g18200 | DnaJ domain containing protein, expressed |
| Os.11929.1.S1_at | 3.28 | 0.0406 | LOC_Os03g38730 | peroxisomal membrane protein, putative, expressed |
| Os.1193.1.S1_at | 16.76 | 0.0124 | LOC_Os04g43070 | Ammonium transporter 1, member 2, putative, expressed |
| Os.11935.1.S1_at | 3.44 | 0.0163 | LOC_Os02g32520 | ERD1 protein, chloroplast precursor, putative, expressed |
| Os.11943.1.S1_s_at | 1.75 | 0.0126 | LOC_Os08g45140 | Glutaredoxin, putative, expressed |
| Os.11947.1.S1_a_at | 6.81 | 0.0001 | LOC_Os11g11000 | ABC1 family protein, expressed |
| Os.11949.1.S1_at | 1.68 | 0.0140 | LOC_Os03g12350 | myb-like DNA-binding domain, SHAQKYF class family protein, |
| Os.11962.1.S1_at | 14.73 | 0.0021 | LOC_Os09g29200 | Glutathione S-transferase, N-terminal domain containing protein |
| Os.11970.1.S1_at | 2.92 | 0.0067 | LOC_Os09g20240 | expressed protein |
| Os.11970.2.S1_x_at | 3.54 | 0.0271 | LOC_Os09g20240 | expressed protein |
| Os.11972.1.S1_s_at | 2.56 | 0.0179 | LOC_Os05g33010 | DnaJ domain containing protein, expressed |
| Os.11976.1.S1_a_at | 3.16 | 0.0192 | LOC_Os01g67120 | Rhodanese-like domain containing protein, expressed |
| Os.11981.1.S1_x_at | 8.72 | 0.0388 | LOC_Os02g39730 | expressed protein |
| Os.11985.2.A1_a_at | 1.64 | 0.0322 | LOC_Os10g42710 | Poly polymerase catalytic domain containing protein |
| Os.11987.1.S1_at | 4.33 | 0.0358 | LOC_Os03g07360 | Dof domain, zinc finger family protein, expressed |
| Os.11988.1.S1_at | 2.00 | 0.0273 | LOC_Os08g08820 | START domain containing protein, expressed |
| Os.11990.1.S1_a_at | 3.68 | 0.0317 | Os01g0763700 | Exo70 exocyst complex subunit family protein. |
| Os.11990.1.S2_at | 4.24 | 0.0316 | Os01g0763700 | Exo70 exocyst complex subunit family protein. |
| Os.11994.1.S1_at | 2.71 | 0.0305 | Os07g0604500 | Mitochondrial inner membrane translocase TM17-2 (Fragment). |
| Os.11995.1.S1_at | 3.22 | 0.0001 | LOC_Os02g07160 | 4-hydroxyphenylpyruvate dioxygenase, putative, expressed |
| Os.11998.1.S1_at | 4.06 | 0.0037 | LOC_Os02g19150 | ATP-dependent Clp protease ATP-binding subunit clpX |
| Os.11999.1.S1_at | 2.28 | 0.0206 | LOC_Os05g02010 | expressed protein |
| Os.12008.1.S1_at | 3.99 | 0.0031 | Os06g0360300 | Conserved hypothetical protein. |
| Os.12020.1.S1_at | 1.76 | 0.0108 | LOC_Os05g35170 | No apical meristem protein, expressed |
| Os.12024.1.S1_at | 1.94 | 0.0317 | LOC_Os06g43900 | Plastid ribosomal protein L35, putative, expressed |
| Os.12025.1.S1_a_at | 8.82 | 0.0361 | LOC_Os04g55180 | alpha/beta hydrolase fold PF|00561 containing protein |
| Os.12037.1.S1_at | 21.29 | 0.0287 | LOC_Os03g146 | 69 nuclear transcription factor Y subunit C-1, putative, expressed |
| Os.12049.1.S1_at | 1.51 | 0.0298 | Os04g0451900 | Protein phosphatase 2C-like domain containing protein. |
| Os.12052.1.S1_at | 2.01 | 0.0125 | Os10g0430700 | Conserved hypothetical protein. |
| Os.12053.1.S1_at | 3.12 | 0.0059 | LOC_Os05g32220 | 50S ribosomal protein L1, putative, expressed |
| Os.12056.1.S1_at | 2.50 | 0.0091 | LOC_Os07g05480 | Photosystem I reaction center subunit psaK |
| Os.12065.1.S1_at | 3.64 | 0.0067 | LOC_Os05g51450 | ATP-dependent Clp protease proteolytic subunit, putative, |
| Os.12078.1.S1_at | 1.57 | 0.0483 | LOC_Os02g42730 | expressed protein |
| Os.12089.1.S1_at | 24.83 | 0.0244 | Os07g0601000 | NADPH HC toxin reductase |
| Os.12100.1.S1_at | 8.20 | 0.0035 | LOC_Os07g29410 | thioredoxin family protein, putative, expressed |
| Os.12101.2.A1_a_at | 2.19 | 0.0131 | LOC_Os03g31839 | unspliced-genomic transposon protein, putative,unclassified |
| Os.12102.1.S1_at | 8.07 | 0.0005 | Os05g0168300 | Haloacid dehalogenase-like hydrolase domain containing protein. |
| Os.12110.1.S1_at | 7.09 | 0.0244 | LOC_Os12g02530 | RPT2, putative, expressed |
| Os.12112.1.S1_at | 20.14 | 0.0106 | LOC_Os01g60830 | expressed protein |
| Os.12115.1.S1_at | 2.17 | 0.0030 | LOC_Os06g09900 | expressed protein |
| Os.12125.1.S1_at | 2.12 | 0.0209 | LOC_Os04g54430 | expressed protein |
| Os.12126.2.S1_at | 8.43 | 0.0012 | LOC_Os04g43420 | peptidoglycan-binding domain-containing protein, putative, |
| Os.12127.2.S1_x_at | 4.69 | 0.0004 | LOC_Os09g30340 | Photosystem I reaction center subunit V |
| Os.12134.1.S1_at | 3.92 | 0.0001 | LOC_Os04g45810 | Homeobox domain containing protein, expressed |
| Os.12141.1.S1_at | 2.71 | 0.0067 | LOC_Os03g32270 | hydrolase, alpha/beta fold family protein, expressed |
| Os.12148.1.S1_at | 1.91 | 0.0067 | LOC_Os03g52840 | Serine hydroxymethyltransferase, mitochondrial precursor, |
| Os.12165.1.S1_at | 1.85 | 0.0471 | LOC_Os07g32570 | 5'-adenylylsulfate reductase 2, chloroplast precursor |
| Os.12196.1.S1_at | 2.05 | 0.0167 | LOC_Os02g33450 | 2-cys peroxiredoxin BAS1, chloroplast precursor, putative, |
| Os.12216.1.S1_at | 1.81 | 0.0382 | LOC_Os04g57600 | Zinc finger C-x8-C-x5-C-x3-H type family protein, expressed |
| Os.12218.1.S1_at | 2.03 | 0.0225 | LOC_Os03g59050 | ATP-dependent RNA helicase An3, putative, expressed |
| Os.12261.1.S1_a_at | 2.74 | 0.0025 | LOC_Os03g20380 | CIPK-like protein 1, putative, expressed |
| Os.12277.1.S1_at | 2.36 | 0.0095 | LOC_Os06g04070 | Arginine decarboxylase, putative, expressed |
| Os.12283.1.S1_at | 1.86 | 0.0020 | LOC_Os03g56670 | Photosystem I reaction center subunit III, |
| Os.1229.2.S1_at | 8.52 | 0.0263 | LOC_Os01g13740 | unspliced-genomic myb-like DNA-binding domain containing protein, expressed |
| Os.1229.2.S1_x_at | 6.43 | 0.0118 | LOC_Os01g13740 | myb-like DNA-binding domain, SHAQKYF class family protein, |
| Os.12295.1.S1_at | 1.66 | 0.0272 | LOC_Os04g33830 | 16kDa membrane protein, putative, expressed |
| Os.12312.1.S1_at | 2.72 | 0.0174 | LOC_Os01g69920 | histidine kinase 2, putative, expressed |
| Os.12313.1.S1_at | 6.33 | 0.0460 | LOC_Os05g15260 | Photosystem I |
| Os.12333.1.S1_at | 1.71 | 0.0391 | LOC_Os08g13070 | BTB/POZ domain containing protein, expressed |
| Os.12347.1.S1_x_at | 5.14 | 0.0061 | LOC_Os11g04390 | RNA recognition motif family protein, expressed |
| Os.12371.1.S1_at | 14.89 | 0.0076 | LOC_Os04g41340 | phosphoglycolate/pyridoxal phosphate phosphatase family |
| Os.12371.1.S2_at | 4.42 | 0.0041 | LOC_Os07g32880 | ATP synthase gamma chain, chloroplast precursor |
| Os.12376.1.S1_at | 2.29 | 0.0342 | LOC_Os06g19990 | expressed protein |
| Os.12394.1.S1_at | 12.02 | 0.0059 | LOC_Os03g57220 | expressed protein |
| Os.12449.1.A1_at | 2.12 | 0.0259 | LOC_Os08g37950 | Copper-transporting ATPase PAA1, putative, expressed |
| Os.12484.1.S1_at | 3.72 | 0.0400 | LOC_Os02g45670 | myb family transcription factor, putative, expressed |
| Os.12592.1.S1_at | 1.55 | 0.0343 | LOC_Os07g36140 | Histone H2A, putative, expressed |
| Os.12594.1.S1_x_at | 10.09 | 0.0074 | LOC_Os12g17600 | Ribulose bisphosphate carboxylase small chain C |
| Os.12607.1.S1_s_at | 2.44 | 0.0082 | LOC_Os08g44860 | Peptidase family M1 containing protein, expressed |
| Os.12608.1.S1_at | 7.41 | 0.0003 | LOC_Os07g06450 | RNA recognition motif family protein, expressed |
| Os.12611.1.S1_at | 2.57 | 0.0017 | LOC_Os04g36760 | enzyme of the cupin superfamily, putative, expressed |
| Os.12615.1.A1_at | 5.03 | 0.0404 | LOC_Os03g23980 | NAD dependent epimerase/dehydratase family protein |
| Os.12622.1.S1_at | 2.67 | 0.0081 | LOC_Os03g07300 | Ribulose-phosphate 3-epimerase, chloroplast precursor |
| Os.12623.1.S1_at | 5.03 | 0.0063 | LOC_Os01g66850 | Pectinacetylesterase, putative, expressed |
| Os.12627.1.S1_at | 1.95 | 0.0075 | LOC_Os07g04840 | Oxygen-evolving enhancer protein 2, chloroplast precursor, |
| Os.12628.1.S1_at | 1.62 | 0.0333 | LOC_Os06g02380 | RuBisCO subunit binding-protein beta subunit |
| Os.12645.1.S1_at | 1.96 | 0.0410 | LOC_Os03g27280 | Abscisic acid-inducible protein kinase, putative, expressed |
| Os.12653.1.S1_at | 1.60 | 0.0011 | LOC_Os04g51370 | Protein kinase domain containing protein, expressed |
| Os.12669.1.S1_at | 1.73 | 0.0406 | LOC_Os09g03620 | Protein kinase domain containing protein, expressed |
| Os.12691.1.A1_x_at | 1.56 | 0.0295 | LOC_Os03g20780 | ETHYLENE-INSENSITIVE3-like 1 protein, putative, expressed |
| Os.12696.1.S1_at | 4.22 | 0.0240 | LOC_Os04g16680 | Sedoheptulose-1,7-bisphosphatase, chloroplast precursor |
| Os.12699.1.S1_at | 7.92 | 0.0003 | LOC_Os03g15050 | Phosphoenolpyruvate carboxykinase, putative, expressed |
| Os.12701.1.S2_at | 1.52 | 0.0214 | LOC_Os04g55650 | Low-temperature-induced cysteine proteinase precursor |
| Os.12713.1.S1_at | 1.86 | 0.0351 | LOC_Os06g21590 | Chlorophyll a-b binding protein 6A, chloroplast precursor, |
| Os.12728.1.S1_at | 2.06 | 0.0399 | LOC_Os03g12290 | Glutamine synthetase root isozyme 5, putative, expressed |
| Os.12738.1.S2_a_at | 3.78 | 0.0129 | LOC_Os01g48960 | Glutamate synthase, chloroplast precursor, putative |
| Os.12771.1.S1_at | 9.76 | 0.0205 | LOC_Os01g73910 | expressed protein |
| Os.12788.1.S1_at | 1.72 | 0.0345 | LOC_Os01g11240 | expressed protein |
| Os.12795.1.S1_at | 2.07 | 0.0090 | LOC_Os11g05930 | CCT motif family protein, expressed |
| Os.12805.1.S1_at | 5.21 | 0.0008 | LOC_Os04g41560 | B-box zinc finger family protein, expressed |
| Os.12837.1.S1_at | 3.19 | 0.0444 | LOC_Os03g55720 | cytochrome b6f complex subunit, putative, expressed |
| Os.12844.1.A1_a_at | 4.37 | 0.0047 | Os07g0595700 | Conserved hypothetical protein. |
| Os.12865.1.S1_at | 3.77 | 0.0480 | Os12g0550600 | Conserved hypothetical protein. |
| Os.12931.1.S1_at | 1.83 | 0.0244 | LOC_Os05g23940 | Transcription factor TFIIB repeat family protein, expressed |
| Os.12932.1.S1_at | 4.55 | 0.0268 | LOC_Os11g43600 | Peptide chain release factor 1, putative, expressed |
| Os.12967.1.A1_a_at | 1.55 | 0.0409 | LOC_Os01g68320 | ATP-dependent RNA helicase DBP2, putative, expressed |
| Os.12967.2.A1_at | 7.73 | 0.0311 | LOC_Os09g28740 | 2-Hydroxyisoflavanone dehydratase, putative, expressed |
| Os.12977.1.S1_at | 5.89 | 0.0001 | LOC_Os06g19430 | expressed protein |
| Os.12993.1.S1_at | 4.72 | 0.0159 | LOC_Os05g07090 | Acyl-coenzyme A oxidase 4, peroxisomal, putative, expressed |
| Os.13014.1.S1_a_at | 2.66 | 0.0299 | LOC_Os10g38140 | Glutathione S-transferase, N-terminal domain containing protein |
| Os.13102.1.S1_at | 2.18 | 0.0080 | LOC_Os01g55650 | patatin, putative, expressed |
| Os.1316.1.S1_a_at | 2.01 | 0.0471 | LOC_Os01g58890 | Cysteine proteinase inhibitor-I, putative, expressed |
| Os.13253.1.S1_at | 2.28 | 0.0474 | LOC_Os01g59980 | Zn-finger in Ran binding protein |
| Os.13478.1.S1_at | 1.90 | 0.0255 | LOC_Os03g01030 | expressed protein |
| Os.13481.1.S1_at | 2.23 | 0.0309 | LOC_Os07g37250 | chloroplast inositol phosphatase, putative, expressed |
| Os.13493.1.S1_at | 4.92 | 0.0023 | LOC_Os05g49970 | Translation initiation factor IF-2, chloroplast precursor, |
| Os.13649.1.S1_at | 1.93 | 0.0100 | LOC_Os02g03560 | expressed protein |
| Os.137.1.S1_at | 1.95 | 0.0208 | LOC_Os07g29620 | Serine carboxypeptidase-like precursor, putative, expressed |
| Os.13717.1.S1_at | 21.35 | 0.0331 | LOC_Os10g15300 | expressed protein |
| Os.13734.1.S1_s_at | 3.06 | 0.0358 | LOC_Os08g21880 | retrotransposon, putative, centromere-specific |
| Os.13735.1.S1_at | 4.63 | 0.0143 | LOC_Os02g58150 | expressed protein |
| Os.1385.2.S1_x_at | 6.90 | 0.0068 | LOC_Os01g71670 | Glucan endo-1,3-beta-glucosidase GII precursor, putative, |
| Os.13874.1.S1_x_at | 3.64 | 0.0041 | LOC_Os10g42670 | xyloglucan endotransglucosylase/hydrolase protein 28 |
| Os.13927.1.S1_at | 4.08 | 0.0151 | LOC_Os02g35900 | thioredoxin family protein, putative, expressed |
| Os.13937.1.S1_at | 2.50 | 0.0226 | LOC_Os07g48810 | peptidyl-prolyl cis-trans isomerase, putative, expressed |
| Os.13995.1.S1_at | 5.64 | 0.0236 | LOC_Os05g34470 | expressed protein |
| Os.14046.1.S1_at | 2.05 | 0.0436 | LOC_Os04g51060 | expressed protein |
| Os.14058.1.S1_at | 3.54 | 0.0048 | LOC_Os09g19670 | retrotransposon protein, putative, unclassified |
| Os.14087.1.S1_at | 2.14 | 0.0487 | LOC_Os02g29550 | AP2 domain containing protein, expressed |
| Os.1411.1.S1_at | 9.96 | 0.0341 | LOC_Os01g10890 | CBL-interacting serine/threonine-protein kinase 15 |
| Os.14145.1.A1_at | 19.89 | 0.0013 | LOC_Os04g36720 | ferric reductase-like transmembrane component family protein |
| Os.14148.1.S1_at | 2.31 | 0.0101 | LOC_Os12g07650 | glutaredoxin-related protein, expressed |
| Os.14151.1.S1_at | 3.28 | 0.0067 | LOC_Os01g49310 | expressed protein |
| Os.14173.1.S1_at | 2.23 | 0.0337 | Os08g0305000 | Hypothetical protein. |
| Os.14176.1.S1_at | 2.61 | 0.0025 | LOC_Os05g01090 | Peroxisomal membrane anchor protein conserved region |
| Os.14200.2.S1_x_at | 1.70 | 0.0006 | LOC_Os10g24070 | expressed protein |
| Os.14216.1.S1_at | 2.81 | 0.0250 | LOC_Os01g74530 | protein phosphatase 2C, putative, expressed |
| Os.14245.1.S1_s_at | 4.46 | 0.0025 | LOC_Os03g05320 | expressed protein |
| Os.14251.1.S2_at | 1.71 | 0.0098 | LOC_Os03g14380 | Autophagy protein Apg9 containing protein, expressed |
| Os.14256.1.S1_a_at | 44.58 | 0.0338 | LOC_Os09g36930 | aquaporin PIP2.8, putative, expressed |
| Os.14271.1.S1_at | 3.40 | 0.0235 | LOC_Os07g43810 | 29 kDa ribonucleoprotein A, chloroplast precursor, putative |
| Os.14274.1.S1_at | 4.57 | 0.0115 | LOC_Os05g28500 | ubiquitin family protein, putative, expressed |
| Os.14283.1.A1_at | 8.44 | 0.0188 | LOC_Os02g06300 | GTP-binding protein LepA containing protein, expressed |
| Os.14286.1.S1_at | 3.12 | 0.0452 | LOC_Os02g50010 | APO protein 3, mitochondrial precursor, putative, expressed |
| Os.14326.1.S1_at | 1.59 | 0.0298 | LOC_Os04g35920 | Smg-4/UPF3 family protein, expressed |
| Os.14353.1.S1_s_at | 1.84 | 0.0179 | LOC_Os03g49710 | 30S ribosomal protein S13, putative, expressed |
| Os.14369.2.S1_at | 8.51 | 0.0405 | LOC_Os08g29170 | Quinone oxidoreductase, putative, expressed |
| Os.14404.1.S1_at | 3.44 | 0.0044 | LOC_Os02g38820 | expressed protein |
| Os.14409.1.S1_at | 1.70 | 0.0176 | Os06g0717400 | Pseudouridine synthase, Rlu family protein. |
| Os.14444.1.S1_at | 4.96 | 0.0457 | LOC_Os10g38700 | glutathione S-transferase GSTU6, putative, expressed |
| Os.14445.1.S1_at | 1.56 | 0.0288 | LOC_Os03g54760 | Mitochondrial carrier protein, putative, expressed |
| Os.1445.1.S1_at | 1.90 | 0.0253 | LOC_Os01g06660 | Pyruvate decarboxylase isozyme 1, putative, expressed |
| Os.14496.1.S1_at | 4.01 | 0.0247 | LOC_Os08g09270 | pentatricopeptide, putative, expressed |
| Os.14558.1.S1_at | 1.64 | 0.0084 | LOC_Os07g14350 | methyltransferase, putative, TIGR00027 family protein |
| Os.14585.1.S1_s_at | 2.94 | 0.0003 | Os03g0772300 | Conserved hypothetical protein. |
| Os.14690.1.S1_at | 1.54 | 0.0273 | LOC_Os06g44380 | Ubiquitin carboxyl-terminal hydrolase family protein |
| Os.14719.1.S1_at | 2.18 | 0.0312 | LOC_Os09g12770 | transfactor, putative, expressed |
| Os.14750.1.S1_x_at | 2.69 | 0.0066 | Os03g0848100 | Lysine decarboxylase family protein. |
| Os.14762.1.S1_x_at | 3.25 | 0.0025 | Os11g0706100 | Protein of unknown function DUF37 family protein. |
| Os.14762.2.S1_s_at | 2.62 | 0.0136 | LOC_Os11g47930 | expressed protein |
| Os.14762.2.S1_x_at | 2.30 | 0.0199 | LOC_Os11g47930 | expressed protein |
| Os.14762.3.S1_x_at | 2.47 | 0.0046 | LOC_Os11g47930 | expressed protein |
| Os.14793.1.S1_at | 7.05 | 0.0200 | LOC_Os02g49570 | Peptidase family U7 containing protein, expressed |
| Os.14817.1.S1_a_at | 4.26 | 0.0045 | LOC_Os10g36860 | CRS1/YhbY domain containing protein, expressed |
| Os.14820.1.S2_s_at | 2.74 | 0.0486 | LOC_Os05g51150 | Sigma-70, region 4 family protein, expressed |
| Os.14831.1.S1_at | 2.79 | 0.0045 | LOC_Os09g04440 | DNA-binding protein, putative, expressed |
| Os.14870.1.S1_at | 3.89 | 0.0337 | LOC_Os03g34040 | ribosomal protein S5 containing protein, expressed |
| Os.14872.1.S1_at | 2.36 | 0.0010 | LOC_Os04g33210 | ERD1 protein, chloroplast precursor, putative, expressed |
| Os.14874.1.S1_at | 2.46 | 0.0048 | LOC_Os01g47430 | expressed protein |
| Os.14875.1.S1_at | 3.30 | 0.0089 | LOC_Os10g42500 | plastid-lipid associated protein 3, chloroplast precursor, |
| Os.14877.1.S1_at | 6.97 | 0.0340 | LOC_Os03g10180 | expressed protein |
| Os.14911.1.S1_at | 1.82 | 0.0004 | LOC_Os08g16480 | ATPase, AFG1 family protein, expressed |
| Os.14915.1.S1_a_at | 2.61 | 0.0123 | LOC_Os12g37910 | GDSL-like Lipase/Acylhydrolase family protein, expressed |
| Os.14935.1.S1_at | 4.43 | 0.0100 | LOC_Os10g09990 | Cytokinin-O-glucosyltransferase 3, putative, expressed |
| Os.14941.2.S1_a_at | 3.19 | 0.0068 | LOC_Os12g09570 | AIG1 family protein, expressed |
| Os.14956.1.S1_at | 2.28 | 0.0301 | LOC_Os11g32360 | expressed protein |
| Os.14960.1.S1_at | 2.93 | 0.0015 | Os08g0431100 | Bromo adjacent region domain containing protein. |
| Os.14984.1.S1_a_at | 1.74 | 0.0189 | LOC_Os03g03610 | 1,3-beta-glucan synthase component family protein, expressed |
| Os.14992.1.S1_x_at | 2.58 | 0.0004 | LOC_Os05g05210 | LEC14B protein, putative, expressed |
| Os.1500.2.S1_x_at | 3.11 | 0.0132 | LOC_Os01g43070 | oxygen-evolving complex, putative, expressed |
| Os.15007.1.S1_at | 2.84 | 0.0466 | unknown | unknown |
| Os.15041.1.S1_a_at | 2.74 | 0.0238 | LOC_Os04g32050 | transposon protein, putative, unclassified, expressed |
| Os.15046.1.S1_at | 1.78 | 0.0133 | LOC_Os02g33230 | expressed protein |
| Os.15062.1.S1_a_at | 43.54 | 0.0191 | LOC_Os01g22980 | Serine carboxypeptidase family protein, expressed |
| Os.15065.1.S1_at | 4.20 | 0.0465 | LOC_Os06g45370 | fructose-1,6-bisphosphatase family protein, expressed |
| Os.15071.2.S1_s_at | 5.95 | 0.0497 | LOC_Os02g51470 | ATP synthase delta chain, chloroplast precursor, putative, |
| Os.15099.1.S1_at | 1.97 | 0.0252 | LOC_Os08g23430 | glycoside hydrolase starch-binding domain-containing protein |
| Os.15117.1.S1_at | 2.47 | 0.0210 | LOC_Os01g56200 | Regulatory protein NPR1, putative, expressed |
| Os.15134.1.S1_at | 2.40 | 0.0153 | LOC_Os01g61560 | phosphatidate cytidylyltransferase family protein |
| Os.15142.1.S1_at | 7.15 | 0.0192 | LOC_Os04g49350 | pentatricopeptide, putative, expressed |
| Os.15183.1.S1_at | 3.95 | 0.0074 | LOC_Os01g40710 | expressed protein |
| Os.15189.1.S1_at | 2.35 | 0.0016 | LOC_Os02g35470 | Calmodulin-binding protein, putative, expressed |
| Os.15194.1.S1_at | 6.39 | 0.0015 | LOC_Os01g37130 | Protein phosphatase 2C PPH1, putative, expressed |
| Os.1521.1.S1_at | 2.23 | 0.0365 | LOC_Os01g22630 | YT521-B-like family protein, expressed |
| Os.15221.1.S1_a_at | 4.49 | 0.0040 | Os05g0334400 | Heat shock protein DnaJ family protein |
| Os.15238.1.S1_at | 2.50 | 0.0269 | LOC_Os03g20310 | Transcription factor HBP-1b, putative, expressed |
| Os.15243.2.S1_x_at | 10.42 | 0.0083 | LOC_Os02g01340 | Ferredoxin-NADP reductase, leaf isozyme |
| Os.15247.1.S1_s_at | 2.82 | 0.0420 | LOC_Os12g39630 | Serine/threonine-protein kinase SAPK9, putative, expressed |
| Os.15283.1.S1_at | 4.48 | 0.0007 | LOC_Os04g57310 | Uncharacterized conserved protein, putative, expressed |
| Os.15295.3.S1_x_at | 2.46 | 0.0172 | LOC_Os01g70460 | expressed protein |
| Os.15338.1.S1_a_at | 2.12 | 0.0301 | LOC_Os01g34060 | myb-like DNA-binding domain, SHAQKYF class family protein, |
| Os.15427.1.S1_at | 4.57 | 0.0120 | LOC_Os03g64020 | expressed protein |
| Os.15428.1.S1_at | 5.04 | 0.0211 | Os01g0702000 | Conserved hypothetical protein. |
| Os.15436.1.S1_at | 3.60 | 0.0228 | LOC_Os08g15460 | Preprotein translocase secY subunit, chloroplast precursor |
| Os.15570.1.S1_at | 4.68 | 0.0005 | LOC_Os07g32630 | UDP-glucoronosyl and UDP-glucosyl transferase family protein |
| Os.15600.1.S1_a_at | 6.88 | 0.0015 | LOC_Os12g07110 | AMP-binding enzyme family protein, expressed |
| Os.15618.1.S1_a_at | 7.93 | 0.0103 | LOC_Os03g18550 | Mitochondrial carrier protein, expressed |
| Os.15622.1.S1_at | 1.65 | 0.0291 | LOC_Os11g01190 | expressed protein |
| Os.15641.1.S1_at | 12.08 | 0.0412 | LOC_Os01g73500 | expressed protein |
| Os.15707.1.S1_at | 2.67 | 0.0123 | LOC_Os08g40430 | mitochondrial transcription termination factor, putative, |
| Os.15711.1.S1_at | 3.46 | 0.0123 | LOC_Os09g28310 | bZIP transcription factor family protein, expressed |
| Os.15722.1.S1_s_at | 3.76 | 0.0092 | LOC_Os06g39900 | Agenet domain containing protein, expressed |
| Os.15729.1.S1_at | 2.33 | 0.0250 | LOC_Os04g49570 | Glutamate receptor 3.3 precursor, putative, expressed |
| Os.15734.1.S1_at | 4.00 | 0.0057 | LOC_Os08g25700 | expressed protein |
| Os.15977.1.S1_at | 1.67 | 0.0014 | LOC_Os03g06880 | pfkB-type carbohydrate kinase family protein |
| Os.15988.1.S1_at | 1.85 | 0.0169 | LOC_Os06g27770 | Isoflavone reductase homolog, putative, expressed |
| Os.15991.1.S1_at | 2.30 | 0.0256 | LOC_Os01g51860 | violaxanthin de-epoxidase, putative, expressed |
| Os.16014.1.S1_at | 2.13 | 0.0054 | LOC_Os04g47860 | INDETERMINATE-related protein 9, putative, expressed |
| Os.1605.1.S1_at | 4.95 | 0.0000 | LOC_Os04g37630 | expressed protein |
| Os.1606.2.S1_a_at | 3.89 | 0.0104 | LOC_Os01g31680 | Sec-independent protein translocase TatC containing protein |
| Os.16067.1.S1_at | 3.20 | 0.0244 | LOC_Os08g20020 | PB1 domain containing protein, expressed |
| Os.16076.1.S1_s_at | 3.44 | 0.0124 | LOC_Os06g33340 | expressed protein |
| Os.16119.1.S1_at | 2.75 | 0.0289 | LOC_Os03g05310 | Pheophorbide a oxygenase, chloroplast precursor, putative, |
| Os.16220.1.A1_at | 5.67 | 0.0213 | LOC_Os06g03990 | aminotransferase ACS12, putative, expressed |
| Os.16261.1.S1_at | 2.33 | 0.0173 | LOC_Os06g30910 | CAAX amino terminal protease family protein, expressed |
| Os.16309.1.S1_at | 5.24 | 0.0303 | LOC_Os09g25550 | puative protein, putative, expressed |
| Os.16318.1.S1_at | 1.69 | 0.0100 | LOC_Os12g22030 | Serine hydroxymethyltransferase, mitochondrial precursor, |
| Os.16324.1.S1_at | 4.80 | 0.0430 | LOC_Os12g38870 | expressed protein |
| Os.16333.1.S1_at | 2.86 | 0.0001 | LOC_Os04g40310 | isocitrate dehydrogenase, NAD-dependent family protein |
| Os.16429.1.S1_at | 2.75 | 0.0200 | LOC_Os01g52630 | Regulator of chromosome condensation family protein |
| Os.16460.1.S1_at | 1.97 | 0.0251 | LOC_Os05g23700 | DNA-binding storekeeper protein, putative, expressed |
| Os.16530.1.S1_at | 1.51 | 0.0486 | LOC_Os01g05420 | IWS1 C-terminus family protein, expressed |
| Os.16631.1.S1_x_at | 2.20 | 0.0370 | LOC_Os11g19460 | haloacid dehalogenase-like hydrolase family protein |
| Os.1665.1.S1_a_at | 2.52 | 0.0096 | LOC_Os01g07300 | expressed protein |
| Os.16692.1.S1_x_at | 2.99 | 0.0419 | LOC_Os10g35810 | Thylakoid lumenal 17.4 kDa protein, chloroplast precursor, |
| Os.16709.1.S1_at | 1.62 | 0.0315 | LOC_Os01g16080 | expressed protein |
| Os.16724.1.S1_at | 4.55 | 0.0005 | Os07g0502200 | Multi antimicrobial extrusion protein MatE family protein. |
| Os.16736.1.S1_s_at | 1.78 | 0.0479 | LOC_Os01g39270 | NAD binding domain of 6-phosphogluconate dehydrogenase |
| Os.16757.1.S1_s_at | 2.27 | 0.0106 | LOC_Os07g41640 | Agenet domain containing protein, expressed |
| Os.16765.1.S1_at | 2.52 | 0.0496 | LOC_Os05g07860 | UvrB/uvrC motif family protein, expressed |
| Os.16772.1.S1_at | 4.23 | 0.0065 | LOC_Os08g27010 | expressed protein |
| Os.16806.1.S1_at | 2.79 | 0.0263 | Os02g0161400 | Non-protein coding transcript, uncharacterized transcript. |
| Os.16838.1.S1_at | 6.41 | 0.0256 | LOC_Os03g51960 | UPF0308 protein, chloroplast precursor, putative, expressed |
| Os.16854.1.S1_at | 2.56 | 0.0251 | LOC_Os04g52500 | lecithine cholesterol acyltransferase, putative, expressed |
| Os.16867.1.S1_at | 1.51 | 0.0262 | Os08g0300200 | C2 domain containing protein. |
| Os.16883.1.S1_a_at | 2.12 | 0.0441 | LOC_Os02g50840 | BAH domain containing protein, expressed |
| Os.16884.1.S1_at | 2.04 | 0.0100 | LOC_Os03g50870 | expressed protein |
| Os.16894.1.S1_at | 2.40 | 0.0424 | LOC_Os05g32140 | UDP-sulfoquinovose synthase, chloroplast precursor |
| Os.16918.1.S1_at | 2.49 | 0.0220 | LOC_Os12g37890 | expressed protein |
| Os.16927.1.S1_at | 1.73 | 0.0306 | LOC_Os02g56170 | PAP-specific phosphatase, putative, expressed |
| Os.16966.1.S1_s_at | 8.44 | 0.0152 | LOC_Os11g13850 | Rieske domain containing protein, expressed |
| Os.16968.1.S1_at | 2.97 | 0.0076 | LOC_Os03g03990 | chromo' domain containing protein, expressed |
| Os.16976.1.S1_at | 2.06 | 0.0415 | Os05g0115800 | MAP kinase phosphatase |
| Os.16983.1.S1_at | 1.87 | 0.0117 | LOC_Os04g30890 | gonidia forming protein GlsA, putative, expressed |
| Os.17002.1.S1_at | 7.01 | 0.0007 | Os01g0362100 | Esterase/lipase/thioesterase domain containing protein. |
| Os.17072.1.S1_at | 1.82 | 0.0032 | LOC_Os06g06190 | ATPase, putative, expressed |
| Os.17074.1.S1_at | 2.49 | 0.0421 | LOC_Os03g44200 | Beclin-1, putative, expressed |
| Os.17091.1.S1_at | 3.74 | 0.0017 | LOC_Os08g41040 | expressed protein |
| Os.17111.2.S1_x_at | 18.53 | 0.0250 | LOC_Os04g48460 | Cytochrome P450 family protein, expressed |
| Os.17122.1.S1_at | 4.48 | 0.0027 | LOC_Os05g49380 | Protease Do-like 1, chloroplast precursor, putative |
| Os.17125.1.S1_at | 4.49 | 0.0220 | LOC_Os08g41090 | Peroxidase family protein, expressed |
| Os.17149.1.S1_at | 2.65 | 0.0347 | LOC_Os03g60910 | pentatricopeptide, putative, expressed |
| Os.17149.2.S1_x_at | 2.57 | 0.0161 | LOC_Os03g60910 | pentatricopeptide, putative, expressed |
| Os.17158.1.S1_at | 12.48 | 0.0251 | LOC_Os01g70490 | Potassium transporter 5, putative, expressed |
| Os.17162.2.S1_x_at | 2.01 | 0.0299 | LOC_Os01g57110 | SNF2 domain-containing protein, putative, expressed |
| Os.17198.1.S1_at | 2.54 | 0.0287 | LOC_Os06g50130 | expressed protein |
| Os.17210.1.S1_at | 2.35 | 0.0009 | LOC_Os02g57750 | expressed protein |
| Os.17215.1.S2_at | 1.79 | 0.0154 | LOC_Os02g08120 | Calmodulin-binding protein, putative, expressed |
| Os.17226.1.S1_s_at | 1.88 | 0.0102 | LOC_Os01g45750 | bile acid transporter family protein, expressed |
| Os.17227.1.S1_at | 3.00 | 0.0380 | LOC_Os01g69060 | hydrolase, putative, expressed |
| Os.17268.1.S1_at | 4.09 | 0.0022 | LOC_Os09g33470 | Peroxisomal membrane carrier protein, putative, expressed |
| Os.17279.1.S1_s_at | 4.65 | 0.0005 | LOC_Os06g01400 | glycyl-tRNA synthetase beta subunit family protein |
| Os.17282.1.S1_at | 4.99 | 0.0058 | LOC_Os11g08670 | ATP-NAD kinase family protein, expressed |
| Os.17351.1.A1_at | 2.15 | 0.0143 | LOC_Os03g22780 | isoflavone reductase, putative, expressed |
| Os.17360.1.A1_at | 4.96 | 0.0111 | LOC_Os02g15660 | tetratricopeptide repeat, putative, expressed |
| Os.17368.1.S1_at | 4.59 | 0.0245 | LOC_Os02g52420 | methionine aminopeptidase, putative, expressed |
| Os.17369.1.S1_a_at | 5.41 | 0.0003 | LOC_Os04g37920 | Cryptochrome 1 apoprotein, putative, expressed |
| Os.17377.1.S1_at | 3.01 | 0.0417 | LOC_Os04g41850 | RWP-RK domain containing protein, expressed |
| Os.17380.1.S1_x_at | 1.81 | 0.0159 | Os09g0109800 | Conserved hypothetical protein. |
| Os.17380.4.A1_x_at | 2.19 | 0.0064 | Os09g0109800 | Conserved hypothetical protein. |
| Os.17390.1.S1_at | 3.10 | 0.0160 | LOC_Os02g35010 | mitogen-activated kinase kinase kinase alpha, putative |
| Os.17391.1.S1_at | 1.59 | 0.0291 | LOC_Os05g10810 | MBOAT family protein, expressed |
| Os.17393.1.S1_at | 5.40 | 0.0118 | LOC_Os01g12810 | leaf protein, putative, expressed |
| Os.17419.1.S1_at | 2.56 | 0.0398 | LOC_Os10g09850 | 39 kDa EF-Hand containing protein, putative, expressed |
| Os.17432.1.S1_at | 1.92 | 0.0441 | LOC_Os04g43700 | Glycosyl transferase family 8 protein, expressed |
| Os.17435.2.S1_at | 3.86 | 0.0481 | Os11g0427500 | Transposase |
| Os.17440.1.S1_at | 2.34 | 0.0348 | LOC_Os04g44610 | ABC-2 type transporter family protein, expressed |
| Os.17444.1.S1_a_at | 1.55 | 0.0290 | LOC_Os04g37540 | expressed protein |
| Os.17449.1.A1_at | 6.93 | 0.0113 | LOC_Os01g69070 | Auxin Efflux Carrier family protein, expressed |
| Os.17487.1.S1_at | 3.71 | 0.0063 | LOC_Os03g50310 | CCT motif family protein, expressed |
| Os.17490.1.A1_at | 4.71 | 0.0013 | Os03g0654600 | Short-chain dehydrogenase/reductase SDR family protein. |
| Os.17490.2.A1_a_at | 3.36 | 0.0115 | Os03g0654600 | Short-chain dehydrogenase/reductase SDR family protein. |
| Os.17501.1.A1_at | 8.04 | 0.0149 | LOC_Os09g38410 | Major Facilitator Superfamily protein, expressed |
| Os.17502.1.S1_at | 5.49 | 0.0028 | LOC_Os11g32880 | CarD-like transcriptional regulator family protein |
| Os.17540.1.S1_at | 2.25 | 0.0359 | LOC_Os10g37980 | prephenate dehydratase family protein, expressed |
| Os.17547.1.S1_at | 2.66 | 0.0471 | LOC_Os08g06640 | expressed protein |
| Os.17549.1.S1_at | 3.69 | 0.0136 | LOC_Os04g44340 | expressed protein |
| Os.17566.1.S1_at | 7.63 | 0.0206 | LOC_Os02g18450 | GTP-binding protein TypA/BipA homolog, putative, expressed |
| Os.17597.1.S1_at | 2.01 | 0.0385 | LOC_Os02g40040 | expressed protein |
| Os.17618.1.S1_at | 1.67 | 0.0072 | LOC_Os03g43570 | UBX domain-containing protein 1, putative, expressed |
| Os.17625.1.S1_at | 3.32 | 0.0143 | LOC_Os07g41710 | expressed protein |
| Os.17640.1.S1_at | 2.49 | 0.0049 | LOC_Os03g54150 | expressed protein |
| Os.17652.1.S1_s_at | 2.97 | 0.0307 | LOC_Os03g43720 | major facilitator superfamily protein, expressed |
| Os.17666.1.S1_at | 1.71 | 0.0015 | LOC_Os05g48660 | Protein kinase domain containing protein, expressed |
| Os.17679.1.S1_at | 5.88 | 0.0002 | LOC_Os04g52330 | expressed protein |
| Os.17692.1.S1_at | 3.09 | 0.0168 | LOC_Os10g35140 | Integral membrane protein DUF6 containing protein, expressed |
| Os.17708.1.S1_at | 4.06 | 0.0012 | LOC_Os01g48380 | pentatricopeptide, putative, expressed |
| Os.17714.1.S1_at | 2.17 | 0.0216 | LOC_Os03g09080 | Ubiquitin carboxyl-terminal hydrolase 4, putative, expressed |
| Os.17721.1.S1_at | 6.35 | 0.0032 | Os09g0493600 | Iojap-related protein family protein. |
| Os.17741.1.S1_at | 2.79 | 0.0242 | LOC_Os01g22900 | beta-fructofuranosidase, putative, expressed |
| Os.17754.1.S1_at | 4.38 | 0.0235 | LOC_Os09g39680 | Major Facilitator Superfamily protein, expressed |
| Os.17768.1.S1_at | 9.49 | 0.0043 | LOC_Os06g41930 | zinc-binding family protein, putative, expressed |
| Os.17778.1.S1_at | 3.66 | 0.0200 | LOC_Os04g58410 | MutS domain V family protein, expressed |
| Os.17779.1.S1_at | 4.43 | 0.0086 | LOC_Os05g05140 | expressed protein |
| Os.17783.1.S1_at | 2.49 | 0.0342 | LOC_Os05g41000 | Endonuclease/Exonuclease/phosphatase family protein |
| Os.17828.1.S1_s_at | 1.83 | 0.0452 | LOC_Os03g12550 | expressed protein |
| Os.17851.1.S1_at | 1.67 | 0.0078 | LOC_Os05g23610 | Protein phosphatase inhibitor 2 containing protein |
| Os.17876.1.S1_at | 2.58 | 0.0098 | LOC_Os03g59450 | transporter, putative, expressed |
| Os.17893.1.S1_at | 1.70 | 0.0046 | LOC_Os02g02480 | Helix-loop-helix DNA-binding domain containing protein |
| Os.17898.1.S1_at | 2.47 | 0.0413 | LOC_Os02g42330 | Nitrilase 4, putative, expressed |
| Os.17909.1.S1_a_at | 5.25 | 0.0202 | LOC_Os12g08260 | 2-oxoisovalerate dehydrogenase alpha subunit, mitochondrial |
| Os.17917.1.S1_at | 4.18 | 0.0017 | LOC_Os05g11090 | Thioredoxin-like 2, chloroplast precursor, putative |
| Os.17919.1.S1_at | 1.90 | 0.0235 | LOC_Os04g52130 | Coproporphyrinogen III oxidase, chloroplast precursor |
| Os.17937.1.S1_a_at | 2.16 | 0.0032 | LOC_Os04g21110 | Phosphoribulokinase/Uridine kinase family protein |
| Os.17959.1.S1_a_at | 3.70 | 0.0215 | LOC_Os08g34210 | NADP-dependent glyceraldehyde-3-phosphate dehydrogenase |
| Os.1798.1.S1_at | 3.56 | 0.0381 | LOC_Os01g05080 | Thylakoid lumenal 15 kDa protein, chloroplast precursor |
| Os.18054.1.S1_s_at | 2.97 | 0.0077 | LOC_Os04g31270 | ribosome-associated GTPase YjeQ family protein, expressed |
| Os.18089.1.S1_at | 1.63 | 0.0422 | Os08g0344600 | Phosphate/phosphoenolpyruvate translocator. |
| Os.18168.1.S1_at | 5.33 | 0.0044 | LOC_Os04g51940 | YT521-B-like family protein, expressed |
| Os.18211.1.S1_at | 6.16 | 0.0226 | Os03g0122000 | Protein kinase domain containing protein. |
| Os.18211.2.S1_x_at | 5.06 | 0.0100 | LOC_Os03g02980 | Protein kinase domain containing protein, expressed |
| Os.18305.1.S2_at | 5.47 | 0.0012 | LOC_Os05g50550 | Polyprenyl synthetase family protein, expressed |
| Os.18321.1.S1_at | 2.00 | 0.0004 | LOC_Os02g44780 | Polyprenyl synthetase family protein, expressed |
| Os.18333.1.S1_at | 1.66 | 0.0086 | LOC_Os08g04390 | bHLH transcription factor, putative, expressed |
| Os.18384.1.S1_at | 7.74 | 0.0140 | LOC_Os03g39830 | expressed protein |
| Os.18469.1.S1_at | 1.61 | 0.0225 | LOC_Os09g04800 | BadF/BadG/BcrA/BcrD ATPase family protein, expressed |
| Os.18490.3.S1_at | 2.15 | 0.0153 | LOC_Os03g47610 | Thiamine biosynthesis protein thiC, putative, expressed |
| Os.18589.1.S1_at | 1.96 | 0.0159 | LOC_Os04g33280 | expressed protein |
| Os.18600.1.S1_at | 2.13 | 0.0404 | LOC_Os06g29120 | protein kinase family protein, putative, expressed |
| Os.18608.1.S1_at | 3.13 | 0.0016 | Os12g0562300 | TPR-like domain containing protein. |
| Os.18653.1.S1_at | 2.00 | 0.0004 | Os03g0363400 | Protein of unknown function UPF0153 family protein. |
| Os.18663.1.S1_at | 2.48 | 0.0226 | LOC_Os09g37230 | protein kinase family protein, putative, expressed |
| Os.18774.1.S1_s_at | 2.16 | 0.0383 | Os01g0214400 | Protein of unknown function DUF455 family protein. |
| Os.18786.1.S1_at | 8.94 | 0.0099 | LOC_Os03g05470 | Protein kinase domain containing protein, expressed |
| Os.18818.1.S1_a_at | 5.27 | 0.0121 | LOC_Os01g12710 | oxidoreductase, short chain dehydrogenase/reductase family |
| Os.18825.1.S1_at | 2.45 | 0.0238 | LOC_Os02g53860 | subtilase family protein, putative, expressed |
| Os.18850.1.S1_at | 3.83 | 0.0186 | LOC_Os10g30870 | expressed protein |
| Os.18856.1.S1_at | 12.88 | 0.0246 | LOC_Os04g02050 | Bifunctional 3'-phosphoadenosine 5'-phosphosulfate synthethase |
| Os.18863.1.S1_at | 2.40 | 0.0140 | LOC_Os01g53150 | GTP-binding protein, putative, expressed |
| Os.18913.1.S1_x_at | 12.34 | 0.0001 | LOC_Os05g39800 | Nodulin-like family protein, expressed |
| Os.18923.1.S1_a_at | 1.94 | 0.0079 | LOC_Os09g31400 | ETHYLENE-INSENSITIVE3-like 3 protein, putative, expressed |
| Os.18927.1.S1_at | 2.90 | 0.0043 | LOC_Os05g25450 | protein kinase family protein, putative, expressed |
| Os.18955.1.S1_at | 3.57 | 0.0130 | LOC_Os06g51260 | myb-like DNA-binding domain, SHAQKYF class family protein, |
| Os.18958.1.S1_at | 8.80 | 0.0351 | LOC_Os06g49250 | POT family protein, expressed |
| Os.1899.1.S1_at | 6.47 | 0.0154 | LOC_Os01g06210 | PrMC3, putative |
| Os.19007.1.S1_at | 2.72 | 0.0212 | LOC_Os06g50030 | CDPK-related protein kinase, putative, expressed |
| Os.19014.1.S1_at | 3.41 | 0.0096 | LOC_Os01g23680 | expressed protein |
| Os.19016.1.S1_at | 1.62 | 0.0225 | LOC_Os01g68630 | expressed protein |
| Os.19035.1.S1_at | 3.17 | 0.0034 | LOC_Os01g61400 | expressed protein |
| Os.19036.1.S1_at | 3.00 | 0.0152 | LOC_Os06g15700 | expressed protein |
| Os.19044.1.S1_at | 21.80 | 0.0193 | LOC_Os10g04520 | expressed protein |
| Os.19084.1.S1_at | 1.76 | 0.0477 | LOC_Os04g55090 | pentatricopeptide, putative, expressed |
| Os.19100.1.S1_at | 8.51 | 0.0457 | LOC_Os03g53300 | expressed protein |
| Os.19131.1.A1_at | 4.19 | 0.0063 | LOC_Os09g28910 | Carbonic anhydrase family protein, expressed |
| Os.19141.1.S1_at | 9.50 | 0.0002 | LOC_Os01g47630 | expressed protein |
| Os.19155.1.S1_at | 3.47 | 0.0293 | LOC_Os01g37870 | pentatricopeptide, putative, expressed |
| Os.19165.1.S1_at | 4.27 | 0.0185 | LOC_Os11g29900 | expressed protein |
| Os.19279.2.S1_x_at | 1.91 | 0.0254 | LOC_Os01g72860 | MAC/Perforin domain containing protein, expressed |
| Os.19307.1.S1_at | 1.77 | 0.0104 | LOC_Os08g03430 | expressed protein |
| Os.19308.1.S1_x_at | 2.69 | 0.0365 | LOC_Os03g58970 | expressed protein |
| Os.19310.1.S1_at | 2.76 | 0.0151 | LOC_Os09g38070 | expressed protein |
| Os.19325.1.S1_at | 2.39 | 0.0036 | LOC_Os09g11520 | Ycf20, putative, expressed |
| Os.19335.1.S1_at | 3.32 | 0.0059 | LOC_Os06g36080 | Kinesin motor domain containing protein, expressed |
| Os.19361.1.S2_at | 9.96 | 0.0478 | LOC_Os04g45290 | Beta-fructofuranosidase 1 precursor, putative, expressed |
| Os.19378.1.S1_at | 4.00 | 0.0222 | Os02g0291500 | Conserved hypothetical protein. |
| Os.19474.1.S1_a_at | 3.12 | 0.0294 | LOC_Os03g19560 | TPR Domain containing protein, expressed |
| Os.19539.1.S1_at | 3.96 | 0.0012 | Os02g0704300 | Hypothetical protein. |
| Os.19563.1.S1_at | 2.28 | 0.0015 | LOC_Os09g28220 | expressed protein |
| Os.19622.1.S1_at | 2.33 | 0.0111 | LOC_Os02g21430 | RNA recognition motif 2 family protein, expressed |
| Os.19627.1.S1_a_at | 8.70 | 0.0258 | LOC_Os10g10170 | pentatricopeptide, putative, expressed |
| Os.19752.1.S1_at | 3.54 | 0.0439 | LOC_Os10g40030 | oxidoreductase, short chain dehydrogenase/reductase family |
| Os.19755.2.S1_a_at | 3.96 | 0.0006 | LOC_Os01g37690 | Vacuolar cation/proton exchanger 1a, putative, expressed |
| Os.1977.3.S1_x_at | 33.15 | 0.0410 | LOC_Os01g02600 | Ser/Thr receptor-like kinase |
| Os.19777.1.A1_at | 1.85 | 0.0134 | LOC_Os01g13770 | Triose phosphate/phosphate translocator |
| Os.19817.1.S1_at | 1.87 | 0.0076 | LOC_Os03g21370 | expressed protein |
| Os.1983.1.S1_at | 2.89 | 0.0118 | Os02g0182800 | Homeodomain transcription factor KNAT7. |
| Os.19863.1.S1_a_at | 7.37 | 0.0002 | LOC_Os04g36560 | unspliced-genomic chloride channel protein,putative, expressed |
| Os.19896.2.S1_x_at | 42.08 | 0.0410 | LOC_Os06g48800 | Cation transport protein, expressed |
| Os.20050.1.A1_at | 5.57 | 0.0232 | LOC_Os09g24460 | expressed protein |
| Os.20068.1.S1_at | 3.22 | 0.0205 | LOC_Os06g30380 | expressed protein |
| Os.20075.1.S1_at | 2.73 | 0.0231 | LOC_Os03g01570 | Uncharacterised protein family containing protein, expressed |
| Os.20078.1.S1_at | 93.28 | 0.0168 | Os07g0601000 | NADPH HC toxin reductase (Fragment). |
| Os.20084.1.S1_at | 2.94 | 0.0001 | LOC_Os02g39160 | 4-hydroxy-3-methylbut-2-en-1-yl diphosphate synthase family |
| Os.2010.1.S1_at | 5.03 | 0.0329 | LOC_Os01g40870 | Aldehyde dehydrogenase, mitochondrial precursor, putative, |
| Os.20187.2.S1_at | 6.02 | 0.0263 | LOC_Os01g61010 | Nodulin-like family protein, expressed |
| Os.20203.1.S1_at | 8.44 | 0.0379 | LOC_Os01g10210 | expressed protein |
| Os.20204.2.S1_a_at | 2.56 | 0.0027 | LOC_Os02g12350 | histone deacetylase, putative, expressed |
| Os.20299.1.S1_a_at | 1.94 | 0.0098 | Os07g0646300 | Conserved hypothetical protein |
| Os.20299.4.S1_x_at | 1.97 | 0.0039 | LOC_Os07g45520 | hypothetical protein |
| Os.20351.1.S1_at | 5.27 | 0.0022 | LOC_Os03g63360 | expressed protein |
| Os.20361.1.A1_at | 6.06 | 0.0064 | LOC_Os09g31300 | Helix-loop-helix DNA-binding domain containing protein |
| Os.20397.1.S1_a_at | 2.99 | 0.0177 | LOC_Os03g52460 | Glucose-1-phosphate adenylyltransferase large subunit 3 |
| Os.20424.1.S1_s_at | 1.84 | 0.0121 | LOC_Os04g46100 | expressed protein |
| Os.20461.1.S1_at | 3.18 | 0.0445 | LOC_Os03g19760 | HAD-superfamily hydrolase, subfamily IA |
| Os.20465.1.S1_at | 3.98 | 0.0063 | LOC_Os03g21560 | possible Photosystem II reaction center Psb27 protein |
| Os.20473.1.S1_s_at | 2.80 | 0.0332 | LOC_Os05g33620 | expressed protein |
| Os.20476.2.S1_a_at | 3.58 | 0.0126 | LOC_Os06g19960 | Aconitase family containing protein |
| Os.20521.1.S1_at | 11.33 | 0.0112 | LOC_Os03g54040 | 50S ribosomal protein L6, putative, expressed |
| Os.20538.2.S1_x_at | 2.14 | 0.0134 | LOC_Os01g21460 | expressed protein |
| Os.20540.1.S1_at | 3.34 | 0.0103 | LOC_Os02g47800 | monodehydroascorbate reductase, cytoplasmic isoform 2 |
| Os.20541.1.S1_at | 4.45 | 0.0090 | LOC_Os03g37840 | Potassium transporter 1, putative, expressed |
| Os.20570.1.S1_x_at | 2.10 | 0.0312 | LOC_Os06g02500 | Superoxide dismutase, chloroplast, putative, expressed |
| Os.20595.1.S1_a_at | 1.73 | 0.0470 | LOC_Os07g01550 | Leucine Rich Repeat family protein, expressed |
| Os.20603.1.S1_at | 2.76 | 0.0049 | LOC_Os06g28970 | BolA-like protein, expressed |
| Os.20603.1.S1_s_at | 2.73 | 0.0396 | Os06g0484600 | Pherophorin-S precursor. |
| Os.20638.1.S2_at | 5.43 | 0.0177 | LOC_Os03g57110 | expressed protein |
| Os.20642.1.S1_at | 3.52 | 0.0283 | LOC_Os02g08540 | expressed protein |
| Os.20672.1.S1_s_at | 1.77 | 0.0125 | LOC_Os08g37610 | expressed protein |
| Os.20672.1.S2_at | 1.92 | 0.0042 | LOC_Os08g37610 | expressed protein |
| Os.20693.1.S1_at | 2.36 | 0.0334 | LOC_Os11g41910 | small GTP-binding protein domain containing protein |
| Os.20717.1.S1_at | 4.53 | 0.0376 | LOC_Os01g61460 | expressed protein |
| Os.20757.1.S1_a_at | 2.70 | 0.0467 | LOC_Os06g42790 | Nudix hydrolase 2, putative, expressed |
| Os.20810.4.S1_at | 2.76 | 0.0076 | LOC_Os08g31910 | expressed protein |
| Os.20829.1.S1_at | 2.68 | 0.0384 | LOC_Os02g55630 | CobW/P47K family protein, expressed |
| Os.20835.1.S1_at | 2.28 | 0.0443 | LOC_Os06g33570 | Cyclic nucleotide-gated ion channel 1, putative, expressed |
| Os.20864.1.A1_at | 2.03 | 0.0069 | LOC_Os07g44050 | expressed protein |
| Os.20884.1.S1_at | 1.89 | 0.0453 | LOC_Os05g01760 | expressed protein |
| Os.20911.1.S1_at | 3.62 | 0.0087 | LOC_Os04g58780 | pentatricopeptide, putative, expressed |
| Os.20934.1.A1_at | 8.21 | 0.0495 | LOC_Os03g07200 | expressed protein |
| Os.21030.1.S1_at | 2.11 | 0.0033 | LOC_Os10g38030 | Per1-like family protein, expressed |
| Os.21038.1.S1_s_at | 2.63 | 0.0207 | LOC_Os03g56420 | dehydrogenase, putative, expressed |
| Os.21061.1.S1_at | 2.64 | 0.0270 | LOC_Os07g47110 | Tubby protein, putative, expressed |
| Os.21073.1.S1_at | 7.63 | 0.0229 | LOC_Os04g50860 | expressed protein |
| Os.21088.1.S1_at | 9.98 | 0.0234 | LOC_Os07g07480 | HNH endonuclease domain-containing protein |
| Os.21178.1.S1_at | 9.56 | 0.0436 | LOC_Os01g42280 | pentatricopeptide, putative, expressed |
| Os.21192.2.S1_at | 3.63 | 0.0103 | Os01g0680600 | Conserved hypothetical protein. |
| Os.21192.3.S1_x_at | 2.03 | 0.0198 | LOC_Os01g48820 | expressed protein |
| Os.21210.1.S1_at | 5.46 | 0.0037 | LOC_Os03g38230 | membrane-associated zinc metalloprotease family protein |
| Os.21230.1.S1_at | 4.78 | 0.0177 | LOC_Os12g07190 | CBS domain-containing protein, putative, expressed |
| Os.21254.1.S1_at | 3.49 | 0.0132 | LOC_Os03g45400 | antitermination NusB domain-containing protein, putative, |
| Os.21318.1.S1_at | 4.80 | 0.0138 | LOC_Os06g04510 | Enolase 1, putative, expressed |
| Os.21364.2.S1_x_at | 2.62 | 0.0171 | LOC_Os07g43330 | DNAJ heat shock N-terminal domain-containing protein |
| Os.21367.1.S1_at | 3.39 | 0.0279 | LOC_Os10g34170 | Domain found in Dishevelled, Egl-10, and Pleckstrin family |
| Os.21398.1.S1_at | 3.66 | 0.0384 | LOC_Os03g52730 | magnesium-dependent phosphatase-1 family protein |
| Os.21406.1.A1_at | 3.39 | 0.0451 | LOC_Os02g35630 | ATP-dependent Clp protease ATP-binding subunit ClpX |
| Os.21457.1.S1_at | 1.75 | 0.0450 | LOC_Os10g35550 | U5 snRNP-associated 102 kDa protein, putative, expressed |
| Os.21578.1.S1_at | 1.72 | 0.0258 | LOC_Os02g44370 | GRAS family transcription factor containing protein |
| Os.21627.1.S1_at | 1.90 | 0.0281 | LOC_Os05g24010 | Protein kinase domain containing protein, expressed |
| Os.21784.1.S1_at | 4.31 | 0.0023 | LOC_Os08g36140 | expressed protein |
| Os.21835.1.S1_at | 1.88 | 0.0278 | LOC_Os08g37310 | Uncharacterized protein family UPF0029 containing protein, |
| Os.21849.1.S1_at | 2.54 | 0.0140 | LOC_Os03g11120 | Methionyl-tRNA synthetase, putative, expressed |
| Os.21880.2.S1_x_at | 3.99 | 0.0023 | LOC_Os04g49940 | ferredoxin, putative, expressed |
| Os.22029.1.S1_a_at | 3.65 | 0.0004 | LOC_Os10g41780 | chlorophyll a oxygenase, putative, expressed |
| Os.22263.1.S1_a_at | 2.34 | 0.0106 | LOC_Os07g40550 | protein kinase family protein, putative, expressed |
| Os.22374.1.S2_a_at | 2.82 | 0.0103 | LOC_Os04g58710 | AMP-binding enzyme family protein, expressed |
| Os.2242.1.S1_at | 1.97 | 0.0098 | Os04g0691600 | 30S ribosomal protein S17, chloroplast precursor (CS17). |
| Os.2243.2.S1_at | 2.22 | 0.0095 | LOC_Os03g03360 | 50S ribosomal protein L5, chloroplast precursor, putative, |
| Os.2244.1.S1_at | 2.94 | 0.0022 | Os03g0284400 | Ribosomal protein L10-like. |
| Os.22452.1.S1_at | 4.16 | 0.0025 | LOC_Os12g30030 | HesB/YadR/YfhF family protein, putative, expressed |
| Os.22453.1.S1_at | 1.79 | 0.0092 | LOC_Os06g50050 | ATPase, AAA family protein, expressed |
| Os.22472.1.S1_x_at | 3.32 | 0.0121 | LOC_Os10g22430 | Chitin-inducible gibberellin-responsive protein 2 |
| Os.22474.1.S1_at | 8.34 | 0.0000 | Os06g0725900 | Cell division protein ftsH homolog, chloroplast precursor |
| Os.2248.1.S1_at | 2.35 | 0.0220 | LOC_Os07g37030 | Cytochrome b6-f complex iron-sulfur subunit |
| Os.22483.1.A1_at | 5.13 | 0.0011 | LOC_Os03g62510 | FAD dependent oxidoreductase family protein, expressed |
| Os.22579.1.S1_at | 3.23 | 0.0014 | LOC_Os12g17320 | Prenyl transferase, putative, expressed |
| Os.22619.1.S2_at | 1.62 | 0.0078 | LOC_Os04g35200 | hypothetical protein |
| Os.22623.1.S1_at | 2.07 | 0.0257 | LOC_Os05g04070 | expressed protein |
| Os.22623.1.S1_x_at | 1.99 | 0.0075 | LOC_Os05g04090 | hypothetical protein |
| Os.22638.1.S1_x_at | 2.72 | 0.0005 | Os03g0295800 | Gamma interferon inducible lysosomal thiol reductase GILT family protein. |
| Os.22651.1.S1_at | 17.57 | 0.0420 | LOC_Os04g56230 | Farnesyl pyrophosphate synthetase 2, putative, expressed |
| Os.22668.1.A1_at | 1.85 | 0.0117 | LOC_Os03g14120 | dihydrodipicolinate reductase family protein |
| Os.22699.1.S1_at | 5.35 | 0.0259 | LOC_Os09g28650 | esterase, putative, expressed |
| Os.22726.1.A1_at | 6.62 | 0.0028 | LOC_Os10g37110 | transposon protein, putative, unclassified, expressed |
| Os.22749.1.S1_at | 1.65 | 0.0044 | LOC_Os02g18850 | prolyl oligopeptidase family protein, expressed |
| Os.22767.1.S1_at | 5.85 | 0.0384 | Os09g0416200 | H(+)/hexose cotransporter. |
| Os.22795.1.S1_at | 4.05 | 0.0269 | LOC_Os02g33500 | Threonyl-tRNA synthetase, putative, expressed |
| Os.22812.1.S1_a_at | 1.52 | 0.0328 | LOC_Os01g57720 | expressed protein |
| Os.22842.1.S1_at | 9.00 | 0.0398 | LOC_Os12g25170 | NB-ARC domain containing protein, expressed |
| Os.23028.1.S1_s_at | 2.44 | 0.0402 | LOC_Os03g08490 | AP2 domain containing protein, expressed |
| Os.23056.2.S1_x_at | 11.82 | 0.0054 | LOC_Os01g68450 | expressed protein |
| Os.23131.1.S1_at | 1.95 | 0.0081 | LOC_Os08g01590 | expressed protein |
| Os.23135.1.A1_at | 5.94 | 0.0422 | LOC_Os01g21990 | CRS2-associated factor 2, putative, expressed |
| Os.23145.1.S1_at | 11.95 | 0.0283 | LOC_Os08g03310 | Zinc finger C-x8-C-x5-C-x3-H type family protein, expresse |
| Os.23178.1.S1_at | 2.02 | 0.0086 | LOC_Os01g32350 | ATP-dependent Clp protease proteolytic subunit 1, putative |
| Os.23200.1.S1_x_at | 1.81 | 0.0335 | LOC_Os07g27760 | expressed protein |
| Os.23200.2.S1_x_at | 1.80 | 0.0214 | LOC_Os07g27760 | expressed protein |
| Os.23290.2.S1_x_at | 7.21 | 0.0391 | LOC_Os10g40960 | oxidoreductase, 2OG-Fe oxygenase family protein, expressed |
| Os.23296.1.S1_x_at | 17.99 | 0.0032 | LOC_Os02g30110 | Cytochrome P450 family protein, expressed |
| Os.2330.1.S1_at | 2.79 | 0.0014 | LOC_Os03g08570 | Phytoene dehydrogenase, chloroplast precursor |
| Os.23303.1.A1_at | 3.96 | 0.0412 | Os03g0377700 | Cellulose synthase-like A5. |
| Os.23313.1.S1_x_at | 3.14 | 0.0245 | LOC_Os03g51950 | expressed protein |
| Os.23349.1.S1_x_at | 2.22 | 0.0168 | LOC_Os03g06190 | 3'-5' exonuclease family protein, expressed |
| Os.23416.1.S1_x_at | 3.14 | 0.0412 | LOC_Os07g39270 | Geranylgeranyl hydrogenase, putative, expressed |
| Os.23434.1.S1_s_at | 3.89 | 0.0283 | LOC_Os06g51460 | ABC transporter family protein, putative, expressed |
| Os.2353.1.S1_at | 2.22 | 0.0412 | Os03g0444800 | Hypothetical protein. |
| Os.23606.1.S1_at | 3.88 | 0.0283 | LOC_Os01g29330 | expressed protein |
| Os.23613.1.S1_at | 4.71 | 0.0275 | LOC_Os06g07020 | Zinc finger, C2H2 type family protein, expressed |
| Os.23659.1.S1_at | 1.67 | 0.0478 | LOC_Os03g61730 | DnaJ domain containing protein, expressed |
| Os.23664.1.S1_at | 2.16 | 0.0265 | LOC_Os01g12550 | expressed protein |
| Os.23673.1.S1_at | 3.61 | 0.0262 | LOC_Os01g55290 | pentatricopeptide, putative, expressed |
| Os.23677.1.S1_at | 1.59 | 0.0345 | LOC_Os01g56800 | MATH domain containing protein, expressed |
| Os.23687.1.A1_at | 6.18 | 0.0017 | LOC_Os02g32780 | pentatricopeptide, putative, expressed |
| Os.23745.1.S1_at | 10.27 | 0.0100 | LOC_Os04g13220 | glutathione-conjugate transporter, putative, expressed |
| Os.23773.1.A1_at | 6.11 | 0.0014 | LOC_Os09g27900 | D-xylose-proton symporter, putative, expressed |
| Os.23802.1.A1_at | 2.34 | 0.0259 | LOC_Os04g39540 | expressed protein |
| Os.23808.1.S1_x_at | 8.27 | 0.0040 | LOC_Os05g03750 | expressed protein |
| Os.23808.2.S1_at | 4.49 | 0.0181 | unknown | unknown |
| Os.23808.3.S1_x_at | 7.38 | 0.0071 | LOC_Os05g03750 | expressed protein |
| Os.23819.1.S1_s_at | 2.08 | 0.0243 | LOC_Os04g35170 | expressed protein |
| Os.23847.1.S1_at | 3.49 | 0.0208 | LOC_Os05g14270 | expressed protein |
| Os.23916.1.A1_at | 4.52 | 0.0036 | Os05g0294800 | Non-protein coding transcript, putative npRNA. |
| Os.23932.1.A1_at | 2.29 | 0.0439 | LOC_Os04g40140 | expressed protein |
| Os.23936.1.S1_at | 4.14 | 0.0071 | LOC_Os06g01670 | myb family transcription factor, putative, expressed |
| Os.24019.1.S1_at | 2.02 | 0.0100 | LOC_Os02g35720 | Microsomal signal peptidase 25 kDa subunit family protein, |
| Os.24026.1.S1_at | 2.97 | 0.0227 | Os10g0476900 | Protein prenyltransferase domain containing protein. |
| Os.24032.1.S1_at | 7.59 | 0.0021 | LOC_Os03g42464 | unspliced-genomic expressed protein |
| Os.24034.2.S1_x_at | 2.94 | 0.0190 | LOC_Os01g65400 | 5'-3' exonuclease, N-terminal resolvase-like domain containing protein |
| Os.24044.1.S1_at | 2.22 | 0.0150 | LOC_Os03g50940 | Cytochrome c oxidase assembly protein ctaG |
| Os.24093.2.S1_x_at | 2.11 | 0.0235 | unknown | unknown |
| Os.2422.1.S1_a_at | 6.77 | 0.0162 | LOC_Os07g07540 | SHOOT1 protein, putative, expressed |
| Os.24327.1.A1_at | 3.67 | 0.0392 | LOC_Os02g52410 | unspliced-genomic expressed protein |
| Os.24334.1.A1_at | 3.63 | 0.0093 | LOC_Os06g20410 | BAH domain containing protein, expressed |
| Os.24421.1.S1_at | 1.92 | 0.0486 | LOC_Os10g26400 | oxidoreductase, putative, expressed |
| Os.24424.1.S1_at | 2.07 | 0.0388 | LOC_Os08g41270 | Type II inositol-1,4,5-trisphosphate 5-phosphatase 12 |
| Os.24428.1.S1_at | 2.85 | 0.0031 | LOC_Os07g48970 | expressed protein |
| Os.24556.1.S1_at | 1.98 | 0.0022 | LOC_Os08g33640 | expressed protein |
| Os.24561.1.S1_s_at | 1.95 | 0.0254 | LOC_Os06g43790 | Bromodomain containing protein, expressed |
| Os.24631.1.A1_at | 2.28 | 0.0174 | Os02g0267000 | Chloroplast thylakoidal processing peptidase (Putative signal peptidase I). |
| Os.24631.1.A1_s_at | 1.94 | 0.0182 | Os02g0267000 | Chloroplast thylakoidal processing peptidase (Putative signal peptidase I). |
| Os.24642.1.S1_at | 1.66 | 0.0284 | LOC_Os04g41530 | serine/threonine-protein kinase GCN2, putative |
| Os.24660.1.S1_at | 3.46 | 0.0002 | LOC_Os03g63560 | pentatricopeptide, putative, expressed |
| Os.24660.2.S1_at | 1.71 | 0.0134 | Os03g0852600 | Protein of unknown function DUF565 family protein. |
| Os.24663.1.S1_s_at | 2.10 | 0.0054 | LOC_Os02g51480 | pentatricopeptide, putative, expressed |
| Os.2467.1.S1_at | 3.99 | 0.0104 | LOC_Os04g31040 | violaxanthin de-epoxidase, putative, expressed |
| Os.24683.1.A1_s_at | 3.40 | 0.0051 | LOC_Os09g14520 | integral membrane family protein, putative, expressed |
| Os.24684.1.S1_at | 3.28 | 0.0218 | LOC_Os03g20640 | expressed protein |
| Os.24711.1.A1_at | 3.89 | 0.0065 | LOC_Os11g38020 | small GTP-binding protein domain containing protein |
| Os.2476.1.S1_at | 2.07 | 0.0069 | LOC_Os05g01110 | 50S ribosomal protein L28, chloroplast precursor, putative |
| Os.24760.1.A1_at | 1.93 | 0.0162 | LOC_Os02g51030 | methyltransferase, UbiE/COQ5 family protein, expressed |
| Os.24834.1.S1_at | 5.12 | 0.0353 | Os09g0548400 | Conserved hypothetical protein. |
| Os.24860.1.S1_at | 4.35 | 0.0344 | LOC_Os03g42020 | Calcium-transporting ATPase 2, plasma membrane-type |
| Os.24869.1.A1_at | 2.33 | 0.0223 | LOC_Os01g06020 | Aspartyl-tRNA synthetase, putative, expressed |
| Os.24901.1.A1_at | 8.18 | 0.0229 | LOC_Os01g39960 | Lycopene epsilon cyclase, chloroplast precursor, putative, |
| Os.24906.1.S1_at | 10.41 | 0.0166 | LOC_Os01g72460 | NADPH:quinone oxidoreductase, putative, expressed |
| Os.24927.1.S1_at | 4.00 | 0.0212 | LOC_Os03g56160 | lectin receptor kinase 7, putative, expressed |
| Os.25060.1.S1_at | 1.65 | 0.0243 | LOC_Os01g65890 | DNA repair metallo-beta-lactamase family protein, expressed |
| Os.25062.1.A1_a_at | 4.43 | 0.0084 | LOC_Os01g48300 | expressed protein |
| Os.25065.3.S1_at | 2.19 | 0.0456 | Os01g0819000 | Conserved hypothetical protein. |
| Os.25169.1.S1_at | 2.18 | 0.0054 | LOC_Os08g01090 | B3 DNA binding domain containing protein, expressed |
| Os.2517.1.S1_a_at | 1.57 | 0.0410 | LOC_Os10g37630 | UBX domain containing protein, expressed |
| Os.25215.1.A1_at | 1.62 | 0.0383 | LOC_Os03g04270 | Leucine Rich Repeat family protein, expressed |
| Os.25239.1.S1_a_at | 2.92 | 0.0038 | LOC_Os01g72800 | signal recognition particle-docking protein FtsY containing |
| Os.25250.1.S1_at | 3.03 | 0.0112 | Os02g0636300 | DEAD/DEAH box helicase, N-terminal domain containing protein. |
| Os.25261.1.S1_at | 4.79 | 0.0009 | LOC_Os07g10540 | aminopeptidase P, cytosolic, putative, expressed |
| Os.25299.3.A1_at | 2.22 | 0.0092 | LOC_Os06g07010 | transposon protein, putative, unclassified, expressed |
| Os.25324.1.A1_at | 2.61 | 0.0360 | LOC_Os08g12780 | expressed protein |
| Os.25378.1.S1_at | 1.55 | 0.0056 | LOC_Os08g06510 | Zinc finger, C3HC4 type family protein, expressed |
| Os.25486.1.S1_at | 1.71 | 0.0253 | LOC_Os01g56230 | expressed protein |
| Os.25496.1.S1_at | 28.10 | 0.0206 | LOC_Os11g07930 | oxidoreductase, short chain dehydrogenase/reductase family |
| Os.25510.1.S1_at | 5.18 | 0.0364 | LOC_Os08g05650 | Diacylglycerol kinase catalytic domain family protein |
| Os.25529.1.S1_at | 5.92 | 0.0002 | LOC_Os05g44330 | DJ-1 family protein, expressed |
| Os.25530.1.S2_at | 5.86 | 0.0073 | LOC_Os04g43430 | pentatricopeptide, putative, expressed |
| Os.25561.1.S1_at | 5.22 | 0.0005 | LOC_Os05g22730 | one helix protein, putative, expressed |
| Os.25587.1.S1_at | 9.76 | 0.0171 | LOC_Os04g57320 | Immutans protein, putative, expressed |
| Os.25587.3.S1_x_at | 5.10 | 0.0034 | LOC_Os03g63010 | Immutans protein, putative, expressed |
| Os.25613.1.A1_s_at | 7.35 | 0.0499 | LOC_Os02g05470 | CCT motif family protein, expressed |
| Os.25806.1.S1_at | 7.48 | 0.0007 | Os02g0794600 | Cytochrome c oxidase copper chaperone (Dopuin). |
| Os.25806.1.S1_s_at | 1.83 | 0.0103 | Os02g0794600 | Cytochrome c oxidase copper chaperone (Dopuin). |
| Os.25955.1.S1_at | 4.01 | 0.0147 | LOC_Os11g34940 | expressed protein |
| Os.2601.1.S1_at | 1.59 | 0.0210 | LOC_Os10g35930 | LIM domain containing protein, expressed |
| Os.26040.1.S1_at | 3.54 | 0.0035 | LOC_Os03g62630 | ribosomal protein S6 family protein, putative, expressed |
| Os.26206.1.S1_at | 5.31 | 0.0042 | LOC_Os03g56280 | Malate dehydrogenase, glyoxysomal precursor, putative |
| Os.26271.1.A1_s_at | 3.61 | 0.0044 | LOC_Os01g67490 | OTU-like cysteine protease family protein, expressed |
| Os.26359.3.S1_at | 2.68 | 0.0015 | Os03g0344100 | Splicing factor SR1D. |
| Os.26362.1.S1_at | 2.10 | 0.0412 | LOC_Os01g56140 | expressed protein |
| Os.26386.1.S1_a_at | 2.88 | 0.0281 | LOC_Os04g57020 | expressed protein |
| Os.26397.1.S1_at | 3.31 | 0.0025 | Os06g0601000 | Homeodomain-like containing protein. |
| Os.26408.3.S1_x_at | 3.05 | 0.0359 | LOC_Os02g57660 | Phosphatidylinositol-4-phosphate 5-kinase 9, putative |
| Os.26476.1.S1_at | 1.53 | 0.0482 | LOC_Os03g02440 | expressed protein |
| Os.26508.2.S1_a_at | 1.62 | 0.0071 | LOC_Os07g35870 | bHLH transcription factor, putative, expressed |
| Os.26509.1.S1_x_at | 2.30 | 0.0420 | LOC_Os02g24600 | NADH ubiquinone oxidoreductase, 20 Kd subunit family protein |
| Os.26510.1.S1_at | 2.12 | 0.0223 | Os11g0588600 | Disease resistance protein family protein. |
| Os.26522.1.S1_at | 2.30 | 0.0441 | LOC_Os08g15296 | unspliced-genomic photosystem II reaction center protein H, putative, expressed |
| Os.26537.2.S1_x_at | 3.71 | 0.0303 | LOC_Os01g70520 | Glycosyl hydrolase family 1 protein, expressed |
| Os.26548.1.S1_at | 2.45 | 0.0449 | LOC_Os04g38940 | Integral membrane protein, expressed |
| Os.26549.1.S1_at | 4.20 | 0.0187 | LOC_Os02g54980 | Rieske domain containing protein, expressed |
| Os.26552.1.S1_at | 9.91 | 0.0313 | LOC_Os10g39430 | expressed protein |
| Os.2657.1.S1_x_at | 1.99 | 0.0489 | LOC_Os10g39540 | Protein phosphatase 2C containing protein, expressed |
| Os.26570.1.S1_s_at | 4.48 | 0.0277 | LOC_Os01g64270 | expressed protein |
| Os.26579.1.S1_at | 1.60 | 0.0327 | LOC_Os04g45140 | expressed protein |
| Os.26587.1.A1_at | 4.11 | 0.0157 | LOC_Os04g11400 | expressed protein |
| Os.26588.1.S1_x_at | 2.74 | 0.0032 | LOC_Os03g11300 | expressed protein |
| Os.26592.1.S1_s_at | 3.52 | 0.0348 | LOC_Os09g04680 | Photosystem II P680 chlorophyll A apoprotein, putative |
| Os.26592.2.A1_s_at | 4.15 | 0.0175 | LOC_Os10g21310 | Photosystem II P680 chlorophyll A apoprotein, putative |
| Os.26592.2.A1_x_at | 4.45 | 0.0176 | LOC_Os12g10600 | Photosystem II P680 chlorophyll A apoprotein |
| Os.26592.3.S1_x_at | 3.71 | 0.0272 | LOC_Os12g10600 | Photosystem II P680 chlorophyll A apoprotein, putative |
| Os.26642.1.S1_a_at | 3.38 | 0.0001 | LOC_Os01g51410 | Glycine dehydrogenase, mitochondrial precursor, putative, |
| Os.26654.1.S1_at | 3.65 | 0.0468 | LOC_Os10g01060 | U-box domain containing protein, expressed |
| Os.26686.1.S1_a_at | 3.51 | 0.0017 | LOC_Os02g43350 | ATP-dependent metalloprotease FtsH family protein |
| Os.26696.1.S1_at | 1.58 | 0.0325 | LOC_Os12g21710 | expressed protein |
| Os.26702.1.A1_s_at | 1.53 | 0.0133 | LOC_Os06g13050 | Peroxidase family protein, expressed |
| Os.26732.1.S1_at | 6.94 | 0.0027 | Os01g0175700 | HCO3-transporter domain containing protein. |
| Os.26733.3.S1_x_at | 2.49 | 0.0112 | LOC_Os01g45040 | hypothetical protein |
| Os.26751.2.S1_at | 2.20 | 0.0251 | LOC_Os04g16750 | Photosystem I P700 chlorophyll a apoprotein A2, putative, |
| Os.26758.1.A1_a_at | 2.78 | 0.0442 | LOC_Os03g04300 | Ankyrin repeat protein, chloroplast precursor, putative, e |
| Os.26761.1.S1_s_at | 3.29 | 0.0138 | Os01g0881600 | Photosystem II reaction center J protein. |
| Os.26770.2.S1_x_at | 3.13 | 0.0241 | LOC_Os04g16740 | ATP synthase alpha chain, putative, expressed |
| Os.26783.1.S1_at | 2.81 | 0.0050 | LOC_Os10g29530 | expressed protein |
| Os.26783.2.S1_x_at | 2.13 | 0.0046 | LOC_Os10g29530 | expressed protein |
| Os.26786.1.S1_at | 4.39 | 0.0013 | LOC_Os09g15330 | Sugar carrier protein A, putative, expressed |
| Os.26798.1.S1_at | 9.23 | 0.0068 | LOC_Os12g35630 | Elongation factor TS family protein, expressed |
| Os.26807.1.S1_at | 10.32 | 0.0001 | LOC_Os01g12210 | Uncharacterized protein family UPF0005 containing protein, |
| Os.26818.1.S1_a_at | 8.21 | 0.0008 | LOC_Os03g19650 | PPR2, putative, expressed |
| Os.26825.1.S1_at | 3.06 | 0.0092 | Os01g0218700 | Sugar transporter superfamily protein. |
| Os.26832.1.S1_at | 3.60 | 0.0010 | LOC_Os07g12530 | ABC1 family protein, expressed |
| Os.26862.1.S1_s_at | 6.13 | 0.0031 | LOC_Os11g04490 | saccharopine dehydrogenase family protein, expressed |
| Os.26876.1.S1_at | 4.31 | 0.0226 | LOC_Os01g57410 | Rf1 protein, mitochondrial precursor, putative, expressed |
| Os.26965.1.S1_at | 6.28 | 0.0404 | LOC_Os01g61120 | expressed protein |
| Os.26967.1.S2_at | 2.76 | 0.0261 | Os01g0701900 | Phosphatidylinositol transfer-like protein III. |
| Os.26974.1.S1_at | 3.29 | 0.0118 | unknown | unknown |
| Os.2698.3.S1_x_at | 1.76 | 0.0441 | LOC_Os01g59660 | Transcription factor GAMYB, putative, expressed |
| Os.26990.1.S1_at | 2.64 | 0.0408 | LOC_Os01g57350 | Diacylglycerol kinase catalytic domain family protein |
| Os.26991.1.S1_x_at | 5.64 | 0.0237 | LOC_Os01g43120 | DEAD/DEAH box helicase family protein |
| Os.26998.1.S2_at | 1.84 | 0.0489 | LOC_Os02g05660 | ATP-dependent helicase DDX41 |
| Os.27003.1.S2_a_at | 6.84 | 0.0102 | LOC_Os10g28600 | pentatricopeptide, putative, expressed |
| Os.27006.1.S1_at | 6.27 | 0.0430 | LOC_Os03g16430 | Sigma-70 region 3 family protein, expressed |
| Os.27024.1.S1_at | 3.11 | 0.0013 | LOC_Os02g17360 | pentatricopeptide, putative, expressed |
| Os.27038.1.S1_at | 1.94 | 0.0086 | LOC_Os04g35700 | mitogen-activated kinase kinase kinase alpha, putative |
| Os.27079.1.A1_at | 3.86 | 0.0006 | LOC_Os03g16910 | SLT1 protein, putative, expressed |
| Os.27085.1.A1_at | 3.77 | 0.0476 | LOC_Os08g33940 | Myb-like DNA-binding domain containing protein, expressed |
| Os.27127.1.S1_a_at | 3.82 | 0.0143 | LOC_Os05g23320 | expressed protein |
| Os.27146.1.A1_at | 3.66 | 0.0313 | LOC_Os03g21630 | expressed protein |
| Os.27164.1.A1_a_at | 1.79 | 0.0109 | LOC_Os07g30200 | Asparaginyl-tRNA synthetase, putative, expressed |
| Os.27175.1.S1_a_at | 2.96 | 0.0073 | LOC_Os07g12890 | ZIP zinc/iron transport family protein, expressed |
| Os.27196.1.S1_x_at | 2.14 | 0.0015 | LOC_Os04g43220 | Zinc finger, C3HC4 type family protein, expressed |
| Os.27212.1.A1_at | 1.98 | 0.0236 | unknown | unknown |
| Os.27216.1.A1_at | 6.66 | 0.0001 | LOC_Os05g34600 | No apical meristem protein, expressed |
| Os.27221.1.A1_at | 3.68 | 0.0022 | LOC_Os03g63910 | pentatricopeptide, putative, expressed |
| Os.27235.2.S1_x_at | 1.86 | 0.0232 | LOC_Os01g35170 | tRNA methyltransferase family protein, expressed |
| Os.27275.1.S1_at | 2.31 | 0.0387 | LOC_Os08g40570 | Pyridoxamine 5'-phosphate oxidase family protein |
| Os.27278.1.S1_s_at | 1.54 | 0.0367 | LOC_Os01g01720 | Peroxisomal membrane anchor protein conserved region |
| Os.27314.1.S1_at | 2.66 | 0.0067 | LOC_Os02g24580 | NAD, putative |
| Os.27322.1.S1_x_at | 3.89 | 0.0258 | LOC_Os01g52640 | WD-40 repeat family protein, putative, expressed |
| Os.27322.2.S1_at | 3.60 | 0.0439 | LOC_Os01g52640 | WD-40 repeat family protein, putative, expressed |
| Os.27322.3.S1_x_at | 3.19 | 0.0465 | LOC_Os01g52640 | WD-40 repeat family protein, putative, expressed |
| Os.27334.1.S1_at | 9.13 | 0.0193 | LOC_Os02g17350 | VHS domain containing protein, expressed |
| Os.27346.1.S1_at | 15.55 | 0.0012 | LOC_Os03g49630 | expressed protein |
| Os.27353.1.S1_at | 4.27 | 0.0055 | LOC_Os04g53460 | AT hook motif family protein, expressed |
| Os.27354.1.S1_at | 2.25 | 0.0195 | LOC_Os09g17600 | expressed protein |
| Os.27357.1.A1_at | 3.99 | 0.0049 | LOC_Os11g13420 | RNase3 domain containing protein, expressed |
| Os.27375.1.A1_at | 1.66 | 0.0169 | LOC_Os02g04460 | 50S ribosomal protein L3, chloroplast precursor, putative, |
| Os.27377.1.S1_at | 2.18 | 0.0339 | LOC_Os06g11040 | expressed protein |
| Os.27416.1.S1_at | 6.82 | 0.0412 | LOC_Os05g31680 | expressed protein |
| Os.27417.1.S1_at | 5.21 | 0.0064 | LOC_Os01g52170 | Mrp protein homolog, putative, expressed |
| Os.27425.1.S1_at | 3.72 | 0.0013 | LOC_Os01g21820 | Preprotein translocase secA subunit, chloroplast precursor |
| Os.27426.1.S1_at | 2.67 | 0.0000 | LOC_Os05g44080 | MAP kinase-activating protein C22orf5, putative, expressed |
| Os.27433.1.S1_at | 2.57 | 0.0073 | LOC_Os07g30120 | hypothetical protein |
| Os.27438.2.S1_x_at | 4.19 | 0.0060 | LOC_Os07g19550 | expressed protein |
| Os.27449.1.S1_at | 4.33 | 0.0010 | Os02g0725200 | Nuclear protein SET domain containing protein. |
| Os.27452.1.S1_at | 2.00 | 0.0459 | LOC_Os08g02860 | transposon protein, putative, unclassified, expressed |
| Os.27454.1.S1_x_at | 4.88 | 0.0442 | Os07g0102100 | K+ potassium transporter family protein. |
| Os.27454.2.S1_at | 3.34 | 0.0315 | Os07g0102100 | K+ potassium transporter family protein. |
| Os.27469.2.S1_at | 2.19 | 0.0278 | LOC_Os01g09550 | No apical meristem protein, expressed |
| Os.27477.1.S1_at | 4.84 | 0.0270 | LOC_Os07g38830 | hydrolase, alpha/beta fold family protein, putative |
| Os.27502.1.S1_at | 2.65 | 0.0296 | Os04g0436400 | Hypothetical protein. |
| Os.27508.1.S1_a_at | 1.57 | 0.0104 | LOC_Os03g25970 | KH domain-containing protein, putative, expressed |
| Os.27510.1.A1_at | 6.15 | 0.0050 | LOC_Os06g05390 | expressed protein |
| Os.27513.1.A1_a_at | 11.48 | 0.0007 | Os01g0208700 | Phosphoenolpyruvate-carboxylase (Fragment). |
| Os.27516.1.A1_at | 2.33 | 0.0095 | LOC_Os09g24320 | 2-oxo acid dehydrogenases acyltransferase family protein, |
| Os.27519.1.S1_at | 2.68 | 0.0101 | LOC_Os04g51310 | putrescine-binding periplasmic protein, putative, expressed |
| Os.27520.1.S1_at | 3.74 | 0.0148 | LOC_Os12g02320 | Nonspecific lipid-transfer protein 1 precursor, putative, |
| Os.27520.2.S1_x_at | 2.14 | 0.0429 | Os11g0115400 | Nonspecific lipid-transfer protein precursor |
| Os.27530.1.S1_at | 1.64 | 0.0084 | LOC_Os11g46300 | expressed protein |
| Os.27577.1.S1_at | 5.62 | 0.0406 | LOC_Os01g04900 | peptidase M50 family protein, putative, expressed |
| Os.27587.1.S1_at | 5.22 | 0.0058 | LOC_Os10g40740 | Helix-loop-helix DNA-binding domain containing protein |
| Os.27592.1.A1_at | 70.78 | 0.0400 | LOC_Os11g42490 | retrotransposon protein, putative, unclassified, expressed |
| Os.27603.1.S1_at | 6.71 | 0.0072 | Os01g0356000 | Os01g0356000 ABC transporter related domain containing protein |
| Os.27610.1.S1_at | 8.42 | 0.0018 | LOC_Os12g42300 | Potassium transporter, putative, expressed |
| Os.27626.1.S1_at | 4.40 | 0.0349 | LOC_Os06g05950 | expressed protein |
| Os.27627.1.S1_at | 8.02 | 0.0459 | LOC_Os01g28540 | expressed protein |
| Os.27630.1.A1_at | 1.93 | 0.0422 | LOC_Os07g38300 | Ribosome recycling factor, chloroplast precursor, putative |
| Os.27632.1.S1_at | 4.56 | 0.0002 | Os01g0681000 | Protein of unknown function DUF1298 domain containing protein. |
| Os.27634.1.S1_at | 8.09 | 0.0046 | LOC_Os08g14450 | Sigma-70 region 2 family protein, expressed |
| Os.27635.1.S1_at | 3.48 | 0.0263 | LOC_Os01g51710 | zinc finger protein, putative, expressed |
| Os.27642.1.S1_at | 4.72 | 0.0066 | LOC_Os02g30320 | expressed protein |
| Os.27646.1.A1_at | 2.56 | 0.0272 | LOC_Os10g30610 | ABC transporter family protein, putative, expressed |
| Os.27650.1.S1_at | 5.69 | 0.0044 | LOC_Os07g43560 | Protein kinase domain containing protein, expressed |
| Os.27652.1.S1_at | 3.47 | 0.0188 | LOC_Os02g39740 | Tab2 protein, putative, expressed |
| Os.27654.1.S1_at | 3.03 | 0.0116 | LOC_Os05g49590 | phytochrome A supressor spa1, putative, expressed |
| Os.27656.1.S1_at | 2.12 | 0.0171 | LOC_Os05g04950 | expressed protein |
| Os.27663.1.A1_at | 3.75 | 0.0017 | LOC_Os09g28100 | Rhomboid family protein, expressed |
| Os.27664.1.S1_at | 1.72 | 0.0497 | LOC_Os02g40860 | Casein kinase I, epsilon isoform, putative, expressed |
| Os.27670.1.S1_at | 2.81 | 0.0072 | LOC_Os01g66170 | expressed protein |
| Os.27679.1.A1_at | 4.05 | 0.0012 | LOC_Os02g42960 | expressed protein |
| Os.27686.1.S1_at | 5.34 | 0.0028 | LOC_Os02g42570 | Ferredoxin-thioredoxin reductase, variable chain, putative |
| Os.27708.1.S1_at | 1.71 | 0.0306 | LOC_Os04g01520 | expressed protein |
| Os.27709.1.S1_a_at | 32.77 | 0.0185 | LOC_Os01g13570 | phosphoglycerate mutase family protein, expressed |
| Os.27717.1.S1_a_at | 2.69 | 0.0484 | LOC_Os01g58470 | expressed protein |
| Os.27724.2.A1_a_at | 2.11 | 0.0089 | LOC_Os02g43340 | Embryogenesis-associated protein EMB8, putative, expressed |
| Os.27729.1.S1_x_at | 1.90 | 0.0373 | LOC_Os04g16720 | ribosomal protein S15 containing protein |
| Os.27730.1.S1_at | 9.88 | 0.0045 | LOC_Os08g37700 | RNA recognition motif family protein, expressed |
| Os.27742.2.S1_a_at | 3.83 | 0.0058 | LOC_Os06g09090 | Ligand-gated ion channel family protein, expressed |
| Os.27745.1.A1_at | 1.72 | 0.0487 | LOC_Os06g12600 | Fructokinase, putative, expressed |
| Os.27754.1.S1_at | 6.94 | 0.0001 | LOC_Os02g03720 | expressed protein |
| Os.27758.1.S1_at | 3.26 | 0.0076 | Os07g0100300 | Glycosyl transferase, group 1 domain containing protein. |
| Os.27759.1.S1_at | 3.37 | 0.0081 | LOC_Os01g46720 | Protein kinase domain containing protein, expressed |
| Os.27768.1.S1_at | 1.51 | 0.0178 | LOC_Os08g04620 | Membrane associated protein, putative, expressed |
| Os.27770.1.A1_a_at | 6.74 | 0.0007 | LOC_Os03g48920 | expressed protein |
| Os.27770.3.A1_a_at | 7.52 | 0.0073 | LOC_Os03g48920 | expressed protein |
| Os.27773.1.S1_at | 3.72 | 0.0458 | LOC_Os04g52600 | Protein kinase domain containing protein, expressed |
| Os.27779.1.S1_at | 2.25 | 0.0408 | LOC_Os01g17010 | Phospholipid-transporting ATPase 1, putative, expressed |
| Os.27782.1.S1_at | 4.89 | 0.0033 | LOC_Os03g52640 | expressed protein |
| Os.27805.1.S1_at | 6.24 | 0.0014 | LOC_Os03g48000 | magnesium transporter CorA-like family protein |
| Os.27821.1.S1_s_at | 2.70 | 0.0195 | LOC_Os02g33580 | digalactosyldiacylglycerol synthase 1, putative, expressed |
| Os.27825.1.S1_at | 8.22 | 0.0001 | LOC_Os05g34630 | hydrolase, alpha/beta fold family protein, putative |
| Os.27826.1.S1_at | 2.29 | 0.0113 | LOC_Os03g12020 | 50S ribosomal protein L15, chloroplast precursor, putative |
| Os.27830.1.S1_at | 8.02 | 0.0029 | LOC_Os07g01480 | Oxygen evolving enhancer protein 3 containing protein |
| Os.27831.1.S1_at | 4.15 | 0.0114 | LOC_Os01g69120 | expressed protein |
| Os.27832.1.A1_s_at | 1.86 | 0.0278 | LOC_Os08g34750 | serine esterase family protein, expressed |
| Os.27835.1.S1_at | 11.32 | 0.0001 | LOC_Os06g13660 | Alanyl-tRNA synthetase, putative, expressed |
| Os.27837.1.S1_at | 4.27 | 0.0407 | LOC_Os06g37450 | GATA zinc finger family protein, expressed |
| Os.27837.2.S1_x_at | 7.07 | 0.0169 | LOC_Os06g37450 | GATA zinc finger family protein, expressed |
| Os.27861.1.S1_a_at | 3.00 | 0.0087 | Os09g0419100 | Hypothetical protein. |
| Os.27862.1.S1_at | 3.92 | 0.0009 | LOC_Os11g38260 | harpin binding protein 1, putative, expressed |
| Os.27881.1.S1_s_at | 1.63 | 0.0477 | LOC_Os07g39510 | expressed protein |
| Os.27893.1.S2_at | 1.72 | 0.0291 | LOC_Os01g67250 | N terminus of Rad21/Rec8 like protein, expressed |
| Os.27914.1.S1_at | 3.52 | 0.0079 | LOC_Os08g02390 | RNA recognition motif family protein, expressed |
| Os.27933.1.S1_at | 2.84 | 0.0008 | LOC_Os01g44980 | peptide deformylase, putative |
| Os.27942.1.A1_at | 2.78 | 0.0387 | LOC_Os01g49470 | CHY zinc finger family protein, expressed |
| Os.27944.1.S1_at | 4.08 | 0.0025 | LOC_Os03g08080 | YGGT family protein, expressed |
| Os.28045.1.S1_at | 3.55 | 0.0017 | LOC_Os02g44010 | expressed protein |
| Os.28089.1.S2_at | 3.52 | 0.0232 | LOC_Os10g28020 | prolyl oligopeptidase family protein, expressed |
| Os.28094.1.S1_at | 4.26 | 0.0008 | LOC_Os03g10060 | Chloroplast 30S ribosomal protein S10, putative, expressed |
| Os.28133.1.S1_s_at | 2.50 | 0.0033 | Os12g0291100 | Ribulose bisphosphate carboxylase, small chain family protein. |
| Os.28133.1.S1_x_at | 1.82 | 0.0038 | Os12g0291100 | Ribulose bisphosphate carboxylase, small chain family protein. |
| Os.28203.1.S1_a_at | 3.65 | 0.0493 | LOC_Os04g38600 | Glyceraldehyde-3-phosphate dehydrogenase A |
| Os.28216.2.S1_s_at | 1.62 | 0.0076 | LOC_Os07g37550 | Chlorophyll a-b binding protein of LHCII type III, chlorop |
| Os.28218.1.S1_x_at | 2.37 | 0.0284 | Os12g0291400 | Ribulose-1,5-bisphosphate carboxylase/oxygenase small subunit (Fragment). |
| Os.28255.1.S1_at | 2.68 | 0.0022 | LOC_Os04g54790 | ABC1 family protein, expressed |
| Os.28305.1.S1_at | 7.51 | 0.0331 | LOC_Os04g56110 | Protein kinase domain containing protein, expressed |
| Os.28394.1.S1_s_at | 2.81 | 0.0178 | LOC_Os06g41990 | metalloendopeptidase, putative, expressed |
| Os.28403.1.S1_a_at | 1.55 | 0.0063 | LOC_Os02g10390 | Chlorophyll a-b binding protein 8, chloroplast precursor, |
| Os.28404.1.A1_at | 16.51 | 0.0263 | LOC_Os03g07190 | expressed protein |
| Os.28424.1.A1_at | 75.83 | 0.0163 | unknown | unknown |
| Os.28438.4.S1_s_at | 2.47 | 0.0025 | LOC_Os01g31690 | Oxygen-evolving enhancer protein 1, chloroplast precursor, |
| Os.28459.1.S1_at | 3.26 | 0.0257 | LOC_Os11g26160 | Sigma-70 region 3 family protein, expressed |
| Os.2857.1.S1_at | 5.79 | 0.0001 | LOC_Os03g47970 | GATA transcription factor 27, putative, expressed |
| Os.286.1.S1_a_at | 5.91 | 0.0256 | LOC_Os01g17390 | F-box domain containing protein, expressed |
| Os.28814.1.S1_at | 4.92 | 0.0025 | LOC_Os01g49740 | expressed protein |
| Os.28814.2.S1_x_at | 1.51 | 0.0459 | LOC_Os01g49740 | expressed protein |
| Os.28976.1.S1_at | 4.84 | 0.0229 | LOC_Os01g10800 | pentatricopeptide, putative, expressed |
| Os.29051.1.S1_at | 5.39 | 0.0018 | LOC_Os03g17600 | UPF0308 protein, chloroplast precursor, putative, expresse |
| Os.2914.1.S1_at | 3.07 | 0.0263 | LOC_Os07g44330 | ATPase, histidine kinase, DNA gyrase B-, and HSP90-like domain protein |
| Os.29736.1.S1_at | 3.74 | 0.0172 | LOC_Os03g58540 | GTP1/OBG family protein, expressed |
| Os.29958.2.S1_x_at | 2.25 | 0.0159 | LOC_Os07g36400 | expressed protein |
| Os.30049.1.S1_at | 1.84 | 0.0206 | LOC_Os03g25960 | RNA recognition motif family protein, expressed |
| Os.30055.1.S1_x_at | 1.87 | 0.0397 | LOC_Os05g13420 | expressed protein |
| Os.30311.1.S1_at | 13.26 | 0.0143 | LOC_Os07g44860 | alpha/beta hydrolase fold family protein, putative |
| Os.30608.1.S1_at | 2.36 | 0.0047 | LOC_Os10g10180 | CMV 1a interacting protein 1, putative, expressed |
| Os.30724.1.S1_at | 2.49 | 0.0448 | LOC_Os02g51830 | histidyl-tRNA synthetase family protein, expressed |
| Os.30998.1.S1_at | 13.75 | 0.0087 | LOC_Os01g62060 | uncharacterized plant-specific domain TIGR01589 family protein |
| Os.3141.2.S1_at | 5.86 | 0.0032 | LOC_Os10g41260 | unspliced-genomic MYB family transcription factor |
| Os.31486.1.S1_at | 5.90 | 0.0274 | LOC_Os07g02910 | F-box domain containing protein, expressed |
| Os.3167.1.S1_at | 2.53 | 0.0017 | LOC_Os03g50070 | expressed protein |
| Os.31693.3.S1_at | 1.82 | 0.0217 | LOC_Os01g48830 | expressed protein |
| Os.31693.4.S1_x_at | 1.60 | 0.0492 | LOC_Os01g48830 | expressed protein |
| Os.31875.1.S1_at | 2.22 | 0.0021 | LOC_Os07g07060 | Anticodon binding domain containing protein, expressed |
| Os.32013.1.S1_a_at | 1.66 | 0.0205 | LOC_Os04g38900 | SPFH domain/Band 7 family protein, expressed |
| Os.32071.1.S1_at | 1.72 | 0.0046 | LOC_Os01g56470 | Mal d 1-associated protein, putative, expressed |
| Os.32072.1.S1_at | 3.38 | 0.0024 | LOC_Os08g33540 | ATP-dependent Clp protease adaptor protein ClpS containing |
| Os.32113.1.S1_at | 4.55 | 0.0012 | LOC_Os06g02580 | high-affinity nickel-transport family protein, putative |
| Os.32172.1.S1_at | 3.64 | 0.0455 | LOC_Os01g52290 | expressed protein |
| Os.32177.1.S1_at | 4.64 | 0.0061 | LOC_Os05g50930 | Sigma-70, region 4 family protein, expressed |
| Os.32279.1.S1_at | 8.74 | 0.0017 | LOC_Os01g63410 | expressed protein |
| Os.32447.1.S1_at | 6.23 | 0.0005 | LOC_Os01g59080 | expressed protein |
| Os.32449.1.S1_at | 3.39 | 0.0020 | LOC_Os02g51020 | expressed protein |
| Os.32481.1.S1_at | 1.68 | 0.0318 | LOC_Os01g01420 | expressed protein |
| Os.32500.2.A1_at | 1.65 | 0.0129 | LOC_Os06g47860 | expressed protein |
| Os.32503.1.S1_x_at | 2.00 | 0.0258 | LOC_Os07g08430 | Indole-3-glycerol phosphate lyase, chloroplast precursor, |
| Os.32506.1.S1_at | 3.78 | 0.0202 | LOC_Os06g47940 | nitrogen fixation protein, putative, expressed |
| Os.32625.1.S1_s_at | 1.60 | 0.0234 | LOC_Os01g56550 | Transcription initiation factor IIF, beta subunit family protein |
| Os.32721.1.S1_at | 24.45 | 0.0138 | LOC_Os01g53330 | UDP-glucoronosyl and UDP-glucosyl transferase family protein |
| Os.32953.1.S1_at | 1.86 | 0.0206 | LOC_Os04g38970 | expressed protein |
| Os.33110.1.S1_at | 1.83 | 0.0497 | LOC_Os01g45900 | F-box domain containing protein, expressed |
| Os.33271.2.S1_x_at | 2.55 | 0.0210 | LOC_Os08g06200 | Stomatin-like protein 2, putative, expressed |
| Os.33341.2.S1_x_at | 2.04 | 0.0477 | LOC_Os01g45830 | Sulfate transporter, putative, expressed |
| Os.33604.1.S1_a_at | 2.39 | 0.0455 | LOC_Os07g33480 | Cytochrome P450 family protein, expressed |
| Os.33607.1.S1_at | 2.22 | 0.0385 | LOC_Os01g49900 | peptidyl-tRNA hydrolase family protein, expressed |
| Os.33610.3.S1_s_at | 1.98 | 0.0181 | LOC_Os01g59340 | D-hydantoinase family protein, expressed |
| Os.33670.3.S1_s_at | 3.20 | 0.0016 | LOC_Os03g60790 | DnaJ domain containing protein, expressed |
| Os.33708.1.S1_s_at | 3.40 | 0.0115 | LOC_Os08g24390 | expressed protein |
| Os.3372.1.S1_at | 1.91 | 0.0168 | LOC_Os04g42930 | Glutaredoxin, putative, expressed |
| Os.3377.1.S1_at | 1.57 | 0.0152 | LOC_Os12g42090 | 37 kDa inner envelope membrane protein, chloroplast precur |
| Os.33770.1.S1_at | 3.93 | 0.0049 | LOC_Os01g59500 | U3 small nucleolar RNA-associated protein 11, putative, |
| Os.33788.1.S1_at | 3.87 | 0.0044 | LOC_Os01g73540 | YGGT family protein, putative, expressed |
| Os.3395.1.S1_at | 2.39 | 0.0429 | LOC_Os03g22770 | CCT motif family protein, expressed |
| Os.3397.1.S1_at | 4.50 | 0.0031 | LOC_Os02g39360 | B-box zinc finger family protein, expressed |
| Os.3399.1.S1_at | 9.49 | 0.0477 | Os09g0240200 | Zn-finger, CONSTANS type domain containing protein. |
| Os.3400.1.S1_s_at | 3.07 | 0.0348 | LOC_Os02g49230 | CCT motif family protein, expressed |
| Os.3407.1.S1_a_at | 3.37 | 0.0496 | LOC_Os08g06630 | Sigma-70 region 2 family protein, expressed |
| Os.3408.1.A2_a_at | 22.60 | 0.0133 | LOC_Os01g64660 | Fructose-1,6-bisphosphatase, cytosolic, putative, |
| Os.34151.1.S2_a_at | 3.16 | 0.0167 | LOC_Os02g46380 | purine permease family protein, putative, expressed |
| Os.34273.1.S1_at | 72.03 | 0.0165 | LOC_Os01g71710 | Amino acid permease family protein |
| Os.34341.1.S1_at | 1.59 | 0.0363 | LOC_Os01g24440 | expressed protein |
| Os.3445.1.S1_at | 1.67 | 0.0005 | Os11g0704500 | Metallothionein-like protein type 1. |
| Os.34459.1.S1_at | 3.52 | 0.0201 | LOC_Os10g28360 | 1,2-dihydroxy-3-keto-5-methylthiopentene dioxygenase |
| Os.34507.1.A1_at | 2.12 | 0.0051 | LOC_Os07g08770 | YGGT family protein, expressed |
| Os.3451.1.S1_at | 6.16 | 0.0025 | LOC_Os12g15470 | Serine carboxypeptidase I precursor, putative, expressed |
| Os.34512.1.S1_at | 1.51 | 0.0250 | LOC_Os01g53910 | NAD binding domain of 6-phosphogluconate dehydrogenase |
| Os.3454.1.S1_at | 27.28 | 0.0142 | LOC_Os01g69030 | Sucrose-phosphate synthase, putative, expressed |
| Os.34638.1.S1_at | 1.78 | 0.0140 | LOC_Os01g64930 | expressed protein |
| Os.34818.1.S1_x_at | 3.27 | 0.0116 | LOC_Os02g11000 | Agenet domain containing protein, expressed |
| Os.34902.1.S1_at | 1.73 | 0.0452 | Os10g0415400 | Hypothetical protein. |
| Os.35065.1.S1_at | 2.82 | 0.0049 | LOC_Os02g39040 | mitochondrial transcription termination factor, putative, |
| Os.35193.1.S1_at | 1.69 | 0.0240 | LOC_Os04g50870 | Elongation factor Tu C-terminal domain containing protein, |
| Os.35219.1.S1_a_at | 1.60 | 0.0153 | LOC_Os02g37030 | expressed protein |
| Os.35224.1.A1_x_at | 23.06 | 0.0000 | LOC_Os12g25180 | expressed protein |
| Os.353.1.S1_at | 1.82 | 0.0178 | LOC_Os04g08350 | Cysteine synthase, chloroplast precursor, putative, expres |
| Os.35302.1.S1_at | 7.71 | 0.0250 | LOC_Os02g45460 | Uncharacterized conserved protein, putative, expressed |
| Os.35409.1.A1_a_at | 3.61 | 0.0215 | Os12g0292900 | ATP-binding region, ATPase-like domain containing protein. |
| Os.35465.1.S1_at | 17.24 | 0.0231 | LOC_Os01g27120 | expressed protein |
| Os.35543.1.S1_at | 2.39 | 0.0157 | LOC_Os04g17700 | kinesin heavy chain isolog, putative, expressed |
| Os.35567.1.S1_at | 1.50 | 0.0074 | LOC_Os08g29110 | Thioredoxin family protein, expressed |
| Os.35573.1.S1_at | 4.48 | 0.0016 | LOC_Os01g41240 | hydrolase, alpha/beta fold family protein, expressed |
| Os.35613.1.S1_at | 3.04 | 0.0243 | Os05g0215600 | Conserved hypothetical protein. |
| Os.35677.1.S1_at | 3.19 | 0.0482 | LOC_Os03g09060 | 1,4-dihydroxy-2-naphthoate phytyltransferase family protein |
| Os.35688.1.S1_at | 7.21 | 0.0244 | LOC_Os11g44870 | expressed protein |
| Os.35754.1.S1_at | 1.73 | 0.0197 | LOC_Os09g19850 | CRS1/YhbY domain containing protein, expressed |
| Os.35778.1.S1_at | 9.25 | 0.0220 | LOC_Os01g56380 | Tyrosine/DOPA decarboxylase 2, putative, expressed |
| Os.35797.1.S1_at | 1.91 | 0.0274 | LOC_Os07g13890 | F-box domain containing protein, expressed |
| Os.35811.1.S1_at | 3.42 | 0.0094 | LOC_Os01g45370 | radical SAM enzyme, Cfr family protein, expressed |
| Os.35827.1.S1_at | 3.78 | 0.0306 | LOC_Os01g14510 | Protein kinase domain containing protein, expressed |
| Os.36369.1.S1_at | 4.21 | 0.0010 | LOC_Os06g27760 | Peptide methionine sulfoxide reductase msrB, putative |
| Os.36388.2.S1_x_at | 4.35 | 0.0005 | LOC_Os07g34950 | expressed protein |
| Os.36418.1.S1_at | 2.62 | 0.0379 | LOC_Os01g39840 | expressed protein |
| Os.36435.2.S1_x_at | 3.51 | 0.0158 | LOC_Os11g48080 | pyridoxin biosynthesis protein ER1, putative, expressed |
| Os.36529.1.S1_at | 2.73 | 0.0078 | LOC_Os01g68440 | expressed protein |
| Os.36648.1.S1_x_at | 13.52 | 0.0425 | LOC_Os01g13480 | Glutaredoxin family protein, expressed |
| Os.36706.1.S1_at | 1.98 | 0.0255 | LOC_Os07g33660 | expressed protein |
| Os.36729.1.A1_at | 1.58 | 0.0152 | LOC_Os09g26390 | ARID/BRIGHT DNA binding domain containing protein, |
| Os.36779.1.S1_s_at | 5.11 | 0.0008 | LOC_Os12g02330 | Nonspecific lipid-transfer protein precursor |
| Os.37047.1.S1_at | 3.44 | 0.0030 | Os04g0249700 | Hypothetical protein. |
| Os.37075.1.S1_at | 2.88 | 0.0007 | LOC_Os09g28280 | 2-Hydroxyisoflavanone dehydratase, putative, expressed |
| Os.3710.1.S1_at | 4.12 | 0.0183 | LOC_Os02g35440 | E3 ubiquitin ligase EL5, putative |
| Os.37204.1.S1_at | 5.29 | 0.0173 | LOC_Os12g08830 | thylakoid lumenal 29.8 kDa protein, putative, expressed |
| Os.37320.1.S1_at | 1.74 | 0.0224 | LOC_Os03g62950 | expressed protein |
| Os.37320.2.S1_at | 1.76 | 0.0346 | LOC_Os03g62950 | expressed protein |
| Os.37320.2.S1_x_at | 1.85 | 0.0152 | LOC_Os03g62950 | expressed protein |
| Os.37370.1.S1_at | 1.97 | 0.0012 | LOC_Os04g32560 | ATP-dependent Clp protease ATP-binding subunit clpA |
| Os.37384.1.S1_x_at | 2.25 | 0.0152 | LOC_Os05g06510 | 4Fe-4S binding domain containing protein, expressed |
| Os.3739.1.S1_at | 2.70 | 0.0136 | LOC_Os03g44230 | proline transporter, putative, expressed |
| Os.37415.1.S1_at | 3.24 | 0.0035 | LOC_Os07g42880 | unspliced-genomic PPR repeat containing protein,expressed |
| Os.37494.1.S1_x_at | 8.08 | 0.0293 | LOC_Os11g24450 | Mitochondrial carrier protein, expressed |
| Os.37570.1.S1_s_at | 1.69 | 0.0122 | LOC_Os09g02400 | expressed protein |
| Os.37673.1.S1_at | 1.52 | 0.0032 | unknown | unknown |
| Os.3774.1.S1_at | 2.35 | 0.0227 | LOC_Os01g08930 | DEAD/DEAH box helicase family protein, expressed |
| Os.37742.1.S1_at | 1.84 | 0.0004 | LOC_Os03g54790 | ABC transporter transmembrane region family protein |
| Os.37762.1.S1_a_at | 2.80 | 0.0120 | LOC_Os01g72980 | Glucose-inhibited division protein A, putative, expressed |
| Os.37894.1.S1_at | 3.87 | 0.0037 | LOC_Os03g52170 | 4-hydroxy-3-methylbut-2-enyl diphosphate reductase |
| Os.37955.1.S2_at | 2.06 | 0.0061 | Os03g0387900 | Hypothetical protein. |
| Os.37955.2.S1_at | 12.00 | 0.0458 | Os03g0387900 | Hypothetical protein. |
| Os.37961.1.S1_s_at | 2.00 | 0.0076 | LOC_Os01g29820 | expressed protein |
| Os.38045.1.S1_at | 2.58 | 0.0147 | LOC_Os12g08810 | VTC2, putative, expressed |
| Os.38048.2.S1_x_at | 3.26 | 0.0042 | LOC_Os01g01790 | expressed protein |
| Os.38152.1.S1_x_at | 52.29 | 0.0152 | LOC_Os10g42780 | LrgB-like family protein |
| Os.38157.1.S1_s_at | 1.71 | 0.0301 | LOC_Os09g39910 | ATP-binding cassette sub-family F member 2, putative |
| Os.3818.1.S1_a_at | 7.39 | 0.0008 | LOC_Os01g09280 | myb-like DNA-binding domain, SHAQKYF class family protein, |
| Os.38256.1.S1_at | 2.49 | 0.0409 | LOC_Os03g16460 | HEAT repeat family protein, expressed |
| Os.38257.1.S1_x_at | 3.23 | 0.0123 | LOC_Os12g08730 | Thioredoxin M-type, chloroplast precursor, putative, |
| Os.38283.1.S2_a_at | 6.28 | 0.0028 | LOC_Os05g41550 | expressed protein |
| Os.38283.1.S3_a_at | 6.03 | 0.0017 | LOC_Os05g41550 | expressed protein |
| Os.38365.1.S1_a_at | 3.60 | 0.0221 | LOC_Os05g47560 | Serine/threonine-protein kinase SNT7, chloroplast precursor |
| Os.38378.1.S1_a_at | 3.77 | 0.0487 | LOC_Os01g01340 | cytokinin-repressed protein CR9, putative, expressed |
| Os.3838.1.S1_at | 2.64 | 0.0101 | LOC_Os01g13540 | RWP-RK domain containing protein, expressed |
| Os.38386.1.A1_at | 2.84 | 0.0163 | LOC_Os04g42030 | expressed protein |
| Os.38416.1.A1_at | 3.21 | 0.0142 | LOC_Os03g50270 | expressed protein |
| Os.38421.1.S1_at | 20.76 | 0.0365 | LOC_Os01g57560 | S-locus glycoprotein family protein, expressed |
| Os.38760.1.S1_at | 15.81 | 0.0090 | Os05g0348100 | Hypothetical protein. |
| Os.38760.1.S1_s_at | 35.14 | 0.0189 | LOC_Os05g28090 | expressed protein |
| Os.38764.1.S1_x_at | 4.05 | 0.0082 | LOC_Os04g18910 | retrotransposon protein, putative, Ty3-gypsy |
| Os.38774.1.S1_s_at | 1.86 | 0.0438 | LOC_Os11g03050 | phosphoethanolamine cytidylyltransferase, putative, expressed |
| Os.38848.1.S1_at | 13.18 | 0.0002 | LOC_Os06g15330 | CCT motif family protein, expressed |
| Os.39020.1.S1_at | 3.26 | 0.0195 | LOC_Os02g11780 | Transcription factor S-II, central domain containing protein |
| Os.39038.1.A1_at | 15.50 | 0.0411 | Os12g0274700 | Ribulose-1,5-bisphosphate carboxylase/oxygenase small subunit (Fragment). |
| Os.39063.1.A1_s_at | 2.74 | 0.0110 | Os03g0387900 | Hypothetical protein. |
| Os.39065.1.A1_s_at | 3.06 | 0.0041 | LOC_Os02g52390 | Peptidase M16 inactive domain containing protein, expressed |
| Os.39257.1.S1_at | 2.38 | 0.0231 | LOC_Os12g43340 | Actin-depolymerizing factor, putative, expressed |
| Os.39363.1.A1_x_at | 6.29 | 0.0330 | LOC_Os12g17600 | Ribulose bisphosphate carboxylase small chain C |
| Os.39984.1.S1_at | 2.15 | 0.0050 | Os08g0313200 | CBS domain containing protein. |
| Os.39989.1.S1_s_at | 2.29 | 0.0389 | LOC_Os08g31460 | tRNA modification GTPase TrmE family protein |
| Os.40410.1.A1_s_at | 1.85 | 0.0197 | LOC_Os03g44500 | kelch repeat-containing serine/threonine phosphoesterase |
| Os.40415.1.A1_s_at | 1.92 | 0.0453 | LOC_Os05g49060 | expressed protein |
| Os.4148.1.S2_a_at | 3.10 | 0.0030 | LOC_Os06g44230 | DNA-directed RNA polymerase 3, chloroplast precursor |
| Os.41694.1.S1_at | 6.87 | 0.0197 | LOC_Os01g49120 | MATE efflux family protein, expressed |
| Os.4177.1.S1_at | 3.00 | 0.0155 | LOC_Os03g55930 | 30S ribosomal protein S9, chloroplast precursor, putative, |
| Os.41793.1.S1_at | 1.89 | 0.0014 | LOC_Os01g47450 | carboxyl-terminal protease family protein, expressed |
| Os.4180.1.S1_at | 3.09 | 0.0190 | LOC_Os02g36380 | Cryptochrome 1 apoprotein, putative, expressed |
| Os.42075.1.A1_x_at | 4.19 | 0.0000 | LOC_Os01g55290 | pentatricopeptide, putative, expressed |
| Os.4291.1.S1_at | 3.51 | 0.0442 | LOC_Os06g03930 | Cytochrome P450 family protein, expressed |
| Os.42960.1.S1_x_at | 2.12 | 0.0222 | LOC_Os01g73960 | Drought induced 19 protein containing protein, expressed |
| Os.4324.1.S1_at | 11.15 | 0.0088 | LOC_Os03g03670 | expressed protein |
| Os.4329.1.S1_at | 2.57 | 0.0188 | LOC_Os03g03720 | Glyceraldehyde-3-phosphate dehydrogenase B |
| Os.43375.1.S1_at | 1.90 | 0.0348 | unknown | unknown |
| Os.435.1.S1_at | 12.68 | 0.0009 | LOC_Os01g17240 | Major Facilitator Superfamily protein, expressed |
| Os.4387.1.S1_at | 1.99 | 0.0063 | LOC_Os01g15640 | No apical meristem protein, expressed |
| Os.43896.1.S1_at | 5.48 | 0.0032 | LOC_Os03g08910 | MatE family protein, expressed |
| Os.43898.1.S1_x_at | 3.21 | 0.0277 | LOC_Os03g01830 | DEAD/DEAH box helicase, putative, expressed |
| Os.44471.2.S1_at | 2.37 | 0.0108 | LOC_Os07g37840 | hydrolase, alpha/beta fold family protein, expressed |
| Os.4459.2.S1_x_at | 1.53 | 0.0044 | LOC_Os06g03600 | transcriptional co-regulator family protein, putative |
| Os.44735.1.S1_at | 6.96 | 0.0027 | LOC_Os06g07760 | expressed protein |
| Os.45018.1.S1_x_at | 8.13 | 0.0129 | LOC_Os03g25500 | Cytochrome P450 72A1, putative, expressed |
| Os.4518.1.S1_a_at | 5.23 | 0.0202 | LOC_Os06g02490 | Acyl-CoA-binding protein, putative, expressed |
| Os.4531.1.S1_at | 1.90 | 0.0311 | LOC_Os03g63490 | Pantoate-beta-alanine ligase, putative, expressed |
| Os.45906.1.S1_at | 3.54 | 0.0021 | LOC_Os01g58390 | HAD superfamily phosphatase, TIGR01668 containing protein |
| Os.45975.1.S1_a_at | 1.76 | 0.0312 | LOC_Os03g27250 | transcription factor jumonji, putative, expressed |
| Os.4603.1.S1_at | 2.85 | 0.0349 | LOC_Os02g58500 | phospholipase A2, putative, expressed |
| Os.46062.1.S1_at | 2.50 | 0.0391 | LOC_Os10g13940 | MATE efflux family protein, putative, expressed |
| Os.46065.2.S1_x_at | 1.88 | 0.0352 | LOC_Os10g32960 | Pex2/Pex12 amino terminal region family protein, expressed |
| Os.46070.1.S1_at | 2.19 | 0.0137 | Os10g0118900 | Hypothetical protein. |
| Os.46070.1.S1_x_at | 2.47 | 0.0109 | LOC_Os10g02930 | Helicase SEN1, putative, expressed |
| Os.46071.1.S1_at | 9.12 | 0.0199 | LOC_Os12g23180 | RNA binding protein, putative, expressed |
| Os.4608.3.A1_a_at | 5.80 | 0.0046 | LOC_Os06g43860 | Homeobox protein knotted-1-like 3, putative, expressed |
| Os.46135.1.S1_x_at | 1.79 | 0.0104 | Os10g0527100 | Chloroplast ATP synthase a chain precursor |
| Os.46208.1.S1_x_at | 2.90 | 0.0163 | LOC_Os10g21230 | ATP synthase subunit C family protein |
| Os.46223.1.S1_s_at | 2.22 | 0.0022 | LOC_Os09g33690 | retrotransposon protein, putative, unclassified, expressed |
| Os.4627.1.S1_x_at | 1.66 | 0.0067 | LOC_Os07g48100 | CBL-interacting serine/threonine-protein kinase 15 |
| Os.46288.1.A1_at | 5.42 | 0.0130 | Os12g0188700 | Thioredoxin (TRX). |
| Os.46288.1.A1_x_at | 4.07 | 0.0141 | LOC_Os12g08730 | Thioredoxin M-type, chloroplast precursor, putative |
| Os.46305.1.S1_at | 1.67 | 0.0432 | LOC_Os11g01780 | Leucine Rich Repeat family protein, expressed |
| Os.46321.1.A1_s_at | 3.40 | 0.0007 | Os03g0772300 | Conserved hypothetical protein. |
| Os.46329.1.S1_at | 1.98 | 0.0443 | LOC_Os09g38550 | Protein phosphatase 2C containing protein, expressed |
| Os.4633.1.S1_at | 3.01 | 0.0002 | LOC_Os01g59970 | expressed protein |
| Os.4635.1.S1_at | 1.63 | 0.0218 | LOC_Os09g25590 | Tsi1-interacting protein TSIP1, putative, expressed |
| Os.46361.1.A1_at | 3.09 | 0.0237 | unknown | unknown |
| Os.46382.3.S1_at | 3.14 | 0.0161 | Os10g0497500 | KH, type 1 domain containing protein. |
| Os.46383.2.A1_s_at | 3.74 | 0.0070 | LOC_Os02g47020 | Phosphoribulokinase, chloroplast precursor, putative |
| Os.46386.1.S1_at | 3.28 | 0.0447 | LOC_Os06g40640 | Fructose-bisphosphate aldolase, cytoplasmic isozyme 1 |
| Os.46398.1.S1_at | 1.77 | 0.0175 | Os01g0149200 | Metallothionein-like protein type 2. |
| Os.46403.1.S1_at | 2.64 | 0.0115 | LOC_Os01g69950 | 50S ribosomal protein L27, chloroplast precursor, putative |
| Os.46404.1.S1_x_at | 5.81 | 0.0034 | LOC_Os08g35420 | Photosystem Q, putative, expressed |
| Os.4642.1.A1_s_at | 2.31 | 0.0010 | LOC_Os09g24230 | haloacid dehalogenase-like hydrolase family protein |
| Os.46422.1.S1_at | 1.85 | 0.0071 | LOC_Os07g25430 | Photosystem I reaction centre subunit IV/PsaE family protein |
| Os.46437.1.S1_s_at | 4.42 | 0.0004 | LOC_Os04g16780 | Chloroplast 30S ribosomal protein S3, putative, expressed |
| Os.4647.1.S1_at | 1.95 | 0.0210 | LOC_Os05g03430 | Sumoylation ligase E3, putative, expressed |
| Os.46498.1.S1_at | 2.26 | 0.0330 | LOC_Os10g11310 | expressed protein |
| Os.46498.2.S1_x_at | 2.17 | 0.0456 | LOC_Os10g11310 | expressed protein |
| Os.46525.1.S1_at | 3.16 | 0.0234 | Os10g0198600 | Intron-encoded nuclease 2 domain containing protein. |
| Os.46525.2.S1_x_at | 2.75 | 0.0012 | Os10g0198600 | Intron-encoded nuclease 2 domain containing protein. |
| Os.46542.1.S1_at | 25.12 | 0.0083 | Os10g0394100 | Metallophosphoesterase domain containing protein. |
| Os.46545.1.S1_at | 1.69 | 0.0035 | LOC_Os10g24900 | Kelch motif family protein, expressed |
| Os.46545.2.S1_x_at | 1.89 | 0.0181 | LOC_Os10g24900 | Kelch motif family protein, expressed |
| Os.46546.1.S1_at | 1.65 | 0.0239 | LOC_Os10g22560 | POT family protein, expressed |
| Os.46548.2.S1_at | 3.86 | 0.0052 | LOC_Os10g39930 | Cytochrome P450 family protein, expressed |
| Os.46591.1.S1_x_at | 12.05 | 0.0036 | LOC_Os10g35530 | epoxide hydrolase, putative, expressed |
| Os.46592.2.S1_x_at | 1.95 | 0.0302 | Os10g0514400 | Conserved hypothetical protein. |
| Os.46598.1.S1_a_at | 4.29 | 0.0207 | LOC_Os04g42020 | CONSTANS, putative, expressed |
| Os.4661.1.S1_at | 2.34 | 0.0023 | LOC_Os06g47890 | ZEITLUPE, putative, expressed |
| Os.46623.1.S1_at | 4.44 | 0.0345 | LOC_Os10g40830 | Matrixin family protein, expressed |
| Os.46626.1.S1_x_at | 2.00 | 0.0120 | LOC_Os10g40750 | expressed protein |
| Os.46632.1.S1_at | 3.96 | 0.0387 | LOC_Os10g39800 | hypothetical protein |
| Os.46634.1.S1_at | 3.33 | 0.0244 | Os10g0528200 | Tau class GST protein 3. |
| Os.46634.1.S1_x_at | 9.09 | 0.0174 | LOC_Os10g38340 | glutathione S-transferase GSTU6, putative, expressed |
| Os.46638.1.S1_at | 17.44 | 0.0055 | LOC_Os10g37770 | methyltransferase family protein, expressed |
| Os.46641.1.S1_x_at | 5.29 | 0.0366 | LOC_Os10g37330 | aldo/keto reductase family protein, putative, expressed |
| Os.46698.2.S1_x_at | 4.57 | 0.0217 | LOC_Os10g20380 | TRANSPARENT TESTA 12 protein, putative, expressed |
| Os.46774.1.S1_at | 2.62 | 0.0013 | LOC_Os03g54900 | Leucine Rich Repeat family protein, expressed |
| Os.46883.1.S1_at | 3.50 | 0.0129 | LOC_Os10g42180 | Uncharacterized protein family UPF0005 containing protein, |
| Os.4689.1.S1_at | 1.73 | 0.0207 | LOC_Os03g05730 | Cell division cycle protein 48 homolog, putative, expressed |
| Os.4701.1.S1_at | 1.87 | 0.0285 | Os01g0678600 | Ribosomal protein S20 family protein. |
| Os.47073.1.S1_at | 3.45 | 0.0312 | unknown | unknown |
| Os.47087.1.S1_at | 1.57 | 0.0440 | Os07g0483400 | Conserved hypothetical protein. |
| Os.4730.1.S1_at | 2.11 | 0.0319 | Os02g0701700 | Hypothetical protein. |
| Os.47301.1.A1_at | 6.50 | 0.0028 | LOC_Os04g45600 | expressed protein |
| Os.47349.1.S1_at | 1.64 | 0.0036 | LOC_Os03g04390 | pentatricopeptide, putative, expressed |
| Os.47358.1.A1_at | 4.96 | 0.0172 | LOC_Os08g39430 | expressed protein |
| Os.47372.1.S1_x_at | 2.30 | 0.0437 | LOC_Os04g46300 | unspliced-genomic disease resistance protein RPM1,putative, expressed |
| Os.47392.1.A1_x_at | 1.61 | 0.0216 | LOC_Os06g04030 | Histone H3, putative, expressed |
| Os.47398.2.A1_at | 2.11 | 0.0089 | LOC_Os02g46140 | expressed protein |
| Os.47438.1.A1_at | 4.81 | 0.0271 | Os10g0528100 | Glutathione S-transferase GST 42 |
| Os.47575.1.S1_at | 5.33 | 0.0026 | LOC_Os02g38430 | expressed protein |
| Os.47600.1.S1_s_at | 2.43 | 0.0102 | LOC_Os02g54730 | Transmembrane amino acid transporter protein, expressed |
| Os.47644.1.S1_at | 2.16 | 0.0076 | Os05g0432700 | Proteinase inhibitor I9, subtilisin propeptide domain containing protein. |
| Os.47660.1.S1_s_at | 1.61 | 0.0346 | LOC_Os05g03480 | Isovaleryl-CoA dehydrogenase, mitochondrial precursor |
| Os.47679.1.S1_at | 4.41 | 0.0032 | Os12g0420400 | Photosystem I reaction center subunit XI, chloroplast precursor |
| Os.47706.1.S1_at | 5.87 | 0.0071 | LOC_Os03g59090 | cell death suppressor protein Lls1 homolog, putative |
| Os.47708.1.A1_at | 3.02 | 0.0182 | Os07g0495100 | Possible protein kinase CK2 regulatory subunit (Fragment). |
| Os.47712.1.S1_at | 65.56 | 0.0061 | LOC_Os05g28090 | expressed protein |
| Os.47784.1.S1_at | 2.31 | 0.0104 | LOC_Os06g45340 | FK506-binding protein, putative, expressed |
| Os.47818.1.A1_at | 1.66 | 0.0343 | LOC_Os04g23820 | tRNA binding domain containing protein, putative, expressed |
| Os.47826.1.S1_at | 4.04 | 0.0097 | LOC_Os06g05700 | Cysteine synthase, putative, expressed |
| Os.47908.1.S1_at | 2.74 | 0.0016 | LOC_Os02g21110 | Kelch motif family protein, expressed |
| Os.47912.1.S1_at | 2.86 | 0.0260 | LOC_Os05g49610 | membrane associated salt-inducible protein, putative |
| Os.47913.1.A1_at | 3.73 | 0.0008 | Os09g0565200 | 31 kDa ribonucleoprotein, chloroplast precursor. |
| Os.47951.1.S1_at | 2.49 | 0.0394 | LOC_Os03g14290 | expressed protein |
| Os.47958.1.A1_x_at | 1.85 | 0.0390 | LOC_Os01g61410 | Rotenone-insensitive NADH-ubiquinone oxidoreductase |
| Os.48020.1.S1_x_at | 2.12 | 0.0400 | LOC_Os03g56660 | expressed protein |
| Os.48038.1.S1_at | 1.82 | 0.0084 | LOC_Os02g55340 | HEAT repeat family protein, expressed |
| Os.48062.1.S1_s_at | 2.37 | 0.0091 | LOC_Os02g38980 | global transcription factor group E, putative, expressed |
| Os.48079.1.S1_at | 4.69 | 0.0007 | LOC_Os07g38150 | expressed protein |
| Os.48107.1.A1_at | 6.69 | 0.0001 | Os02g0794600 | Cytochrome c oxidase copper chaperone (Dopuin). |
| Os.48148.1.S1_at | 2.76 | 0.0349 | LOC_Os08g42010 | nodulin, putative, expressed |
| Os.4831.1.S1_a_at | 2.20 | 0.0046 | LOC_Os02g38210 | Elongation factor Tu, chloroplast precursor, |
| Os.48332.1.A1_s_at | 2.46 | 0.0162 | LOC_Os07g39010 | lactose permease, putative, expressed |
| Os.48352.1.A1_at | 2.59 | 0.0042 | LOC_Os03g60670 | unspliced-genomic retrotransposon protein,putative, unclassified |
| Os.48438.1.S1_at | 4.51 | 0.0361 | Os03g0152700 | RNA-binding S4 domain containing protein. |
| Os.4851.1.S1_at | 1.75 | 0.0432 | LOC_Os04g52400 | oxidoreductase, short chain dehydrogenase/reductase family |
| Os.4853.1.S2_at | 1.72 | 0.0053 | LOC_Os09g35710 | UBX domain containing protein, expressed |
| Os.48545.1.S1_at | 5.93 | 0.0038 | LOC_Os04g53810 | NAD dependent epimerase/dehydratase family protein |
| Os.48588.1.A1_at | 2.25 | 0.0015 | LOC_Os05g05740 | Proline synthetase co-transcribed bacterial homolog protein |
| Os.4859.1.S1_at | 4.46 | 0.0083 | LOC_Os12g12850 | ATP-dependent Clp protease ATP-binding subunit clpA homology |
| Os.48834.1.S1_at | 2.81 | 0.0006 | LOC_Os09g34900 | bile acid:sodium symporter family protein, putative, |
| Os.4887.1.S1_at | 1.62 | 0.0415 | Os11g0229000 | Hypothetical protein. |
| Os.48911.1.A1_s_at | 2.74 | 0.0247 | Os03g0772300 | Conserved hypothetical protein. |
| Os.48919.1.S1_at | 1.66 | 0.0133 | LOC_Os02g04800 | avirulence-responsive protein, putative, expressed |
| Os.4899.1.S1_a_at | 1.74 | 0.0225 | LOC_Os01g60190 | 2,3-bisphosphoglycerate-independent phosphoglycerate mutase |
| Os.49084.1.S1_at | 3.15 | 0.0181 | LOC_Os06g11140 | NADH dehydrogenase, putative, expressed |
| Os.49087.1.A1_at | 4.58 | 0.0007 | LOC_Os05g19380 | pentatricopeptide, putative, expressed |
| Os.49107.1.A1_at | 8.79 | 0.0150 | unknown | unknown |
| Os.49148.2.S1_at | 1.89 | 0.0190 | LOC_Os01g55890 | expressed protein |
| Os.49149.1.S1_at | 8.95 | 0.0048 | LOC_Os04g34460 | plastid-lipid associated protein PAP, putative, expressed |
| Os.4917.1.S1_at | 3.65 | 0.0005 | LOC_Os05g33500 | mitochondrial transcription termination factor family protein |
| Os.49170.1.S1_at | 4.93 | 0.0116 | LOC_Os03g29680 | EARLY flowering 4 protein, putative, expressed |
| Os.49184.1.S1_at | 2.60 | 0.0038 | LOC_Os08g39990 | expressed protein |
| Os.49185.1.S1_at | 57.59 | 0.0210 | LOC_Os04g45210 | Peroxisomal biogenesis factor 11 family protein, expressed |
| Os.49187.1.S1_at | 1.89 | 0.0002 | LOC_Os02g03850 | Glycosyl transferase family, a/b domain containing protein |
| Os.49212.1.S1_a_at | 2.34 | 0.0053 | Os12g0193100 | Subtilisin inhibitor I (ASI-I) [Contains: Subtilisin inhibitor II (ASI-II)]. |
| Os.49216.1.S1_at | 2.58 | 0.0299 | LOC_Os05g22930 | CAAX amino terminal protease family protein |
| Os.49228.1.S1_at | 3.22 | 0.0122 | LOC_Os02g30410 | expressed protein |
| Os.49230.1.S1_at | 11.52 | 0.0000 | LOC_Os03g04450 | expressed protein |
| Os.49239.1.S1_at | 4.23 | 0.0115 | LOC_Os05g32390 | GTP-binding protein, putative, expressed |
| Os.49267.1.S1_at | 2.92 | 0.0201 | LOC_Os08g40420 | unspliced-genomic ternary complex factor MIP1,putative, expressed |
| Os.49277.1.S1_at | 28.92 | 0.0126 | LOC_Os01g53020 | DnaJ domain containing protein, expressed |
| Os.49353.1.S1_at | 1.91 | 0.0389 | LOC_Os03g10230 | expressed protein |
| Os.49393.1.S1_at | 2.13 | 0.0216 | LOC_Os11g38030 | CDK5RAP1, putative, expressed |
| Os.49406.1.S1_at | 2.20 | 0.0139 | LOC_Os02g29510 | Non-imprinted in Prader-Willi/Angelman syndrome region protein |
| Os.49419.1.S1_at | 2.04 | 0.0319 | LOC_Os11g39320 | NB-ARC domain containing protein, expressed |
| Os.49423.1.S1_at | 4.98 | 0.0017 | Os11g0153700 | Signal recognition particle 54 kDa protein, chloroplast precursor (SRP54) |
| Os.49474.1.S1_at | 4.42 | 0.0058 | LOC_Os02g45660 | 2-C-methyl-D-erythritol 2,4-cyclodiphosphate synthase |
| Os.4948.1.S1_x_at | 2.84 | 0.0031 | LOC_Os02g42290 | ATP-dependent Clp protease proteolytic subunit, |
| Os.49486.1.S1_at | 2.77 | 0.0136 | LOC_Os12g36670 | Leucine Rich Repeat family protein, expressed |
| Os.49493.1.S1_at | 1.54 | 0.0267 | LOC_Os11g05120 | expressed protein |
| Os.4951.1.S1_at | 1.71 | 0.0055 | LOC_Os05g22670 | Transcription factor IIA, alpha/beta subunit family protein |
| Os.49535.1.A1_at | 1.66 | 0.0265 | Os02g0461600 | Conserved hypothetical protein. |
| Os.49556.1.S1_at | 4.10 | 0.0247 | LOC_Os01g12830 | D-isomer specific 2-hydroxyacid dehydrogenase, NAD binding protein |
| Os.49580.1.S2_x_at | 4.47 | 0.0222 | Os12g0290200 | Hypothetical protein. |
| Os.49621.1.S1_at | 2.24 | 0.0134 | LOC_Os07g25590 | Tyrosine decarboxylase 1, putative, expressed |
| Os.49624.1.S1_at | 3.12 | 0.0005 | LOC_Os05g33520 | expressed protein |
| Os.49694.1.S1_at | 4.26 | 0.0157 | LOC_Os03g62370 | WD40-like Beta Propeller Repeat family protein, expressed |
| Os.49705.1.S1_at | 4.82 | 0.0035 | LOC_Os08g31750 | methyltransferase, putative, expressed |
| Os.49718.1.S1_at | 3.97 | 0.0031 | LOC_Os05g03000 | Integral membrane protein TerC family protein, expressed |
| Os.49739.1.S2_at | 2.23 | 0.0079 | LOC_Os03g22670 | triacylglycerol Lipase, putative, expressed |
| Os.49742.1.S1_at | 2.04 | 0.0167 | LOC_Os08g19210 | expressed protein |
| Os.49745.1.S1_at | 1.64 | 0.0419 | LOC_Os03g41460 | Serine/threonine-protein kinase SAPK9, putative, expressed |
| Os.49780.1.S1_x_at | 1.56 | 0.0023 | LOC_Os04g51050 | wall-associated kinase 1, putative, expressed |
| Os.49797.1.A1_at | 5.46 | 0.0009 | LOC_Os02g08140 | Protein kinase domain containing protein, expressed |
| Os.49814.1.S1_at | 4.43 | 0.0303 | Os09g0552300 | Protein kinase domain containing protein. |
| Os.49832.1.S1_at | 3.29 | 0.0223 | LOC_Os08g29980 | retrotransposon protein, putative, unclassified, expressed |
| Os.49838.1.S1_at | 2.98 | 0.0296 | LOC_Os04g47720 | Cis-zeatin O-glucosyltransferase 1, putative, expressed |
| Os.49855.1.S1_at | 4.60 | 0.0054 | LOC_Os02g57560 | Protein kinase domain containing protein, expressed |
| Os.49864.2.S1_x_at | 1.76 | 0.0493 | LOC_Os03g63470 | transducin family protein, putative, expressed |
| Os.4995.1.S1_at | 1.85 | 0.0413 | Os03g0766800 | Conserved hypothetical protein. |
| Os.49951.1.S1_at | 5.18 | 0.0032 | LOC_Os07g48690 | expressed protein |
| Os.5008.1.S1_at | 1.56 | 0.0275 | LOC_Os05g05310 | Fibronectin type III domain containing protein, expressed |
| Os.5010.1.S1_at | 6.18 | 0.0366 | LOC_Os03g61060 | Protein kinase domain containing protein, expressed |
| Os.50204.1.S1_at | 4.35 | 0.0086 | LOC_Os03g11780 | expressed protein |
| Os.50236.1.S1_at | 1.79 | 0.0449 | LOC_Os11g40540 | Sugar transporter, putative, expressed |
| Os.50251.1.S1_at | 4.09 | 0.0269 | LOC_Os09g07660 | ABC1 family protein, expressed |
| Os.50298.1.S1_at | 2.91 | 0.0140 | LOC_Os04g51350 | pentatricopeptide, putative, expressed |
| Os.50299.1.S1_at | 4.78 | 0.0019 | Os02g0470000 | TPR-like domain containing protein. |
| Os.50313.1.S1_at | 5.22 | 0.0213 | LOC_Os07g28790 | PAP_fibrillin family protein, expressed |
| Os.50420.1.S1_s_at | 2.13 | 0.0221 | LOC_Os05g01950 | expressed protein |
| Os.50421.1.S1_at | 2.14 | 0.0472 | LOC_Os02g53990 | cyclin, putative, expressed |
| Os.5049.1.S1_at | 15.34 | 0.0286 | LOC_Os10g20470 | MATE efflux family protein, expressed |
| Os.5054.1.S1_at | 2.98 | 0.0266 | LOC_Os06g20320 | trigger factor family protein, expressed |
| Os.50548.2.S1_x_at | 1.78 | 0.0238 | LOC_Os09g17610 | Zinc finger, C3HC4 type family protein, expressed |
| Os.50556.1.S1_at | 5.80 | 0.0333 | LOC_Os12g07280 | Zinc finger, C2H2 type family protein, expressed |
| Os.50588.1.S1_at | 2.99 | 0.0098 | LOC_Os11g34370 | patatin, putative, expressed |
| Os.50753.1.S1_at | 6.51 | 0.0097 | LOC_Os12g13120 | histidine triad family protein, putative, expressed |
| Os.50756.1.S1_at | 1.94 | 0.0053 | LOC_Os09g21250 | expressed protein |
| Os.50766.1.S1_at | 1.78 | 0.0259 | Os06g0269200 | Hypothetical protein. |
| Os.50774.1.S1_at | 2.06 | 0.0309 | LOC_Os02g19170 | expressed protein |
| Os.50778.1.S1_at | 4.58 | 0.0117 | LOC_Os07g43040 | heavy metal-associated domain containing protein, expressed |
| Os.50806.1.A1_at | 2.80 | 0.0024 | LOC_Os05g12300 | unspliced-genomic expressed protein |
| Os.50813.1.S1_at | 1.81 | 0.0165 | Os09g0436800 | Abortive infection protein family protein. |
| Os.5082.1.S1_a_at | 1.78 | 0.0065 | LOC_Os03g61130 | phosphoesterase family protein, putative, expressed |
| Os.50823.1.S1_at | 5.02 | 0.0064 | Os12g0287300 | NIFS-like protein CpNifsp precursor (Cysteine desulfurase) |
| Os.50857.1.S1_at | 3.37 | 0.0041 | LOC_Os03g62690 | pentatricopeptide, putative, expressed |
| Os.50865.1.S1_at | 3.10 | 0.0177 | Os03g0414400 | Conserved hypothetical protein. |
| Os.50871.1.S1_at | 5.29 | 0.0008 | LOC_Os02g50560 | DEAD/DEAH box helicase, putative, expressed |
| Os.50876.1.S1_at | 2.56 | 0.0051 | LOC_Os06g04150 | Magnesium-protoporphyrin O-methyltransferase, putative, |
| Os.50886.1.S1_at | 7.01 | 0.0151 | Os04g0657900 | Conserved hypothetical protein. |
| Os.50910.1.S1_at | 1.83 | 0.0336 | LOC_Os04g45160 | expressed protein |
| Os.50917.1.S1_at | 6.36 | 0.0002 | LOC_Os06g43140 | expressed protein |
| Os.50919.1.S1_at | 8.81 | 0.0306 | LOC_Os11g03580 | expressed protein |
| Os.50932.1.S1_at | 1.87 | 0.0227 | LOC_Os03g48940 | Chloride channel protein CLC-d, putative, expressed |
| Os.50951.1.S1_at | 3.24 | 0.0101 | LOC_Os02g33610 | RNA-metabolising metallo-beta-lactamase family protein, |
| Os.50976.1.S1_at | 3.69 | 0.0259 | LOC_Os03g48040 | ferredoxin family protein, expressed |
| Os.51003.1.S1_at | 7.55 | 0.0115 | LOC_Os05g42280 | expressed protein |
| Os.51043.1.S1_at | 4.75 | 0.0002 | LOC_Os01g07730 | Triose phosphate/phosphate translocator, non-green plastid |
| Os.51052.1.S1_at | 8.15 | 0.0235 | LOC_Os03g50530 | expressed protein |
| Os.51057.1.S1_s_at | 2.82 | 0.0137 | LOC_Os01g43150 | ATP-dependent metalloprotease FtsH family protein |
| Os.51086.1.S1_x_at | 2.79 | 0.0014 | LOC_Os08g02630 | expressed protein |
| Os.51121.1.S1_x_at | 1.70 | 0.0128 | LOC_Os03g51260 | expressed protein |
| Os.51130.1.S1_at | 8.64 | 0.0150 | LOC_Os05g40180 | Protein kinase domain containing protein, expressed |
| Os.51132.1.S1_at | 6.48 | 0.0039 | LOC_Os08g07060 | expressed protein |
| Os.51175.1.S1_x_at | 1.64 | 0.0142 | Os04g0128300 | hypothetical protein |
| Os.51206.1.S1_at | 2.87 | 0.0100 | LOC_Os04g49620 | expressed protein |
| Os.51208.1.S1_s_at | 4.34 | 0.0127 | LOC_Os02g57020 | expressed protein |
| Os.51211.1.S1_at | 4.65 | 0.0012 | LOC_Os02g20490 | expressed protein |
| Os.51212.1.S1_at | 2.58 | 0.0088 | LOC_Os04g42800 | expressed protein |
| Os.51222.1.S1_at | 3.73 | 0.0312 | LOC_Os11g29230 | PPR986-12, putative, expressed |
| Os.51242.1.S1_at | 1.74 | 0.0490 | LOC_Os12g39770 | hypothetical protein |
| Os.51258.1.S1_at | 2.52 | 0.0158 | Os02g0125000 | Conserved hypothetical protein. |
| Os.51349.1.S1_at | 4.22 | 0.0299 | LOC_Os02g53870 | expressed protein |
| Os.5136.1.S1_a_at | 3.60 | 0.0021 | Os02g0766000 | DCL protein, chloroplast precursor |
| Os.5136.1.S1_at | 3.94 | 0.0005 | Os02g0766000 | DCL protein, chloroplast precursor |
| Os.5136.2.S1_x_at | 3.53 | 0.0001 | Os02g0766000 | DCL protein, chloroplast precursor |
| Os.51374.1.S1_at | 4.08 | 0.0280 | LOC_Os09g30410 | expressed protein |
| Os.51391.1.S1_at | 3.54 | 0.0211 | LOC_Os02g53350 | expressed protein |
| Os.51462.1.S1_at | 4.22 | 0.0129 | Os10g0548000 | Short-chain dehydrogenase Tic32. |
| Os.51464.1.A1_at | 6.78 | 0.0052 | LOC_Os01g58060 | NADH dehydrogen |
| Os.51473.2.S1_x_at | 4.46 | 0.0161 | LOC_Os05g48160 | ferredoxin, putative, expressed |
| Os.51478.1.S1_at | 4.17 | 0.0090 | LOC_Os12g17830 | expressed protein |
| Os.51504.1.A1_at | 17.54 | 0.0046 | LOC_Os01g579 | 41 cytochrome b6-f complex subunit 4, putative, expressed |
| Os.5151.1.S1_at | 1.90 | 0.0411 | LOC_Os03g52450 | CCT motif family protein, expressed |
| Os.5160.1.S1_at | 1.76 | 0.0001 | LOC_Os07g46330 | expressed protein |
| Os.5170.1.S1_at | 2.83 | 0.0416 | LOC_Os06g28950 | FKBP-type peptidyl-prolyl cis-trans isomerase, putative |
| Os.5178.1.A1_s_at | 5.73 | 0.0018 | LOC_Os03g56950 | Helix-loop-helix DNA-binding domain containing protein |
| Os.51804.1.S1_at | 4.69 | 0.0214 | LOC_Os02g46440 | ribosomal protein L18 family protein, putative, expressed |
| Os.51807.1.S1_at | 2.51 | 0.0372 | LOC_Os01g45460 | Carboxylesterase, putative, expressed |
| Os.5188.2.S1_s_at | 11.48 | 0.0364 | LOC_Os08g23410 | Rubredoxin family protein, expressed |
| Os.51896.1.S1_at | 4.61 | 0.0008 | LOC_Os02g49470 | tic20 protein, putative, expressed |
| Os.5191.1.S1_at | 3.84 | 0.0063 | LOC_Os05g04620 | expressed protein |
| Os.51926.1.S1_at | 4.16 | 0.0013 | LOC_Os11g08210 | unspliced-genomic no apical meristem protein, putative, expressed |
| Os.51926.1.S1_x_at | 3.34 | 0.0043 | LOC_Os11g08210 | NAC-domain containing protein 2, putative, expressed |
| Os.51983.1.A1_x_at | 1.89 | 0.0271 | LOC_Os01g12470 | Mitochondrial carrier protein, expressed |
| Os.51989.1.S2_at | 2.68 | 0.0207 | LOC_Os07g37220 | PAP-specific phosphatase, mitochondrial precursor |
| Os.5206.1.S1_at | 2.62 | 0.0078 | LOC_Os02g51790 | 50S ribosomal protein L29, chloroplast precursor, putative |
| Os.52104.1.S1_at | 3.74 | 0.0313 | LOC_Os02g10590 | peptidyl-prolyl cis-trans isomerase, FKBP-type family protein |
| Os.52106.1.S1_at | 5.92 | 0.0204 | LOC_Os02g51450 | mTERF family protein, expressed |
| Os.52130.1.S1_at | 2.91 | 0.0067 | LOC_Os02g50280 | pentatricopeptide, putative, expressed |
| Os.52161.1.S1_x_at | 9.07 | 0.0079 | LOC_Os04g22900 | Protein GOS9, putative, expressed |
| Os.52168.1.S1_at | 2.05 | 0.0073 | LOC_Os12g35060 | expressed protein |
| Os.52207.1.S1_at | 2.17 | 0.0177 | LOC_Os08g44560 | expressed protein |
| Os.52224.1.S1_at | 9.03 | 0.0003 | Os03g0284900 | Protein prenyltransferase domain containing protein. |
| Os.52226.1.S1_at | 2.85 | 0.0175 | LOC_Os07g41630 | expressed protein |
| Os.52229.1.S1_at | 4.76 | 0.0084 | LOC_Os05g45300 | enoyl-CoA hydratase/isomerase family protein, expressed |
| Os.52278.1.S1_s_at | 11.67 | 0.0049 | LOC_Os07g29410 | thioredoxin family protein, putative, expressed |
| Os.52283.1.S1_at | 8.16 | 0.0015 | LOC_Os11g11210 | ATP-dependent protease La, putative |
| Os.5229.1.S1_at | 4.67 | 0.0237 | LOC_Os05g33280 | expressed protein |
| Os.52297.1.S1_at | 2.68 | 0.0004 | Os12g0564600 | Protein of unknown function DUF814 domain containing protein. |
| Os.52346.1.S1_at | 5.52 | 0.0067 | LOC_Os05g45080 | UDP-glucoronosyl and UDP-glucosyl transferase family protein |
| Os.52358.1.S1_at | 2.01 | 0.0174 | LOC_Os06g28590 | U-box domain containing protein, expressed |
| Os.52385.1.S1_at | 3.40 | 0.0364 | LOC_Os02g37600 | expressed protein |
| Os.52400.1.S1_at | 2.77 | 0.0466 | LOC_Os12g17070 | oxygen-independent coproporphyrinogen III oxidase family protein |
| Os.52409.1.S1_at | 1.93 | 0.0335 | LOC_Os10g04620 | hydrolase, alpha/beta fold family protein, putative |
| Os.52416.1.S1_at | 3.03 | 0.0035 | LOC_Os02g49820 | RNA recognition motif family protein, expressed |
| Os.52420.1.A1_at | 2.25 | 0.0156 | LOC_Os09g12540 | Chlorophyll a-b binding protein E, chloroplast precursor, |
| Os.52426.1.S1_at | 2.25 | 0.0115 | Os03g0751800 | Conserved hypothetical protein. |
| Os.52427.1.S1_at | 3.21 | 0.0042 | LOC_Os10g25570 | expressed protein |
| Os.52433.1.S1_at | 6.15 | 0.0364 | LOC_Os04g40530 | expressed protein |
| Os.52459.1.S1_at | 4.03 | 0.0002 | LOC_Os02g57010 | 29 kDa ribonucleoprotein, chloroplast, putative, expressed |
| Os.52467.1.S1_at | 2.53 | 0.0282 | Os05g0516300 | Conserved hypothetical protein. |
| Os.52470.1.S1_at | 2.59 | 0.0186 | LOC_Os03g48190 | expressed protein |
| Os.52476.1.S1_at | 3.76 | 0.0104 | LOC_Os02g02770 | pentatricopeptide, putative, expressed |
| Os.52479.1.S1_at | 1.73 | 0.0296 | LOC_Os05g12130 | pentatricopeptide, putative, expressed |
| Os.52497.1.S1_at | 2.01 | 0.0036 | Os03g0729700 | Conserved hypothetical protein 46 family protein. |
| Os.52507.1.S1_at | 5.34 | 0.0020 | LOC_Os01g43120 | DEAD/DEAH box helicase family protein, expressed |
| Os.52515.1.S1_at | 5.15 | 0.0140 | Os07g0569600 | Conserved hypothetical protein. |
| Os.52523.1.S1_at | 2.54 | 0.0305 | LOC_Os02g09340 | expressed protein |
| Os.52535.1.S1_at | 5.23 | 0.0009 | Os09g0439500 | Chlorophyll A-B binding protein family protein. |
| Os.52535.1.S1_x_at | 4.74 | 0.0066 | LOC_Os09g26810 | Chlorophyll a-b binding protein, chloroplast precursor, |
| Os.52538.1.S1_at | 4.07 | 0.0223 | LOC_Os12g10660 | Salt tolerance-like protein At1g78600, putative, expressed |
| Os.52545.1.S1_at | 2.29 | 0.0153 | LOC_Os08g07880 | DNA/pantothenate metabolism flavoprotein, expressed |
| Os.52551.1.S1_at | 1.63 | 0.0119 | LOC_Os12g42210 | Protease Do-like 5, chloroplast precursor, putative, |
| Os.52584.1.S1_at | 1.77 | 0.0487 | LOC_Os05g05230 | homeotic gene regulator, putative, expressed |
| Os.52588.1.S1_at | 6.91 | 0.0069 | LOC_Os07g30670 | 2Fe-2S iron-sulfur cluster binding domain containing protein |
| Os.52602.1.S1_x_at | 1.86 | 0.0180 | Os10g0510500 | Auxin responsive SAUR protein family protein. |
| Os.5261.1.S1_a_at | 4.58 | 0.0028 | LOC_Os03g20100 | 30S ribosomal protein S1, chloroplast precursor, putative, |
| Os.52638.1.S1_at | 3.12 | 0.0021 | LOC_Os02g58120 | expressed protein |
| Os.52647.1.S1_at | 5.27 | 0.0230 | LOC_Os03g53710 | Aldose 1-epimerase family protein, expressed |
| Os.52657.1.S1_at | 8.71 | 0.0202 | LOC_Os04g42590 | expressed protein |
| Os.52658.1.S1_at | 1.95 | 0.0300 | LOC_Os05g34500 | expressed protein |
| Os.52666.1.S1_at | 7.07 | 0.0116 | LOC_Os10g02240 | POT family protein, expressed |
| Os.52679.1.S1_at | 3.40 | 0.0319 | LOC_Os06g07550 | pentatricopeptide, putative, expressed |
| Os.52757.1.S2_at | 6.00 | 0.0013 | LOC_Os04g25570 | protein phosphatase 2c, putative, expressed |
| Os.52775.1.S1_at | 3.71 | 0.0020 | LOC_Os03g20010 | NifU-like domain containing protein, expressed |
| Os.52809.1.S1_at | 2.60 | 0.0393 | LOC_Os08g39960 | NAD dependent epimerase/dehydratase family protein |
| Os.52820.1.S1_at | 2.14 | 0.0128 | LOC_Os11g22350 | ABC transporter family protein, putative, expressed |
| Os.52821.1.S1_at | 3.53 | 0.0165 | LOC_Os11g37200 | Uncharacterized protein family UPF0005 containing protein, |
| Os.52897.1.S1_at | 4.40 | 0.0194 | Os02g0433600 | Basic helix-loop-helix dimerisation region bHLH domain containing protein. |
| Os.5292.1.S1_at | 1.71 | 0.0393 | LOC_Os03g63040 | expressed protein |
| Os.52929.1.S1_x_at | 1.87 | 0.0202 | LOC_Os01g51370 | expressed protein |
| Os.52934.1.S1_at | 6.90 | 0.0236 | LOC_Os06g14400 | oxidoreductase, 2OG-Fe oxygenase family protein, expressed |
| Os.52947.1.S1_x_at | 2.86 | 0.0076 | LOC_Os09g28490 | EF hand family protein, expressed |
| Os.53001.1.S1_at | 3.41 | 0.0435 | LOC_Os08g19140 | adenylate kinase family protein, putative, expressed |
| Os.53012.1.S1_at | 2.43 | 0.0071 | LOC_Os03g49250 | LysM domain containing protein, expressed |
| Os.53014.1.S1_at | 2.46 | 0.0348 | LOC_Os06g47640 | EF hand family protein, expressed |
| Os.53046.1.S1_at | 6.90 | 0.0438 | Os01g0940600 | Conserved hypothetical protein. |
| Os.53046.1.S1_s_at | 8.28 | 0.0006 | LOC_Os01g71330 | expressed protein |
| Os.5307.1.S1_s_at | 2.43 | 0.0039 | LOC_Os01g16530 | Clp protease family protein, expressed |
| Os.53103.1.S1_at | 2.21 | 0.0449 | LOC_Os05g32970 | expressed protein |
| Os.53108.1.S1_at | 3.75 | 0.0094 | LOC_Os02g58160 | expressed protein |
| Os.5311.1.S1_at | 3.71 | 0.0154 | LOC_Os08g39630 | Helix-loop-helix DNA-binding domain containing protein |
| Os.53135.1.S1_at | 4.06 | 0.0065 | LOC_Os06g05390 | expressed protein |
| Os.53148.1.S1_at | 2.85 | 0.0016 | LOC_Os03g52720 | magnesium-dependent phosphatase-1 family protein |
| Os.53196.1.S1_at | 2.65 | 0.0012 | LOC_Os12g10510 | expressed protein |
| Os.53213.1.S1_x_at | 4.38 | 0.0072 | LOC_Os11g34130 | expressed protein |
| Os.53265.1.S1_at | 2.08 | 0.0088 | LOC_Os01g33490 | F-box domain containing protein, expressed |
| Os.53291.1.S1_at | 2.62 | 0.0028 | LOC_Os03g64280 | oxidoreductase, 2OG-Fe oxygenase family protein, expressed |
| Os.53308.1.A1_at | 2.34 | 0.0011 | LOC_Os06g13170 | methyltransferase GidB family protein |
| Os.53309.1.S1_at | 1.84 | 0.0363 | LOC_Os07g47650 | Kelch motif family protein, expressed |
| Os.5331.1.S1_at | 1.91 | 0.0043 | LOC_Os05g33510 | expressed protein |
| Os.53316.1.S1_at | 2.09 | 0.0350 | LOC_Os12g40540 | expressed protein |
| Os.5332.1.S1_at | 4.25 | 0.0056 | LOC_Os11g04500 | Cytochrome C biogenesis protein |
| Os.53335.1.S1_at | 5.14 | 0.0217 | LOC_Os11g34110 | expressed protein |
| Os.5335.1.S1_at | 5.20 | 0.0310 | LOC_Os04g49450 | myb-like DNA-binding domain, SHAQKYF class family protein, |
| Os.53358.1.S1_at | 2.18 | 0.0034 | LOC_Os11g37560 | expressed protein |
| Os.534.2.S1_x_at | 4.00 | 0.0014 | LOC_Os01g05800 | Inner membrane protein ALBINO3, chloroplast precursor |
| Os.5342.2.S1_x_at | 1.66 | 0.0420 | LOC_Os01g24880 | VIP2 protein, putative, expressed |
| Os.53427.1.S1_at | 17.65 | 0.0178 | LOC_Os12g08220 | Histone deacetylase family protein, expressed |
| Os.53475.1.A1_at | 4.29 | 0.0132 | LOC_Os12g07270 | Sodium Bile acid symporter family protein, expressed |
| Os.5351.2.S1_s_at | 4.18 | 0.0418 | LOC_Os03g61890 | AIG1 family protein, expressed |
| Os.53543.1.A1_at | 1.89 | 0.0113 | LOC_Os05g34480 | expressed protein |
| Os.5357.1.S1_at | 6.44 | 0.0001 | LOC_Os04g48860 | expressed protein |
| Os.53590.1.S1_at | 1.59 | 0.0045 | LOC_Os05g07730 | hypothetical protein |
| Os.5363.1.S1_at | 6.00 | 0.0492 | LOC_Os07g05040 | cadmium-induced protein, putative, expressed |
| Os.5364.1.S1_at | 2.54 | 0.0310 | LOC_Os03g58160 | HSF-type DNA-binding domain containing protein, expressed |
| Os.5370.1.S1_at | 5.51 | 0.0012 | LOC_Os09g07570 | Ferredoxin-thioredoxin reductase catalytic chain, |
| Os.53702.1.S1_x_at | 3.32 | 0.0006 | LOC_Os01g40310 | expressed protein |
| Os.53716.1.S1_at | 2.40 | 0.0420 | LOC_Os03g11230 | expressed protein |
| Os.53732.1.S1_a_at | 1.61 | 0.0092 | LOC_Os11g06170 | Transcription factor RF2a, putative, expressed |
| Os.53755.1.S1_at | 4.99 | 0.0143 | LOC_Os07g09370 | expressed protein |
| Os.53764.1.S1_at | 3.17 | 0.0070 | LOC_Os03g20180 | YT521-B-like family protein, expressed |
| Os.5377.1.S1_at | 3.87 | 0.0081 | LOC_Os02g46980 | pentatricopeptide, putative, expressed |
| Os.5377.2.S1_x_at | 2.87 | 0.0052 | LOC_Os02g46980 | pentatricopeptide, putative, expressed |
| Os.53776.1.S1_at | 3.56 | 0.0099 | LOC_Os06g50860 | expressed protein |
| Os.53778.1.A1_s_at | 1.70 | 0.0383 | LOC_Os12g29990 | Glycoprotease family protein |
| Os.5378.1.S1_at | 1.70 | 0.0165 | LOC_Os01g56680 | Photosystem II reaction centre W protein |
| Os.53782.1.S1_at | 2.52 | 0.0360 | LOC_Os03g44484 | unspliced-genomic DNA-directed RNA polymerase II subunit RPB2, |
| Os.53789.1.S1_at | 4.46 | 0.0353 | LOC_Os03g21780 | expressed protein |
| Os.53808.1.S1_x_at | 4.72 | 0.0093 | LOC_Os02g35090 | expressed protein |
| Os.53870.1.A1_at | 1.54 | 0.0462 | LOC_Os11g16580 | Endonuclease III-like protein 1, putative, expressed |
| Os.5388.1.S1_at | 2.22 | 0.0438 | LOC_Os02g42940 | VAP27, putative, expressed |
| Os.53894.1.S1_x_at | 1.71 | 0.0109 | LOC_Os04g52120 | Potassium transporter 7, putative, expressed |
| Os.5399.1.S1_at | 1.87 | 0.0208 | LOC_Os06g50310 | bZIP transcription factor family protein, expressed |
| Os.54006.1.S1_at | 1.50 | 0.0378 | LOC_Os04g27950 | expressed protein |
| Os.5401.1.S1_at | 3.65 | 0.0021 | LOC_Os02g57670 | 50S ribosomal protein L9, chloroplast precursor, putative, |
| Os.54060.1.S1_at | 2.93 | 0.0064 | LOC_Os02g07250 | expressed protein |
| Os.54064.1.S1_at | 2.69 | 0.0268 | Os03g0570800 | Multi antimicrobial extrusion protein MatE family protein. |
| Os.54097.1.A1_at | 3.36 | 0.0124 | LOC_Os02g15270 | Tyrosine-protein phosphatase YVH1, putative, expressed |
| Os.54106.1.S1_at | 1.84 | 0.0427 | LOC_Os04g35180 | co-chaperone GrpE family protein, expressed |
| Os.5412.1.S1_at | 6.19 | 0.0145 | LOC_Os03g01014 | unspliced-genomic expressed protein |
| Os.54151.1.S1_x_at | 3.19 | 0.0259 | LOC_Os12g22540 | retrotransposon protein, putative |
| Os.5417.3.S1_x_at | 1.53 | 0.0496 | LOC_Os01g46710 | transposon protein, putative, CACTA, En/Spm sub-class |
| Os.54198.1.S1_at | 5.26 | 0.0018 | LOC_Os06g23560 | UDP-glucoronosyl and UDP-glucosyl transferase family protein |
| Os.5424.1.S1_at | 2.54 | 0.0121 | LOC_Os02g57390 | Pumilio/Mpt5 family RNA-binding protein, putative, expressed |
| Os.5433.1.S1_at | 1.86 | 0.0019 | LOC_Os02g55140 | Cytosol aminopeptidase, putative, expressed |
| Os.54379.1.S1_at | 1.52 | 0.0177 | LOC_Os09g04990 | Cytoskeletal protein, putative, expressed |
| Os.5438.1.S1_at | 37.96 | 0.0265 | LOC_Os10g14870 | heavy metal-associated domain containing protein, expressed |
| Os.5439.1.S1_a_at | 2.44 | 0.0077 | LOC_Os01g18320 | Protoporphyrinogen oxidase, chloroplast precursor, putative |
| Os.54411.1.A1_at | 2.85 | 0.0061 | LOC_Os08g29780 | expressed protein |
| Os.5442.1.S1_at | 5.18 | 0.0017 | Os09g0413200 | Abortive infection protein family protein. |
| Os.54425.1.S1_at | 19.26 | 0.0257 | LOC_Os05g46000 | Ras-related protein Rab7, putative, expressed |
| Os.54427.1.A1_at | 2.57 | 0.0285 | LOC_Os01g46080 | GDSL-like Lipase/Acylhydrolase family protein, expressed |
| Os.54437.1.S1_at | 2.81 | 0.0125 | LOC_Os09g36120 | expressed protein |
| Os.5449.1.S1_at | 5.62 | 0.0009 | Os06g0137600 | Ribosome-binding factor A family protein. |
| Os.54496.1.S1_x_at | 5.20 | 0.0172 | Os05g0298900 | Hypothetical protein. |
| Os.54564.1.S1_at | 3.00 | 0.0236 | LOC_Os03g12930 | expressed protein |
| Os.54568.1.S1_at | 5.01 | 0.0184 | LOC_Os10g36530 | MutS domain V family protein, expressed |
| Os.54601.1.S1_s_at | 1.88 | 0.0301 | LOC_Os10g42650 | transposon protein, putative, CACTA, En/Spm sub-class |
| Os.54619.1.S1_at | 1.94 | 0.0235 | LOC_Os01g03050 | Oxidoreductase NAD-binding domain containing protein |
| Os.54673.1.S1_at | 2.03 | 0.0205 | LOC_Os10g079 | 94 autophagy protein 9, putative, expressed |
| Os.54675.1.S1_at | 12.02 | 0.0381 | LOC_Os05g02250 | expressed protein |
| Os.54675.1.S1_x_at | 13.04 | 0.0414 | LOC_Os05g02250 | expressed protein |
| Os.54722.1.S1_at | 3.67 | 0.0175 | LOC_Os07g33710 | expressed protein |
| Os.54727.1.S1_s_at | 3.20 | 0.0453 | LOC_Os04g57850 | AMP-binding enzyme family protein, expressed |
| Os.54742.1.S1_at | 2.03 | 0.0078 | LOC_Os06g49870 | Dihydrouridine synthase family protein, expressed |
| Os.54773.1.S1_at | 2.27 | 0.0266 | Os11g0473200 | Ubiquitin carboxyl-terminal hydrolase 12 |
| Os.54799.1.A1_at | 2.02 | 0.0344 | LOC_Os09g38730 | 26S proteasome subunit P45 family protein |
| Os.5486.1.S1_at | 2.20 | 0.0006 | LOC_Os08g16050 | Tetraspanin family protein, expressed |
| Os.54895.1.S1_at | 4.99 | 0.0153 | LOC_Os02g09240 | Cytochrome P450 family protein, expressed |
| Os.54895.1.S1_x_at | 3.71 | 0.0336 | LOC_Os02g09240 | Cytochrome P450 family protein, expressed |
| Os.54897.1.S1_at | 3.65 | 0.0004 | LOC_Os02g48370 | ARID/BRIGHT DNA-binding domain-containing protein |
| Os.54905.1.S1_at | 2.08 | 0.0085 | Os03g0703600 | Conserved hypothetical protein. |
| Os.55007.1.S1_at | 3.27 | 0.0045 | LOC_Os07g45330 | expressed protein |
| Os.55007.1.S1_x_at | 3.45 | 0.0101 | LOC_Os07g45330 | expressed protein |
| Os.55033.1.A1_at | 6.92 | 0.0122 | LOC_Os03g37470 | MATE efflux family protein, expressed |
| Os.5518.1.S1_at | 3.81 | 0.0021 | LOC_Os12g38640 | expressed protein |
| Os.55208.1.S1_at | 3.46 | 0.0166 | LOC_Os11g04860 | Indole-3-acetate beta-glucosyltransferase, putative |
| Os.5527.1.S1_a_at | 1.74 | 0.0029 | LOC_Os06g23290 | phosphatidylinositol 3- and 4-kinase family protein, |
| Os.55374.1.S1_s_at | 7.19 | 0.0006 | LOC_Os02g30320 | expressed protein |
| Os.5562.1.S1_at | 4.71 | 0.0004 | LOC_Os04g51150 | transposon protein, putative, unclassified, expressed |
| Os.5567.1.S1_at | 2.79 | 0.0345 | Os03g0161000 | Hypothetical protein. |
| Os.5571.1.S1_at | 2.46 | 0.0273 | LOC_Os02g01240 | Ankyrin-1, putative, expressed |
| Os.5577.2.S1_a_at | 3.97 | 0.0435 | LOC_Os04g59570 | expressed protein |
| Os.5577.2.S1_at | 2.68 | 0.0094 | LOC_Os04g59570 | expressed protein |
| Os.5581.1.S1_at | 4.26 | 0.0001 | LOC_Os03g56540 | DNAJ protein, putative, expressed |
| Os.55991.1.S1_at | 1.52 | 0.0225 | LOC_Os10g39090 | expressed protein |
| Os.5613.1.S1_s_at | 6.24 | 0.0362 | LOC_Os08g14440 | ACT domain-containing protein, putative, expressed |
| Os.56167.1.S1_at | 9.21 | 0.0291 | LOC_Os03g05800 | expressed protein |
| Os.5619.1.A1_at | 2.50 | 0.0169 | LOC_Os12g101 | 84 expressed protein |
| Os.5619.1.A1_s_at | 2.10 | 0.0060 | LOC_Os12g10190 | transposon protein, putative, unclassified, expressed |
| Os.56238.1.S1_at | 2.69 | 0.0049 | LOC_Os11g16560 | expressed protein |
| Os.5631.1.S1_s_at | 2.33 | 0.0005 | LOC_Os04g52900 | ABC transporter, putative, expressed |
| Os.5632.1.S1_a_at | 3.82 | 0.0337 | LOC_Os05g35470 | Dienelactone hydrolase family protein, expressed |
| Os.56323.1.S1_at | 3.08 | 0.0008 | LOC_Os02g56970 | glutathione S-transferase C-terminal domain-containing protein |
| Os.5643.1.S1_at | 6.38 | 0.0051 | LOC_Os01g64890 | CorA-like Mg2+ transporter protein, expressed |
| Os.5651.1.S1_a_at | 3.34 | 0.0111 | LOC_Os03g56310 | HAD superfamily hydrolase, 5'-Nucleotidase |
| Os.5656.1.S1_at | 1.73 | 0.0325 | LOC_Os02g36520 | Kelch motif family protein, expressed |
| Os.5694.1.S1_at | 3.20 | 0.0041 | LOC_Os03g17070 | ATP synthase B chain, chloroplast precursor, putative |
| Os.5697.1.S1_at | 2.64 | 0.0312 | LOC_Os07g33270 | expressed protein |
| Os.57028.1.S1_at | 2.93 | 0.0006 | LOC_Os04g57380 | Phospholipase/Carboxylesterase family protein, expressed |
| Os.57062.1.S1_at | 12.25 | 0.0126 | LOC_Os11g35390 | Myb-like DNA-binding domain containing protein, expressed |
| Os.57099.1.S1_at | 3.23 | 0.0312 | LOC_Os09g36130 | expressed protein |
| Os.57181.1.S1_at | 2.13 | 0.0364 | LOC_Os02g53510 | expressed protein |
| Os.5729.1.S1_at | 1.96 | 0.0104 | LOC_Os08g03570 | UDP-glucose 4-epimerase family protein, expressed |
| Os.57294.1.S1_at | 2.52 | 0.0058 | Os07g0265100 | Hypothetical protein. |
| Os.57337.1.S1_at | 8.59 | 0.0155 | LOC_Os01g45750 | unspliced-genomic bile acid sodium symporter family protein |
| Os.5736.2.S1_x_at | 4.69 | 0.0310 | LOC_Os10g26620 | Dof domain, zinc finger family protein, expressed |
| Os.5739.1.S1_at | 3.34 | 0.0124 | LOC_Os05g49300 | NifU-like N-terminal domain containing protein |
| Os.5754.1.S1_at | 6.97 | 0.0024 | LOC_Os08g04450 | DAG protein, chloroplast precursor, putative, expressed |
| Os.57565.1.S1_at | 2.68 | 0.0012 | LOC_Os07g05870 | expressed protein |
| Os.5795.1.S1_at | 1.63 | 0.0179 | LOC_Os08g01850 | hydrolase, alpha/beta fold family protein, expressed |
| Os.5845.1.S1_at | 1.54 | 0.0149 | LOC_Os01g72960 | expressed protein |
| Os.5869.1.S1_a_at | 1.52 | 0.0244 | LOC_Os04g38410 | Chlorophyll a-b binding protein CP24 10B |
| Os.5873.1.S1_at | 6.27 | 0.0121 | LOC_Os04g51400 | Zinc finger, C3HC4 type family protein, expressed |
| Os.5874.1.S1_at | 2.34 | 0.0406 | LOC_Os06g34450 | Zinc finger, C3HC4 type family protein, expressed |
| Os.5878.1.S1_at | 2.13 | 0.0099 | LOC_Os08g21350 | cig3, putative, expressed |
| Os.5884.1.S1_at | 1.63 | 0.0086 | LOC_Os05g34650 | expressed protein |
| Os.5888.1.S1_at | 1.75 | 0.0159 | LOC_Os04g41830 | Myb-like DNA-binding domain containing protein, expressed |
| Os.5960.1.S1_at | 3.76 | 0.0087 | LOC_Os04g37580 | ChaC-like protein, expressed |
| Os.5961.1.S1_at | 2.42 | 0.0092 | LOC_Os07g38360 | expressed protein |
| Os.5986.1.S1_at | 3.91 | 0.0086 | LOC_Os08g44400 | Glutathione S-transferase, N-terminal domain containing protein |
| Os.5992.1.S1_at | 2.50 | 0.0156 | LOC_Os08g19650 | Homeobox protein knotted-1-like 3, putative, expressed |
| Os.6046.1.S2_at | 4.17 | 0.0085 | LOC_Os06g42060 | expressed protein |
| Os.6073.1.S1_at | 3.98 | 0.0267 | LOC_Os03g38590 | Secretory carrier-associated membrane protein |
| Os.6086.2.S1_x_at | 1.86 | 0.0484 | LOC_Os05g48820 | DDT domain containing protein, expressed |
| Os.609.1.S2_a_at | 1.91 | 0.0180 | LOC_Os06g04270 | Transketolase, chloroplast precursor, putative, expressed |
| Os.6126.1.S1_at | 2.22 | 0.0374 | LOC_Os04g41160 | Protein kinase domain containing protein, expressed |
| Os.6165.1.S1_a_at | 4.76 | 0.0202 | LOC_Os09g30120 | cellulose synthase-like protein CslE, putative, expressed |
| Os.6172.1.S1_s_at | 7.69 | 0.0277 | Os12g0290200 | Hypothetical protein. |
| Os.6207.1.S1_s_at | 4.69 | 0.0484 | LOC_Os06g41840 | NAD dependent epimerase/dehydratase family protein |
| Os.6220.1.S1_at | 2.09 | 0.0028 | LOC_Os09g15430 | zinc finger family protein, putative, expressed |
| Os.6225.2.S1_at | 4.01 | 0.0285 | LOC_Os06g30930 | expressed protein |
| Os.6230.1.S1_at | 3.41 | 0.0009 | LOC_Os03g64350 | Rhodanese-like domain containing protein, expressed |
| Os.6257.1.S1_at | 2.01 | 0.0047 | LOC_Os06g08490 | expressed protein |
| Os.6264.1.S1_at | 1.96 | 0.0367 | LOC_Os02g48810 | PHD-finger family protein, expressed |
| Os.6265.1.S1_at | 2.04 | 0.0079 | LOC_Os12g34850 | Fibronectin type III domain containing protein, expressed |
| Os.6294.1.S1_s_at | 4.81 | 0.0071 | LOC_Os01g55880 | hemimethylated DNA binding domain containing protein |
| Os.6334.1.S1_at | 3.44 | 0.0010 | Os09g0361500 | Isochorismate synthase 1, chloroplast precursor |
| Os.6336.1.S1_at | 2.36 | 0.0342 | LOC_Os01g70960 | Cytochrome c1, heme protein, mitochondrial precursor, puta |
| Os.6340.1.S1_at | 8.35 | 0.0229 | Os05g0441500 | Non-protein coding transcript, uncharacterized transcript. |
| Os.6340.1.S2_s_at | 4.54 | 0.0003 | Os05g0441500 | Non-protein coding transcript, uncharacterized transcript. |
| Os.6369.1.S1_at | 2.88 | 0.0285 | LOC_Os12g03400 | expressed protein |
| Os.6371.1.S1_at | 2.61 | 0.0021 | LOC_Os01g67000 | membrane-associated 30 kDa protein, chloroplast precursor |
| Os.6375.1.S1_s_at | 6.54 | 0.0289 | LOC_Os01g63990 | hydrolase, alpha/beta fold family protein, expressed |
| Os.6379.1.S1_at | 6.67 | 0.0083 | LOC_Os12g44350 | Actin-1, putative, expressed |
| Os.6384.1.S1_at | 3.48 | 0.0312 | LOC_Os12g13460 | SET domain containing protein, expressed |
| Os.6448.1.S1_at | 9.11 | 0.0013 | LOC_Os03g02590 | Peroxisomal membrane protein PEX11-1, putative, expressed |
| Os.6452.2.A1_a_at | 4.42 | 0.0066 | LOC_Os01g43710 | Cytochrome P450 72A1, putative, expressed |
| Os.6566.1.S1_at | 2.45 | 0.0262 | LOC_Os02g37090 | hydrolase, alpha/beta fold family protein, expressed |
| Os.6590.1.S1_at | 1.81 | 0.0121 | LOC_Os03g22370 | Photosystem II reaction centre X protein containing protein |
| Os.6595.1.S1_a_at | 3.93 | 0.0211 | LOC_Os07g46310 | CobN/magnesium chelatase family protein |
| Os.6824.1.S1_at | 3.07 | 0.0076 | LOC_Os08g31410 | Sulfate transporter 1.2, putative, expressed |
| Os.6830.1.S1_at | 3.62 | 0.0081 | LOC_Os02g09590 | ribosomal protein S21 containing protein, expressed |
| Os.6831.1.S1_a_at | 2.48 | 0.0356 | LOC_Os08g44290 | 28 kDa ribonucleoprotein, chloroplast, putative, expressed |
| Os.6868.1.S1_at | 1.93 | 0.0172 | LOC_Os03g43420 | single-strand binding protein, expressed |
| Os.6898.1.S1_at | 5.18 | 0.0249 | LOC_Os07g43570 | Protein kinase domain containing protein, expressed |
| Os.6998.1.S1_at | 6.51 | 0.0230 | LOC_Os03g37490 | MATE efflux family protein, expressed |
| Os.7011.1.S1_at | 2.30 | 0.0162 | LOC_Os05g49650 | expressed protein |
| Os.7020.1.S1_x_at | 3.48 | 0.0127 | LOC_Os05g40260 | Ycf52, putative, expressed |
| Os.7029.1.S1_at | 4.48 | 0.0048 | LOC_Os05g24650 | expressed protein |
| Os.7057.1.S1_at | 1.82 | 0.0036 | Os09g0131000 | Conserved hypothetical protein. |
| Os.7132.1.S1_at | 5.48 | 0.0056 | LOC_Os09g39560 | Genetic modifier, putative, expressed |
| Os.7137.1.S1_at | 9.34 | 0.0440 | LOC_Os01g66980 | expressed protein |
| Os.7140.1.S1_at | 4.44 | 0.0046 | LOC_Os11g16550 | GUN4-like family protein, expressed |
| Os.7165.1.S1_at | 3.52 | 0.0036 | Os01g0707300 | Vesicle transport v-SNARE family protein. |
| Os.7167.1.S1_at | 1.53 | 0.0266 | LOC_Os03g31570 | proline-rich family protein, putative, expressed |
| Os.7170.1.S1_at | 1.71 | 0.0036 | LOC_Os12g18110 | Lysosomal Cystine Transporter family protein, expressed |
| Os.7218.1.S1_at | 4.37 | 0.0013 | LOC_Os02g01150 | D-isomer specific 2-hydroxyacid dehydrogenase, |
| Os.7230.1.S1_at | 2.54 | 0.0012 | LOC_Os07g10490 | Zeta-carotene desaturase, chloroplast precursor, putative, |
| Os.7243.1.S1_s_at | 1.54 | 0.0160 | LOC_Os08g06250 | GMFP4, putative, expressed |
| Os.7282.1.S1_at | 3.34 | 0.0164 | LOC_Os03g12000 | DEAD/DEAH box helicase family protein, putative, expressed |
| Os.7284.1.S1_a_at | 4.49 | 0.0232 | LOC_Os07g46410 | thioredoxin-disulfide reductase family protein, expressed |
| Os.7304.1.S1_at | 3.96 | 0.0470 | LOC_Os01g10450 | protein kinase, putative, expressed |
| Os.7308.1.S1_x_at | 3.20 | 0.0079 | LOC_Os03g31150 | expressed protein |
| Os.7308.1.S2_x_at | 5.33 | 0.0022 | LOC_Os03g31150 | expressed protein |
| Os.7312.1.S1_a_at | 2.87 | 0.0159 | LOC_Os07g49030 | PHD-finger family protein, expressed |
| Os.7317.1.S1_a_at | 1.70 | 0.0296 | LOC_Os02g58340 | Peptidase family M3 containing protein, expressed |
| Os.7346.1.S1_at | 8.88 | 0.0031 | LOC_Os03g58100 | pentatricopeptide, putative, expressed |
| Os.7396.2.S1_a_at | 1.79 | 0.0286 | LOC_Os02g47580 | expressed protein |
| Os.7418.1.S1_a_at | 1.68 | 0.0320 | Os02g0635700 | Conserved hypothetical protein. |
| Os.7443.1.S1_s_at | 10.96 | 0.0092 | LOC_Os03g02020 | expressed protein |
| Os.7451.1.S1_at | 4.94 | 0.0001 | LOC_Os03g55820 | Thioredoxin family protein, expressed |
| Os.7465.1.S1_at | 1.72 | 0.0057 | LOC_Os05g28980 | expressed protein |
| Os.7497.1.S1_at | 1.76 | 0.0422 | LOC_Os02g17870 | Micro-fibrillar-associated protein 1 C-terminus containing |
| Os.7502.1.S1_at | 1.91 | 0.0347 | Os01g0705300 | Conserved hypothetical protein. |
| Os.7504.2.S1_at | 1.61 | 0.0497 | LOC_Os05g43280 | MATH domain containing protein, expressed |
| Os.7525.1.S1_at | 1.88 | 0.0455 | LOC_Os01g73000 | Copine family protein, expressed |
| Os.7527.1.S1_at | 19.39 | 0.0004 | LOC_Os01g01280 | expressed protein |
| Os.7552.1.S1_at | 3.04 | 0.0029 | LOC_Os06g08670 | Phospholipid hydroperoxide glutathione peroxidase, |
| Os.7589.1.S1_at | 2.04 | 0.0025 | LOC_Os06g46930 | 50S ribosomal protein L24, chloroplast precursor, |
| Os.7626.1.S1_at | 3.44 | 0.0210 | LOC_Os03g31300 | Chaperone clpB 1, putative, expressed |
| Os.7662.1.S1_at | 9.43 | 0.0064 | LOC_Os02g10350 | Mlo family protein, expressed |
| Os.7676.1.S1_at | 2.91 | 0.0247 | LOC_Os02g49680 | calcium sensing receptor, putative, expressed |
| Os.7679.1.S1_at | 4.82 | 0.0186 | LOC_Os07g23990 | tetratricopeptide repeat, putative, expressed |
| Os.7691.1.S1_s_at | 3.89 | 0.0035 | LOC_Os02g33010 | expressed protein |
| Os.7695.1.S1_at | 13.32 | 0.0314 | LOC_Os04g11390 | expressed protein |
| Os.7707.1.S1_at | 4.19 | 0.0133 | LOC_Os04g54320 | expressed protein |
| Os.7712.1.S1_a_at | 6.04 | 0.0027 | LOC_Os01g48990 | Kinase C630.09c, putative, expressed |
| Os.7718.1.S1_at | 4.07 | 0.0145 | LOC_Os05g27930 | AP2 domain containing protein, expressed |
| Os.773.1.S1_s_at | 5.47 | 0.0443 | LOC_Os01g43750 | Cytochrome P450 family protein, expressed |
| Os.7747.1.S1_at | 2.81 | 0.0109 | LOC_Os01g62300 | SLT1 protein, putative, expressed |
| Os.7752.1.S1_at | 3.20 | 0.0033 | LOC_Os03g41080 | seed maturation protein PM23, putative, expressed |
| Os.7754.1.S1_at | 2.73 | 0.0128 | LOC_Os08g44640 | Serine carboxypeptidase family protein, expressed |
| Os.7759.1.S1_at | 2.84 | 0.0174 | LOC_Os08g37790 | Phospho-2-dehydro-3-deoxyheptonate aldolase 2, |
| Os.7760.1.S1_at | 3.66 | 0.0326 | LOC_Os01g51370 | expressed protein |
| Os.7816.1.S1_at | 1.72 | 0.0406 | LOC_Os11g31640 | Serine palmitoyltransferase 2, putative, expressed |
| Os.7826.2.A1_at | 6.51 | 0.0015 | LOC_Os05g10730 | multidrug resistance associated protein 1 |
| Os.7859.1.S1_at | 2.86 | 0.0027 | Os11g0181500 | Nitrogen fixation like protein. |
| Os.7860.1.S1_at | 2.56 | 0.0103 | LOC_Os09g27030 | Cysteine proteinase 2 precursor, putative, expressed |
| Os.7864.1.S1_at | 1.99 | 0.0411 | LOC_Os03g60100 | 50S ribosomal protein L17, putative, expressed |
| Os.7865.1.S1_at | 1.95 | 0.0267 | LOC_Os06g15400 | expressed protein |
| Os.7866.1.S1_at | 4.55 | 0.0008 | LOC_Os10g42240 | carbohydrate kinase, putative, expressed |
| Os.7868.1.S1_at | 11.72 | 0.0174 | LOC_Os01g17170 | Magnesium-protoporphyrin IX monomethyl ester cyclase, |
| Os.7872.1.S1_at | 5.28 | 0.0085 | LOC_Os01g19740 | chloroplast protein 12, putative, expressed |
| Os.7873.1.S1_at | 2.26 | 0.0432 | LOC_Os07g05820 | expressed protein |
| Os.7873.1.S1_x_at | 2.34 | 0.0380 | LOC_Os07g05820 | expressed protein |
| Os.7890.1.S1_x_at | 1.56 | 0.0342 | LOC_Os08g33820 | Chlorophyll a-b binding protein 4, chloroplast precursor, |
| Os.7896.2.S1_at | 1.78 | 0.0453 | LOC_Os12g31440 | expressed protein |
| Os.7907.1.S2_at | 9.38 | 0.0018 | LOC_Os07g46460 | Ferredoxin-dependent glutamate synthase |
| Os.7914.2.S1_at | 3.68 | 0.0017 | LOC_Os05g09400 | expressed protein |
| Os.7915.1.S1_a_at | 1.53 | 0.0074 | LOC_Os06g01210 | Plastocyanin, chloroplast precursor, putative, expressed |
| Os.7931.1.S1_a_at | 5.93 | 0.0102 | LOC_Os06g51290 | Phytoene synthase, chloroplast precursor, putative |
| Os.7931.1.S1_s_at | 7.11 | 0.0002 | LOC_Os06g51290 | Phytoene synthase, chloroplast precursor, putative |
| Os.7933.1.S1_at | 2.90 | 0.0016 | LOC_Os05g25840 | ABC1 family protein, expressed |
| Os.7934.1.S1_at | 3.01 | 0.0263 | LOC_Os04g58730 | AT hook motif family protein, expressed |
| Os.7948.1.S1_a_at | 3.41 | 0.0055 | LOC_Os01g17150 | expressed protein |
| Os.7961.1.S1_s_at | 1.84 | 0.0278 | LOC_Os07g41800 | Peroxisome assembly protein 10, putative, expressed |
| Os.7962.1.S1_a_at | 2.07 | 0.0191 | LOC_Os08g10020 | Photosystem II 10 kDa polypeptide, chloroplast precursor, |
| Os.7969.1.S1_x_at | 3.39 | 0.0317 | LOC_Os08g01270 | Protein kinase domain containing protein, expressed |
| Os.7969.2.S1_at | 3.98 | 0.0264 | Os08g0103000 | Serine/thronine protein kinase-like protein. |
| Os.7971.1.S1_x_at | 2.02 | 0.0454 | LOC_Os01g47330 | 50S ribosomal protein L12-2, chloroplast precursor, |
| Os.7972.1.S2_at | 1.83 | 0.0500 | LOC_Os02g39790 | S-adenosylmethionine decarboxylase proenzyme, putative, |
| Os.7974.1.S1_at | 1.79 | 0.0360 | LOC_Os08g44680 | Photosystem I reaction center subunit II, |
| Os.7977.1.S1_at | 3.33 | 0.0201 | LOC_Os12g14070 | Stromal 70 kDa heat shock-related protein |
| Os.7986.1.S1_at | 4.16 | 0.0225 | LOC_Os03g03910 | Catalase-1, putative, expressed |
| Os.7988.1.S1_s_at | 2.84 | 0.0058 | LOC_Os11g07020 | Fructose-bisphosphate aldolase, chloroplast precursor |
| Os.7994.1.S1_at | 3.48 | 0.0070 | LOC_Os04g45490 | Elongation factor G, chloroplast precursor |
| Os.7999.1.S1_at | 1.76 | 0.0052 | LOC_Os07g43380 | zinc finger family protein, putative, expressed |
| Os.8003.1.S1_at | 2.14 | 0.0280 | LOC_Os10g41400 | Peptide methionine sulfoxide reductase |
| Os.8012.1.S1_at | 15.37 | 0.0335 | LOC_Os08g01140 | Cytochrome b561 family protein, expressed |
| Os.8037.1.S2_at | 2.11 | 0.0337 | unknown | unknown |
| Os.8081.1.A1_at | 3.72 | 0.0125 | LOC_Os07g36390 | CRP1, putative, expressed |
| Os.8103.1.S1_at | 2.00 | 0.0003 | LOC_Os06g19680 | RING finger family protein, putative, expressed |
| Os.8112.1.S1_at | 16.65 | 0.0259 | LOC_Os07g04990 | oxidoreductase, aldo/keto reductase family protein |
| Os.8117.2.S1_at | 4.70 | 0.0032 | Os04g0442300 | ZmRR2 protein (Response regulator 2). |
| Os.8133.1.S1_at | 5.15 | 0.0006 | LOC_Os02g32730 | alkaline/neutral invertase, putative, expressed |
| Os.8136.1.A1_at | 1.82 | 0.0232 | LOC_Os06g40030 | D-mannose binding lectin family protein, expressed |
| Os.8138.1.S1_at | 4.80 | 0.0152 | LOC_Os05g39230 | expressed protein |
| Os.8149.1.S1_at | 4.99 | 0.0140 | LOC_Os02g46030 | myb-like DNA-binding domain, SHAQKYF class family protein, |
| Os.8150.1.S1_at | 1.54 | 0.0361 | LOC_Os05g42350 | Sulfite reductase, putative, expressed |
| Os.8168.1.S1_at | 1.93 | 0.0050 | LOC_Os09g25320 | polyubiquitin 2, putative, expressed |
| Os.8200.1.S1_s_at | 1.85 | 0.0047 | LOC_Os03g63770 | Poly polymerase catalytic domain containing protein |
| Os.8213.1.S1_at | 3.36 | 0.0119 | LOC_Os11g23790 | expressed protein |
| Os.8323.1.S1_a_at | 1.53 | 0.0416 | LOC_Os02g07790 | Protein kinase domain containing protein, expressed |
| Os.834.1.S1_a_at | 2.25 | 0.0255 | LOC_Os01g43870 | NLI interacting factor-like phosphatase family protein |
| Os.8347.1.S1_at | 1.99 | 0.0119 | LOC_Os09g35680 | F-box domain containing protein, expressed |
| Os.8353.1.S1_at | 2.20 | 0.0115 | LOC_Os01g68770 | Selenium-binding protein, putative, expressed |
| Os.8381.1.S1_at | 2.05 | 0.0186 | LOC_Os05g06270 | VIP2 protein, putative, expressed |
| Os.8399.1.S1_at | 5.62 | 0.0024 | LOC_Os12g18640 | pentatricopeptide, putative, expressed |
| Os.8406.1.S1_at | 2.48 | 0.0019 | LOC_Os02g30230 | Exo70 exocyst complex subunit family protein, expressed |
| Os.8444.1.S1_s_at | 2.61 | 0.0377 | LOC_Os04g57700 | expressed protein |
| Os.8453.1.S1_a_at | 1.78 | 0.0213 | LOC_Os06g09890 | Smr domain containing protein, expressed |
| Os.8455.1.S1_at | 1.93 | 0.0324 | LOC_Os07g28610 | expressed protein |
| Os.8457.1.S1_at | 3.37 | 0.0260 | LOC_Os05g27100 | heavy metal-associated domain containing protein |
| Os.8465.1.S1_a_at | 5.26 | 0.0006 | LOC_Os12g37530 | flagellar biosynthesis protein FLHA, putative, expressed |
| Os.8468.1.S1_s_at | 11.35 | 0.0000 | LOC_Os12g25180 | expressed protein |
| Os.8476.1.S1_at | 1.84 | 0.0186 | LOC_Os03g19510 | ATP-dependent Clp protease proteolytic subunit 1 |
| Os.8479.1.S1_s_at | 4.32 | 0.0173 | LOC_Os04g34460 | plastid-lipid associated protein PAP, putative, expressed |
| Os.8495.1.S1_at | 2.05 | 0.0089 | LOC_Os02g08350 | expressed protein |
| Os.8504.1.S1_at | 2.08 | 0.0023 | LOC_Os04g42420 | nodulin, putative, expressed |
| Os.8507.1.S1_at | 5.44 | 0.0460 | LOC_Os09g35800 | UDP-glucose 4-epimerase, putative, expressed |
| Os.8520.1.S1_at | 2.35 | 0.0126 | LOC_Os06g22690 | bundle sheath defective protein 2, putative, expressed |
| Os.8521.2.S1_x_at | 7.28 | 0.0320 | LOC_Os07g11110 | mRNA-binding protein precursor, putative, expressed |
| Os.8549.1.S1_at | 2.80 | 0.0005 | LOC_Os03g50130 | MAPEG family protein, expressed |
| Os.8553.1.S1_at | 2.24 | 0.0281 | LOC_Os03g52130 | expressed protein |
| Os.8554.1.S1_at | 3.20 | 0.0000 | LOC_Os01g74190 | expressed protein |
| Os.8554.2.S1_x_at | 5.93 | 0.0006 | LOC_Os01g74190 | expressed protein |
| Os.8571.1.S1_s_at | 3.47 | 0.0025 | LOC_Os01g40310 | expressed protein |
| Os.8611.1.S1_at | 3.88 | 0.0097 | LOC_Os05g29760 | Ferrochelatase II, chloroplast precursor, putative |
| Os.8686.1.S1_at | 5.29 | 0.0014 | LOC_Os03g57840 | Sterol-regulatory element binding protein site 2 protease |
| Os.8708.1.S1_at | 1.75 | 0.0086 | LOC_Os11g41160 | Phosphoserine phosphatase, putative, expressed |
| Os.8712.1.S2_at | 3.50 | 0.0077 | LOC_Os08g33320 | expressed protein |
| Os.872.2.S1_at | 24.13 | 0.0275 | LOC_Os01g14590 | Pathogen-related protein, putative, expressed |
| Os.8741.1.S1_at | 5.47 | 0.0099 | LOC_Os03g12820 | Poly polymerase catalytic domain containing protein |
| Os.8756.2.A1_at | 1.93 | 0.0159 | LOC_Os07g46380 | glycosyltransferase, putative, expressed |
| Os.8758.1.S1_s_at | 2.51 | 0.0094 | LOC_Os01g47540 | CTD-phosphatase, putative, expressed |
| Os.8773.1.S1_a_at | 1.52 | 0.0020 | Os06g0196900 | 20 kDa chaperonin, chloroplast precursor |
| Os.8776.1.S1_at | 1.86 | 0.0344 | Os03g0859800 | Ovarian tumour, otubain domain containing protein. |
| Os.8785.1.S1_at | 9.81 | 0.0161 | LOC_Os03g40100 | ACT domain containing protein, putative, expressed |
| Os.8788.1.S1_at | 1.85 | 0.0257 | LOC_Os10g32730 | HAD-superfamily hydrolase, subfamily IA |
| Os.8858.1.S1_a_at | 2.37 | 0.0100 | LOC_Os02g50850 | BAH domain containing protein, expressed |
| Os.8865.2.S1_x_at | 2.61 | 0.0053 | LOC_Os01g61320 | expressed protein |
| Os.8865.4.S1_x_at | 2.39 | 0.0067 | LOC_Os01g61320 | expressed protein |
| Os.8888.1.S1_at | 10.77 | 0.0016 | unknown | unknown |
| Os.8900.1.S1_a_at | 4.72 | 0.0052 | Os03g0157400 | ABC transporter related domain containing protein. |
| Os.8907.1.S1_at | 2.33 | 0.0196 | LOC_Os01g58690 | Apurinic endonuclease-redox protein, putative, expressed |
| Os.8913.1.S1_at | 1.94 | 0.0029 | LOC_Os02g03260 | homoaconitate hydratase family protein, expressed |
| Os.8921.1.S1_at | 3.11 | 0.0011 | Os04g0607000 | PAP fibrillin family protein. |
| Os.8936.1.S1_at | 3.02 | 0.0179 | Os08g0170800 | Hypothetical protein. |
| Os.8938.1.S1_at | 2.62 | 0.0083 | LOC_Os09g10760 | Plastid-specific 30S ribosomal protein 2 |
| Os.8946.1.S1_at | 1.52 | 0.0085 | LOC_Os07g05820 | expressed protein |
| Os.8970.1.S1_at | 2.55 | 0.0021 | LOC_Os12g41800 | VTC2, putative, expressed |
| Os.8987.1.S1_at | 2.70 | 0.0312 | LOC_Os08g10400 | expressed protein |
| Os.8994.1.S1_at | 5.87 | 0.0337 | LOC_Os05g41030 | Protease inhibitor/seed storage/LTP family protein, expressed |
| Os.9021.1.S1_at | 3.17 | 0.0012 | LOC_Os03g22430 | ATP-dependent Clp protease proteolytic subunit, putative, |
| Os.9023.1.S1_x_at | 1.57 | 0.0079 | LOC_Os01g04010 | expressed protein |
| Os.9077.1.S1_at | 1.96 | 0.0438 | LOC_Os10g41940 | expressed protein |
| Os.9097.1.A1_at | 3.04 | 0.0122 | LOC_Os09g34280 | ankyrin repeat family protein, putative, expressed |
| Os.9104.1.S1_at | 3.26 | 0.0243 | LOC_Os07g30300 | small G protein family protein, putative, expressed |
| Os.9105.1.S1_at | 5.22 | 0.0423 | LOC_Os09g33680 | Glycosyl hydrolase family 1 protein, expressed |
| Os.9116.1.S1_at | 2.30 | 0.0294 | LOC_Os01g28680 | LEC14B homolog, putative, expressed |
| Os.9118.1.S1_at | 2.38 | 0.0177 | LOC_Os05g49060 | expressed protein |
| Os.9125.1.S1_at | 2.05 | 0.0255 | LOC_Os03g52150 | major surface like glycoprotein, putative, expressed |
| Os.9134.1.S1_at | 2.53 | 0.0018 | LOC_Os03g06740 | Glutathione reductase, chloroplast precursor |
| Os.9142.1.S1_at | 2.10 | 0.0239 | Os05g0587200 | Conserved hypothetical protein. |
| Os.9145.1.S1_at | 4.74 | 0.0016 | LOC_Os05g09430 | expressed protein |
| Os.9166.4.S1_x_at | 3.05 | 0.0391 | LOC_Os10g41210 | expressed protein |
| Os.9205.1.S1_x_at | 1.54 | 0.0081 | LOC_Os03g62340 | Protein kinase domain containing protein, expressed |
| Os.9224.1.S1_at | 2.04 | 0.0100 | LOC_Os05g05950 | chloroplast outer membrane protein, putative, expressed |
| Os.9226.1.S1_a_at | 4.60 | 0.0128 | LOC_Os03g15870 | 50S ribosomal protein L4, chloroplast precursor, putative, |
| Os.9230.1.S1_x_at | 2.77 | 0.0130 | LOC_Os10g35370 | Protochlorophyllide reductase B, chloroplast precursor |
| Os.9235.1.S1_at | 1.90 | 0.0008 | LOC_Os02g10510 | Ubiquitin family protein, expressed |
| Os.9240.1.S1_at | 1.62 | 0.0172 | LOC_Os04g31330 | Exo70 exocyst complex subunit family protein, expressed |
| Os.9242.1.A1_at | 1.85 | 0.0160 | LOC_Os01g62820 | Transcription initiation factor TFIID subunit A family protein |
| Os.9247.2.S1_x_at | 2.55 | 0.0463 | LOC_Os06g07230 | Protein kinase APK1B, chloroplast precursor, putative |
| Os.9262.1.S1_at | 1.91 | 0.0366 | LOC_Os03g21720 | Vacuolar sorting receptor 1 precursor, putative, expressed |
| Os.9272.1.S1_at | 2.22 | 0.0145 | LOC_Os06g21600 | Uncharacterized ACR, YdiU/UPF0061 family protein, expressed |
| Os.9279.1.S1_at | 4.43 | 0.0096 | LOC_Os12g38270 | Metallothionein-like protein 1, putative, expressed |
| Os.9332.1.S1_at | 21.38 | 0.0092 | Os06g0108900 | Homeobox domain containing protein. |
| Os.9354.1.S1_at | 2.29 | 0.0043 | LOC_Os08g44820 | No apical meristem protein, expressed |
| Os.9354.2.S1_at | 4.03 | 0.0212 | Os08g0562200 | No apical meristem (NAM) protein domain containing protein. |
| Os.9367.1.S1_a_at | 3.60 | 0.0013 | LOC_Os09g16910 | Cysteine desulfurase, mitochondrial precursor, putative |
| Os.9440.1.S1_a_at | 5.49 | 0.0009 | LOC_Os06g45820 | Cell division protein ftsH homolog, putative, expressed |
| Os.9448.1.A2_at | 2.67 | 0.0073 | LOC_Os09g38540 | Plastocyanin-like domain containing protein, expressed |
| Os.9466.1.S1_a_at | 4.92 | 0.0340 | LOC_Os12g10730 | Glutathione S-transferase, putative, expressed |
| Os.9476.1.S1_at | 1.57 | 0.0327 | LOC_Os11g34180 | Uncharacterized protein family UPF0016 containing protein, |
| Os.9487.1.S1_a_at | 1.86 | 0.0235 | LOC_Os01g10250 | hydrolase, alpha/beta fold family protein, expressed |
| Os.9506.1.S1_a_at | 4.17 | 0.0171 | LOC_Os02g38020 | Phosphate transporter family protein, expressed |
| Os.9536.1.S1_at | 2.70 | 0.0359 | LOC_Os03g04930 | pre-mRNA processing protein PRP39, putative, expressed |
| Os.9619.1.S1_at | 2.32 | 0.0228 | LOC_Os02g51150 | Protein SUR2, putative, expressed |
| Os.9620.1.S1_at | 2.60 | 0.0009 | LOC_Os03g19410 | prolyl oligopeptidase family protein, expressed |
| Os.9633.1.S1_a_at | 2.08 | 0.0394 | LOC_Os07g38110 | Tic20, putative, expressed |
| Os.9650.1.A1_at | 2.35 | 0.0328 | LOC_Os04g57210 | Actin family protein, expressed |
| Os.9678.1.S1_at | 5.54 | 0.0017 | LOC_Os07g47300 | GTP1/OBG family protein, putative, expressed |
| Os.9688.1.S1_at | 2.22 | 0.0010 | LOC_Os05g23740 | Stromal 70 kDa heat shock-related protein |
| Os.9750.1.S1_at | 2.87 | 0.0025 | LOC_Os02g49320 | sucrase, putative, expressed |
| Os.9752.2.S1_x_at | 4.83 | 0.0185 | Os12g0133100 | Major facilitator superfamily protein. |
| Os.9752.3.S1_x_at | 4.10 | 0.0195 | Os12g0133100 | Major facilitator superfamily protein. |
| Os.9756.1.S1_a_at | 7.94 | 0.0010 | LOC_Os11g05260 | phosphoglycerate mutase family protein, expressed |
| Os.9765.1.S1_a_at | 4.07 | 0.0100 | LOC_Os03g03470 | expressed protein |
| Os.9767.1.S1_at | 4.04 | 0.0464 | LOC_Os03g38950 | expressed protein |
| Os.9768.1.S1_a_at | 2.93 | 0.0135 | LOC_Os02g57410 | OTU-like cysteine protease family protein, expressed |
| Os.9773.1.S1_at | 2.26 | 0.0032 | LOC_Os01g59060 | ribosomal protein L34 containing protein, expressed |
| Os.9779.1.S1_at | 2.72 | 0.0026 | LOC_Os01g32730 | tetratricopeptide repeat, putative, expressed |
| Os.9780.1.S1_at | 4.01 | 0.0017 | LOC_Os07g36250 | Peptide chain release factor 2, putative, expressed |
| Os.9792.1.S1_at | 7.80 | 0.0085 | LOC_Os02g05890 | expressed protein |
| Os.9818.1.S1_at | 4.38 | 0.0065 | LOC_Os02g52260 | expressed protein |
| Os.9834.1.S1_a_at | 1.57 | 0.0255 | LOC_Os11g10070 | expressed protein |
| Os.9880.1.S1_a_at | 2.54 | 0.0146 | LOC_Os01g68480 | Thioredoxin F-type 2, chloroplast precursor, putative |
| Os.9885.2.S1_x_at | 4.87 | 0.0486 | LOC_Os03g61090 | expressed protein |
| Os.9889.1.S1_at | 1.92 | 0.0206 | LOC_Os06g19730 | HEAT repeat family protein, expressed |
| Os.9902.1.S1_at | 1.89 | 0.0059 | LOC_Os01g65900 | GRAS family transcription factor containing protein |
| Os.9908.1.S1_at | 3.49 | 0.0450 | LOC_Os05g39760 | VHS domain containing protein, expressed |
| Os.9948.1.S1_at | 1.90 | 0.0173 | LOC_Os08g38880 | WD-40 repeat family protein, putative, expressed |
| Os.9954.1.S1_at | 1.86 | 0.0307 | LOC_Os03g42840 | Calcineurin B-like protein 3, putative, expressed |
| Os.9996.1.S1_at | 11.77 | 0.0153 | LOC_Os11g10990 | DNAJ heat shock N-terminal domain-containing protein |
| Os.9996.2.S1_at | 2.23 | 0.0055 | LOC_Os11g10980 | pyruvate kinase family protein, expressed |
| OsAffx.11002.1.S1_at | 2.61 | 0.0329 | LOC_Os01g10810 | Rho termination factor, N-terminal domain containing protein |
| OsAffx.11050.1.S1_x_at | 2.30 | 0.0265 | LOC_Os01g14440 | WRKY DNA binding domain containing protein, expressed |
| OsAffx.11057.1.S1_x_at | 1.98 | 0.0420 | LOC_Os01g14890 | hypothetical protein |
| OsAffx.11154.2.S1_s_at | 2.41 | 0.0444 | LOC_Os01g21980 | hypothetical protein |
| OsAffx.11628.1.S1_x_at | 3.46 | 0.0124 | LOC_Os01g56840 | expressed protein |
| OsAffx.11847.1.S1_at | 3.92 | 0.0450 | LOC_Os01g74350 | Soluble inorganic pyrophosphatase, putative, expressed |
| OsAffx.11871.1.S1_s_at | 4.09 | 0.0269 | LOC_Os02g03020 | calcium-binding protein, putative, expressed |
| OsAffx.11906.1.S1_at | 2.18 | 0.0073 | LOC_Os02g05700 | ZEITLUPE, putative, expressed |
| OsAffx.11954.1.S1_s_at | 3.60 | 0.0133 | LOC_Os02g09420 | cyclase family protein, putative, expressed |
| OsAffx.11955.1.S1_at | 3.85 | 0.0235 | LOC_Os02g09440 | expressed protein |
| OsAffx.12071.1.S1_s_at | 4.69 | 0.0226 | LOC_Os02g16630 | N-anthranilate isomerase family protein, expressed |
| OsAffx.12107.1.S1_at | 2.19 | 0.0438 | LOC_Os02g18870 | GDSL-motif lipase/hydrolase family protein, putative |
| OsAffx.12252.1.S1_at | 2.80 | 0.0216 | LOC_Os02g28240 | Transferase family protein |
| OsAffx.12262.1.S1_at | 10.68 | 0.0006 | LOC_Os02g29010 | Cupin family protein |
| OsAffx.12382.1.S1_at | 1.64 | 0.0060 | LOC_Os02g37060 | expressed protein |
| OsAffx.12501.1.S1_at | 1.74 | 0.0036 | LOC_Os02g45080 | myb-like DNA-binding domain, SHAQKYF class family protein |
| OsAffx.12520.1.S1_s_at | 9.20 | 0.0105 | LOC_Os02g46340 | Zinc finger, C3HC4 type family protein, expressed |
| OsAffx.12689.1.S1_s_at | 2.11 | 0.0065 | LOC_Os02g58250 | expressed protein |
| OsAffx.12824.1.S1_at | 6.40 | 0.0037 | LOC_Os03g11310 | expressed protein |
| OsAffx.12830.1.S1_at | 2.44 | 0.0346 | LOC_Os03g11890 | expressed protein |
| OsAffx.12906.1.S1_at | 3.27 | 0.0173 | LOC_Os03g18020 | rhodanese-like family protein, putative |
| OsAffx.13195.1.S1_at | 3.33 | 0.0021 | LOC_Os03g36080 | expressed protein |
| OsAffx.13521.1.S1_at | 9.49 | 0.0031 | Os03g0785200 | Mitochodrial transcription termination factor-related family protein. |
| OsAffx.13521.1.S1_x_at | 8.39 | 0.0032 | Os03g0785200 | Mitochodrial transcription termination factor-related family protein. |
| OsAffx.13616.1.S1_x_at | 3.01 | 0.0143 | LOC_Os02g58450 | Uncharacterized ACR, COG1399 family protein |
| OsAffx.13800.1.S1_s_at | 1.66 | 0.0098 | LOC_Os04g20990 | expressed protein |
| OsAffx.13891.1.S1_s_at | 12.68 | 0.0329 | LOC_Os04g20560 | Methyl-CpG binding domain containing protein, expressed |
| OsAffx.13950.2.S1_at | 6.18 | 0.0001 | LOC_Os04g24170 | RNA recognition motif family protein, expressed |
| OsAffx.14122.1.S1_at | 2.10 | 0.0251 | LOC_Os04g33940 | expressed protein |
| OsAffx.14131.1.S1_at | 6.26 | 0.0134 | LOC_Os04g34330 | D-mannose binding lectin family protein |
| OsAffx.14179.1.S1_s_at | 1.71 | 0.0107 | Os04g0451900 | Protein phosphatase 2C-like domain containing protein. |
| OsAffx.14459.1.S1_at | 8.74 | 0.0250 | LOC_Os04g57930 | Thioredoxin X, chloroplast precursor, putative, expressed |
| OsAffx.14480.1.S1_at | 2.80 | 0.0290 | LOC_Os04g59480 | POT family protein, expressed |
| OsAffx.14496.1.S1_at | 1.85 | 0.0080 | LOC_Os05g02030 | OB-fold nucleic acid binding domain containing protein |
| OsAffx.14582.1.S1_at | 2.19 | 0.0068 | LOC_Os05g07700 | 60S ribosomal protein L10-3, putative, expressed |
| OsAffx.14620.1.S1_at | 1.59 | 0.0108 | LOC_Os05g10480 | expressed protein |
| OsAffx.14661.1.S1_s_at | 3.83 | 0.0320 | Os05g0215600 | Conserved hypothetical protein. |
| OsAffx.14819.1.S1_at | 2.31 | 0.0238 | LOC_Os05g25060 | expressed protein |
| OsAffx.15124.1.S1_at | 4.06 | 0.0121 | LOC_Os05g46250 | expressed protein |
| OsAffx.15320.1.S1_x_at | 1.93 | 0.0193 | LOC_Os06g08850 | Cyclic nucleotide-gated ion channel 14, putative |
| OsAffx.15782.1.S1_at | 2.41 | 0.0005 | LOC_Os06g36650 | ABC transporter family protein, expressed |
| OsAffx.16018.1.S1_at | 3.76 | 0.0127 | LOC_Os06g51470 | expressed protein |
| OsAffx.16049.1.S1_at | 4.12 | 0.0013 | LOC_Os07g02540 | acetyltransferase, GNAT family protein, expressed |
| OsAffx.16139.1.S1_at | 3.52 | 0.0001 | LOC_Os07g07620 | exostosin family protein, putative, expressed |
| OsAffx.16345.1.S1_s_at | 2.03 | 0.0049 | LOC_Os07g20410 | expressed protein |
| OsAffx.16693.1.S1_x_at | 2.59 | 0.0162 | Os07g0626300 | Conserved hypothetical protein. |
| OsAffx.16825.1.S1_s_at | 19.99 | 0.0001 | LOC_Os08g04430 | expressed protein |
| OsAffx.16875.1.S1_x_at | 2.79 | 0.0198 | LOC_Os08g07390 | Mla1, putative, expressed |
| OsAffx.16965.1.S1_x_at | 2.63 | 0.0025 | LOC_Os08g12780 | expressed protein |
| OsAffx.17231.1.S1_at | 3.11 | 0.0137 | LOC_Os08g29770 | Endoglucanase precursor, putative, expressed |
| OsAffx.17386.1.S1_at | 2.04 | 0.0484 | LOC_Os08g37820 | expressed protein |
| OsAffx.17389.1.S1_s_at | 18.97 | 0.0213 | LOC_Os08g37930 | Rare lipoprotein A like double-psi beta-barrel containing |
| OsAffx.17491.1.S1_at | 5.07 | 0.0139 | LOC_Os08g43230 | TraB protein, putative, expressed |
| OsAffx.17638.1.S1_at | 1.99 | 0.0094 | LOC_Os09g10690 | expressed protein |
| OsAffx.17671.1.S1_at | 1.91 | 0.0347 | LOC_Os09g12790 | calcium-activated outward-rectifying potassium channel 6, |
| OsAffx.1782.2.S1_at | 5.64 | 0.0208 | LOC_Os01g22510 | Streptomyces cyclase/dehydrase family protein, expressed |
| OsAffx.17904.1.S1_at | 3.74 | 0.0107 | LOC_Os09g26190 | CBS domain containing protein, expressed |
| OsAffx.17904.1.S1_s_at | 3.21 | 0.0009 | LOC_Os09g26190 | CBS domain containing protein, expressed |
| OsAffx.17919.1.S1_at | 3.17 | 0.0285 | LOC_Os09g27090 | F-box domain containing protein |
| OsAffx.18004.1.S1_x_at | 4.78 | 0.0104 | LOC_Os09g36040 | Rhodanese-like domain containing protein, expressed |
| OsAffx.18601.1.S1_at | 4.20 | 0.0155 | LOC_Os10g41770 | expressed protein |
| OsAffx.18792.1.S1_x_at | 1.76 | 0.0195 | LOC_Os11g10880 | hypothetical protein |
| OsAffx.18836.1.S1_x_at | 1.75 | 0.0145 | LOC_Os11g13890 | Chlorophyll A-B binding protein, expressed |
| OsAffx.19033.1.S1_at | 3.51 | 0.0206 | LOC_Os11g26890 | expressed protein |
| OsAffx.1916.1.S1_s_at | 43.07 | 0.0380 | LOC_Os01g02710 | LRk-type protein, putative |
| OsAffx.1926.1.S1_at | 4.14 | 0.0176 | LOC_Os01g04130 | peptidyl-tRNA hydrolase family protein, expressed |
| OsAffx.19268.1.S1_at | 2.54 | 0.0038 | LOC_Os11g40120 | hypothetical protein |
| OsAffx.19451.3.S1_s_at | 17.37 | 0.0299 | LOC_Os02g13190 | expressed protein |
| OsAffx.19451.3.S1_x_at | 20.23 | 0.0170 | LOC_Os02g13190 | expressed protein |
| OsAffx.19559.1.S1_at | 35.84 | 0.0146 | LOC_Os12g08790 | expressed protein |
| OsAffx.19737.1.S1_x_at | 3.75 | 0.0086 | LOC_Os12g18900 | cysteine desulfurase, putative, expressed |
| OsAffx.19773.1.S1_at | 2.14 | 0.0438 | LOC_Os12g22090 | expressed protein |
| OsAffx.20119.1.S1_at | 2.34 | 0.0200 | LOC_Os12g27520 | Protein kinase AFC1, putative, expressed |
| OsAffx.21119.2.S1_at | 5.31 | 0.0086 | LOC_Os01g28520 | F-box domain containing protein |
| OsAffx.21381.1.S1_s_at | 1.95 | 0.0430 | LOC_Os01g42140 | expressed protein |
| OsAffx.21509.1.S1_at | 5.82 | 0.0428 | LOC_Os01g48360 | expressed protein |
| OsAffx.21616.1.S1_s_at | 4.11 | 0.0348 | LOC_Os01g54890 | AP2 domain containing protein, expressed |
| OsAffx.21812.1.S1_at | 2.36 | 0.0386 | unknown | unknown |
| OsAffx.22013.1.S1_at | 1.69 | 0.0167 | unknown | unknown |
| OsAffx.22431.1.S1_s_at | 2.30 | 0.0008 | LOC_Os07g12510 | AP2 domain containing protein, expressed |
| OsAffx.22955.1.S1_x_at | 1.95 | 0.0338 | LOC_Os06g16830 | retrotransposon protein, putative, Ty1-copia subclass |
| OsAffx.23156.1.S1_at | 5.94 | 0.0084 | LOC_Os01g07800 | hypothetical protein |
| OsAffx.23225.1.S1_x_at | 1.99 | 0.0029 | LOC_Os01g13430 | CAS/CSE protein, C-terminus containing protein, expressed |
| OsAffx.23268.1.S1_at | 1.57 | 0.0037 | LOC_Os01g16210 | Protein of unknown function, DUF614 containing protein |
| OsAffx.23277.1.S1_at | 5.96 | 0.0051 | Os01g0272800 | Conserved hypothetical protein. |
| OsAffx.23277.2.S1_at | 4.03 | 0.0283 | LOC_Os01g16620 | expressed protein |
| OsAffx.2362.1.S1_at | 5.33 | 0.0483 | LOC_Os01g59000 | Cytochrome P450 family protein, expressed |
| OsAffx.23740.1.S1_at | 2.53 | 0.0148 | LOC_Os01g50830 | expressed protein |
| OsAffx.23795.1.S1_at | 1.66 | 0.0048 | LOC_Os01g54930 | expressed protein |
| OsAffx.2388.1.S1_at | 3.04 | 0.0102 | LOC_Os01g62440 | RNB-like protein, expressed |
| OsAffx.23918.1.S1_x_at | 1.66 | 0.0139 | LOC_Os01g64780 | transmembrane protein, putative, expressed |
| OsAffx.23932.1.S1_at | 10.37 | 0.0204 | LOC_Os01g66000 | expressed protein |
| OsAffx.23944.2.S1_at | 2.48 | 0.0279 | LOC_Os05g34450 | AS2, putative, expressed |
| OsAffx.23959.2.S1_at | 4.66 | 0.0008 | LOC_Os01g67490 | OTU-like cysteine protease family protein, expressed |
| OsAffx.24022.1.S1_x_at | 4.48 | 0.0025 | LOC_Os01g73020 | Uncharacterised protein family containing protein |
| OsAffx.24035.1.S1_s_at | 2.93 | 0.0084 | LOC_Os01g73890 | Transcription initiation factor IIA gamma chain, putative, |
| OsAffx.24076.1.S1_at | 5.30 | 0.0337 | LOC_Os02g03010 | expressed protein |
| OsAffx.24076.1.S1_s_at | 4.41 | 0.0223 | LOC_Os02g03010 | expressed protein |
| OsAffx.24153.1.S1_s_at | 2.81 | 0.0191 | LOC_Os02g07690 | VQ motif family protein, expressed |
| OsAffx.24269.1.S1_at | 4.45 | 0.0021 | LOC_Os02g15660 | tetratricopeptide repeat, putative, expressed |
| OsAffx.24325.1.S1_at | 1.95 | 0.0422 | LOC_Os02g19440 | CbiX family protein, expressed |
| OsAffx.24550.1.S1_at | 7.68 | 0.0008 | LOC_Os02g33020 | SOUL heme-binding protein, expressed |
| OsAffx.24657.1.S1_at | 7.04 | 0.0228 | LOC_Os02g40110 | expressed protein |
| OsAffx.24693.1.S1_at | 1.95 | 0.0070 | LOC_Os02g42890 | Cytochrome b561 family protein, expressed |
| OsAffx.24694.1.S1_x_at | 2.32 | 0.0377 | LOC_Os02g42920 | expressed protein |
| OsAffx.24761.1.S1_at | 2.91 | 0.0126 | LOC_Os02g47120 | RelA/SpoT containing protein, expressed |
| OsAffx.24765.1.S1_at | 2.95 | 0.0240 | LOC_Os02g47360 | LAGLIDADG DNA endonuclease family protein, expressed |
| OsAffx.2480.1.S1_at | 1.85 | 0.0499 | LOC_Os02g01920 | diphosphomevalonate decarboxylase family protein, |
| OsAffx.24877.1.S1_at | 6.21 | 0.0215 | LOC_Os02g55130 | Serine carboxypeptidase II precursor, putative, expressed |
| OsAffx.24885.1.S1_at | 3.05 | 0.0117 | LOC_Os02g55540 | F-box domain containing protein, expressed |
| OsAffx.2490.1.S1_at | 2.84 | 0.0095 | LOC_Os02g02980 | Enhanced disease susceptibility 5, putative, expressed |
| OsAffx.25078.1.S1_s_at | 2.60 | 0.0203 | LOC_Os03g12930 | expressed protein |
| OsAffx.25081.1.S1_at | 1.52 | 0.0279 | LOC_Os03g13370 | TPR Domain containing protein, expressed |
| OsAffx.25184.1.S1_at | 2.41 | 0.0247 | LOC_Os03g21680 | phospholipid-translocating P-type ATPase, flippase family |
| OsAffx.25398.1.S1_x_at | 4.22 | 0.0324 | LOC_Os03g37640 | MATE efflux family protein, expressed |
| OsAffx.25620.1.S1_x_at | 2.28 | 0.0498 | LOC_Os03g48170 | Isoamylase N-terminal domain containing protein, expressed |
| OsAffx.25646.1.S1_at | 2.22 | 0.0140 | LOC_Os03g49710 | 30S ribosomal protein S13, putative, expressed |
| OsAffx.2572.1.S1_at | 2.76 | 0.0191 | LOC_Os02g10990 | 6-4 photolyase, putative, expressed |
| OsAffx.25889.1.S1_at | 3.01 | 0.0000 | LOC_Os02g05420 | expressed protein |
| OsAffx.25952.1.S1_x_at | 1.78 | 0.0321 | LOC_Os11g01600 | Macrophage migration inhibitory factor family protein |
| OsAffx.26050.2.S2_x_at | 1.84 | 0.0225 | LOC_Os05g35330 | Ribulose bisphosphate carboxylase large chain precursor |
| OsAffx.26050.8.S1_x_at | 1.78 | 0.0324 | LOC_Os04g16830 | RNA polymerase beta' subunit-2, putative |
| OsAffx.26103.1.S1_at | 3.75 | 0.0215 | LOC_Os04g20070 | Tropinone reductase homolog At1g07440, putative |
| OsAffx.26273.1.S1_at | 2.02 | 0.0051 | LOC_Os02g15560 | transposon protein, putative, Mutator sub-class, expressed |
| OsAffx.26288.1.S1_at | 1.61 | 0.0240 | LOC_Os04g32580 | expressed protein |
| OsAffx.26321.1.S1_at | 10.98 | 0.0171 | LOC_Os04g34290 | D-mannose binding lectin family protein, expressed |
| OsAffx.26381.1.S1_at | 1.59 | 0.0454 | LOC_Os04g38920 | expressed protein |
| OsAffx.26382.1.S1_at | 3.65 | 0.0041 | LOC_Os04g39060 | CRS1/YhbY domain containing protein, expressed |
| OsAffx.26384.8.S1_x_at | 1.95 | 0.0096 | LOC_Os05g09400 | expressed protein |
| OsAffx.26462.1.S1_at | 2.56 | 0.0089 | LOC_Os04g44590 | expressed protein |
| OsAffx.26590.1.S1_at | 1.99 | 0.0370 | LOC_Os04g53300 | Common central domain of tyrosinase family protein |
| OsAffx.26669.1.S1_at | 2.71 | 0.0116 | LOC_Os04g43030 | Lipase family protein, expressed |
| OsAffx.26722.2.S1_s_at | 3.46 | 0.0348 | LOC_Os05g03900 | WRKY DNA binding domain containing protein, expressed |
| OsAffx.27242.1.S1_x_at | 3.10 | 0.0112 | LOC_Os05g39680 | PX domain containing protein, expressed |
| OsAffx.27388.1.S1_at | 2.39 | 0.0024 | LOC_Os05g49920 | pentatricopeptide, putative, expressed |
| OsAffx.27399.1.S1_at | 1.80 | 0.0224 | LOC_Os05g50950 | PPR986-12, putative, expressed |
| OsAffx.27460.1.S1_at | 2.94 | 0.0109 | LOC_Os06g05060 | early flowering 3, putative, expressed |
| OsAffx.27508.126.S1_x_at | 1.67 | 0.0043 | LOC_Os12g08770 | Photosystem I reaction centre subunit N, |
| OsAffx.27597.1.S1_at | 4.17 | 0.0059 | LOC_Os10g13870 | expressed protein |
| OsAffx.27606.1.S1_at | 4.37 | 0.0107 | LOC_Os06g13220 | axi 1 gene, putative, expressed |
| OsAffx.27742.1.S1_at | 4.02 | 0.0140 | LOC_Os06g20890 | hypothetical protein |
| OsAffx.28001.1.S1_s_at | 7.73 | 0.0448 | LOC_Os06g39040 | NAD dependent epimerase/dehydratase family protein |
| OsAffx.2808.1.S1_s_at | 2.79 | 0.0319 | LOC_Os02g30410 | expressed protein |
| OsAffx.28133.1.S1_at | 18.84 | 0.0464 | LOC_Os06g46460 | expressed protein |
| OsAffx.28136.1.S1_at | 2.42 | 0.0133 | LOC_Os06g46590 | expressed protein |
| OsAffx.28174.1.S1_x_at | 1.78 | 0.0260 | LOC_Os06g48620 | Para-aminobenzoate synthase, putative, expressed |
| OsAffx.28387.1.S1_s_at | 1.98 | 0.0380 | LOC_Os07g10420 | expressed protein |
| OsAffx.28769.2.S1_at | 5.60 | 0.0280 | LOC_Os07g35880 | Beta-amylase, putative, expressed |
| OsAffx.28980.1.S1_at | 2.94 | 0.0216 | LOC_Os08g01600 | glycoside hydrolase family 28 protein, putative |
| OsAffx.29059.1.S1_s_at | 3.68 | 0.0302 | LOC_Os08g06060 | Mitogen-activated protein kinase homolog MMK2, putative, |
| OsAffx.29101.1.S1_at | 2.06 | 0.0001 | LOC_Os08g08440 | hypothetical protein |
| OsAffx.29152.1.S1_s_at | 2.34 | 0.0098 | Os08g0214900 | Hypothetical protein. |
| OsAffx.29627.2.S1_at | 4.12 | 0.0215 | LOC_Os08g41820 | Exo70 exocyst complex subunit family protein, expressed |
| OsAffx.29695.1.S1_s_at | 2.20 | 0.0465 | LOC_Os09g02710 | CBS domain containing protein, expressed |
| OsAffx.29871.1.S1_x_at | 2.03 | 0.0416 | LOC_Os09g16330 | PDR5-like ABC transporter, putative, expressed |
| OsAffx.29987.1.S1_at | 5.88 | 0.0115 | LOC_Os09g23430 | hypothetical protein |
| OsAffx.30042.1.S1_at | 2.34 | 0.0413 | LOC_Os09g26570 | CAAX amino terminal protease family protein, expressed |
| OsAffx.3006.1.S1_at | 3.63 | 0.0388 | LOC_Os02g49240 | expressed protein |
| OsAffx.30188.1.S1_s_at | 1.52 | 0.0048 | Os09g0555100 | Sulfotransferase family protein. |
| OsAffx.30475.9.S1_s_at | 7.23 | 0.0010 | LOC_Os12g10570 | ATP synthase beta chain |
| OsAffx.30524.1.S1_x_at | 5.98 | 0.0372 | LOC_Os10g25040 | Red chlorophyll catabolite reductase family protein |
| OsAffx.3056.1.S1_at | 6.90 | 0.0054 | LOC_Os02g53400 | expressed protein |
| OsAffx.30652.1.S1_at | 2.92 | 0.0270 | LOC_Os10g33410 | hypothetical protein |
| OsAffx.3106.1.S1_x_at | 2.71 | 0.0291 | LOC_Os02g40460 | S1 RNA binding domain containing protein, expressed |
| OsAffx.31341.1.S1_s_at | 2.32 | 0.0070 | LOC_Os11g37200 | Uncharacterized protein family UPF0005 containing protein, |
| OsAffx.31358.1.S1_at | 2.35 | 0.0086 | LOC_Os11g37990 | CRS1/YhbY domain containing protein, expressed |
| OsAffx.31580.1.S1_x_at | 3.33 | 0.0005 | LOC_Os12g04660 | Zinc finger, C3HC4 type family protein |
| OsAffx.31629.1.S1_at | 1.56 | 0.0360 | LOC_Os12g07230 | Calcium-dependent protein kinase, isoform AK1, putative, |
| OsAffx.31788.1.S1_s_at | 2.95 | 0.0158 | LOC_Os12g17830 | expressed protein |
| OsAffx.3180.1.S1_at | 2.16 | 0.0034 | LOC_Os03g09940 | Sulfate transporter 2.1, putative, expressed |
| OsAffx.3193.1.S1_at | 2.12 | 0.0422 | LOC_Os03g10780 | XPG I-region family protein, expressed |
| OsAffx.31942.1.S1_at | 3.23 | 0.0168 | LOC_Os12g30190 | hypothetical protein |
| OsAffx.32195.1.A1_at | 2.88 | 0.0333 | LOC_Os10g21346 | unspliced-genomic chloroplast 30S ribosomal protein S15, putative |
| OsAffx.32196.1.A1_at | 2.38 | 0.0418 | LOC_Os12g30460 | hypothetical protein |
| OsAffx.32204.1.A1_x_at | 1.83 | 0.0207 | unknown | unknown |
| OsAffx.32204.1.S1_x_at | 1.97 | 0.0135 | unknown | unknown |
| OsAffx.32206.1.S1_at | 3.31 | 0.0183 | LOC_Os04g168 | 19 DNA-directed RNA polymerase beta chain, putative, expressed |
| OsAffx.32206.1.S1_x_at | 2.87 | 0.0251 | LOC_Os02g24610 | DNA-directed RNA polymerase beta chain |
| OsAffx.32207.1.A1_at | 8.94 | 0.0021 | LOC_Os01g58020 | Ribulose bisphosphate carboxylase large chain precursor |
| OsAffx.32208.1.S1_x_at | 2.73 | 0.0194 | LOC_Os01g58010 | ATP synthase a chain, putative |
| OsAffx.32210.1.A1_at | 4.03 | 0.0050 | LOC_Os04g16748 | unspliced-genomic ATP synthase B chain, putative |
| OsAffx.32212.1.A1_at | 2.12 | 0.0056 | LOC_Os12g39060 | hypothetical protein |
| OsAffx.32220.1.A1_s_at | 4.62 | 0.0158 | LOC_Os04g16728 | unspliced-genomic chloroplast 30S ribosomal protein S15, putative |
| OsAffx.32220.1.S1_s_at | 2.49 | 0.0149 | LOC_Os10g21340 | NAD, putative |
| OsAffx.32221.1.A1_s_at | 3.32 | 0.0496 | LOC_Os10g21350 | retrotransposon protein, putative, Ty3-gypsy subclass |
| OsAffx.32221.1.S1_s_at | 2.04 | 0.0241 | LOC_Os12g34550 | ribosomal protein S15 containing protein |
| OsAffx.32225.1.S1_x_at | 3.70 | 0.0385 | LOC_Os01g09700 | 1-aminocyclopropane-1-carboxylate synthase 7, putative, ex |
| OsAffx.32229.1.S1_x_at | 2.21 | 0.0053 | LOC_Os10g21260 | NAD, putative |
| OsAffx.32230.1.A1_at | 5.49 | 0.0102 | unknown | unknown |
| OsAffx.32230.1.A1_x_at | 10.78 | 0.0145 | LOC_Os03g55874 | unspliced-genomic ATP synthase subunit beta,putative |
| OsAffx.32240.1.A1_at | 3.48 | 0.0253 | LOC_Os10g21396 | unspliced-genomic NADPH-dependent oxidoreductase,putative, expressed |
| OsAffx.32241.1.S1_x_at | 7.20 | 0.0165 | LOC_Os10g21340 | NAD, putative |
| OsAffx.32256.1.A1_x_at | 3.00 | 0.0214 | unknown | unknown |
| OsAffx.32256.1.S1_x_at | 3.75 | 0.0394 | unknown | unknown |
| OsAffx.32257.1.A1_at | 3.30 | 0.0104 | LOC_Os01g58049 | unspliced-genomic photosystem I assembly protein ycf4, putative, expressed |
| OsAffx.32257.1.S1_x_at | 2.16 | 0.0493 | Os01g0792400 | Photosystem I assembly protein ycf4. |
| OsAffx.32262.1.S1_x_at | 2.98 | 0.0089 | Os01g0881600 | Photosystem II reaction center J protein. |
| OsAffx.32263.1.S1_x_at | 2.22 | 0.0253 | LOC_Os03g45710 | ferredoxin family protein, expressed |
| OsAffx.32268.1.A1_x_at | 3.56 | 0.0334 | LOC_Os03g46010 | hypothetical protein |
| OsAffx.32268.1.S1_x_at | 2.18 | 0.0126 | LOC_Os02g05210 | retrotransposon protein, putative, Ty3-gypsy subclass |
| OsAffx.32313.1.A1_at | 2.93 | 0.0176 | LOC_Os08g15306 | unspliced-genomic chloroplast 50S ribosomal protein L20, putative |
| OsAffx.32313.1.S1_x_at | 4.87 | 0.0235 | LOC_Os08g15306 | unspliced-genomic chloroplast 50S ribosomal protein L20, putative |
| OsAffx.32314.1.A1_at | 2.61 | 0.0223 | LOC_Os06g39712 | Putative Clp protease homologue |
| OsAffx.32323.1.A1_at | 2.82 | 0.0440 | unknown | unknown |
| OsAffx.32324.1.A1_at | 4.53 | 0.0023 | LOC_Os04g16770 | unspliced-genomic photosynthetic reaction center protein, putative, expressed |
| OsAffx.32328.1.A1_at | 4.89 | 0.0088 | LOC_Os09g04690 | hypothetical protein |
| OsAffx.32329.1.S1_x_at | 5.24 | 0.0010 | LOC_Os04g30480 | hypothetical protein |
| OsAffx.32330.1.S1_x_at | 7.43 | 0.0010 | LOC_Os01g57940 | Serine/threonine-protein kinase RLCKVII, putative, express |
| OsAffx.32335.1.A1_at | 2.25 | 0.0490 | LOC_Os09g19960 | hypothetical protein |
| OsAffx.32336.1.A1_at | 2.60 | 0.0344 | LOC_Os08g17650 | Complex 1 protein containing protein, expressed |
| OsAffx.3309.1.S1_s_at | 6.63 | 0.0032 | LOC_Os03g24590 | mTERF family protein, expressed |
| OsAffx.3560.1.S1_at | 4.92 | 0.0078 | LOC_Os03g50080 | immunophilin, putative, expressed |
| OsAffx.3602.1.S1_at | 1.95 | 0.0133 | LOC_Os03g53390 | expressed protein |
| OsAffx.3615.1.S1_s_at | 7.91 | 0.0484 | LOC_Os03g55530 | N-acetyltransferase, putative, expressed |
| OsAffx.3874.1.S1_s_at | 1.62 | 0.0411 | Os04g0304000 | Conserved hypothetical protein. |
| OsAffx.4028.1.S1_x_at | 2.08 | 0.0095 | LOC_Os04g39190 | cell division protein FtsH, putative, expressed |
| OsAffx.4083.1.S1_at | 1.60 | 0.0171 | LOC_Os04g44200 | Oxygen-evolving enhancer protein 3, chloroplast precursor, |
| OsAffx.4104.1.S1_at | 3.49 | 0.0378 | LOC_Os04g46010 | pentatricopeptide, putative, expressed |
| OsAffx.4110.1.S1_at | 15.20 | 0.0026 | LOC_Os04g47290 | expressed protein |
| OsAffx.4145.1.S1_x_at | 4.44 | 0.0008 | LOC_Os04g51290 | expressed protein |
| OsAffx.4156.1.S1_at | 3.09 | 0.0051 | LOC_Os04g52520 | hypothetical protein |
| OsAffx.4277.1.S1_s_at | 3.58 | 0.0334 | LOC_Os05g08750 | cold-induced glucosyl transferase, putative, expressed |
| OsAffx.4648.1.S1_at | 3.72 | 0.0235 | LOC_Os05g47850 | CRS1/YhbY domain containing protein, expressed |
| OsAffx.4662.1.S1_at | 7.21 | 0.0002 | LOC_Os05g49320 | 50S ribosomal protein L12-1, chloroplast precursor |
| OsAffx.4983.2.S1_x_at | 5.80 | 0.0043 | LOC_Os02g58150 | expressed protein |
| OsAffx.5226.1.S1_at | 2.06 | 0.0191 | LOC_Os07g05960 | expressed protein |
| OsAffx.5257.1.S1_at | 4.34 | 0.0198 | LOC_Os07g08970 | expressed protein |
| OsAffx.543.1.S1_at | 1.55 | 0.0017 | unknown | unknown |
| OsAffx.5460.1.S1_at | 3.44 | 0.0492 | unknown | unknown |
| OsAffx.5487.1.S1_at | 3.63 | 0.0156 | LOC_Os07g30960 | CTF2A, putative, expressed |
| OsAffx.5582.1.S1_at | 4.12 | 0.0001 | LOC_Os07g40120 | pentatricopeptide, putative, expressed |
| OsAffx.5881.1.S1_s_at | 1.77 | 0.0167 | LOC_Os08g20400 | Adenylate cyclase family protein, expressed |
| OsAffx.6098.1.S1_at | 9.44 | 0.0279 | LOC_Os08g41000 | expressed protein |
| OsAffx.6114.1.S1_at | 2.39 | 0.0009 | LOC_Os08g42520 | hypothetical protein |
| OsAffx.6135.1.S1_s_at | 2.81 | 0.0306 | LOC_Os08g44470 | expressed protein |
| OsAffx.6438.1.S1_at | 2.66 | 0.0036 | LOC_Os09g29300 | expressed protein |
| OsAffx.6713.1.S1_at | 8.30 | 0.0422 | Os10g0350800 | Hypothetical protein. |
| OsAffx.6713.2.S1_at | 7.51 | 0.0271 | LOC_Os10g21020 | UMUC-like DNA repair family protein, putative |
| OsAffx.6960.1.S1_s_at | 1.94 | 0.0089 | LOC_Os11g01880 | hypothetical protein |
| OsAffx.7038.1.S1_s_at | 3.83 | 0.0142 | LOC_Os11g07580 | expressed protein |
| OsAffx.7294.1.S1_at | 7.79 | 0.0388 | LOC_Os11g34870 | expressed protein |
| OsAffx.7704.1.S1_s_at | 2.57 | 0.0153 | LOC_Os12g26030 | metallopeptidase family M24 containing protein, expressed |
| OsAffx.7764.1.S1_at | 4.50 | 0.0134 | LOC_Os12g32250 | WRKY DNA binding domain containing protein, expressed |
| OsAffx.7765.1.S1_at | 1.79 | 0.0053 | LOC_Os12g32280 | SWIB/MDM2 domain containing protein, expressed |
| OsAffx.7876.1.S1_s_at | 3.58 | 0.0422 | LOC_Os12g43140 | expressed protein |
| OsAffx.9908.1.S1_x_at | 6.01 | 0.0018 | LOC_Os01g12200 | expressed protein |
| OsAffx.9982.1.S1_at | 1.63 | 0.0035 | unknown | unknown |

Ratio 1/2 indicates signal1(avg)/signal2(avg) from Wilcoxon Rank-Sum tests

*P* is the probability associated with the *t*-tests.
