# Supplementary material for: Identification of rhizome-specific genes by genome-wide differential expression Analysis in Oryza longistaminata
Source: BMC Plant Biol. 2011 Jan 24;11:18. doi: 10.1186/1471-2229-11-18 (PMC3036607; doi:10.1186/1471-2229-11-18)
Supplement: Additional file 7 — The RT-PCR profiles of 21 selected tissue-specifically expressed genes. PPT file type, the RT-PCR profiles of the organ specific expressed genes [file 1471-2229-11-18-S7.PPT]

## Slide 1
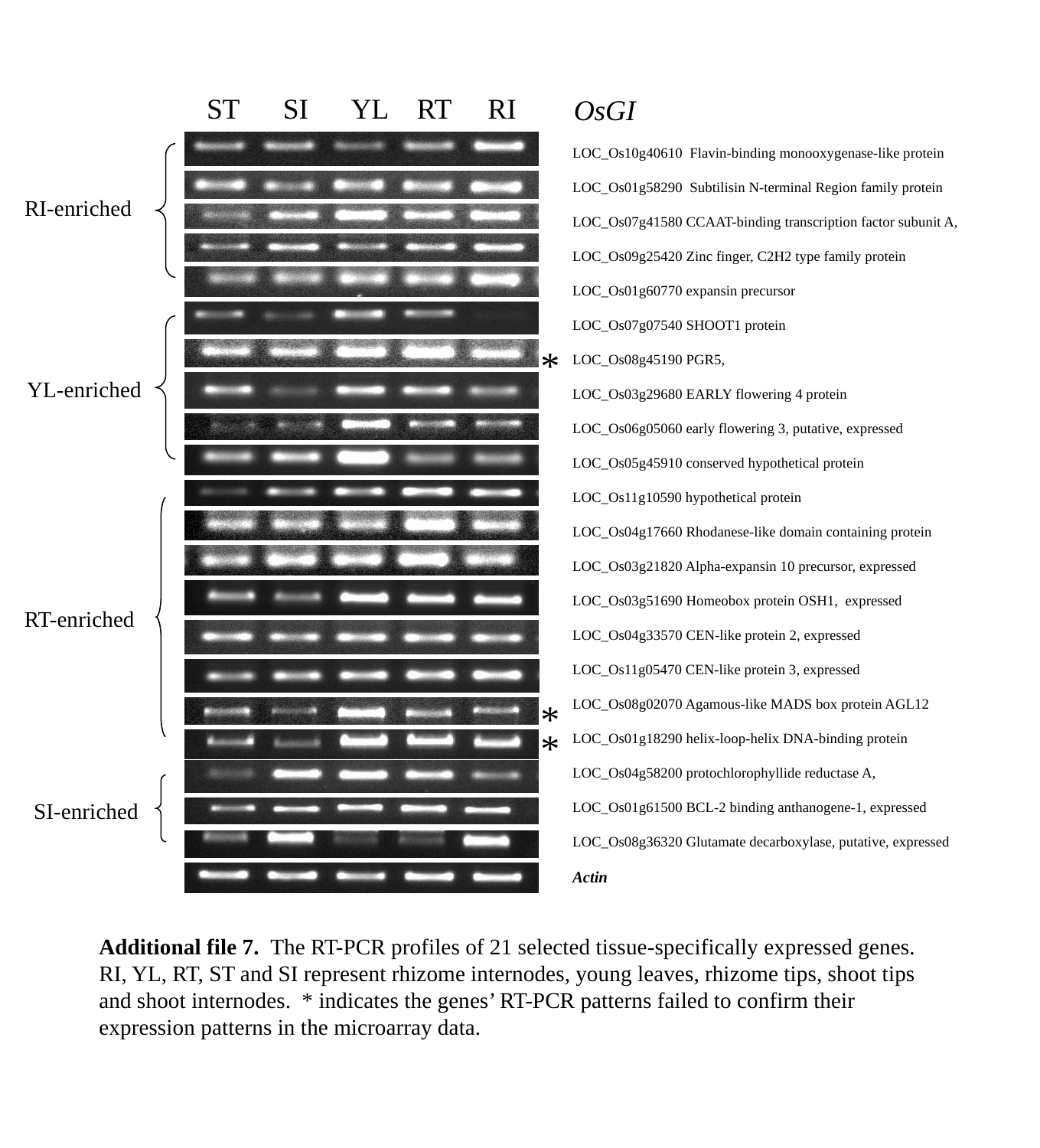

ST SI YL RT RI
OsGI
LOC_Os10g40610 Flavin-binding monooxygenase-like protein
LOC_Os01g58290 Subtilisin N-terminal Region family protein
LOC_Os07g41580 CCAAT-binding transcription factor subunit A,
LOC_Os09g25420 Zinc finger, C2H2 type family protein
LOC_Os01g60770 expansin precursor
LOC_Os07g07540 SHOOT1 protein
LOC_Os08g45190 PGR5,
LOC_Os03g29680 EARLY flowering 4 protein
LOC_Os06g05060 early flowering 3, putative, expressed
LOC_Os05g45910 conserved hypothetical protein
LOC_Os11g10590 hypothetical protein
LOC_Os04g17660 Rhodanese-like domain containing protein
LOC_Os03g21820 Alpha-expansin 10 precursor, expressed
LOC_Os03g51690 Homeobox protein OSH1, expressed
LOC_Os04g33570 CEN-like protein 2, expressed
LOC_Os11g05470 CEN-like protein 3, expressed
LOC_Os08g02070 Agamous-like MADS box protein AGL12
LOC_Os01g18290 helix-loop-helix DNA-binding protein
LOC_Os04g58200 protochlorophyllide reductase A,
LOC_Os01g61500 BCL-2 binding anthanogene-1, expressed
LOC_Os08g36320 Glutamate decarboxylase, putative, expressed
Actin
RI-enriched
*
YL-enriched
RT-enriched
*
*
SI-enriched
Additional file 7. The RT-PCR profiles of 21 selected tissue-specifically expressed genes. RI, YL, RT, ST and SI represent rhizome internodes, young leaves, rhizome tips, shoot tips and shoot internodes. * indicates the genes’ RT-PCR patterns failed to confirm their expression patterns in the microarray data.
