# Supplementary material for: Identification of rhizome-specific genes by genome-wide differential expression Analysis in Oryza longistaminata
Source: BMC Plant Biol. 2011 Jan 24;11:18. doi: 10.1186/1471-2229-11-18 (PMC3036607; doi:10.1186/1471-2229-11-18)
Supplement: Additional file 8 — The list of 424 genes up- and down-regulated in the rhizome tips (RT) relative to shoot tips (ST) of O. longistaminata and their annotated functions detected by the Affymetrix GeneChip Rice Genome Array. Word file for the list of up- and down-regulated genes in rhizome tip compared with shoot tip in Oryza longistaminata [file 1471-2229-11-18-S8.DOC]

**Additional file 8**. The list of 424 genes up- and down-regulated in the rhizome tips (RT) relative to shoot tips (ST) of *O. longistaminata* and their annotated functions detected by the Affymetrix GeneChip Rice Genome Array.

| **Probe Set ID** | **OsGI** | **Fold Change** | **Annotation** |
| --- | --- | --- | --- |
| Os.4766.1.S1_at | LOC_Os10g28350 | 3.28 | 1,2-dihydroxy-3-keto-5-methylthiopentene dioxygenase, putative, expressed |
| Os.49711.1.S1_at | LOC_Os08g35110 | 3.09 | auxin-responsive protein, putative, expressed |
| Os.9945.1.S1_at | LOC_Os03g43410 | 4.98 | AUX/IAA family protein, expressed |
| Os.9172.2.S1_at | LOC_Os02g02400 | 5.27 | Catalase isozyme A, putative, expressed |
| Os.7314.1.S1_at | LOC_Os10g22520 | 5.99 | Cellulase containing protein, expressed |
| Os.21634.1.S1_at | LOC_Os01g55240 | 5.75 | Gibberellin 2-beta-dioxygenase, putative, expressed |
| Os.21805.1.S1_s_at | LOC_Os06g51320 | 2.24 | Gibberellin regulated protein, expressed |
| Os.11445.1.S1_at | LOC_Os06g35970 | 8.04 | meiosis 5, putative, expressed |
| Os.8984.1.S1_at | LOC_Os02g13660 | 13.96 | meiosis 5, putative, expressed |
| Os.51101.1.S1_at | LOC_Os07g49370 | 2.65 | Glycosyltransferase family 43 protein, expressed |
| Os.55671.1.S1_at | LOC_Os06g44250 | 6.40 | Haemolysin-III related family protein, expressed |
| Os.6617.1.S1_s_at | LOC_Os09g15520 | 8.74 | Oleosin family protein, expressed |
| Os.9327.1.S1_at | LOC_Os05g45460 | 3.94 | Pollen proteins Ole e I family protein, expressed |
| Os.10678.1.S1_at | LOC_Os10g05990 | 17.45 | proline-rich protein, putative, expressed |
| Os.11398.1.S1_at | LOC_Os10g05980 | 6.47 | proline-rich protein, putative, expressed |
| Os.8203.1.S1_at | LOC_Os10g05750 | 2.27 | proline-rich protein, putative, expressed |
| Os.8385.1.S1_s_at | LOC_Os10g05970 | 7.23 | proline-rich protein, putative, expressed |
| Os.8385.2.S1_at | LOC_Os10g06000 | 8.54 | proline-rich protein, putative, expressed |
| Os.4079.2.S1_a_at | LOC_Os06g12790 | 2.42 | RAC-like GTP binding protein ARAC10 |
| Os.16980.1.S1_s_at | LOC_Os01g07560 | 2.39 | Leucine Rich Repeat family protein, expressed |
| Os.9534.1.S1_at | LOC_Os01g65830 | 3.99 | Acyl-desaturase, chloroplast precursor |
| Os.26839.1.S1_at | LOC_Os04g39020 | 2.32 | Betaine-aldehyde dehydrogenase, putative, expressed |
| Os.10251.1.S1_at | LOC_Os10g33370 | 2.36 | beta-ketoacyl-CoA synthase family protein |
| Os.12145.1.S1_at | LOC_Os02g47470 | 4.89 | Cytochrome P450 family protein, expressed |
| Os.17076.1.S1_at | LOC_Os09g10340 | 4.50 | Cytochrome P450 family protein, expressed |
| Os.33625.5.S1_x_at | LOC_Os01g70840 | 4.13 | Esterase PIR7B, putative, expressed |
| Os.6638.1.S1_x_at | LOC_Os01g57770 | 4.31 | hydrolase, alpha/beta fold family protein, expressed |
| Os.12035.1.S1_at | LOC_Os02g50130 | 3.09 | hydrolase, NUDIX family protein, expressed |
| Os.27969.1.S1_at | LOC_Os06g14420 | 4.09 | hydrolase, NUDIX family protein, expressed |
| Os.7085.2.S1_x_at | LOC_Os07g47700 | 2.96 | NAD-dependent epimerase/dehydratase family protein, putative, expressed |
| Os.24471.1.S1_at | LOC_Os07g30760 | 3.10 | UDP-glucoronosyl and UDP-glucosyl transferase family protein, expressed |
| OsAffx.15319.1.S1_at | LOC_Os06g08830 | 2.09 | UDP-glucoronosyl and UDP-glucosyl transferase family protein, expressed |
| Os.12416.1.S1_at | LOC_Os11g32650 | 3.32 | Chalcone synthase, putative, expressed |
| Os.10772.1.S1_x_at | LOC_Os06g06250 | 2.80 | GDSL-like Lipase/Acylhydrolase family protein |
| Os.43491.1.S1_x_at | LOC_Os01g11620 | 3.01 | GDSL-like Lipase/Acylhydrolase family protein |
| Os.50483.1.S1_at | LOC_Os04g42860 | 2.60 | GDSL-like Lipase/Acylhydrolase family protein |
| Os.5270.1.S1_at | LOC_Os05g44200 | 3.60 | GDSL-like Lipase/Acylhydrolase family protein |
| Os.5727.1.S1_at | LOC_Os06g05550 | 3.83 | GDSL-like Lipase/Acylhydrolase family protein |
| Os.8666.1.S1_at | LOC_Os02g57110 | 2.97 | GDSL-like Lipase/Acylhydrolase family protein |
| Os.38849.1.S1_at | LOC_Os02g51620 | 3.17 | Glycosyl hydrolase family 3 C terminal domain containing protein, expressed |
| Os.8256.1.S1_at | LOC_Os12g38760 | 3.36 | diphosphonucleotide phosphatase 1, putative, expressed |
| Os.3396.1.S1_at | LOC_Os06g49880 | 2.94 | B-box zinc finger family protein, expressed |
| Os.4698.1.S1_at | LOC_Os01g70770 | 2.48 | Glutathione S-transferase III, putative, expressed |
| Os.7911.1.S1_at | LOC_Os01g27210 | 3.45 | Glutathione S-transferase IV, putative, expressed |
| Os.50961.1.S1_at | LOC_Os03g58890 | 4.20 | oxidoreductase, 2OG-Fe oxygenase family protein, putative, expressed |
| Os.153.1.S1_at | LOC_Os05g41990 | 2.68 | Peroxidase 1 precursor |
| Os.11552.1.S2_at | LOC_Os04g59150 | 10.49 | Peroxidase 12 precursor, putative, expressed |
| Os.11557.1.S1_at | LOC_Os07g49360 | 3.19 | Peroxidase 21 precursor, putative, expressed |
| Os.11565.1.S1_at | LOC_Os10g02040 | 6.19 | Peroxidase 53 precursor, putative, expressed |
| Os.1153.1.S1_at | LOC_Os01g15830 | 2.58 | Peroxidase 72 precursor, putative, expressed |
| Os.14191.1.S1_at | LOC_Os08g04310 | 3.35 | Plastocyanin-like domain containing protein, expressed |
| Os.12922.1.S1_at | LOC_Os09g36700 | 7.73 | Ribonuclease T2 family protein, expressed |
| Os.49726.1.S1_at | LOC_Os11g05470 | 3.15 | CEN-like protein 2, putative, expressed |
| Os.8120.1.S1_at | LOC_Os04g33570 | 8.88 | CEN-like protein 2, putative, expressed |
| Os.5356.1.S1_at | LOC_Os01g38980 | 4.90 | calmodulin-binding family protein, putative, expressed |
| Os.9817.1.S1_at | LOC_Os04g28250 | 3.44 | cystatin, putative, expressed |
| Os.10058.1.S1_at | LOC_Os05g31620 | 5.37 | EF hand family protein, expressed |
| Os.6102.1.S1_s_at | LOC_Os06g40500 | 2.44 | phosphatidic acid phosphatase, putative, expressed |
| Os.11334.1.S1_x_at | LOC_Os09g25090 | 3.23 | Protein kinase domain containing protein, expressed |
| Os.18692.1.S1_at | LOC_Os02g43290 | 3.02 | Protein kinase domain containing protein, expressed |
| Os.8653.1.S1_at | LOC_Os02g32610 | 2.30 | Protein kinase domain containing protein, expressed |
| Os.14294.1.S1_at | LOC_Os09g15480 | 6.75 | Ser/Thr-rich protein T10 in DGCR region, putative, expressed |
| Os.24952.1.S1_at | LOC_Os04g57720 | 2.53 | Two-component response regulator ARR3 |
| Os.11244.1.S1_x_at | LOC_Os06g04200 | 2.27 | Granule-bound starch synthase 1, chloroplast precursor, putative, expressed |
| Os.49701.1.S1_at | LOC_Os04g33990 | 3.26 | Harpin-induced protein 1 containing protein, expressed |
| Os.55637.1.S1_at | LOC_Os05g45070 | 3.21 | Harpin-induced protein 1 containing protein, expressed |
| Os.8655.1.S1_at | LOC_Os06g31960 | 2.97 | Plant thionin family protein |
| OsAffx.4981.1.S1_at | LOC_Os06g32350 | 7.50 | Plant thionin family protein |
| Os.23224.1.S1_at | LOC_Os06g32600 | 9.54 | Plant thionin family protein, expressed |
| OsAffx.21963.1.S1_s_at | LOC_Os01g19770 | 2.96 | stress-inducible membrane pore protein |
| Os.26698.4.S1_s_at | LOC_Os01g19820 | 4.90 | universal stress protein family protein |
| Os.26698.1.S1_a_at | LOC_Os01g19820 | 6.11 | universal stress protein family protein, expressed |
| Os.7705.1.S1_at | LOC_Os04g54300 | 13.39 | wound induced protein, putative, expressed |
| Os.5682.1.S1_at | LOC_Os09g30320 | 4.23 | BURP domain containing protein, expressed |
| Os.37729.1.S1_s_at | LOC_Os01g03360 | 7.15 | Bowman-Birk type bran trypsin inhibitor precursor |
| Os.4653.1.S1_at | LOC_Os01g03340 | 3.74 | Bowman-Birk type bran trypsin inhibitor precursor |
| Os.49337.1.S1_at | LOC_Os02g39140 | 4.98 | bHLH transcription factor GBOF-1, putative, expressed |
| OsAffx.15137.1.S1_at | LOC_Os05g47650 | 6.07 | DNA-binding protein RAV1, putative, expressed |
| Os.28450.1.S1_at | LOC_Os01g70730 | 3.18 | flowering promoting factor-like 1, putative, expressed |
| Os.5311.1.S1_at | LOC_Os08g39630 | 2.52 | Helix-loop-helix DNA-binding domain containing protein |
| OsAffx.15360.1.S1_at | LOC_Os06g10820 | 7.44 | Helix-loop-helix DNA-binding domain containing protein |
| Os.6271.1.S1_at | LOC_Os07g39320 | 3.63 | Homeobox domain containing protein, expressed |
| Os.9086.1.S1_at | LOC_Os03g10210 | 4.04 | Homeobox domain containing protein, expressed |
| Os.13012.1.S1_at | LOC_Os03g49880 | 5.12 | TCP family transcription factor containing protein |
| Os.23028.1.S1_s_at | LOC_Os03g08490 | 3.89 | AP2 domain containing protein |
| Os.28406.1.S1_at | LOC_Os09g11480 | 4.98 | AP2 domain containing protein |
| Os.4893.1.S1_at | LOC_Os05g29810 | 3.54 | AP2 domain containing protein |
| Os.55579.1.S1_at | LOC_Os06g08340 | 4.38 | AP2 domain containing protein |
| Os.55837.1.S1_at | LOC_Os01g10370 | 5.06 | AP2 domain containing protein |
| Os.5816.1.S1_at | LOC_Os09g35010 | 7.19 | AP2 domain containing protein |
| Os.55684.1.S1_at | LOC_Os01g55150 | 4.46 | bZIP transcription factor family protein, expressed |
| OsAffx.2611.1.S1_at | LOC_Os02g14910 | 2.87 | bZIP transcription factor family protein, expressed |
| Os.27705.1.S1_at | LOC_Os01g18290 | 2.71 | Helix-loop-helix DNA-binding domain containing protein |
| Os.34471.1.S1_at | LOC_Os01g64310 | 4.35 | NAC-domain containing protein 90, putative, expressed |
| Os.33131.1.A1_at | LOC_Os03g20550 | 3.58 | WRKY DNA binding domain containing protein |
| Os.33534.1.S1_s_at | LOC_Os07g06620 | 3.66 | YABBY protein, putative, expressed |
| Os.20060.1.S1_at | LOC_Os03g05570 | 3.06 | Zinc finger, C3HC4 type family protein, expressed |
| Os.52944.1.A1_at | LOC_Os06g09310 | 4.40 | Zinc finger, C3HC4 type family protein, expressed |
| Os.12345.1.S1_s_at | LOC_Os02g44080 | 4.42 | aquaporin TIP-type RB7-5A, putative, expressed |
| Os.9273.1.S1_x_at | LOC_Os12g36220 | 18.53 | MPI, putative |
| Os.10120.1.S2_a_at | LOC_Os12g02340 | 10.60 | Nonspecific lipid-transfer protein 3 precursor |
| Os.4380.1.S1_at | LOC_Os11g02350 | 4.42 | Nonspecific lipid-transfer protein 4 precursor |
| Os.28462.1.S1_s_at | LOC_Os12g02290 | 3.08 | Nonspecific lipid-transfer protein 5 precursor |
| Os.36779.1.S1_s_at | LOC_Os12g02330 | 8.52 | Nonspecific lipid-transfer protein precursor |
| Os.7242.1.S1_at | LOC_Os07g07930 | 2.03 | Protease inhibitor/seed storage/LTP family protein |
| Os.9151.1.S1_at | LOC_Os07g09970 | 2.68 | Protease inhibitor/seed storage/LTP family protein |
| OsAffx.27248.1.S1_x_at | LOC_Os05g40010 | 2.90 | Protease inhibitor/seed storage/LTP family protein |
| Os.34982.1.A1_at | LOC_Os04g17660 | 4.81 | Rhodanese-like domain containing protein, expressed |
| Os.6838.1.S1_at | LOC_Os10g40510 | 6.42 | Cortical cell delineating protein precursor |
| Os.12176.1.S1_at | LOC_Os03g07590 | 6.18 | expressed protein |
| Os.12856.1.S1_x_at | LOC_Os05g46240 | 2.92 | expressed protein |
| Os.15026.1.S1_at | LOC_Os09g37540 | 12.59 | expressed protein |
| Os.20153.1.S1_x_at | LOC_Os01g09030 | 4.79 | expressed protein |
| Os.3496.1.S1_at | LOC_Os01g12110 | 25.60 | expressed protein |
| Os.35688.1.S1_at | LOC_Os11g44870 | 3.52 | expressed protein |
| Os.37622.1.S1_at | LOC_Os01g67010 | 11.36 | expressed protein |
| Os.38806.1.A1_x_at | LOC_Os06g04230 | 3.40 | expressed protein |
| Os.4683.2.S1_at | LOC_Os01g42520 | 3.61 | expressed protein |
| Os.47997.1.A1_at | LOC_Os01g43950 | 3.92 | expressed protein |
| Os.48260.1.S1_at | LOC_Os02g52490 | 2.44 | expressed protein |
| Os.49159.1.A1_x_at | LOC_Os05g32970 | 2.38 | expressed protein |
| Os.4940.1.S1_at | LOC_Os06g46970 | 5.79 | expressed protein |
| Os.50862.1.S1_x_at | LOC_Os10g37240 | 2.88 | expressed protein |
| Os.52493.1.A1_s_at | LOC_Os03g22210 | 3.90 | expressed protein |
| Os.52694.1.S1_at | LOC_Os06g46030 | 2.20 | expressed protein |
| Os.53467.1.S1_at | LOC_Os03g22230 | 3.90 | expressed protein |
| Os.54229.1.S1_at | LOC_Os04g36670 | 5.24 | expressed protein |
| Os.5592.1.S1_at | LOC_Os02g48710 | 4.58 | expressed protein |
| Os.6417.1.S1_at | LOC_Os07g37850 | 3.59 | expressed protein |
| Os.7983.1.S1_at | LOC_Os01g58140 | 14.34 | expressed protein |
| Os.8682.1.S1_a_at | LOC_Os10g08780 | 10.12 | expressed protein |
| Os.9072.1.S1_at | LOC_Os11g30360 | 6.12 | expressed protein |
| Os.9559.1.S1_at | LOC_Os03g37570 | 4.77 | expressed protein |
| Os.9913.1.S1_at | LOC_Os04g54230 | 13.37 | expressed protein |
| OsAffx.16059.1.S1_at | LOC_Os07g03120 | 4.50 | expressed protein |
| OsAffx.23168.1.S1_at | LOC_Os01g09030 | 3.55 | expressed protein |
| OsAffx.27508.7.S1_s_at | LOC_Os11g44380 | 4.61 | expressed protein |
| OsAffx.27557.1.S1_at | LOC_Os02g52880 | 3.87 | expressed protein |
| OsAffx.7458.1.S1_x_at | LOC_Os12g02020 | 6.55 | expressed protein |
| Os.11272.1.S1_at | LOC_Os02g37070 | 3.34 | hypothetical protein |
| Os.9836.1.S1_at | LOC_Os11g10590 | 14.12 | hypothetical protein |
| OsAffx.29465.1.S1_at | LOC_Os08g32290 | 2.35 | hypothetical protein |
| Os.6042.1.S1_at | LOC_Os07g25810 | 4.96 | transposon protein, |
| Os.9975.1.S1_at | LOC_Os03g17200 | 8.54 | uncharacterized plant-specific domain |
| Os.10534.1.S1_at | unknown | 8.39 | unknown |
| Os.14042.1.S1_at | unknown | 2.43 | unknown |
| Os.26968.1.S1_at | unknown | 5.93 | unknown |
| Os.27520.2.S1_x_at | unknown | 21.11 | unknown |
| Os.28030.2.A1_at | unknown | 3.89 | unknown |
| Os.46645.1.S1_at | unknown | 4.60 | unknown |
| Os.4867.1.S1_at | unknown | 5.92 | unknown |
| Os.51360.1.S1_at | unknown | 3.35 | unknown |
| Os.52641.1.S1_at | unknown | 2.65 | unknown |
| Os.57006.1.S1_at | unknown | 2.42 | unknown |
| Os.5843.1.S1_at | unknown | 3.79 | unknown |
| Os.6764.2.S1_at | unknown | 7.82 | unknown |
| Os.7431.1.S1_a_at | unknown | 2.51 | unknown |
| Os.9167.1.A1_at | unknown | 3.71 | unknown |
| OsAffx.22476.1.S1_x_at | unknown | 3.62 | unknown |
| OsAffx.24682.1.S1_s_at | unknown | 5.70 | unknown |
| OsAffx.27291.1.S1_at | unknown | 3.19 | unknown |
| OsAffx.27508.100.S1_s_at | unknown | 3.64 | unknown |
| Os.12295.1.S1_at | LOC_Os04g33830 | 0.03 | 16kDa membrane protein, putative, expressed |
| Os.519.1.S1_at | LOC_Os01g08860 | 0.22 | 17.5 kDa class II heat shock protein, putative, expressed |
| Os.47381.1.S1_x_at | LOC_Os04g36800 | 0.43 | 3-oxoacyl-synthase I, chloroplast precursor |
| Os.37384.1.S1_s_at | LOC_Os05g06510 | 0.46 | 4Fe-4S binding domain containing protein, expressed |
| Os.24545.2.S1_x_at | LOC_Os03g30300 | 0.47 | 6-phosphogluconolactonase family protein, expressed |
| Os.10975.1.S1_at | LOC_Os11g39020 | 0.48 | ABC transporter family protein, expressed |
| Os.9904.2.S1_x_at | LOC_Os01g67720 | 0.50 | ABC1 family protein, expressed |
| Os.11616.1.S1_at | LOC_Os02g36570 | 0.39 | ABC1 family protein, putative, expressed |
| Os.21096.1.S1_at | LOC_Os03g63500 | 0.38 | AFG1-like ATPase family protein, putative, expressed |
| Os.37813.1.S1_at | LOC_Os10g39130 | 0.31 | Agamous-like MADS box protein AGL19 |
| Os.11846.1.S1_at | LOC_Os05g34980 | 0.46 | amino acid carrier, putative, expressed |
| Os.17259.1.S1_at | LOC_Os01g65660 | 0.33 | amino acid permease, putative, expressed |
| Os.15600.1.S1_a_at | LOC_Os12g07110 | 0.16 | AMP-binding enzyme family protein, expressed |
| Os.7979.1.S1_at | LOC_Os03g63480 | 0.37 | Ankyrin repeat domain protein 2, putative, expressed |
| OsAffx.32126.1.S1_at | LOC_Os12g40960 | 0.09 | AP2 domain containing protein |
| Os.8118.1.S1_at | LOC_Os07g26630 | 0.12 | Aquaporin PIP2.2, putative, expressed |
| Os.18459.2.S1_x_at | LOC_Os01g66590 | 0.44 | AS2, putative, expressed |
| Os.3623.1.S1_at | LOC_Os01g18630 | 0.28 | Aspartic proteinase precursor, putative, expressed |
| Os.49163.1.S1_at | LOC_Os03g47730 | 0.48 | Associated with HOX family protein |
| Os.11431.1.S1_at | LOC_Os04g49990 | 0.41 | AT hook motif family protein, expressed |
| Os.11860.1.S1_at | LOC_Os05g05600 | 0.43 | ATA15 protein, putative, expressed |
| Os.50938.1.S1_at | LOC_Os11g04190 | 0.49 | Auxin Efflux Carrier family protein, expressed |
| Os.7177.2.S1_a_at | LOC_Os04g57610 | 0.47 | Auxin response factor 8, putative, expressed |
| Os.49319.1.S1_at | LOC_Os02g52990 | 0.44 | Auxin responsive protein, expressed |
| Os.12805.1.S1_at | LOC_Os04g41560 | 0.43 | B-box zinc finger family protein, expressed |
| Os.3397.1.S1_at | LOC_Os02g39360 | 0.37 | B-box zinc finger family protein, expressed |
| Os.1502.1.S1_at | LOC_Os02g07840 | 0.49 | bZIP transcription factor family protein, expressed |
| Os.7676.1.S1_at | LOC_Os02g49680 | 0.20 | calcium sensing receptor, putative, expressed |
| Os.17487.1.S1_at | LOC_Os03g50310 | 0.18 | CCT motif family protein, expressed |
| Os.22029.1.S1_a_at | LOC_Os10g41780 | 0.19 | chlorophyll a oxygenase, putative, expressed |
| Os.28216.1.S1_a_at | LOC_Os01g52240 | 0.12 | Chlorophyll a-b binding protein 2, chloroplast precursor |
| Os.7941.2.S1_a_at | LOC_Os01g41710 | 0.02 | Chlorophyll a-b binding protein 2, chloroplast precursor, putative, expressed |
| Os.7890.1.S1_x_at | LOC_Os08g33820 | 0.20 | Chlorophyll a-b binding protein 4, chloroplast precursor, putative, expressed |
| Os.12713.1.S1_at | LOC_Os06g21590 | 0.09 | Chlorophyll a-b binding protein 6A, chloroplast precursor, putative, expressed |
| Os.28403.1.S1_a_at | LOC_Os02g10390 | 0.07 | Chlorophyll a-b binding protein 8, chloroplast precursor, putative, expressed |
| Os.5869.1.S1_a_at | LOC_Os04g38410 | 0.20 | Chlorophyll a-b binding protein CP24 10B, chloroplast precursor |
| Os.5869.3.S1_x_at | LOC_Os04g38410 | 0.27 | Chlorophyll a-b binding protein CP24 10B, chloroplast precursor, putative, expressed |
| Os.28216.3.S1_x_at | LOC_Os07g37550 | 0.13 | Chlorophyll a-b binding protein of LHCII type III, chloroplast precursor |
| Os.52535.1.S1_x_at | LOC_Os09g26810 | 0.36 | Chlorophyll a-b binding protein, chloroplast precursor, putative, expressed |
| Os.12296.1.S1_at | LOC_Os03g39610 | 0.06 | Chlorophyll a-b binding protein, chloroplast precursor, putative, expressed |
| OsAffx.18836.1.S1_x_at | LOC_Os11g13890 | 0.06 | Chlorophyll A-B binding protein, expressed |
| Os.8014.2.S1_x_at | LOC_Os10g10180 | 0.28 | CMV 1a interacting protein 1, putative, expressed |
| Os.30608.1.S1_x_at | LOC_Os10g10180 | 0.14 | CMV 1a interacting protein 1, putative, expressed |
| Os.6595.1.S1_a_at | LOC_Os07g46310 | 0.30 | CobN/magnesium chelatase family protein |
| Os.50763.1.S1_at | LOC_Os03g30260 | 0.33 | COBRA-like protein 2 precursor, putative, expressed |
| Os.46598.1.S1_a_at | LOC_Os04g42020 | 0.17 | CONSTANS, putative, expressed |
| Os.10732.1.S1_s_at | LOC_Os03g19380 | 0.19 | CP12, putative, expressed |
| Os.8081.1.A1_at | LOC_Os07g36390 | 0.43 | CRP1, putative, expressed |
| Os.14817.1.S1_a_at | LOC_Os10g36860 | 0.35 | CRS1/YhbY domain containing protein, expressed |
| Os.25325.1.A1_at | LOC_Os06g49200 | 0.22 | CUE domain containing protein, expressed |
| OsAffx.11954.1.S1_s_at | LOC_Os02g09420 | 0.36 | cyclase family protein, putative, expressed |
| Os.2248.1.S1_at | LOC_Os07g37030 | 0.32 | Cytochrome b6-f complex iron-sulfur subunit, chloroplast precursor, putative, expressed |
| Os.17180.1.S1_at | LOC_Os08g43390 | 0.21 | Cytochrome P450 78A3, putative, expressed |
| Os.10200.1.S1_at | LOC_Os08g05620 | 0.11 | Cytochrome P450 89A2, putative, expressed |
| Os.15204.2.S1_x_at | LOC_Os07g49110 | 0.32 | D-alanine ligase family, putative, expressed |
| Os.11002.1.S1_at | LOC_Os08g44210 | 0.05 | Dihydroneopterin aldolase, putative, expressed |
| Os.9679.1.S1_at | LOC_Os11g07830 | 0.33 | disease resistance-responsive family protein |
| Os.11987.1.S1_at | LOC_Os03g07360 | 0.41 | Dof domain, zinc finger family protein, expressed |
| Os.28370.1.A1_s_at | LOC_Os06g47000 | 0.47 | EF hand family protein, expressed |
| Os.26798.1.S1_at | LOC_Os12g35630 | 0.34 | Elongation factor TS family protein, expressed |
| Os.21318.1.S1_at | LOC_Os06g04510 | 0.08 | Enolase 1, putative, expressed |
| Os.46397.1.S1_x_at | LOC_Os10g35490 | 0.16 | epoxide hydrolase, putative, expressed |
| Os.6791.1.S1_x_at | LOC_Os01g72510 | 0.26 | Eukaryotic aspartyl protease family protein, expressed |
| OsAffx.30394.1.S1_at | LOC_Os10g11820 | 0.49 | expressed protein |
| Os.51208.1.S1_s_at | LOC_Os02g57020 | 0.48 | expressed protein |
| Os.55328.1.S1_at | LOC_Os07g14890 | 0.47 | expressed protein |
| Os.23164.1.S1_a_at | LOC_Os07g39980 | 0.46 | expressed protein |
| Os.19296.2.S1_at | LOC_Os06g38320 | 0.46 | expressed protein |
| Os.22796.1.S1_at | LOC_Os03g47140 | 0.46 | expressed protein |
| Os.20351.1.S1_at | LOC_Os03g63360 | 0.46 | expressed protein |
| Os.46471.1.S1_at | LOC_Os10g20650 | 0.45 | expressed protein |
| Os.27679.1.A1_at | LOC_Os02g42960 | 0.45 | expressed protein |
| Os.32622.1.S1_at | LOC_Os01g01170 | 0.45 | expressed protein |
| OsAffx.4181.1.S1_at | LOC_Os04g55130 | 0.44 | expressed protein |
| Os.47558.1.S1_at | LOC_Os08g33680 | 0.44 | expressed protein |
| Os.16772.1.S1_at | LOC_Os08g27010 | 0.44 | expressed protein |
| Os.27626.1.S1_at | LOC_Os06g05950 | 0.44 | expressed protein |
| Os.49593.1.S1_x_at | LOC_Os09g29060 | 0.42 | expressed protein |
| Os.21243.1.S1_at | LOC_Os02g36490 | 0.41 | expressed protein |
| Os.26587.1.A1_at | LOC_Os04g11400 | 0.40 | expressed protein |
| OsAffx.18947.1.S1_x_at | LOC_Os11g19730 | 0.40 | expressed protein |
| Os.50398.1.S1_at | LOC_Os02g44090 | 0.39 | expressed protein |
| Os.9814.1.S1_at | LOC_Os01g55570 | 0.39 | expressed protein |
| Os.38316.1.S1_s_at | LOC_Os01g62200 | 0.39 | expressed protein |
| OsAffx.12382.1.S1_at | LOC_Os02g37060 | 0.39 | expressed protein |
| Os.55696.1.S1_at | LOC_Os12g39100 | 0.38 | expressed protein |
| Os.52938.1.S1_a_at | LOC_Os04g59610 | 0.38 | expressed protein |
| OsAffx.25555.1.S1_s_at | LOC_Os03g45270 | 0.38 | expressed protein |
| Os.26349.1.S1_at | LOC_Os04g44590 | 0.38 | expressed protein |
| Os.27642.1.S1_at | LOC_Os02g30320 | 0.37 | expressed protein |
| Os.53755.1.S1_at | LOC_Os07g09370 | 0.36 | expressed protein |
| Os.8469.1.S1_at | LOC_Os09g34140 | 0.35 | expressed protein |
| Os.32447.1.S1_at | LOC_Os01g59080 | 0.35 | expressed protein |
| Os.10003.1.S1_at | LOC_Os01g13390 | 0.34 | expressed protein |
| OsAffx.31174.3.S1_x_at | LOC_Os04g51490 | 0.33 | expressed protein |
| Os.51371.1.S1_at | LOC_Os02g40410 | 0.32 | expressed protein |
| Os.51086.1.S1_x_at | LOC_Os08g02630 | 0.32 | expressed protein |
| Os.12058.1.S1_at | LOC_Os02g49870 | 0.30 | expressed protein |
| Os.32455.1.S1_at | LOC_Os01g54670 | 0.29 | expressed protein |
| Os.14493.1.S1_at | LOC_Os02g03670 | 0.28 | expressed protein |
| Os.35273.1.S1_at | LOC_Os07g29240 | 0.28 | expressed protein |
| Os.33308.1.S1_at | LOC_Os04g53490 | 0.26 | expressed protein |
| Os.810.2.S1_x_at | LOC_Os01g01510 | 0.25 | expressed protein |
| Os.19296.1.S1_at | LOC_Os06g38320 | 0.24 | expressed protein |
| Os.29068.1.S1_at | LOC_Os03g56200 | 0.22 | expressed protein |
| Os.10410.1.S1_at | LOC_Os08g02210 | 0.22 | expressed protein |
| Os.8455.1.S1_at | LOC_Os07g28610 | 0.19 | expressed protein |
| Os.7865.1.S1_at | LOC_Os06g15400 | 0.19 | expressed protein |
| Os.32492.1.S1_at | LOC_Os03g14040 | 0.15 | expressed protein |
| Os.12624.1.S1_s_at | LOC_Os07g37240 | 0.11 | expressed protein |
| Os.12393.2.S1_s_at | LOC_Os08g01380 | 0.13 | Ferredoxin-1, chloroplast precursor, putative, expressed |
| Os.10696.1.S1_at | LOC_Os06g01850 | 0.33 | Ferredoxin-NADP reductase, leaf isozyme, chloroplast precursor, putative, expressed |
| Os.27686.1.S1_at | LOC_Os02g42570 | 0.33 | Ferredoxin-thioredoxin reductase, variable chain, putative, expressed |
| Os.56099.1.S1_at | LOC_Os01g62610 | 0.34 | FK506-binding protein 4, putative, expressed |
| Os.8230.1.S1_at | LOC_Os08g42850 | 0.40 | FKBP-type peptidyl-prolyl cis-trans isomerase 2, chloroplast precursor, putative, expressed |
| Os.2348.1.S1_at | LOC_Os03g54160 | 0.10 | Floral homeotic protein APETALA1, putative, expressed |
| Os.27745.1.A1_at | LOC_Os06g12600 | 0.44 | Fructokinase, putative, expressed |
| Os.11756.1.S1_s_at | LOC_Os03g16050 | 0.24 | Fructose-1,6-bisphosphatase, chloroplast precursor, putative, expressed |
| Os.7988.1.S1_s_at | LOC_Os11g07020 | 0.23 | Fructose-bisphosphate aldolase, chloroplast precursor, putative, expressed |
| Os.55686.2.S1_at | LOC_Os02g41910 | 0.24 | Gamma-thionins family protein, expressed |
| Os.53733.1.S1_at | LOC_Os02g12790 | 0.38 | GATA zinc finger family protein, expressed |
| Os.10275.1.S1_at | LOC_Os02g51080 | 0.40 | Geranylgeranyl hydrogenase, putative, expressed |
| Os.20397.1.S1_a_at | LOC_Os03g52460 | 0.41 | Glucose-1-phosphate adenylyltransferase large subunit 3, chloroplast precursor, putative, expressed |
| Os.7879.1.S1_at | LOC_Os04g56400 | 0.35 | Glutamine synthetase, chloroplast precursor |
| Os.4329.1.S1_at | LOC_Os03g03720 | 0.12 | Glyceraldehyde-3-phosphate dehydrogenase B, chloroplast precursor, putative, expressed |
| Os.8488.1.S1_s_at | LOC_Os10g37180 | 0.22 | Glycine cleavage system H protein, mitochondrial precursor, putative, expressed |
| Os.15324.1.S1_at | LOC_Os06g02130 | 0.43 | G-protein alpha subunit, putative, expressed |
| Os.32618.1.S1_at | LOC_Os06g02560 | 0.36 | growth-regulating factor, putative, expressed |
| Os.17566.1.S1_at | LOC_Os02g18450 | 0.32 | GTP-binding protein TypA/BipA homolog |
| Os.7140.1.S1_at | LOC_Os11g16550 | 0.20 | GUN4-like family protein, expressed |
| Os.51415.1.S1_at | LOC_Os05g34790 | 0.49 | GYF domain containing protein, expressed |
| Os.27862.1.S1_at | LOC_Os11g38260 | 0.35 | harpin binding protein 1, putative, expressed |
| Os.11822.1.S1_at | LOC_Os03g60620 | 0.42 | Heat shock cognate 70 kDa protein 2, putative, expressed |
| Os.38581.1.S1_s_at | LOC_Os05g38530 | 0.33 | Heat shock cognate 70 kDa protein, putative, expressed |
| Os.2292.3.S1_x_at | LOC_Os03g53340 | 0.07 | Heat shock factor protein HSF30, putative, expressed |
| Os.12257.1.S1_at | LOC_Os02g52150 | 0.19 | Hsp20/alpha crystallin family protein, expressed |
| Os.11566.1.S1_at | LOC_Os02g47620 | 0.29 | hydrolase, alpha/beta fold family protein, expressed |
| Os.55604.1.S1_at | LOC_Os03g24070 | 0.22 | hydrolase, putative, expressed |
| Os.49940.1.S1_at | LOC_Os04g10650 | 0.48 | hydroxyproline-rich glycoprotein family protein, putative, expressed |
| OsAffx.5213.1.S1_x_at | LOC_Os07g04980 | 0.46 | hypothetical protein |
| OsAffx.24672.1.S1_at | LOC_Os02g41770 | 0.42 | hypothetical protein |
| OsAffx.12089.1.S1_at | LOC_Os02g17620 | 0.34 | isochorismatase family protein, expressed |
| Os.8649.1.S1_at | LOC_Os11g43590 | 0.47 | Kelch motif family protein, expressed |
| Os.17232.1.S2_at | LOC_Os04g51300 | 0.28 | L-ascorbate peroxidase, chloroplast precursor |
| Os.17393.1.S1_at | LOC_Os01g12810 | 0.23 | leaf protein, putative, expressed |
| OsAffx.24863.1.S1_x_at | LOC_Os02g54170 | 0.49 | Leucine Rich Repeat family protein |
| Os.48036.1.A1_at | LOC_Os09g15700 | 0.46 | Leucine Rich Repeat family protein, expressed |
| Os.54353.1.S1_at | LOC_Os06g04370 | 0.45 | leucine-rich repeat transmembrane protein kinase, putative, expressed |
| Os.54791.1.S1_at | LOC_Os02g53000 | 0.49 | LysM domain GPI-anchored protein 1 precursor, putative, expressed |
| Os.50876.1.S1_at | LOC_Os06g04150 | 0.48 | Magnesium-protoporphyrin O-methyltransferase, putative, expressed |
| Os.7496.1.S1_a_at | LOC_Os10g29470 | 0.46 | mannitol dehydrogenase |
| Os.12703.1.S1_at | LOC_Os03g10090 | 0.28 | mannitol transporter, putative, expressed |
| Os.6998.1.S1_at | LOC_Os03g37490 | 0.47 | MATE efflux family protein, expressed |
| OsAffx.12806.1.S1_x_at | LOC_Os03g10220 | 0.30 | Methyladenine glycosylase family protein, expressed |
| OsAffx.18042.1.S1_at | LOC_Os09g38100 | 0.44 | Mitochondrial carrier protein |
| Os.11670.1.S1_at | LOC_Os11g24450 | 0.35 | Mitochondrial carrier protein, expressed |
| Os.55640.1.S1_at | LOC_Os02g37000 | 0.36 | Mitochondrial prohibitin complex protein 1 |
| Os.52106.1.S1_at | LOC_Os02g51450 | 0.43 | mTERF family protein, expressed |
| OsAffx.3309.1.S1_s_at | LOC_Os03g24590 | 0.27 | mTERF family protein, expressed |
| Os.3340.1.S1_a_at | LOC_Os06g24070 | 0.42 | myb-like DNA-binding domain, SHAQKYF class family protein, expressed |
| Os.5739.1.S1_at | LOC_Os05g49300 | 0.25 | NifU-like N-terminal domain containing protein, mitochondrial precursor, putative, expressed |
| Os.26393.1.S1_at | LOC_Os06g49120 | 0.46 | NmrA-like family protein, expressed |
| Os.15708.1.S1_a_at | LOC_Os04g38720 | 0.25 | No apical meristem protein, expressed |
| OsAffx.20681.1.S1_s_at | LOC_Os06g10750 | 0.23 | nodulin MtN21 family protein, putative, expressed |
| Os.18714.1.S1_a_at | LOC_Os10g14920 | 0.20 | nodulin MtN21 family protein, putative, expressed |
| Os.6664.1.S1_at | LOC_Os05g47700 | 0.48 | nonspecific lipid-transfer protein AKCS9 precursor, putative, expressed |
| Os.7613.1.S1_at | LOC_Os06g47830 | 0.47 | OB-fold nucleic acid binding domain containing protein, expressed |
| Os.18818.1.S1_a_at | LOC_Os01g12710 | 0.48 | oxidoreductase, short chain dehydrogenase/reductase family protein, expressed |
| Os.28438.1.S1_x_at | LOC_Os01g31690 | 0.08 | Oxygen-evolving enhancer protein 1, chloroplast precursor, putative, expressed |
| Os.12627.1.S1_at | LOC_Os07g04840 | 0.13 | Oxygen-evolving enhancer protein 2, chloroplast precursor, putative, expressed |
| Os.11078.1.S1_a_at | LOC_Os07g36080 | 0.14 | Oxygen-evolving enhancer protein 3-1, chloroplast precursor, putative, expressed |
| Os.28976.1.S1_at | LOC_Os01g10800 | 0.46 | pentatricopeptide, putative, expressed |
| Os.36369.1.S1_at | LOC_Os06g27760 | 0.18 | Peptide methionine sulfoxide reductase msrB |
| Os.14368.1.S1_at | LOC_Os08g29370 | 0.32 | Peptidyl-prolyl cis-trans isomerase, chloroplast precursor, putative, expressed |
| Os.11841.1.S1_at | LOC_Os08g45190 | 0.22 | PGR5, putative, expressed |
| Os.9506.1.S1_a_at | LOC_Os02g38020 | 0.24 | Phosphate transporter family protein, expressed |
| Os.6248.1.S1_s_at | LOC_Os08g27840 | 0.38 | Phosphoenolpyruvate carboxylase 2, putative, expressed |
| OsAffx.29501.1.S1_at | LOC_Os08g34050 | 0.49 | phosphofructokinase family protein, putative, expressed |
| Os.5319.1.S1_at | LOC_Os10g11140 | 0.42 | Phosphoglucomutase, chloroplast precursor |
| Os.12371.1.S1_at | LOC_Os04g41340 | 0.35 | phosphoglycolate/pyridoxal phosphate phosphatase family protein, expressed |
| Os.26952.1.S1_at | LOC_Os01g66500 | 0.50 | phosphoribosylformylglycinamidine synthase, chloroplast precursor, putative, expressed |
| Os.46383.2.A1_s_at | LOC_Os02g47020 | 0.11 | Phosphoribulokinase, chloroplast precursor |
| Os.7974.1.S1_at | LOC_Os08g44680 | 0.34 | Photosystem I reaction center subunit II, chloroplast precursor, putative, expressed |
| Os.12283.1.S1_at | LOC_Os03g56670 | 0.17 | Photosystem I reaction center subunit III, chloroplast precursor, putative, expressed |
| Os.12056.1.S1_at | LOC_Os07g05480 | 0.18 | Photosystem I reaction center subunit psaK, chloroplast precursor, putative, expressed |
| Os.10136.1.S1_at | LOC_Os05g48630 | 0.06 | Photosystem I reaction center subunit VI, chloroplast precursor, putative, expressed |
| Os.46422.1.S1_at | LOC_Os07g25430 | 0.23 | Photosystem I reaction centre subunit IV/PsaE family protein, expressed |
| OsAffx.27508.126.S1_x_at | LOC_Os12g08770 | 0.09 | Photosystem I reaction centre subunit N, chloroplast precursor, putative, expressed |
| Os.28301.1.S1_at | LOC_Os07g05360 | 0.24 | Photosystem II 10 kDa polypeptide, chloroplast precursor, putative, expressed |
| Os.7962.1.S1_a_at | LOC_Os08g10020 | 0.07 | Photosystem II 10 kDa polypeptide, chloroplast precursor, putative, expressed |
| Os.25590.1.S1_at | LOC_Os01g71190 | 0.26 | Photosystem II reaction center W protein |
| Os.5378.1.S1_at | LOC_Os01g56680 | 0.33 | Photosystem II reaction centre W protein containing protein, expressed |
| Os.10370.2.S1_at | LOC_Os07g47640 | 0.12 | Photosystem II reaction centre X protein containing protein, expressed |
| Os.6590.1.S1_at | LOC_Os03g22370 | 0.08 | Photosystem II reaction centre X protein containing protein, expressed |
| Os.7931.1.S1_s_at | LOC_Os06g51290 | 0.30 | Phytoene synthase, chloroplast precursor, putative, expressed |
| Os.5684.1.S1_at | LOC_Os07g09340 | 0.46 | Plasma membrane ATPase 1, putative, expressed |
| Os.7915.1.S1_a_at | LOC_Os06g01210 | 0.27 | Plastocyanin, chloroplast precursor, putative, expressed |
| Os.11673.2.S1_x_at | LOC_Os01g56780 | 0.06 | Plus-3 domain containing protein, expressed |
| Os.9303.1.S1_at | LOC_Os02g46460 | 0.33 | POT family protein, expressed |
| Os.13534.1.S1_at | LOC_Os06g44840 | 0.47 | prenyltransferase, UbiA family protein, expressed |
| Os.12266.1.S1_at | LOC_Os05g06780 | 0.31 | protease inhibitor/seed storage/lipid transfer protein, putative, expressed |
| Os.27759.1.S1_at | LOC_Os01g46720 | 0.48 | Protein kinase domain containing protein, expressed |
| Os.33127.2.S1_x_at | LOC_Os01g07940 | 0.41 | Protein kinase domain containing protein, expressed |
| Os.9230.1.S1_x_at | LOC_Os10g35370 | 0.20 | Protochlorophyllide reductase B, chloroplast precursor, putative, expressed |
| Os.14369.2.S1_at | LOC_Os08g29170 | 0.49 | Quinone oxidoreductase, putative, expressed |
| Os.51021.1.S1_at | LOC_Os04g52920 | 0.43 | Remorin, C-terminal region family protein, expressed |
| Os.49670.1.S1_at | LOC_Os02g02500 | 0.36 | Remorin, C-terminal region family protein, expressed |
| Os.19872.1.S1_at | LOC_Os02g38240 | 0.41 | rhodanese family protein, putative, expressed |
| Os.18520.1.S1_at | LOC_Os09g10750 | 0.15 | rhodanese-like domain-containing protein, putative, expressed |
| Os.12594.1.S1_x_at | LOC_Os12g17600 | 0.15 | Ribulose bisphosphate carboxylase small chain C, chloroplast precursor, putative, expressed |
| Os.10959.1.S1_at | LOC_Os12g19470 | 0.05 | Ribulose bisphosphate carboxylase small chain C, chloroplast precursor, putative, expressed |
| OsAffx.12575.1.S1_at | LOC_Os02g50330 | 0.38 | RNA-directed RNA polymerase 2, putative, expressed |
| Os.48075.1.S1_at | LOC_Os03g62780 | 0.35 | S1 RNA binding domain containing protein, expressed |
| Os.9500.1.S1_s_at | LOC_Os07g32170 | 0.43 | SBP domain containing protein, expressed |
| Os.6636.1.S1_at | LOC_Os08g39890 | 0.32 | SBP-domain protein 5, putative, expressed |
| Os.18552.1.S1_at | LOC_Os11g31980 | 0.40 | Serine carboxypeptidase family protein, expressed |
| Os.2422.1.S1_a_at | LOC_Os07g07540 | 0.25 | SHOOT1 protein, putative, expressed |
| Os.27634.1.S1_at | LOC_Os08g14450 | 0.11 | Sigma-70 region 2 family protein, expressed |
| Os.32177.1.S1_at | LOC_Os05g50930 | 0.19 | Sigma-70, region 4 family protein, expressed |
| Os.9306.1.S1_at | LOC_Os01g11230 | 0.32 | SOUL, putative, expressed |
| Os.4600.1.S1_at | LOC_Os06g33710 | 0.43 | Spermidine synthase 2, putative, expressed |
| OsAffx.13633.1.S1_s_at | LOC_Os04g03060 | 0.18 | Subtilase family protein, expressed |
| Os.27076.1.S1_at | LOC_Os06g05160 | 0.13 | sulfate transporter 3.4, putative, expressed |
| Os.54132.1.S1_at | LOC_Os04g09800 | 0.40 | SWI/SNF-related matrix-associated actin-dependent regulator of chromatin subfamily A member 3-like 3, putative |
| Os.19844.1.S1_at | LOC_Os03g57190 | 0.32 | TCP family transcription factor containing protein |
| Os.9779.1.S1_at | LOC_Os01g32730 | 0.43 | tetratricopeptide repeat, putative, expressed |
| Os.18490.3.S1_at | LOC_Os03g47610 | 0.30 | Thiamine biosynthesis protein thiC, putative, expressed |
| Os.12100.1.S1_at | LOC_Os07g29410 | 0.24 | thioredoxin family protein, putative, expressed |
| Os.9880.1.S1_a_at | LOC_Os01g68480 | 0.42 | Thioredoxin F-type 2, chloroplast precursor |
| Os.38257.1.S1_x_at | LOC_Os12g08730 | 0.26 | Thioredoxin M-type, chloroplast precursor |
| Os.22795.1.S1_at | LOC_Os02g33500 | 0.48 | Threonyl-tRNA synthetase, putative, expressed |
| Os.22683.1.S1_at | LOC_Os06g49160 | 0.36 | thylakoid lumenal 16.5 kDa protein, chloroplast precursor, putative, expressed |
| Os.53453.1.S1_at | LOC_Os12g43940 | 0.27 | TPR Domain containing protein, expressed |
| Os.57558.1.S1_at | LOC_Os04g51000 | 0.26 | Transcription factor FL, putative, expressed |
| Os.46988.1.A1_at | LOC_Os07g45250 | 0.42 | transposon protein, putative, Mutator sub-class, expressed |
| Os.19899.1.S1_x_at | LOC_Os12g42190 | 0.10 | transposon protein, putative, unclassified, expressed |
| Os.38110.1.S1_at | LOC_Os10g37660 | 0.49 | Trehalase family protein, expressed |
| Os.57080.1.S1_at | LOC_Os12g22600 | 0.38 | tRNA synthetases class II family protein, expressed |
| Os.33770.1.S1_s_at | LOC_Os01g59500 | 0.46 | U3 small nucleolar RNA-associated protein 11 |
| Os.25227.2.S1_at | LOC_Os01g53460 | 0.49 | UDP-glucoronosyl and UDP-glucosyl transferase family protein, expressed |
| Os.5771.1.S1_a_at | unknown | 0.48 | unknown |
| Os.33894.2.A1_at | unknown | 0.47 | unknown |
| Os.11908.1.S1_s_at | unknown | 0.39 | unknown |
| Os.6205.2.S1_at | unknown | 0.38 | unknown |
| OsAffx.5613.1.S1_x_at | unknown | 0.36 | unknown |
| Os.51732.1.S1_at | unknown | 0.33 | unknown |
| Os.34206.1.S1_at | unknown | 0.32 | unknown |
| Os.51597.1.S1_at | unknown | 0.31 | unknown |
| Os.28133.1.S1_x_at | unknown | 0.31 | unknown |
| Os.13866.1.S1_at | unknown | 0.30 | unknown |
| Os.27387.1.S1_x_at | unknown | 0.28 | unknown |
| Os.5412.1.S1_at | unknown | 0.27 | unknown |
| OsAffx.24336.1.S1_x_at | unknown | 0.24 | unknown |
| Os.27615.1.S1_at | unknown | 0.16 | unknown |
| OsAffx.11552.1.S1_at | unknown | 0.15 | unknown |
| Os.12239.1.S1_at | unknown | 0.06 | unknown |
| Os.29051.1.S1_at | LOC_Os03g17600 | 0.44 | UPF0308 protein, chloroplast precursor, putative, expressed |
| Os.53486.1.S1_at | LOC_Os02g43560 | 0.29 | WRKY family transcription factor, putative, expressed |
| Os.2293.1.S1_at | LOC_Os03g44710 | 0.33 | YABBY protein, putative, expressed |
| Os.48725.1.S1_at | LOC_Os04g45990 | 0.47 | YDG/SRA domain containing protein, expressed |
| Os.57347.1.S1_at | LOC_Os05g50310 | 0.48 | ZF-HD protein dimerisation region containing protein |
| Os.18572.1.S1_at | LOC_Os04g52310 | 0.24 | ZIP zinc/iron transport family protein, expressed |
