# Supplementary material for: Identification of rhizome-specific genes by genome-wide differential expression Analysis in Oryza longistaminata
Source: BMC Plant Biol. 2011 Jan 24;11:18. doi: 10.1186/1471-2229-11-18 (PMC3036607; doi:10.1186/1471-2229-11-18)
Supplement: Additional file 9 — Rhizome-specific genes located in the genomic regions of QTLs for rhizome-related traits identified in both rice and sorghum. Word file for the list of rhizome-specific expressed genes associated with the previously mapped QTLs related to rhizome abundance and length. [file 1471-2229-11-18-S9.DOC]

**Additional file 9**. Rhizome-specific genes located in the genomic regions of QTLs for rhizome-related traits identified in both rice and sorghum

| **Rice rhizome QTLs ^a^** | **RT/ST ^b^** | **TIGR** | **NCBI Annotation** | **Sorghum transcript**  **Name** | **Corresponding QTL intervals in the sorghum genome ^c^** |
| --- | --- | --- | --- | --- | --- |
| ***QRl1*** | 0.48 | LOC_Os01g46720 | Protein kinase domain containing protein. | Sb03g029870.1 |  |
|  |  |  |  |  |  |
| ***QRbd2*** | 0.34 | LOC_Os02g17620 | Isochorismatase hydrolase family protein. | Sb04g010640.1 | F: pSB193-pSB341* |
|  | 0.32 | LOC_Os02g18450 | GTP-binding protein TypA family protein. | Sb04g011160.1 | F: pSB193-pSB341* |
|  | 0.24 | LOC_Os02g19924 | Conserved hypothetical protein. |  |  |
|  |  |  |  |  |  |
| ***QRn2*** | 2.30 | LOC_Os02g32610 | **MAP3K delta-1 protein kinase** | Sb04g021500.1 | F: pSBO38-pSB512 |
|  |  |  |  |  |  |
| ***Rhz2*** | 0.16 | LOC_Os03g11734 | Multi antimicrobial extrusion protein  MatE family protein. | Sb01g042740.1 | C: pSB300a-pSBO88;C: pSB300a-pSBO88*;C: pSB300-pSBO88 |
|  |  |  |  |  |  |
| ***QRn3*** | RT | LOC_Os03g21820 | Expansin S1. | Sb09g023440.1 |  |
|  | 3.90 | LOC_Os03g22210 | Hypothetical protein. |  |  |
|  | 3.90 | LOC_Os03g22230 | Hypothetical protein. |  |  |
|  | 0.08 | LOC_Os03g22370 | Photosystem II protein PsbX family protein. | Sb02g042680.1 |  |
|  |  |  |  |  |  |
| ***Rhz3*** | RT | LOC_Os04g35520 | Stromal ascorbate peroxidase. | Sb06g017080.1 | D: pSBO95-pSB428;D: pSB188-pSB428 |
|  | 5.24 | LOC_Os04g36670 | Conserved hypothetical protein. |  |  |
|  | 0.43 | LOC_Os04g36800 | 3-oxoacyl-[acyl-carrier-protein] synthase I | Sb10g006430.1 |  |
|  | 0.20 | LOC_Os04g38410 | Chlorophyll a/b-binding protein CP24, photosystem II | Sb06g032690.1 | D: pSBO95-pSB428;D: pSB188-pSB428 |
|  | 0.25 | LOC_Os04g38720 | **No apical meristem (NAM) protein** | Sb06g019010.1 | D: pSBO95-pSB428;D: pSB188-pSB428 |
|  | 2.32 | LOC_Os04g39020 | Betaine-aldehyde dehydrogenase(BADH). | Sb06g019210.1 | D: pSBO95-pSB428;D: pSB188-pSB428 |
|  |  |  |  |  |  |
| ***QRn5*** | 0.46 | LOC_Os05g34980 | Amino acid carrier. | Sb09g020790.1 |  |
|  | 0.33 | LOC_Os05g38530 | Heat shock protein 70. | Sb09g022580.1 |  |
|  | 2.90 | LOC_Os05g40010 | Nonspecific lipid-transfer protein 1 (LTP 1) |  |  |
|  | 2.68 | LOC_Os05g41990 | Peroxidase precursor | Sb09g024580.1 |  |
|  | 3.60 | LOC_Os05g44200 | Lipolytic enzyme, G-D-S-L family protein. | Sb09g025780.1 |  |
|  | RT | LOC_Os05g44210 | SL-TPS/P. | Sb09g025790.1 |  |
|  |  |  |  |  |  |
| ***QRl6*** | 6.40 | LOC_Os06g44250 | Hly-III related proteins family protein. | Sb10g025870.1 | I: pSB106-pSB430a |
|  |  |  |  |  |  |
| ***QRn6*** | RI | LOC_Os06g50230 | Heat shock protein DnaJ family protein. | Sb10g030200.1 | I: pSB106-pSB430a |
|  | 0.30 | LOC_Os06g51290 | Phytoene synthase. |  |  |
|  | 2.24 | LOC_Os06g51320 | Gibberellin-regulated protein 2 precursor. |  |  |
|  |  |  |  |  |  |
| ***QRl7*** | 0.32 | LOC_Os07g37030 | Rieske FeS protein precursor. | Sb09g020820.1 |  |
|  | 0.11 | LOC_Os07g37240 | Chlorophyll a/b-binding protein CP29 precursor. | Sb02g036260.1 |  |
|  | 0.09 | LOC_Os07g37550 | Type III chlorophyll a/b-binding protein (Fragment). | Sb03g036380.1 |  |
|  | 3.59 | LOC_Os07g37850 | Conserved hypothetical protein. |  |  |
|  | 3.63 | LOC_Os07g39320 | Homeodomain leucine zipper protein CPHB-4. | Sb02g037560.1 |  |
|  | 0.46 | LOC_Os07g39980 | Hypothetical protein. | Sb02g038020.1 |  |
|  | RI | LOC_Os07g41580 | Histone-like transcription factor | Sb01g032590.1 | C: pSB300a-pSBO88;C: pSB300a-pSBO88*;C: pSB300-pSBO88 |
|  |  |  |  |  |  |
| ***QRn10*** | RT | LOC_Os10g31930 | Conserved hypothetical protein. |  |  |
|  | 2.36 | LOC_Os10g33370 | Naringenin-chalcone synthase family protein. | Sb01g019520.1 | C: pSB102-pSB158* |

^a^ Rhizome QTLs identified in the F_2_ population between RD23 (*O. sativa*) and *O. longistaminata* [11].

^b^ The ratio of the expression level in root tips (RT) over that in the shoot tips (ST), RT or RI indicates genes specifically expressed in RT or root internodes (RI).

^c^ Sorghum rhizome QTLs identified in the interspecific F_2_ population of *S. propinquum* [1,2].
