# Supplementary material for: Identification of rhizome-specific genes by genome-wide differential expression Analysis in Oryza longistaminata
Source: BMC Plant Biol. 2011 Jan 24;11:18. doi: 10.1186/1471-2229-11-18 (PMC3036607; doi:10.1186/1471-2229-11-18)
Supplement: Additional file 10 — Primer list for the RT-PCR analysis used for identification of gene expression pattern detected by microarray analysis. Word file for the list of PCR primers used for identification of gene expression pattern by semi-quantitative reverse transcription. [file 1471-2229-11-18-S10.DOC]

Additional file 10. Primer list for the RT-PCR analysis used for identification of gene expression pattern detected by microarray analysis

| **Gene Code** | **Primer** | **Sequence 5’-3’** |
| --- | --- | --- |
| LOC_Os10g40610 | RI1F | TCAAATGTTGCCAAGGAAGTG |
|  | RI1R | CGATACCCTGTGCAGTAAAGAAAG |
| LOC_Os01g58290 | RI4F | TACTCTTGTCACTGCCTCAC |
|  | RI4R | CTGCTTGCTCCTCTGTTAG |
| LOC_Os07g41580 | RI5F | ATCGACCCGCTCAAGCTC |
|  | RI5R | CTTTCCCTCCCATCACCA |
| LOC_Os09g25420 | RI6F | CTTGGGTATTTGCTTGGTG |
|  | RI6R | CGAAAGTGGCAGGATTGG |
| LOC_Os01g60770 | RI3F | TAGTGCCCTGCTCCTCCT |
|  | RI3R | GCAGGGCACTACTGCGTGAGGC |
| LOC_Os07g07540 | YL1F | GCAGGGTACGGACAGACCA |
|  | YL1R | CAGCAGCACCATCCCATTT |
| LOC_Os08g45190 | YL4F | AGGGCTCTCCCGACGTGGTCC |
|  | YL4R | GTGCAGCGCGATGGCCTTTCC |
| LOC_Os03g29680 | YL3F | TTCGTGCAGGTGCAGAGCTTGCTG |
|  | YL3R | ATGAGGCCGACGTTGCGGGAGA |
| LOC_Os06g05060 | YL5F | TTTATGGTTCCTTCTGTC |
|  | YL5R | GGATTCTCTCCAATTTCT |
| LOC_Os05g45910 | YL2F | GGCGGCTTCTTCCTCTAC |
|  | YL2R | TCTCGCTGGTTTTCTGGT |
| LOC_Os11g10590 | RT1F | ATGGCGAGCACTAAGATGGCG |
|  | RT1R | CCGTAGCAAACCCCGACAGGA |
| LOC_Os04g17660 | RT6F | TAGCATCTGCCGATCTCAT |
|  | RT6R | GGTTTATTCACCGCAAGG |
| LOC_Os03g21820 | RT7F | GCGTCAGGTTCACCGTCAAC |
|  | RT7R | CGCCTTGCCGTCGAGGTA |
| LOC_Os03g51690 | RT9F | GAGGAGGACCAAGAAGGTAGCGG |
|  | RT9R | CACCAGTTGAGGAGCTGTTGACGAG |
| LOC_Os04g33570 | RT2F | CTTTGGGAGGGAGGTGGTGAG |
|  | RT2R | GCTTGAACAGCACGAGGACGA |
| LOC_Os11g05470 | RT5F | GGGTGACCTGCGATCTTTCTTC |
|  | RT5R | TTACAGTTTGCCTGCGCTTCTG |
| LOC_Os08g02070 | RT8F | ATCATCTTCTCCGCCCACG |
|  | RT8R | GAGCACCATTGCCTCCTG |
| LOC_Os01g18290 | RT12F | AAGGAGGAGAAGGGACAGGAT |
|  | RT12R | TGCCGAGATGAGGGAAATG |
| LOC_Os04g58200 | SI1F | ACACCGTCATGCACCTGGACC |
|  | SI1R | GGTAGTCGGATTTCTTGAGGTCGT |
| LOC_Os01g61500 | SI3F | GGCTTGTGAGGCTGTTGG |
|  | SI3R | TCCTTTGTGCCCTTGCTT |
| LOC_Os08g36320 | SI5F | AGGTGTTGGTTGGGTCATT |
|  | SI5R | GCCTTCCCTTAGTATTGCTGT |
| *Actin* | Actin7F | TTATGGTTGGGATGGGACA |
|  | Actin7R | AGCACGGCTTGAATAGCG |
